# Targeting MDM2–p53 Axis through Drug Repurposing for Cancer Therapy: A Multidisciplinary Approach

**DOI:** 10.1021/acsomega.3c03471

**Published:** 2023-09-15

**Authors:** Naeem Abdul Ghafoor, Aysegul Yildiz

**Affiliations:** † Department of Molecular Biology and Genetics, Graduate School of Natural and Applied Sciences, Mugla Sitki Kocman University, 48000 Mugla, Turkey; ‡ Department of Molecular Biology and Genetics, Faculty of Science, Mugla Sitki Kocman University, 48000 Mugla, Turkey

## Abstract

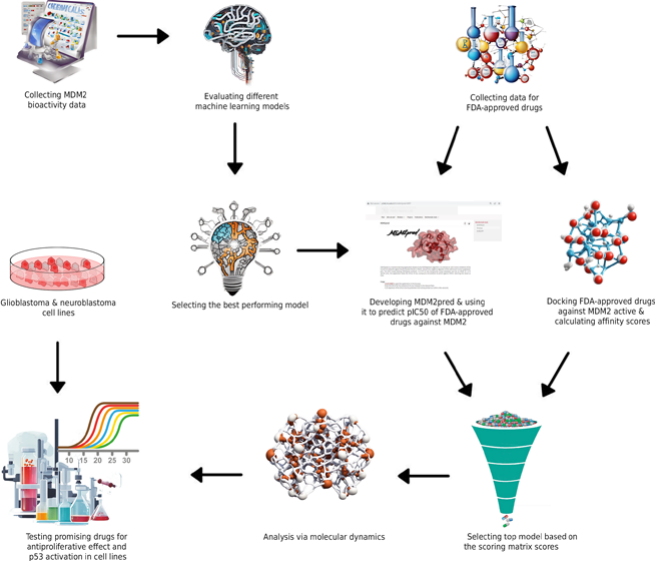

Cancer remains a major cause of morbidity and mortality worldwide, and while current therapies, such as chemotherapy, immunotherapy, and cell therapy, have been effective in many patients, the development of novel therapeutic options remains an urgent priority. Mouse double minute 2 (MDM2) is a key regulator of the tumor suppressor protein p53, which plays a critical role in regulating cellular growth, apoptosis, and DNA repair. Consequently, MDM2 has been the subject of extensive research aimed at developing novel cancer therapies. In this study, we employed a machine learning-based approach to establish a quantitative structure–activity relationship model capable of predicting the potential in vitro efficacy of small molecules as MDM2 inhibitors. Our model was used to screen 5883 FDA-approved drugs, resulting in the identification of promising hits that were subsequently evaluated using molecular docking and molecular dynamics simulations. Two antihistamine drugs, cetirizine (CZ) and rupatadine (RP), exhibited particularly favorable results in the initial in silico analyses. To further assess their potential use as the activators of the p53 pathway, we investigated the antiproliferative capability of the abovementioned drugs on human glioblastoma and neuroblastoma cell lines. Both the compounds exhibited significant antiproliferative effects on the abovementioned cell lines in a dose-dependent manner. The half-maximal inhibitory concentration (IC_50_) of CZ was found to be 697.87 and 941.37 μM on U87 and SH-SY5Y cell lines, respectively, while the IC_50_ of RP was found to be 524.28 and 617.07 μM on the same cell lines, respectively. Further investigation by quantitative reverse transcriptase polymerase chain reaction analysis revealed that the CZ-treated cell lines upregulate the expression of the p53-regulated genes involved in cell cycle arrest, apoptosis, and DNA damage response compared to their respective vehicle controls. These findings suggest that CZ activates the p53 pathway by inhibiting MDM2. Our results provide compelling preclinical evidence supporting the potential use of CZ as a modulator of the MDM2–p53 axis and its plausible repurposing for cancer treatment.

## 1 Introduction

According to recent data published by the American Cancer Society, an estimated 1,958,310 new cases of cancer and 609,820 cancer-related fatalities are projected to occur in the United States in 2023.^1^ A sizable proportion of these cases, ranging from 30 to 50%, may be avoided by adopting preventive measures, including abstaining from alcohol and tobacco use and various other cancer prevention approaches. For the remaining cases, clinical interventions such as radiotherapy, chemotherapy, or immunotherapy are required. Cancer formation can be characterized by the uncontrolled proliferation of healthy cells. Unlike normal cells, cancerous cells divide and grow uncontrollably without the usual constraints on their proliferation and growth mechanisms.^2^

Mutations are among the primary factors implicated in cancer initiation and progression. These mutations can arise from various sources, including exposure to ionizing radiation, hypoxia, smoking, and certain viral infections. Additionally, during natural DNA replication under normal cellular conditions, DNA is continuously subjected to mutations caused by increased salt concentration and oxidative stress, leading to the formation of reactive oxygen species (ROS). Such damage is actively and dynamically corrected through DNA damage and repair mechanisms. When the DNA damage and repair elements fail to repair the DNA damage resulting from random mutations, cell proliferation is arrested, and the cells are directed to apoptosis through the activity of apoptotic pathways. However, mutations in apoptotic mechanisms enable these cells to avoid apoptosis due to dysfunctional apoptotic elements, leading to the onset of cancer etiology. Basic DNA damage and repair mechanisms include nucleotide excision repair, base excision repair, mismatch repair, homologous recombination repair, and non-homologous end joining. Different combinations of these pathways operate at different stages of the cell cycle, and at each stage, various DNA damage and repair elements trigger the activation of the genes and signaling pathways crucial in maintaining genome integrity and preventing the onset of cancer and related conditions.^3^ Most anticancer drugs function by compensating for DNA damage and repair mechanism failure, either by blocking DNA replication or by inhibiting the enzymes or pathways essential for cell survival, thereby driving otherwise a cancerous cell to undergo apoptosis.^4^

**Figure 1:**
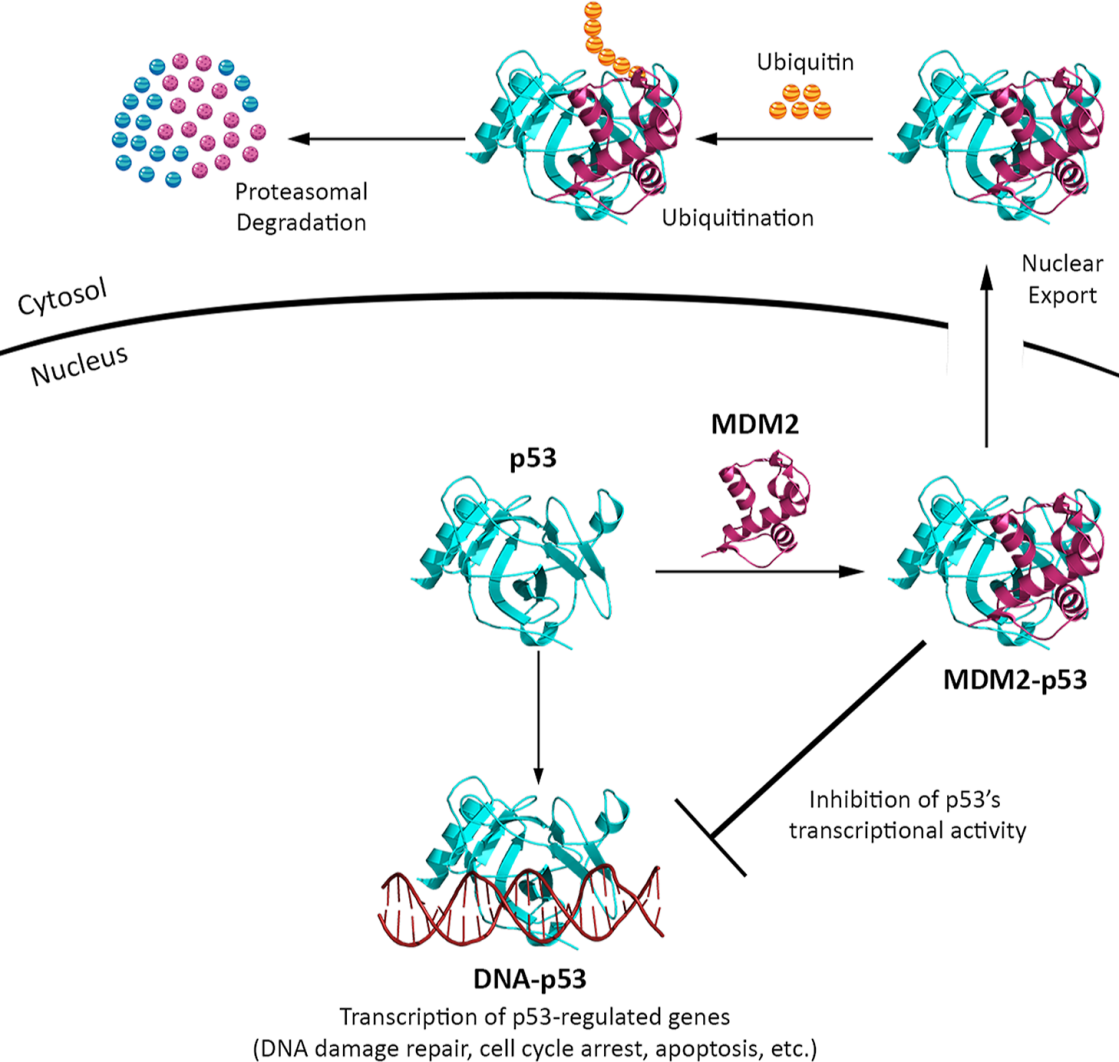
Mechanism of regulation of the p53 transcription factor by MDM2. In response to cellular stress signals, the MDM2 (magenta) protein interacts with the p53 (cyan) transcription factor in various ways to negatively modulate its activity. Upon binding to p53, MDM2 facilitates the translocation of p53 from the nucleus to the cytoplasm by utilizing a nuclear export signal sequence and promotes the ubiquitination and subsequent degradation of p53 via its ubiquitin ligase activity. In the absence of MDM2 binding, p53 interacts with the genomic DNA (dark red) and activates the downstream genes.

Cancer molecular genetics research has identified the transcription factor p53, which is encoded by the *TP53* gene, as a prominent element in cancer development. Acting as the “genome savior”, p53 serves as a tumor suppressor by regulating a variety of genes. It responds to stresses such as DNA damage, hypoxia, and activation of oncogenes by becoming activated, and once activated, it suppresses cell division by acting as a transactivator for various downstream genes. Genome-wide studies have identified approximately 3509 genes potentially regulated by p53. These genes are involved in a wide range of cellular processes including cell cycle arrest, DNA repair, apoptosis, metabolism, autophagy, mRNA translation, and feedback mechanisms.^5^ The activity of p53 is regulated by the Murine double minute 2 (MDM2; also known as E3 ubiquitin-protein ligase) protein, which acts as a negative modulator of p53. MDM2 is transcriptionally activated by p53; however, it then inhibits p53 activity in various ways. MDM2 contains a signal sequence similar to the nuclear export signals of some viral proteins. After binding to p53, MDM2 uses this sequence to transport p53 from the nucleus to the cytoplasm through the nuclear pore complex. However, because p53 is a transcription factor, it must remain in the nucleus to access genomic DNA for its functioning. MDM2 is also a ubiquitin ligase enzyme that can tag p53 with ubiquitin and thereby traffic it to ubiquitin-dependent proteasomal degradation. Under normal cellular conditions, MDM2 continuously and dynamically degrades p53 and maintains it at a low level. However, when the cells experience stress signals such as DNA damage, ribosomal stress, oncogene activation, and hypoxia, MDM2’s interaction with p53 is downregulated, and p53 is activated, resulting in the expression of the p53-regulated genes.^6,7^ This regulatory mechanism is schematically illustrated in Figure 1. The negative modulatory effect of MDM2 on p53 is a key point of interest in the study of the cancers with wild-type p53. One of the first group of MDM2 inhibitors was a series of *cis*-imidazolines named nutlins (named after the US town of Nutley, where they were first identified), which were first identified in 2004, and several compounds derived from the series have been investigated ever since. However, to date, no FDA-approved drug has targeted MDM2.^8^ Nevertheless, several drug candidates are in clinical trials, including nutlins and its derivatives, such as ALRN-6924, SAR405838, NVP-CGM097, MK-8242, RG7112, RG7388, DS-3032b, and AMG232.^9−11^ These drugs are all based on the MDM2 inhibition mechanism and have shown promising results in preclinical and early clinical trials, suggesting the potential of targeting the MDM2–p53 axis as a promising therapeutic strategy for cancer treatment.

Mutations in the *TP53* gene are prevalent in approximately 50–60% of human cancers, with the majority of these being homozygous missense mutations affecting approximately 190 codons within the DNA-binding domain of the gene. These mutations lead to a reduction in the ability of the resulting mutant p53 protein to bind to its specific DNA sequence, which is responsible for regulating the transcriptional pathway of p53.^12^ Neuroblastoma is a malignant neoplasm that originates from undifferentiated nerve cells. In the majority of cases, wild-type p53 with intact transcriptional activity is detected in most neuroblastomas, and the *TP53* mutation rate does not exceed 2%. Notably, MDM2 overexpression is common in a neuroblastoma, which subsequently leads to p53 inhibition. This makes the targeting of the MDM2–p53 axis in these types of cancers especially favorable.^13^ Glioblastoma is a highly aggressive cancer that originates in astrocytes and is associated with an average survival rate of 12–15 months. *TP53* mutations are observed in up to 30% of primary glioblastomas, and the *TP53* mutation status is closely linked to the disease progression and survival rates of the patients with glioblastoma who receive radiotherapy and chemotherapy.^14^

The development of new drugs is a costly and time-consuming process with an estimated cost of 2–3 billion USD. The attrition rates in the field of anticancer drug development remain a significant challenge as up to 95% of drugs assessed in phase I trials in oncology do not receive marketing authorization later on. To address these issues, drug repurposing has emerged as an attractive option for the development of new cancer treatments. This approach involves the use of existing drugs that have been approved for other indications or in other words, de-risked compounds (i.e., compounds with known safety profiles and hence reduced risk of side effects and adverse drug reactions). Drug repurposing has the potential to reduce costs and shorten the development time of new cancer treatments. Several examples of successful drug repurposing include the pre-clinical use of Raltegravir, an antiviral drug, in cancer treatment; Astemizole, an antihistamine drug, in prostate cancer treatment; Ketorolac, an anti-inflammatory drug, in ovarian cancer treatment; and phenothiazines, an antipsychotic drug, in inflammation and cancer treatment.^15−17^ This concept can be combined with data science techniques, such as machine learning, to further enhance the drug repurposing pipeline. Quantitative structure–activity relationship (QSAR) is a computational modeling method that reveals the relationships between the biological activities and structural properties of chemical compounds. The changes in the structural properties lead to different biological activities, and these features determine the pharmacokinetic properties, such as absorption, distribution, and metabolism or even biological properties, such as half-maximal inhibitory concentration (IC_50_), half maximal effective concentration (EC_50_), and dissociation constant (Kd). Using sophisticated machine learning algorithms, the QSAR models can predict the biological activity, toxicity, and pharmacokinetic properties of drug candidates prior to them being tested in vitro. The QSAR has emerged as a valuable computational tool for drug discovery by enabling the prediction of the activity of existing drugs against new therapeutic targets. By analyzing the chemical structure of a drug and its known biological properties, the QSAR models can predict the biological properties of a drug with an unknown label. This approach can significantly reduce the time and cost associated with drug repurposing, enabling researchers to identify new therapeutic uses for existing drugs more efficiently. This can accelerate the drug development process and improve the success rate of drug discovery programs, potentially transforming drug discovery and development.^18,19^

The present study aimed to re-evaluate FDA-approved drugs targeting the MDM2-p53 mechanism for the treatment of cancers with wild-type p53. The project intends to offer novel treatment options for wild-type p53 cancers by integrating data science methods with structural bioinformatics and molecular biology to streamline the drug discovery and development process with an economical and time-efficient approach by implementing a multi-disciplinary approach.

## 2 Materials and Methods

### 2.1 Developing the QSAR Model

#### 2.1.1 Preparing the MDM2 Inhibitor Datasets

To develop the QSAR model for MDM2 inhibitor prediction, a compound library was constructed from the ChEMBL30 database.^20,21^ Specifically, we identified compounds with known bioactivity against MDM2 based on their reported IC_50_ values (IC_50_ refers to the concentration of a given drug or inhibitor required to inhibit a biological process or response by 50%, a metric that is commonly used in drug discovery and development and provides a useful means of quantifying a compound’s potency) and curated their corresponding simplified molecular-input line-entry system (SMILES) strings, as well as publication information including the journal name and year of publication. Duplicate entries in the dataset were removed in the order of their occurrence (i.e., the latest reported entry was kept in case of repetition). The SMILES strings reported with non-complexed metals (salts) were cleared from it, and negative log transformation was applied to the IC_50_ (in molar unit) values to obtain the pIC_50_ values. Finally, the datapoints with molecular weight, LogP (logarithm of the partition coefficient, a measure of the lipophilicity or hydrophobicity, of a molecule), and pIC_50_ values greater than ±1.5 standard deviations from each column mean were removed. The final dataset consisted of 1647 compounds and is provided in Supporting Information Data 1 (S1).

#### 2.1.2 Featurizing and Selecting the Best Machine Learning Algorithm

The SMILES strings from the dataset of compounds were used to compute the molecular descriptors via the *mol2vec* algorithm.^22^*Mol2vec* is an unsupervised machine learning method inspired by natural language processing techniques and learns the vector representations of molecular substructures. The resulting model yields dense vector representations that can be used as vectors to predict compound properties by encoding the compounds as vectors through the summation of individual substructure vectors. In this study, the pre-trained *mol2vec* model (based on 20 million compounds downloaded from the ZINC database) was used to generate a 300 features/SMILES string.

Using the *Scikit-learn* library in a Python environment, regression-based QSAR models were developed with random forest, k-neighbors, extra trees, light gradient boosting, histogram-based gradient boosting, extreme gradient boosting, decision tree, stochastic gradient descent, multilayer perceptron, and adaptive boosting regressor algorithms.^23,24^ The model developed using each algorithm was evaluated by *k*-fold cross validation (*k* = 10); briefly, the entire dataset was split randomly into 10 parts, and 10 iterations of model building-testing was performed. In each iteration, the model was trained on 9 parts of the constructed dataset and evaluated on the remaining 1 part, and the scores of each iteration were recorded. The performance of each algorithm was evaluated based on the average coefficient of determination (*R*^2^), mean squared error (MSE), mean absolute error (MAE), and root mean squared error (RMSE) obtained from the 10-fold cross-validation.

The algorithm that yielded the highest *R*^2^ was pre-selected, and its hyperparameters were determined via the “GridSearchCV” module from *Scikit-learn*. The hyperparameter search for the K-neighbors model was performed for the “leaf_size” (5–50, with increments of 5), “n_neighbors” (2–32 with increments of 1), “p” (1–10, with increments of 1), and “weights” (uniform and distance) hyperparameters. The hyperparameterized model was then serialized using the “Pickle” module to enable its deployment in a Python environment. The model was also deployed as a web application through the *Streamlit* library and was made available online for public use.

#### 2.1.3 Analyzing the Top Model’s Applicability Domain and Robustness

The estimation of the applicability domain (AD), which defines the chemical space where the developed QSAR model can reliably predict is very important in QSAR modeling to ensure the robustness and reliability of a model’s predictions. To assess the AD of the developed model with the best performance, we utilized the Hotelling’s test and its associated leverage statistics (collectively referred to as the leverage approach) as recommended by the Organization for Economic Co-operation and Development (OECD) under their guiding principles for QSAR modeling.^25^ To visualize the AD of the QSAR model, we plotted the standardized cross-validated residuals (RES) against the leverage values (Hat matrix diagonal, a value that represents the influence of each compound in the dataset on a regression model), which is also referred to as the Williams plot.

To establish the limits of the normal values, horizontal and vertical straight lines were incorporated into the plot. The horizontal line indicates the threshold for identifying Y outliers (outliers in the response variable), i.e., the compounds with cross-validated standardized residuals exceeding ± 2.5 standard deviation units. The vertical line indicates the threshold for identifying X outliers, i.e., the value for the limit of “normal values” to determine the X outliers. The leverage threshold (h*) was calculated using the formula 3*P*/*n*, where *P* represents the number of the model variables plus one (300 + 1 in this case) and *n* is the number of datapoints (1647 in this case). This is the recommended protocol in the OECD guidelines for the leverage approach and has been previously used in similar QSAR modeling studies as well.^25−27^

To further asses the top model’s performance on datapoints within its AD, we performed a 10-fold cross-validation of the model with datapoints within its AD, i.e., we removed the X and Y outliers identified via the leverage approach and performed a 10-fold cross-validation and reported the average *R*^2^, RMSE, and MAE. To evaluate the model’s performance on data outside its AD chemical space, we trained the model on the datapoints within its AD and tested it on the datapoints identified via the leverage approach as outliers (i.e., outside the model’s AD) and reported the average errors.

### 2.2 Structure-Based Virtual Screening and Preliminary Selection of Hits

#### 2.2.1 Preparing the FDA-Approved Drug Dataset

A total of 5883 FDA-approved drugs were curated from the ZINC15 and DrugBank databases.^28,29^ The dataset included the names, SMILES strings, and structure files (MDL MOL; mol2) of the drugs.

#### 2.2.2 Molecular Docking

The 3D mol2 file of each FDA-approved drug was converted to the PDBQT format (after water and ions were removed, polar hydrogens were added, and the Gasteiger charge model was applied) using *RDKit* (v2022.3.3.3) and *Meeko* (v0.3.3.3; https://github.com/forlilab/Meeko) in a Python environment.^30^ MDM2’s 3D crystal structure was obtained from the MDM2/Nutlin-3a complex determined at 1.15 Å resolution by X-ray radiation (PDB ID:5C5A) from the Research Collaboratory for Structural Bioinformatics Protein Data Bank.^31^ The complexed inhibitor Nutlin-3a (NT), along with other ions/solvents, was stripped from the MDM2 structure, and the file was converted into the PDBQT format by adding polar hydrogen, merging non-polar hydrogens, and applying the Gasteiger charge model using *MGLTools* (v1.5.6).^32^ The molecular docking experiment was performed using *AutoDock Vina* (v1.1.2) by selecting a search space of 25 × 25 × 20 Å with reference to the position of the NT in the MDM2–NT complex structure (site-specific docking), and the exhaustiveness parameter of *AutoDock Vina* was set to 256.^33^ All molecular docking calculations were carried out using Trott and Olson’s (2009) protocol, and the complete configuration file for *AutoDock Vina* docking used in the study is provided in Supporting Information Data 2 (S2). Each FDA-approved drug in the PDBQT format was docked to the MDM2 structure in the specified search space using *AutoDock Vina*. The highest affinity score (the most negative in terms of kcal/mol unit) for each FDA-approved drug was recorded. NT was also docked under the same setup as the experimental control for comparison as a reference.

#### 2.2.3 Preliminary Selection of Promising Hits

A scoring matrix was devised to facilitate the identification of the drug candidates that were the most promising for further screening and validation in the present study. The scoring matrix was constructed to assign each FDA-approved drug a numerical score ranging from 0 to 1, with the highest score corresponding to the most favorable candidates. Detailed information regarding the methodologies employed to construct the scoring matrix is provided in Supporting Information Data 3 (S3). To explain it briefly, the developed scoring matrix was used to assign a numerical score (between 0 and 1) for each of the 5883 FDA-approved drugs, and subsequent investigations were primarily based on each drug’s scores with some consideration for availability and accessibility.

The selected hits were re-docked to MDM2 using the same configuration, except that the search space was set to 70 × 70 × 70 Å (blind docking) to confirm the docking site specificity and reproducibility of the results. Blind docking at this stage was performed more specifically to identify any sites other than the active site of MDM2 to which the FDA-approved drugs could bind with a higher affinity than that with the MDM2’s active site. This was performed to filter any FDA-approved drugs with higher “off-site” affinity as these compounds would (theoretically) preferentially bind to the former (i.e., the off-site) in vitro (and provide undesirable results in further downstream analyses).

The interaction profiles between the top hits and MDM2 in their docked pose were analyzed and visualized using Schrödinger *PyMOL* and Schrödinger *Maestro’s* “Ligand Interaction Diagram” modules.^34,35^

### 2.3 Molecular Dynamics Analysis

#### 2.3.1 Building the Simulation System

To validate the in silico interaction of the selected candidate drugs (top hits) within the active site of MDM2 under cellular conditions, molecular dynamics simulation was conducted for each MDM2–drug complex. The molecular dynamics system was generated using *VMD* (v1.9.3) and *CHARMM-GUI* tools and was aimed to mimic the physiological conditions within the cell’s microenvironment.^36,37^ The system was set up in a simulation environment with periodic boundary conditions encompassing a rectangular box with a 10 × 10 × 10 Å buffer region from the edge of the protein to the edge of the rectangle, a temperature of 310.15 K (i.e., physiological temperature), and a concentration of 0.15 mol/L of Na^+^, K^+^, and Cl^–^ ions (i.e., cellular ion concentration), and the system was saturated with water using the TIP3P water model. The parameter and topology files for both MDM2 and the candidate drugs were generated using the CHARMM36m force field, and molecular dynamics simulations were conducted using *Nanoscale Molecular Dynamics* software (v2.14 CUDA).^38,39^

#### 2.3.2 Molecular Dynamics Simulation

Molecular dynamics simulations were performed in two steps. First, the simulation system was subjected to energy minimization for 50,000 steps using the conjugate gradient descent algorithm, followed by an equilibration run in which the candidate drugs and the backbone of MDM2 were constrained under NVT (constant number of atoms, *N*; constant volume, *V*; constant temperature, and *T*; temperature controlled via the Langevin thermostat) conditions for 10 ns. During this short simulation, the side chains of MDM2 and the water and ions in the system were allowed to move freely. Subsequently, all the constraints were removed from the system, and MDM2 and the candidate drugs were allowed to equilibrate freely under *NPT* (constant number of atoms, *N*; constant pressure, *P*; and constant temperature, *T*) conditions for 100 ns (production run). The Langevin thermostat was used for temperature control, and a Nose–Hoover Langevin piston was used for pressure control. The configuration files for running both the steps of the dynamics simulation are provided in Supporting Information Data 4 (S4).

#### 2.3.3 Analysis of the Trajectory

Root-mean-square deviation (RMSD) analysis was used to quantify the motion of the ligands (i.e., the candidate drugs and the control NT) docked within the p53-binding site of MDM2 during the production run of the molecular dynamics simulation. Root-mean-square fluctuation (RMSF) analysis was used to explain the average RMSD contribution per residue in the simulation. The first frame of the production simulation was used as the reference for the RMSD and RMSF analyses. The cut-off distance for the donor–acceptor pair for hydrogen bonding analysis was set to 4.0 Å, and for hydrophobic interaction analysis, it was calculated as the number of hydrophobic amino acids (G, A, V, L, I, P, F, M, and W) within 4.0 Å of the ligand. All analyses were performed using *MDAnalysis* (v2.2.0).^40^ The results were plotted and visualized using *Matplolib* and *Seaborn* libraries in a Python environment.^41,42^

### 2.4 Cell Line Maintenance and Drug Preparation

#### 2.4.1 Selecting the Cell Lines

The primary focus of this study was to identify the MDM2 inhibitors that can bind to the p53-binding site of MDM2 by quantifying the end-point outcomes indirectly (cell death due to p53 activation and/or the upregulation of the p53-regulated genes). To attain such a goal, the selected cell lines have to express functional MDM2 and p53, without hotspots or damaging mutations, in other words, the cell lines with a functional MDM2–p53 axis. Additionally, we aimed to validate the selected hits specifically for glioblastoma and neuroblastoma; hence, to identify the suitable cell lines for these cancers, we utilized the Cell Line Selector tool from the Cancer Dependency Map portal and screened for “neuroblastoma” and “glioblastoma” with null hotspots or damaging mutations in the *TP53* and *MDM2* genes.^43^ From this screening, we selected the U87MG (also known as U87) and SH-SY5Y cell lines as models for glioblastoma and neuroblastoma, respectively, mainly due to their accessibility. A complete list of the candidate glioblastoma and neuroblastoma cell lines that fit the inclusion criteria is available in Supporting Information Data 5 (S5) for further evaluation.

#### 2.4.2 Cell Line Maintenance

Cell line maintenance was performed by thawing the U87 (passage:13) and SH-SY5Y (passage:9) cells and culturing them in sterile T75 flasks containing DMEM (high glucose Dulbecco’s modified Eagle’s medium with stable l-glutamine, sodium pyruvate, and sodium bicarbonate; Sigma-Aldrich, United States) supplemented with 10% FBS [(Fetal Bovine Serum), Sigma-Aldrich, United States] and 1% PenStrep (10^4^ units/mL penicillin and 10^4^ μg/mL Streptomycin; Gibco, United States). The cells were then incubated at 37 °C in a humidified 5% CO_2_ incubator, and the medium was changed every two days until the cells reached 90% confluency, at which point they were harvested using trypsinization for subsequent analyses. The first passage after immediate thawing was discarded for each cell line, and all the assays were performed from the second passage. This process remained constant for all the cell lines and cell-derived assays in this study.

#### 2.4.3 Drug Preparation

To prepare Cetirizine dihydrochloride (CZ; Santa Farma, Turkey) and Rupatadine (RP; Abdi Ibrahim, Turkey), 10 mg/0.16 mL of each drug was incubated in dimethyl sulfoxide [(DMSO); Merck, United States] for 60 min at 30 °C in an ultrasonic bath (62.5 mg/mL). The drugs were then diluted 80-fold with DMEM to a working concentration of 0.78 mg/mL and sterilized by passing through a 0.2 μm filter to remove any non-dissolved matter. DMSO was used as the vehicle control in all experiments, and its concentration was also diluted like that of the drugs (80-fold with DMEM).

### 2.5 Cell Proliferation Assay

#### 2.5.1 Experimental Setup

Confluent U87 and SH-SY5Y cells were seeded in 96-well plates at a density of 10^4^ cells/well/200 μL and incubated at 37 °C in a humidified 5% CO_2_ incubator for 24 h. The medium was removed, and the cells were washed with 200 μL of PBS (Sigma-Aldrich, United States). Each cell line was treated with either CZ or RP (1 drug per plate) in sextuplicate, and the remaining six wells were treated with the vehicle control at an equivalent concentration. The cells were then incubated for 24 h at 37 °C in a humidified incubator with 5% CO_2_. All experiments were carried out using a 1/2 serial dilution (starting from 780 μg/mL) and repeated at least twice.

#### 2.5.2 MTT Assay

After the 24 h incubation period, the plates were rinsed with PBS, and 100 μL of DMEM containing 0.5 mg/mL 3-(4,5-dimethylthiazol-2-yl)-2,5-diphenyltetrazolium bromide [(MTT); Sigma-Aldrich, United States] was added to each well. The plates were then incubated at 37 °C in a 5% CO_2_ incubator for 3 h. After 3 h, the supernatant was removed, and 100 μL of pure DMSO was added to each well. The plates were then incubated in a shaking incubator at 120 rpm for 30 min. The absorbance of the microplates was measured at wavelengths of 590 and 690 nm. The IC_50_ values were calculated, and the dose–response graph was generated using *GraphPad Prism* (v8.4.3), and the calculations are further explained in Supporting Information Data 6 (S6).

### 2.6 Quantitative Real-Time PCR Assay for Gene Expression Analysis

#### 2.6.1 Experimental Setup

Confluent U87 and SH-SY5Y cells were seeded in 6-well plates at a density of 3 × 10^5^ cells/well/3 mL and incubated at 37 °C in a humidified 5% CO_2_ incubator for 24 h. The medium was removed, and the cells were washed with 3 mL of PBS. Each cell line was treated with either CZ or RP at half of their IC_50_ concentration (as calculated in the previous step); the experiments were performed in triplicate with the remaining three wells of the 6-well plates being treated with the vehicle control at an equivalent concentration. The cells were incubated for 24 h at 37 °C in a humidified 5% CO_2_ incubator, after which they were harvested.

#### 2.6.2 Identifying Target Genes and Primer Selection

To analyze the activation of the p53 pathway in the CZ- and RP-treated cell lines, quantitative reverse transcriptase polymerase chain reaction (qRT-PCR) analysis was performed to analyze the changes in gene expression. Following the work of Fischer (2017), three genes transcriptionally regulated by p53, which are responsible for different cellular functions were selected for analysis. Among the selected genes were *BAX* (Bcl-2-associated X protein; an apoptosis regulator), *CDKN1A* (cyclin-dependent kinase inhibitor 1A; inducer of cell cycle arrest), and *DDB2* (damage-specific DNA binding protein 2; DNA damage sensor). Additionally, the protein–protein interaction network functional enrichment analysis using the STRING database highlighted that the interactions between *TP53* and *BAX*, *CDKN1A*, and *DDB2* have a local clustering coefficient of 0.991, 0.999, and 0.967, respectively (i.e., they are involved in the same pathway).^44^ The Gene Ontology and Reactome pathway’s enrichment support the transcription regulation of the genes by p53; more details are provided in Supporting Information Data 7 (S7).

The primers against the mRNA transcripts of the *BAX*, *CDKN1A*, and *DDB2* genes were designed using the NCBI Primer-BLAST tool.^45^*ACTB* (β-actin) and *GAPDH* (glyceraldehyde 3-phosphate dehydrogenase) were used as the housekeeping gene (endogenous) controls. The sequence for the primers is provided in Supporting Information Data 8 (S8).

#### 2.6.3 Total RNA Isolation and cDNA Synthesis

Total RNA was isolated from the harvested cell pellets using the EcoPURE Total RNA kit (EcoTech Biotechnology, Turkey), following the manufacturer’s protocol (Cat No: E2075). Immediately afterward, cDNA synthesis was performed using the SensiFast cDNA Synthesis kit (Bioline, United Kingdom) following the manufacturer’s protocol (Cat No: BIO-65053). To ensure the success of the RNA isolation, the RNA samples were quantified before the cDNA synthesis; more details are provided in Supporting Information Data 9 (S9).

#### 2.6.4 qRT-PCR Assay Run and Analysis

qRT-PCR was performed using a LightCycler 96 instrument (Roche, Switzerland). The assay was performed using A.B.T. 2X qPCR SYBR-Green MasterMix without ROX kit (Cat No: Q03-01-05) (Atlas Biyoteknoloji, Turkey). The reaction mixture consisted of 10 μL of A.B.T. 2X qPCR SYBR-Green Master Mix, 1 μL of the forward primer, 1 μL of the reverse primer, 5 μL of the cDNA template, and 3 μL of RNase-free water, and the final tube volume was 20 μL. The kit manufacturer’s recommended protocol was followed for the instrument’s settings; briefly, the program consisted of a pre-incubation step at 40 °C for 30 s, followed by an initial denaturation step at 95 °C for 10 s. Amplification was performed in three steps: denaturation at 95 °C for 10 s, annealing at 66 °C for 20 s, and acquisition at 72 °C for 25 s, and the process was repeated for 25 cycles.

The cycle threshold (Ct) values were obtained using the instrument software (*LightCycler 96 Instrument Software* v1.1.1). The expression levels of the exposed target genes after exposure to CZ or RP relative to the vehicle control in each cell line were calculated using the 2^–ΔΔCt^ method, after normalizing to the average of the *ACTB* and *GAPDH* genes (endogenous control).^46^*GraphPad Prism* (v8.4.3) was used to generate plots, and an unpaired *t*-test was used for statistical analysis. All the experiments were performed in triplicate, and the results are expressed as mean ± standard deviation.

## 3 Results

### 3.1 Performance of Different Machine Learning Models in Predicting MDM2 Inhibitors

A curated set of 1647 MDM2 inhibitors from the ChEMBL30 database was employed in this study to develop ten distinct regression-based machine learning models using the *mol2vec* featurizers. Table 1 presents the performance of each model following a 10-fold cross-validation. The K-neighbors model demonstrated the highest R^2^ value of 0.74 and the lowest RMSE value of 0.71, outperforming all the other generated models. The best hyperparameters for the model following GridSearchCV are provided in Table 2, and the serialized model can be accessed at https://github.com/naeemmrz/MDM2pred. An online version of the K-neighbors model is available as a web application named *MDM2pred*; *MDM2pred* accepts the SMILES (or a list of SMILES) strings as the input and outputs the predicted pIC_50_/IC_50_ values. The developed application can be accessed at http://ynlab.mu.edu.tr/tr/mdm2pred-6997. More details about the model usage are available in Supporting Information Data 10 (S10).

**Table 1:** Performance Comparison of Regression-Based Machine Learning Models for MDM2 Inhibitor Prediction Using *mol2vec* Featurizers^a^

algorithm	*R*^2^	RMSE	MAE
K-neighbors	0.74	0.70	0.53
multilayer perceptron	0.71	0.74	0.53
random forest	0.72	0.74	0.54
extra trees	0.71	0.74	0.51
light gradient boosting	0.71	0.74	0.53
stochastic gradient descent	0.71	0.75	0.57
histogram-based gradient boosting	0.71	0.74	0.53
extreme gradient boosting	0.67	0.79	0.56
adaptive boosting	0.65	0.83	0.69
decision tree	0.52	0.96	0.67

a All the values are the average of the 10-fold cross-validation on the test set.

**Table 2:** Performance of the K-Neighbors Model in Each Iteration of the 10-Fold Cross Validation after Applying the Hyperparameters^a^

iteration	test *R*^2^	test RMSE	test MAE
**1**	0.79	0.66	0.49
**2**	0.76	0.71	0.53
**3**	0.65	0.79	0.54
**4**	0.83	0.57	0.43
**5**	0.76	0.68	0.50
**6**	0.66	0.74	0.56
**7**	0.77	0.67	0.48
**8**	0.79	0.66	0.48
**9**	0.63	0.85	0.63
**10**	0.83	0.61	0.43
**Mean**	**0.75**	**0.69**	**0.51**
**standard deviation**	**0.07**	**0.08**	**0.06**

a The hyperparameters of the model were as follows: “algorithm”, “auto”; “leaf_size”, 30; “metric”, “minkowski”; “metric_params”, None; “n_neighbors”, 5; and “p”, 2; “weights”, “uniform”.

All the values are the average of the 10-fold cross-validation on the test set. The K-neighbors model’s performance for each iteration of the 10-fold cross validation is provided in Table 2. The lower limit values for the *R*^2^, RMSE, and MAE were 0.63, 0.57, and 0.43, respectively, and their upper limits were 0.83, 0.85, and 0.63, respectively. The standard deviation over 10 iterations for the *R*^2^, RMSE, and MAE were 0.07, 0.08, and 0.06, respectively.

**Figure 2:**
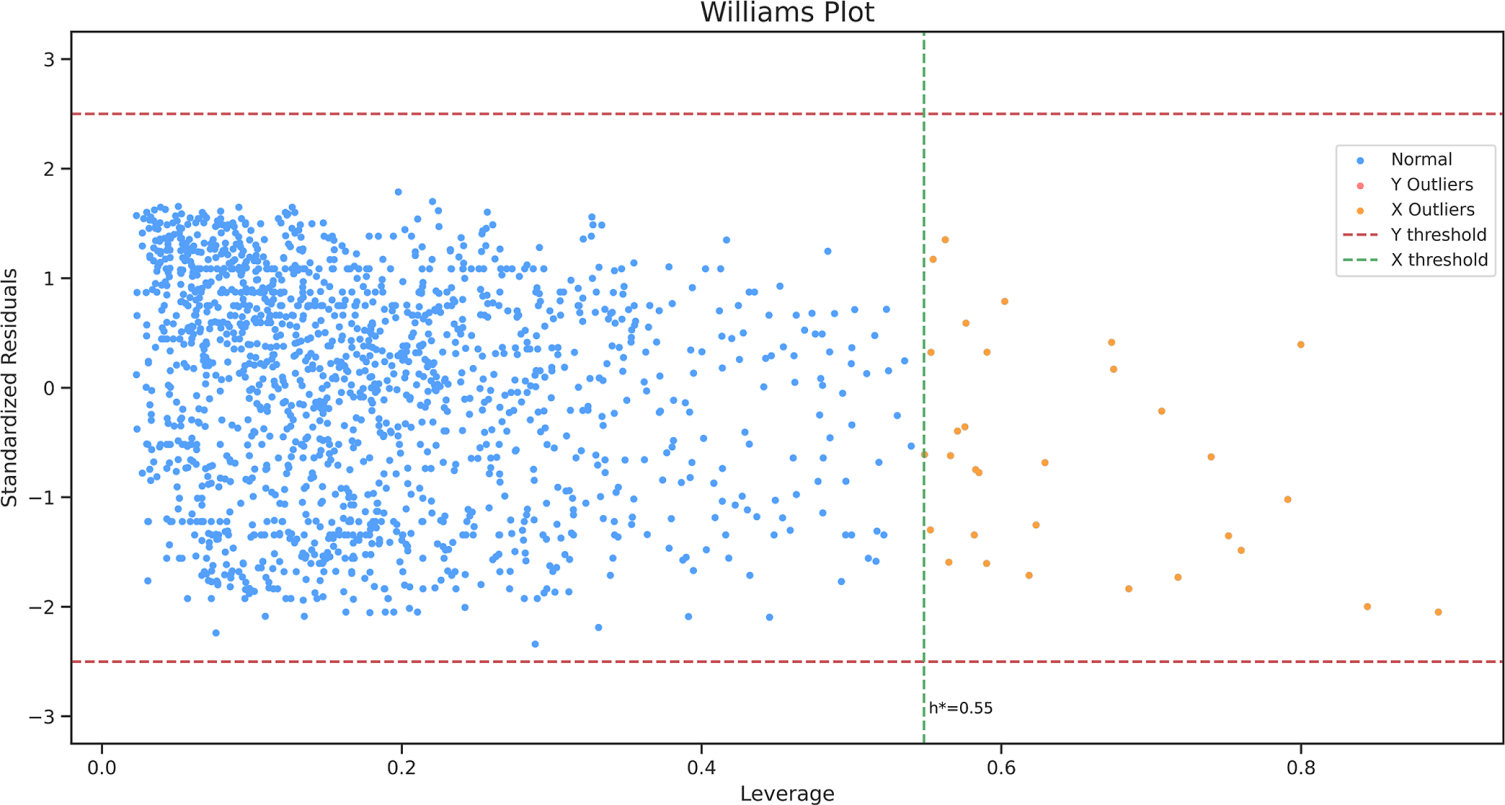
Williams plot for describing the AD of the K-neighbors model. The areas enclosed by the red and green dotted lines represent the model’s AD, and the datapoints outside the enclosed space are outliers. No Y outliers were identified.

By following the leverage approach with a ±2.5 standard deviation unit cut-off (Figure 2, red dotted lines) for standardized residuals and a leverage threshold (h*) of 0.55 (Figure 2, green dotted line), the applicability of the K-neighbors model was determined. Based on these thresholds, 98.12% of the datapoints (1616) used to train the model fell within the model’s AD (Figure 2, blue dots), and 1.88% of the datapoints (31) were identified as the X outliers (Figure 2, yellow dots), and there were no Y outliers detected. The K-neighbors model’s 10-fold cross-validated *R*^2^, RMSE, and MAE on datapoints within its AD were 0.76 ± 0.03, 0.68 ± 0.06, and 0.49 ± 0.03, respectively. The RMSE and MAE of the former model on the 31 datapoints outside its AD were 1.29 and 1.01, respectively.

### 3.2 Molecular Docking and Dynamics of MDM2 with CZ and RP

**Figure 3:**
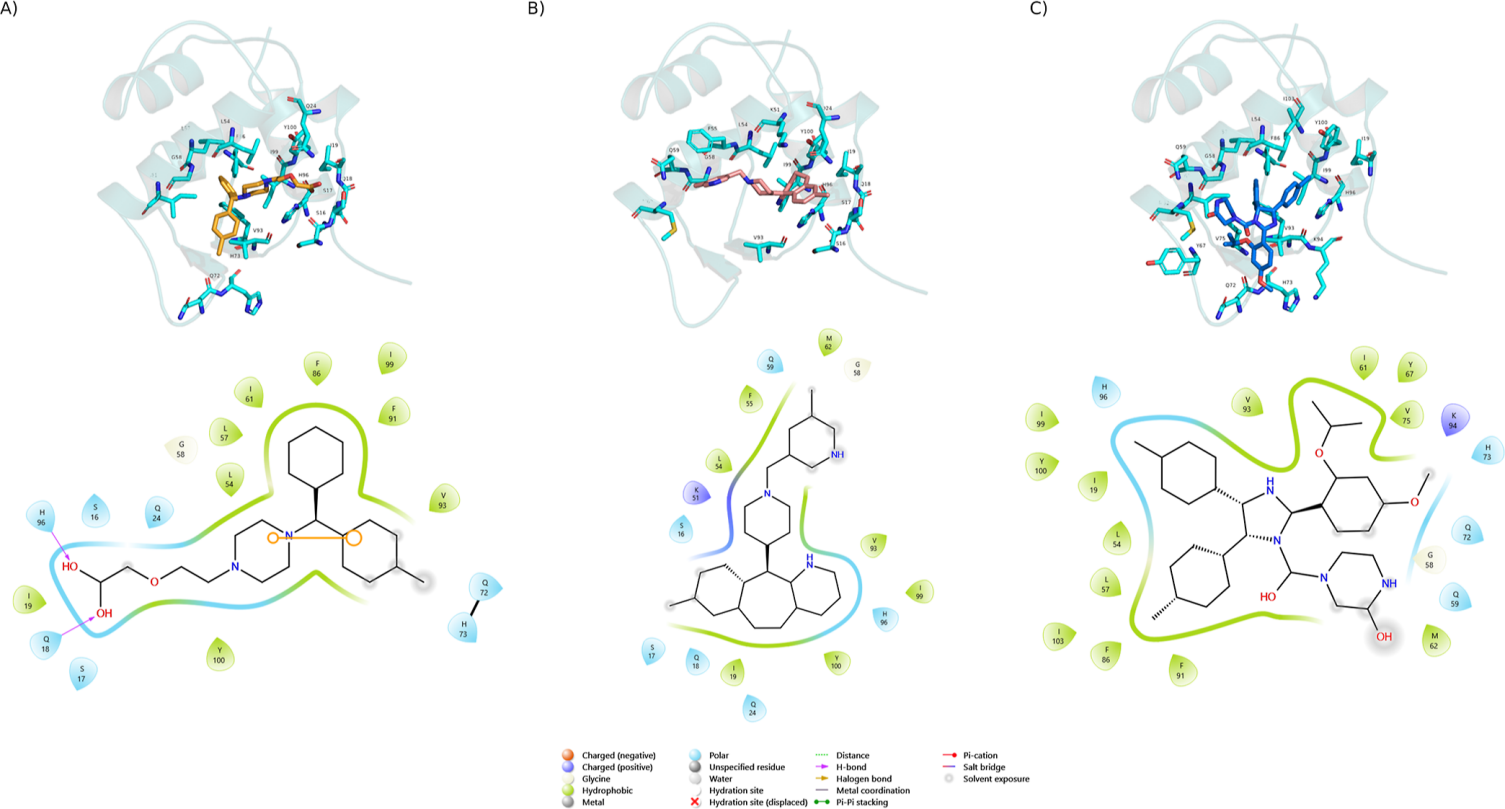
Interaction diagram for MDM2–CZ (orange, A), MDM2–RP (pink, B), and MDM2–NT (blue, C). The top panel illustrates a 3D view of the ligand structure within MDM2’s p53-binding site, and all the residues within 4 Å are shown in sticks. The bottom panel illustrates the interaction environment and the residue position in 2D.

The *MDM2pred* model predicted the IC_50_ values for CZ and RP to be 34.995 and 33.963 nM, with *AutoDock Vina* affinity values of −7.6 and −8.8 kcal/mol, respectively. CZ and RP obtained scores of 0.70 and 0.78, respectively, according to the scoring matrix explained in Supporting Information S3. By comparison, NT, the control used, was predicted to have an IC_50_ value of 33.42 nM and an *AutoDock Vina* affinity of −8.3 kcal/mol, resulting in a score of 0.75. The interactions between MDM2 and CZ, RP, and NT are illustrated in Figure 3.

**Figure 4:**
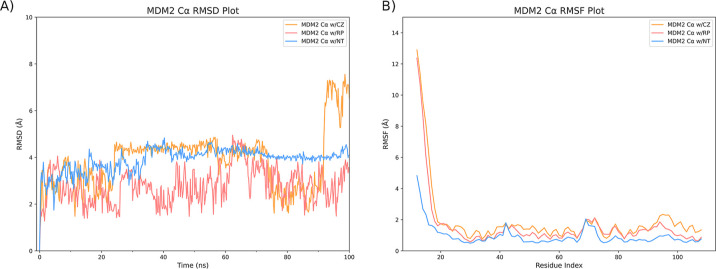
Analysis of MDM2 dynamics during the molecular dynamics simulation. (A) Cα RMSD and (B) Cα RMSD of MDM2 in the presence of CZ, RP, and NT, with respect to the first frame of the simulation.

**Figure 5:**
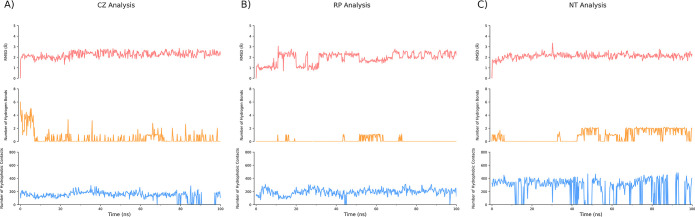
Analysis of the ligand dynamics and interactions during the molecular dynamics simulation. The top (pink), middle (orange), and bottom (blue) subplots illustrate the ligand RMSD, number of hydrogen bonds, and number of hydrophobic contacts between MDM2 and CZ (A), RP (B), and NT (C), respectively. The cut-off distance for the hydrogen bond and hydrophobic contacts was set to 4.0 Å.

The production run of the molecular dynamics simulations of MDM2 with CZ, RP, and NT was performed for 100 ns, and the resulting Cα RMSD for each molecule was analyzed. The Cα RMSD plot for MDM2 in each simulation is presented in Figure 4A and the RMSD of the ligands’ backbone in each simulation is presented in Figure 5. Both plots show the respective molecules aligned to the first frame of the trajectory. The number of feasible hydrogen bonds between MDM2 and CZ, RP, and NT atoms over simulation time within a 4.0 Å range and a donor–acceptor cut-off angle of 120° and the number of feasible hydrophobic contacts over simulation time within a 4.0 Å range of MDM2’s hydrophobic residues are depicted in Figure 5.

### 3.3 Antiproliferative Effect of CZ and RP on U87 and SH-SY5Y Cell Lines

**Figure 6:**
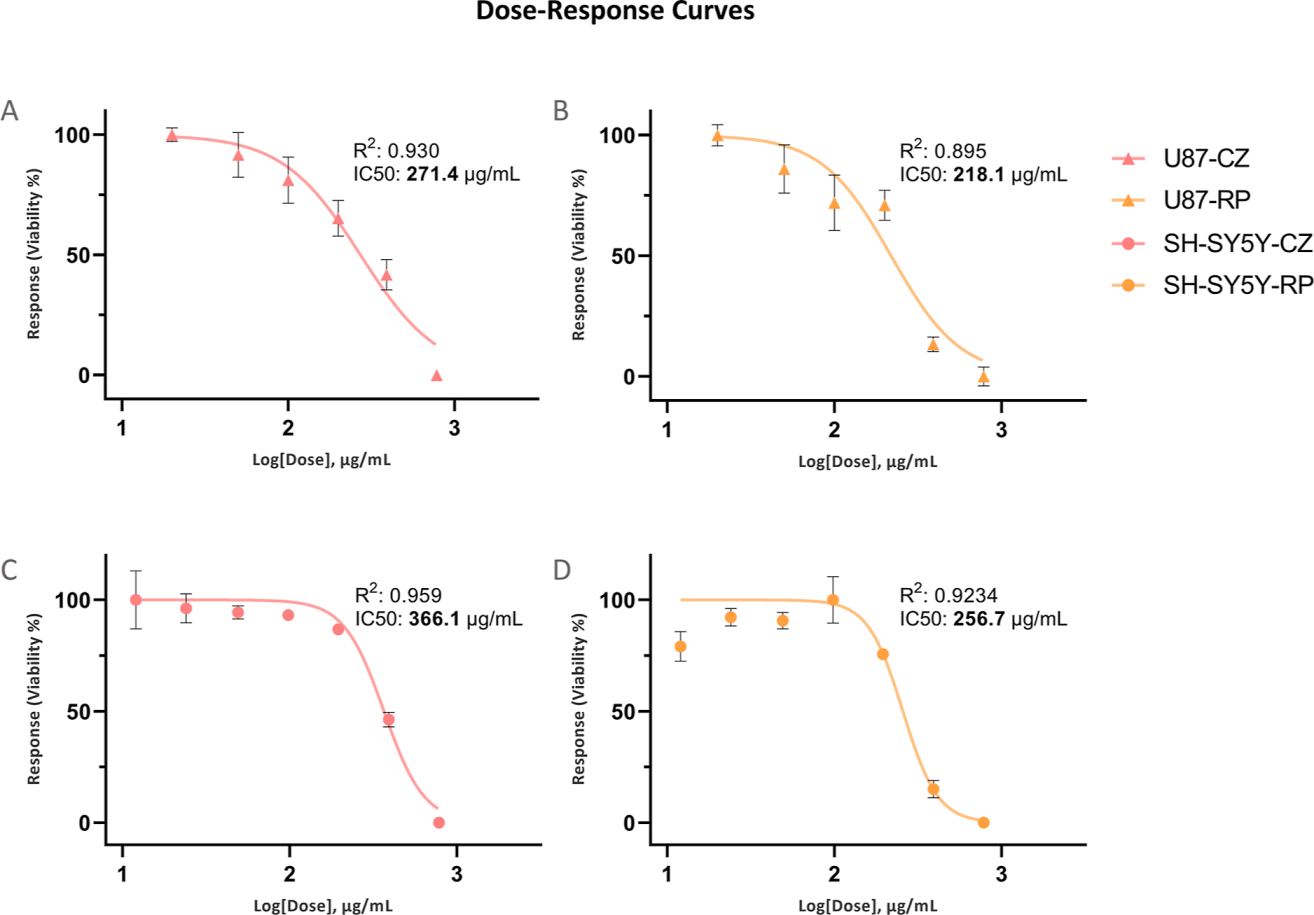
Dose–response curves of the U87 and SH-SY5Y cell lines treated with CZ and RP for 24 h (A) U87-CZ, (B) SH-SY5Y-CZ, (C) U87-RP, and (D) SH-SY5Y-RP. The IC_50_ and *R*^2^ values of CZ and RP on the U87 and SH-SY5Y cells were calculated using the module. The IC_50_ of CZ on the U87 and SH-SY5Y cell lines was calculated as 271.4 and 366.1 μg/mL, respectively. The IC_50_ values of RP on the U87 and SH-SY5Y cell lines were calculated as 218.1 and 256.7 μg/mL, respectively. The lines represent the fitted nonlinear curve. The plot was generated using *GraphPad Prism*’s log(inhibitor) vs normalized response—variable slope nonlinear regression curve fit module. The Error bars indicate standard deviation, and the results represent data from two independent experiments conducted in sextuples.

Figure 6 illustrates the dose–response plot for CZ- and RP-treated U87 and SH-SY5Y cell lines. A nonlinear regression curve fit with variable response was used to analyze the cell viability against drug concentration and calculate the IC_50_ values. Figure 6A,B shows the response in the U87 and SH-SY5Y cells to CZ, and Figure 6C,D depicts the response in the U87 and SH-SY5Y cells to RP. The calculated IC_50_ values of the CZ treatment were 271.4 (95% CI: 240.7–305.3) and 366.1 (95% CI: 339.8–390.9) μg/mL for U87 and SH-SY5Y cell lines, respectively. The calculated IC_50_ values of the RP treatment were 218.1 (95% CI: 185.1–254.1) and 256.7 (95% CI: 233.3–282.7) μg/mL for U87 and SH-SY5Y cell lines, respectively.

### 3.4 Effects of the CZ and RP Treatments on the Expression of p53-Regulated Genes

**Figure 7:**
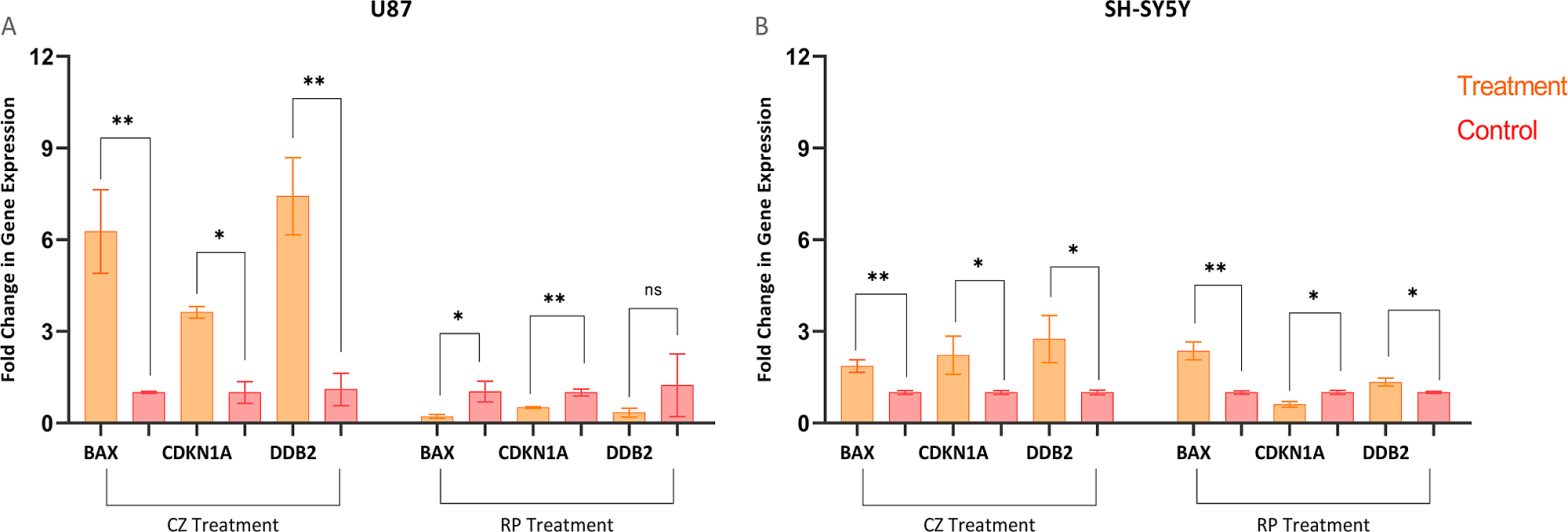
Relative mRNA expression levels of *BAX*, *CDKN1A*, and *DDB2* genes in (A) U87 and (B) SH-SY5Y cells treated with CZ or RP. The fold changes in gene expression levels were calculated relative to the vehicle control for each cell line by following the 2^–ΔΔCt^ method, *ACTB* and *GAPDH* were used as the endogenous controls for each cell line and treatment group. The error bars indicate standard deviation, and the results represent data from two independent experiments conducted in triplicates. Significance symbols * *P*-value < 0.05, ** *P*-value < 0.01, and ns not significant.

Figure 7 shows the fold changes in the gene expression levels between drug-treated and vehicle controls for the U87 and SH-SY5Y cell lines. In the U87 cells, the CZ treatment resulted in 6.26-, 3.63-, and 7.43-fold changes in the expression levels of *BAX*, *CDKN1A*, and *DDB2*, respectively. In the SH-SY5Y cells, the CZ treatment led to 1.87-, 2.22-, and 2.75-fold changes in the expression levels of *BAX*, *CDKN1A*, and *DDB2*, respectively. The RP treatment in the U87 cell line resulted in 0.22-, 0.51-, and 0.34-fold changes in expression levels of *BAX*, *CDKN1A*, and *DDB2*, respectively. The RP treatment in the SH-SY5Y cell line led to 2.36-, 0.61-, and 1.34-fold changes in the expression levels of *BAX*, *CDKN1A*, and *DDB2*, respectively.

## 4 Discussion

Cancer is a multifaceted malady characterized by the unrestrained growth and proliferation of cells, which have the propensity to invade nearby tissues and disseminate to distant organs. The etiology of cancer is multifactorial, involving genetic mutations, environmental factors, and lifestyle choices. Despite the advancements in cancer therapeutics, there remains an ongoing need to identify novel therapeutic targets and develop innovative drugs to treat cancer. One such potential target for cancer therapy is the MDM2 protein, which plays a pivotal role in regulating the activity of the tumor suppressor protein p53. The inhibition of the MDM2–p53 interaction has emerged as a promising strategy for developing new cancer treatments. In this context, drug repurposing, which entails repurposing existing drugs for new indications, has the potential to be a compelling approach to identifying MDM2 inhibitors. Drug repurposing not only presents practical advantages but also offers safety and economic benefits. In this study, we aimed to explore the potential of drug repurposing for identifying the MDM2 inhibitors using a multidisciplinary data-driven approach. To achieve this, a set of 1647 MDM2 inhibitors were curated from the ChEMBL30 database to develop ten distinct machine learning models utilizing the *mol2vec* featurizer. These models were compared based on their performances in predicting the MDM2 inhibitors. The K-neighbors model performed the best, with a cross-validated *R*^2^ value of 0.74 (0.63–0.80), RMSE of 0.71 (0.61–0.85), and MAE of (0.48–0.62) as shown in Table 1. The K-neighbors algorithm, while fundamentally straightforward neighbor-based (compared to the more sophisticated gradient-boosting or tree-based algorithms), has demonstrated remarkable performance in our study. We postulated that the exceptional efficacy of the K-neighbors model could be partially attributed to the inherent characteristics of our dataset. To investigate this further, we conducted k-mean clustering and principal component analysis, shedding light on the dataset’s influence on the obtained results. Comprehensive details of this subsequent analysis are available in Supporting Information Data 11 (S11). Moreover, apart from its efficacy, the K-neighbors model confers an added advantage in terms of computational speed; this feature proves particularly advantageous in large-scale screening scenarios, where timely processing of vast datasets is paramount.^47^ The *R*^2^ metric holds a prominent position as one of the most widely used evaluation measures for regression-based QSAR models. It provides insight into the proportion of variance accounted for by the model; however, it is equally crucial to take into consideration the RMSE and MAE values of the QSAR models. In this regard, the top model detailed in Table 2 exhibits RMSE and MAE values of 0.69 ± 0.08 and 0.51 ± 0.06, respectively. While these values are moderately high in magnitude, a similar trend is observed across the other models listed in Table 1 as well. It is important to contextualize these results within the training data, which encompasses compounds with pIC_50_ values spanning from 4.99 to 9.93 (provided in Supporting Information S1). When viewed in this light, the MAE of 0.51 ± 0.06 units represents only a small fraction of the overall pIC_50_ range. Nonetheless, it is equally important to acknowledge the logarithmic scale of the response variable in this case. As a consequence, even seemingly minor errors in the predicted pIC_50_ values can lead to more substantial errors when converting back to the IC_50_ values. Consequently, meticulous attention to the predictive accuracy of the QSAR model remains a crucial aspect of this analysis.

The Williams plot visually depicts the AD of the top-performing K-neighbors model, wherein the region enclosing the blue dots demarcates the AD of this model (Figure 2). The leverage threshold, determined by applying the formula 3*P*/*n*, was calculated to be 0.55, encompassing approximately 98.12% of the training data. Notably, the K-neighbors model exhibited comparable performance when trained and cross-validated solely on datapoints within its AD, with no inclusion of the X or Y outliers (Table 2). This finding suggests that the model’s predictive capability remained relatively unaffected by the presence of these outliers; however, it is also plausible that the low overall count of outliers in the dataset contributed to this outcome. To address this, instead of the recommended 3*P*/*n* formula to calculate the leverage threshold, we used 2*P*/*n*; this resulted in more datapoints being considered as the outliers, but the cross-validated results of the model still remained similar (Supporting Information Data 12). Conversely, upon assessing the K-neighbors model’s performance on the datapoints falling outside its AD, a notable discrepancy was observed in the RMSE and MAE values, recorded at 1.29 and 1.01, respectively, when trained with data solely within its AD. These values were nearly twice as high as the cross-validated RMSE and MAE (0.68 ± 0.06 and 0.49 ± 0.03, respectively) when the model was evaluated on datapoints within its AD only. This evidence supports the conclusion that the K-neighbors model yields reliable predictions with a reasonable margin of error for datapoints within its AD, but its performance substantially declines when dealing with the datapoints outside this domain. An online version of the K-neighbors model, named *MDM2pred*, is available as a web application, the application accepts SMILES strings as input and gives as output the predicted pIC_50_/IC_50_ values.

In this study, *MDM2pred* was used to screen a dataset of 5883 FDA-approved drugs curated from the ZINC15 and DrugBank databases for their potential efficacy in inhibiting MDM2. To further increase the accuracy of the screening process, we performed molecular docking of each of the 5883 FDA-approved drugs against the p53-binding site of MDM2; hence, in addition to considering the pIC_50_ values, we also utilized the molecular docking results in the selection hits. To this extent, we utilized a scoring matrix (explained in detail in Supporting Information S3) that factored in both the predicted pIC_50_ values and the affinity scores from *AutoDock Vina* to preselect hit compounds. As a result, CZ and RP, two antihistamine drugs which scored 0.70 and 0.78, respectively, were preselected for further analysis.^48,49^ For comparison, NT, a known inhibitor of MDM2 in vivo, scored 0.75 on the same matrix. The predicted IC_50_ values for CZ and RP were 34.995 and 33.963 nM, respectively, while their *AutoDock Vina* affinity scores were −7.6 and −8.8 kcal/mol, respectively.

It has been previously shown that MDM2 has a narrow, continuous, and hydrophobic pocket (residues 25–110) that is essential for the interaction with p53 and that competitive inhibition of this interaction can effectively hinder the MDM2–p53 interaction. The most important of these residues are the F19, W23, and L26 located in the N-terminal domain of p53.^50−52^ In this context, NT has been shown to mimic the hydrophobic interactions of p53 and to interact with several critical residues in MDM2 that are involved in the p53–MDM2 disruption mechanism.^53−55^ In our in silico study, NT was used as a control and reproduced these hydrophobic interactions with L54, L57, I61, V75, H96, I99, and Y100, residues of MDM2 (Figure 3C). These results suggest that CZ and RP have an affinity toward MDM2 as both CZ and RP exhibited hydrophobic interactions with the key residues of MDM2 that are necessary for the latter’s interaction with p53 (Figure 3A,B). CZ was found to interact with several of these hydrophobic residues as well (Figure 3A, green ovals) and form π–π stacking (orange stick connecting 2 circles) interactions and hydrogen bonding with Q18 and H96 (Figure 3A, purple arrow). Similarly, RP was found to interact with several hydrophobic residues as well (Figure 3B, green ovals) and appears to have an equally balanced hydrophobic and polar residues surrounding it (Figure 3B). Although NT does not form any hydrogen bond with MDM2, other potent MDM2 inhibitors like SAR405838 (MI-77301) are known to form hydrogen bonds along with hydrophobic interactions and π–π stacking interactions to competitively bind to the p53-binding site of MDM2.^52,55^

Figure 4A displays the Cα RMSD of MDM2 during simulations with CZ, RP, and NT. In all the three simulation systems, the protein RMSD remained relatively stable, except for the CZ simulation, where a sudden increase was observed at approximately 90 ns. This anomalous behavior can be attributed to the high RMSF observed in the N-terminal loop region of MDM2 (residues ∼1–20). Notably, the RMSD discrepancy is visually evident from the trajectory as well (as a randomly floating loop, Supporting Information Data 13). Interestingly, the NT simulation exhibited no such phenomenon, suggesting enhanced stability of MDM2 Cα upon NT binding. This observation aligns with the interaction diagram in Figure 3, where NT (C) is enclosed by hydrophobic interactions on all sides, a feature not shared by CZ and RP (A and B). Analysis of the molecular dynamics simulations for CZ and RP in complex with MDM2 also revealed that both ligands maintained a consistently low RMSD throughout the 100 ns production simulation, akin to the behavior of NT (Figure 5A, top panel, depicted in pink). Hydrogen bonding analysis showed comparable results for CZ and NT, averaging approximately two hydrogen bonds. This outcome can be rationalized by referring to the interaction diagram in Figure 3 (panels A and C), wherein both ligands present two hydroxy groups available for hydrogen bonding, a feature absent in RP, thus accounting for the lack of detectable hydrogen bonds (Figures 5B and 3B). Although the number of hydrophobic contacts between MDM2 and CZ, RP, and NT was quite similar, NT demonstrated a greater number of hydrophobic contacts. This observation further explains the lower Cα RMSF value for NT compared to those for CZ and RP, indicative of more extensive hydrophobic interactions and a more stable MDM2–NT complex. These interactions are crucial for binding to the p53-binding domain of MDM2, as previously reported.^56^ Drawing from these interpretations, we conducted further investigations involving CZ and RP in vitro on the U87 and SH-SY5Y cell lines, employing MTT assay and qRT-PCR.

The MTT assay results showed that both CZ and RP exhibited a dose-dependent inhibition of U87 and SH-SY5Y proliferation after 24 h of exposure (Figure 6). The calculated IC_50_ values of the CZ treatment were 271.4 (95% CI: 240.7–305.3) and 366.1 (95% CI: 339.8–390.9) μg/mL for the U87 (Figure 6A) and SH-SY5Y (Figure 6B) cell lines, respectively. Similarly, the calculated IC_50_ values of RP treatment were 218.1 (95% CI: 185.1–254.1) and 256.7 (95% CI: 233.3–282.7) μg/mL for the U87 (Figure 6C) and SH-SY5Y (Figure 6D) cell lines. While the antiproliferative effect of NT on the U87 and SH-SY5Y cell lines was not investigated in vitro in our study, the Genomics of Drug Sensitivity in Cancer (GDSC) database has previously reported the IC_50_ value of NT on SK-N-SH, a neuroblastoma cell line that shares similar characteristics to SH-SY5Y, to be 4.83 ± 0.81 μM (2808.70 μg/mL). The IC_50_ value of NT on the U87 cell line was reported as 28.31 ± 0.92 μM (16451.17 μg/mL) by GDSC. The data presented in GDSC are based on the results obtained via Syto60, Resazurin, or CellTiter-Glo methods after 72 h of incubation.^57^ These results strongly indicate that both CZ and RP are potent inhibitors of the U87 and SH-SY5Y cell lines’ proliferation in vitro; this can be observed as the 24 h exposure IC_50_ dose of both compounds in each cell line was multiple folds lower than the 72 h exposure IC_50_ dose reported for the SK-N-SH and U87 cell lines by GDSC.

The genes *BAX*, *CDKN1A*, and *DDB2* are integral for the regulation of cell growth and survival. *BAX* functions as a pro-apoptotic gene and helps initiate programmed cell death when prompted by various signals^58^*CDKN1A* encodes p21, which can halt the cell cycle and avert the replication of the damaged DNA.^59^*DDB2* is a vital constituent of the DNA damage recognition complex, which recognizes and repairs the DNA damage.^60^ The commonality among *BAX*, *CDKN1A*, and *DDB2* is their regulation by the p53 tumor suppressor protein, which plays a crucial role in cancer prevention by regulating cell growth and inducing apoptosis via the expression of these genes.^61^ Consequently, these genes were utilized as an indirect marker to assess qualitatively and quantitatively the activation of p53, i.e., the inhibition of MDM2 upon treatment with CZ or RP. As shown in Figure 7, CZ treatment significantly increased the expression of *BAX*, *CDKN1A*, and *DDB2* in the U87 and SH-SY5Y (Figure 7) cell lines. The expression of *BAX*, *CDKN1A*, and *DDB2* in the U87 cells was upregulated 6.26 (*p* < 0.01), 3.63 (*p* < 0.05), and 7.43 (*p* < 0.01) folds, respectively (Figure 7A), while their expression in the SH-SY5Y cells exhibited a 1.87 (*p* < 0.01)-, 2.22 (*p* < 0.05)-, and 2.75 (*p* < 0.05)-fold increase, respectively (Figure 7B) upon CZ treatment. This data demonstrates that CZ treatment upregulates the expression of the three p53-regulated genes and suggests p53 activation or MDM2 inhibition as supported by the interaction profiles observed in the molecular docking and molecular dynamics analysis of CZ with MDM2 (Figures 3A and 4). In contrast, treatment of the U87 cells with RP resulted in a reverse effect on the expression of *BAX* (*p* < 0.05), *CDKN1A* (*p* < 0.001), and *DDB2* (*p* > 0.05) (Figure 7A). A similar trend was observed in the expression of *CDKN1A* (*p* < 0.05) in the SH-SY5Y cells, while the expression of *BAX* and *DDB2* increased by 2.36 (*p* < 0.001) and 1.34 (*p* < 0.05) folds (Figure 7B). The disparate findings between the RP-treated cells could suggest the possibility of a compensatory mechanism that may contribute to maintaining p53 in its inactive form despite RP treatment. It is also plausible that RP exhibits MDM2–p53 axis-independent cytotoxic effects on cells as it was observed to inhibit the proliferation of both cell lines(Figure 6). Although the results of the RP-treated cells were not further examined in this study, a recent in vivo study has demonstrated that RP exhibits important anti-inflammatory and anti-apoptotic effects against l-arginine-induced acute pancreatitis by reducing the expression of nuclear factor kappa-B (NF-κB) and caspase 3, with the latter playing a crucial role in the apoptosis cascade that is anticipated upon p53 activation.^62,63^ A confluence of the anti-apoptotic effects due to caspase 3 downregulation and the pro-apoptotic effects due to MDM2 inhibition could contribute to the near-basal/down-regulated levels of *BAX*, *CDKN1A*, and *DDB2* expression in the RP-treated U87 cell lines. Despite the relatively low IC_50_ dose, the same mechanism might not be as effective in the SH-SY5Y cell lines, hence the increased *BAX* and *DDB2* expression but the decreased level of *CDKN1A* expression. Nonetheless, the current data do not provide conclusive evidence for the possible MDM2–p53 axis-dependent mechanisms of RP-induced cytotoxicity or cell death in vitro*.* Further studies are needed to elucidate the underlying molecular mechanisms involved in the RP-mediated inhibition of the U87 and SH-SY5Y cell lines’ proliferation.

Drug repurposing presents several benefits over *de novo* drug discovery, such as reduced costs, shorter development time, and improved safety profiles. This approach involves identifying novel therapeutic uses for existing drugs, thus offering a shortcut to drug discovery. Our study used this strategy to screen for potent MDM2 inhibitors by integrating data-driven techniques with computational and cellular methods. Our findings revealed that two anti-histamine drugs, CZ and RP, exhibited a robust affinity toward the p53-binding site of MDM2 in silico and anti-proliferative activity on both U87 glioblastoma and SHSY5Y neuroblastoma cell lines in vitro. Analysis of the p53-regulated genes in the CZ-treated but not RP-treated cells demonstrated statistically significant upregulation in all 3 genes, *BAX*, *CDKN1A*, and *DDB2*. This alteration in the expression profile overlaps with the profile of p53 activation. The gene expression level analysis of the same genes in the RP-treated SH-SY5Y cells revealed upregulation only in *BAX* and *DDB2* but not in *CDKN1A*, whereas the RP-treated U87 cells exhibited the downregulation of all 3 genes. Therefore, CZ, but not RP, was found to be an ideal p53 activator in vitro by upregulating the expression of the p53-regulated genes responsible for cell cycle arrest and apoptosis. CZ’s pharmacokinetic and toxicity profiles are also well-known and have been FDA-approved since 1995, facilitating the transition from preclinical studies to clinical trials and accelerating its potential use in the clinical practice for cancer treatment. Further investigation of CZ in preclinical and clinical studies is warranted to establish its potential as a cancer treatment, particularly for the tumors with wild-type p53.

## 5 Conclusions

In conclusion, our study highlights the potential of drug repurposing as a viable approach for identifying MDM2 inhibitors. The use of machine learning models and molecular docking techniques has enabled us to identify the potential MDM2 inhibitors from a pool of FDA-approved drugs. The in silico screening of this drug pool has led to the identification of CZ and RP as promising candidates for MDM2 inhibition. Subsequent in vitro experiments using glioblastoma and neuroblastoma cell lines have revealed that both CZ and RP effectively inhibited their proliferation, and qRT-PCR has shown that CZ upregulated the expression of the p53-regulated genes involved in cell cycle arrest, apoptosis, and DNA damage response. These findings show that CZ can be an effective p53 activator in vitro, and given its known safety profile and approval status, it could easily be translated into a clinical treatment method if the results are reproducible in vivo. Further studies are warranted to validate these findings and investigate the repurposing of CZ for the treatment of wild-type p53 tumors.

## Supporting Information Available

Spreadsheet of the datapoints used to build the QSAR model, Code snippet used to run the docking experiment, details on the scoring matrix, NAMD configuration files to run the molecular dynamic simulation, spreadsheet of the neuroblastoma and glioblastoma cell lines with wild-type p53-MDM2, IC50 value calculations from MTT results, Reactome and GO enrichment analysis results for the selected p53-regulated genes, primer sequences used in the qRT-PCR, concentration of the RNA samples used for the qRT-PCR, MDM2pred web applications user guide, K-mean clustering and principle component analysis, and AD (PDF)
Molecular dynamics simulation (MP4)
