# Supplementary material for: Targeting MDM2–p53 Axis through Drug Repurposing for Cancer Therapy: A Multidisciplinary Approach
Source: ACS Omega. 2023 Sep 15;8(38):34583–96. doi: 10.1021/acsomega.3c03471 (PMC10536845; doi:10.1021/acsomega.3c03471)
Supplement: Supplementary file 1 — ao3c03471_si_001.pdf [file ao3c03471_si_001.pdf]

TARGETING MDM2-p53 AXIS THROUGH DRUG REPURPOSING FOR CANCER THERAPY: A MULTIDISCIPLINARY APPROACH

Naeem ABDUL GHAFOOR[1]

Ayşegül YILDIZ[1,2*] aysegulyildiz@mu.edu.tr

[1] Department of Molecular Biology and Genetics, Graduate School of Natural and Applied Sciences, Mugla Sitki Kocman University, 48000 Mugla, Turkey

[2] Department of Molecular Biology and Genetics, Faculty of Science, Mugla Sitki Kocman University, 48000 Mugla, Turkey

[*] Corresponding author

S01: SPREADSHEET OF THE DATA POINTS USED TO BUILD THE QSAR MODEL

Table S1. MDM2 inhibitors dataset.

document_journal	document_year	molecule_chembl_id	canonical_smiles	IC50_nM	pIC50
Bioorg. Med. Chem. Lett.	2005	CHEMBL178578	O=C(O)[C@H](c1ccccc1)N1C(=O)c2cc(I)ccc2NC(=O)[C@@H]1c1ccc(C(F)(F)F)cc1	1700.0	5.77
J. Med. Chem.	2006	CHEMBL379505	CC(C)(C)C[C@H]1N[C@@H](C(=O)NCCN2CCOCC2)[C@H](c2cccc(Cl)c2)[C@@]12C(=O)Nc1cc(Cl)ccc12	800.0	6.10
Bioorg. Med. Chem. Lett.	2006	CHEMBL378662	C[C@H](c1ccc(Cl)cc1N)N1C(=O)c2cc(I)ccc2N(C(=O)CN2CCNCC2)C(=O)[C@@H]1c1ccc(Cl)cc1	1550.0	5.81
Bioorg. Med. Chem. Lett.	2006	CHEMBL207541	C[C@H](c1ccc(Cl)cc1)N1C(=O)c2cc(I)ccc2N(C(=O)CN2CCOCC2)C(=O)[C@@H]1c1ccc(Cl)cc1	5400.0	5.27
Bioorg. Med. Chem. Lett.	2006	CHEMBL207202	COCCOCCN1C(=O)[C@H](c2ccc(Cl)cc2)N([C@H](C)c2ccc(Cl)cc2)C(=O)c2cc(I)ccc21	2390.0	5.62
Bioorg. Med. Chem. Lett.	2006	CHEMBL378851	C[C@H](c1ccc(Cl)cc1)N1C(=O)c2cc(I)ccc2N(CCN2CCOCC2)C(=O)[C@H]1c1ccc(Cl)cc1	6250.0	5.20
Bioorg. Med.	2006	CHEMBL209201	C[C@H](c1ccc(Cl)cc1)N1C(=O)c2cc(I)ccc2N(CCN2CCOCC2)C(=O)[C@@H]1c1ccc(Cl)cc1	2700.0	5.57

Chem. Lett.					
Bioorg. Med. Chem. Lett.	2006	CHEMBL411220	O=C(O)CCN1C(=O)C(c2ccc(Cl)cc2)N(C(C(=O)O)c2ccc(Cl)cc2)C(=O)c2cc(I)ccc21	1090.0	5.96
Bioorg. Med. Chem. Lett.	2006	CHEMBL209156	O=C(O)CN1C(=O)C(c2ccc(Cl)cc2)N(C(C(=O)O)c2ccc(Cl)cc2)C(=O)c2cc(I)ccc21	10200.0	4.99
Bioorg. Med. Chem. Lett.	2006	CHEMBL208020	O=C(O)C(c1ccc(Cl)cc1)N1C(=O)c2cc(I)ccc2N(Cc2ccncc2)C(=O)C1c1ccc(Cl)cc1	4350.0	5.36
Bioorg. Med. Chem. Lett.	2006	CHEMBL210443	CNC(=O)CN1C(=O)C(c2ccc(Cl)cc2)N(C(C(=O)O)c2ccc(Cl)cc2)C(=O)c2cc(I)ccc21	3330.0	5.48
Bioorg. Med. Chem. Lett.	2006	CHEMBL443053	CN1C(=O)C(c2ccc(Cl)cc2)N(C(C(=O)O)c2ccc(Cl)cc2)C(=O)c2cc(I)ccc21	2180.0	5.66
Bioorg. Med. Chem. Lett.	2006	CHEMBL207255	O=C(O)CCNC(=O)[C@H](c1ccc(Cl)cc1)N1C(=O)c2cc(I)ccc2NC(=O)[C@@H]1c1ccc(Cl)cc1	850.0	6.07
Bioorg. Med. Chem. Lett.	2006	CHEMBL382422	O=C(NCCO)[C@H](c1ccc(Cl)cc1)N1C(=O)c2cc(I)ccc2NC(=O)[C@@H]1c1ccc(Cl)cc1	2120.0	5.67
Bioorg. Med. Chem. Lett.	2006	CHEMBL207364	CC#Cc1ccc2c(c1)C(=O)N([C@H](C)c1ccc(Cl)cc1N)[C@@H](c1ccc(Cl)cc1)C(=O)N2CCN1CCOCC1	10000.0	5.00

Bioorg. Med. Chem. Lett.	2006	CHEMBL379487	CN1CCN(CCCCN2C(=O)[C@H](c3ccc(Cl)cc3)N(Cc3ccc(Cl)cc3N)C(=O)c3cc(I)ccc32)CC1	5660.0	5.25
Bioorg. Med. Chem. Lett.	2006	CHEMBL380399	CN1CCN(CCCN2C(=O)[C@H](c3ccc(Cl)cc3)N(Cc3ccc(Cl)cc3N)C(=O)c3cc(I)ccc32)CC1	5410.0	5.27
Bioorg. Med. Chem. Lett.	2006	CHEMBL378998	CN(C)CCCCN1C(=O)[C@H](c2ccc(Cl)cc2)N(Cc2ccc(Cl)cc2N)C(=O)c2cc(I)ccc21	2830.0	5.55
Bioorg. Med. Chem. Lett.	2006	CHEMBL378030	O=C1[C@H](c2ccc(Cl)cc2)N(Cc2ccc(Cl)cc2F)C(=O)c2cc(I)ccc2N1CCN1CCOCC1	8600.0	5.07
Bioorg. Med. Chem. Lett.	2006	CHEMBL208999	C[C@H](c1cc(O)c(Cl)cc1N)N1C(=O)c2cc(I)ccc2N(CCN2CCOCC2)C(=O)[C@@H]1c1ccc(Cl)cc1	2700.0	5.57
Bioorg. Med. Chem. Lett.	2006	CHEMBL210853	C[C@H](c1cc(F)c(Cl)cc1N)N1C(=O)c2cc(I)ccc2N(CCN2CCOCC2)C(=O)[C@@H]1c1ccc(Cl)cc1	2600.0	5.59
Bioorg. Med. Chem. Lett.	2006	CHEMBL380178	Nc1cc(Cl)ccc1CN1C(=O)c2cc(I)ccc2N(CCCN2CCOCC2)C(=O)[C@@H]1c1ccc(Cl)cc1	3600.0	5.44
Bioorg. Med. Chem. Lett.	2006	CHEMBL424876	COCCOCCN1C(=O)[C@H](c2ccc(Cl)cc2)N(Cc2ccc(Cl)cc2O)C(=O)c2cc(I)ccc21	2440.0	5.61
Bioorg. Med.	2006	CHEMBL210545	COCCOCCN1C(=O)[C@H](c2ccc(Cl)cc2)N([C@H](C)c2ccc(Cl)cc2N)C(=O)c2cc(I)ccc21	700.0	6.15

Chem. Lett.					
Bioorg. Med. Chem. Lett.	2006	CHEMBL208554	Nc1cc(Cl)ccc1CN1C(=O)c2cc(I)ccc2N(CCCCC(=O)O)C(=O)[C@@H]1c1ccc(Cl)cc1	810.0	6.09
Bioorg. Med. Chem. Lett.	2006	CHEMBL207332	CC(c1ccc(Cl)cc1N)N1C(=O)c2cc(I)ccc2N(CCCCC(=O)O)C(=O)[C@@H]1c1ccc(Cl)cc1	250.0	6.60
Bioorg. Med. Chem. Lett.	2006	CHEMBL379293	CC(c1ccc(Cl)cc1)N1C(=O)c2cc(I)ccc2N(CCCCC(=O)O)C(=O)[C@@H]1c1ccc(Cl)cc1	850.0	6.07
Bioorg. Med. Chem. Lett.	2006	CHEMBL377203	C[C@H](c1ccc(Cl)cc1N)N1C(=O)c2cc(I)ccc2N(CCN2CCOCC2)C(=O)[C@@H]1c1ccc(Cl)cc1	790.0	6.10
Nature	2007	CHEMBL361103	O=C(O)[C@H](c1ccc(Cl)cc1)N1C(=O)c2cc(I)ccc2NC(=O)[C@@H]1c1ccc(Cl)cc1	420.0	6.38
Bioorg. Med. Chem. Lett.	2008	CHEMBL515487	COc1ccc(C2=NC(c3ccc(Cl)cc3)C(c3ccc(Cl)cc3)N2)c(OC(C)C)c1	90.0	7.05
J. Med. Chem.	2009	CHEMBL577393	CN1CCN(CCNC(=O)[C@@H]2N[C@H](CC(C)(C)C)[C@]3(C(=O)Nc4cc(Cl)ccc43)[C@H]2c2cccc(Cl)c2F)CC1	25.7	7.59
J. Med. Chem.	2009	CHEMBL379173	CC(C)(C)C[C@H]1N[C@@H](C(=O)NCCN2CCOCC2)[C@H](c2cccc(Cl)c2F)[C@@]12C(=O)Nc1cc(Cl)ccc12	28.3	7.55
J. Med. Chem.	2009	CHEMBL575121	CN1CCC(CCNC(=O)[C@@H]2N[C@H](CC(C)(C)C)[C@]3(C(=O)Nc4cc(Cl)ccc43)[C@H]2c2cccc(Cl)c2F)CC1	32.4	7.49

J. Med. Chem.	2009	CHEMBL575538	COCCOCCNC(=O)[C@@H]1N[C@H](CC(C)(C)C)[C@]2(C(=O)Nc3cc(Cl)ccc32)[C@H]1c1cccc(Cl)c1F	32.4	7.49
J. Med. Chem.	2011	CHEMBL1688151	Nc1ccc(CCN2C(=O)c3ccccc3C2(O)c2ccc(Cl)cc2)cc1	6200.0	5.21
J. Med. Chem.	2011	CHEMBL1688150	O=C1c2ccccc2C(O)(c2ccc(Cl)cc2)N1CCc1ccc([N+](=O)[O-])cc1	6200.0	5.21
J. Med. Chem.	2011	CHEMBL1688149	O=C1c2ccccc2C(O)(c2ccc(Cl)cc2)N1CCc1ccccc1	7200.0	5.14
J. Med. Chem.	2011	CHEMBL1688147	O=C1c2ccccc2C(O)(c2ccc(Br)cc2)N1Cc1ccc([N+](=O)[O-])cc1	3100.0	5.51
J. Med. Chem.	2011	CHEMBL1688146	O=C1c2ccccc2C(O)(c2ccc(Cl)cc2)N1Cc1ccc([N+](=O)[O-])cc1	2400.0	5.62
J. Med. Chem.	2011	CHEMBL1688143	N#Cc1ccc(CN2C(=O)c3ccccc3C2(O)c2ccc(Cl)cc2)cc1	6000.0	5.22
J. Med. Chem.	2011	CHEMBL1688140	O=C1c2ccccc2C(O)(c2ccc(Cl)cc2)N1Cc1ccc(Br)cc1	4500.0	5.35
J. Med. Chem.	2011	CHEMBL1688139	O=C1c2ccccc2C(O)(c2ccc(Cl)cc2)N1Cc1ccc(Cl)cc1	4200.0	5.38
J. Med. Chem.	2011	CHEMBL1688170	O=C1c2ccccc2C(OC2CCC(O)C2)(c2ccc(Cl)cc2)N1Cc1ccccc1	3000.0	5.52
J. Med.	2011	CHEMBL1688251	O=C1c2ccccc2C(OC2CCC(O)C2)(c2ccc(Cl)cc2)N1Cc1ccc([N+](=O)[O-])cc1	700.0	6.15

Chem.					
J. Med. Chem.	2011	CHEMBL1688252	O=C1c2ccccc2C(O[C@H]2CC[C@@H](O)C2)(c2ccc(Cl)cc2)N1Cc1ccc([N+](=O)[O-])cc1	400.0	6.40
J. Med. Chem.	2011	CHEMBL1688253	N#Cc1ccc(CN2C(=O)c3ccccc3C2(O[C@H]2CC[C@@H](O)C2)c2ccc(Cl)cc2)cc1	2300.0	5.64
J. Med. Chem.	2011	CHEMBL1688259	O=C1c2ccccc2C(OCCCO)(c2ccc(Cl)cc2)N1Cc1ccc([N+](=O)[O-])cc1	450.0	6.35
J. Med. Chem.	2011	CHEMBL1688260	O=C1c2ccccc2C(OCCCCO)(c2ccc(Cl)cc2)N1Cc1ccc([N+](=O)[O-])cc1	360.0	6.44
J. Med. Chem.	2011	CHEMBL1688270	O=C1c2ccccc2C(O[C@H]2CCC[C@H](O)CCC2)(c2ccc(Cl)cc2)N1Cc1ccc([N+](=O)[O-])cc1	380.0	6.42
J. Med. Chem.	2011	CHEMBL1688275	O=C1c2ccccc2C(O[C@H]2CC[C@H](O)CC2)(c2ccc(Cl)cc2)N1Cc1ccc([N+](=O)[O-])cc1	390.0	6.41
J. Med. Chem.	2011	CHEMBL1688278	O=C1c2ccccc2C(OCC2(CO)CC2)(c2ccc(Cl)cc2)N1Cc1ccc(F)cc1	2600.0	5.59
J. Med. Chem.	2011	CHEMBL1688284	C[C@H](c1ccc(Cl)cc1)N1C(=O)c2ccccc2C1(OCC1(CO)CC1)c1ccc(Cl)cc1	8800.0	5.06
J. Med. Chem.	2011	CHEMBL1688303	O=C1c2ccccc2[C@](OCC2(CO)CC2)(c2ccc(Cl)cc2)N1Cc1ccc([N+](=O)[O-])cc1	170.0	6.77
J. Med. Chem.	2011	CHEMBL1688304	O=C1c2ccccc2[C@@](OCC2(CO)CC2)(c2ccc(Cl)cc2)N1Cc1ccc([N+](=O)[O-])cc1	1300.0	5.89

Bioorg. Med. Chem. Lett.	2012	CHEMBL2152318	CC1=C(C(=O)N2CCNC(=O)C2)SC2=N[C@@H](c3ccc(Cl)cc3)[C@@H](c3ccc(Cl)cc3)N21	2700.0	5.57
Bioorg. Med. Chem. Lett.	2012	CHEMBL2152319	CCC1=C(C(=O)N2CCNC(=O)C2)SC2=N[C@@H](c3ccc(Cl)cc3)[C@@H](c3ccc(Cl)cc3)N21	1800.0	5.74
Bioorg. Med. Chem. Lett.	2012	CHEMBL2152320	CC(C)C1=C(C(=O)N2CCNC(=O)C2)SC2=N[C@@H](c3ccc(Cl)cc3)[C@@H](c3ccc(Cl)cc3)N21	260.0	6.59
Bioorg. Med. Chem. Lett.	2012	CHEMBL2152321	CCCC1=C(C(=O)N2CCNC(=O)C2)SC2=N[C@@H](c3ccc(Cl)cc3)[C@@H](c3ccc(Cl)cc3)N21	3100.0	5.51
Bioorg. Med. Chem. Lett.	2012	CHEMBL2152322	O=C1CN(C(=O)C2=C(C3CC3)N3C(=N[C@@H](c4ccc(Cl)cc4)[C@H]3c3ccc(Cl)cc3)S2)CCN1	9500.0	5.02
Bioorg. Med. Chem. Lett.	2012	CHEMBL2152323	CC(C)(C)C1=C(C(=O)N2CCNC(=O)C2)SC2=N[C@@H](c3ccc(Cl)cc3)[C@@H](c3ccc(Cl)cc3)N21	3000.0	5.52
Bioorg. Med. Chem. Lett.	2012	CHEMBL2151052	COCC1=C(C(=O)N2CCNC(=O)C2)SC2=N[C@@H](c3ccc(Cl)cc3)[C@@H](c3ccc(Cl)cc3)N21	7100.0	5.15
Bioorg. Med. Chem. Lett.	2012	CHEMBL2152324	CC(=O)N1CCN(C(=O)C2=C(C(C)C)N3C(=N[C@@H](c4ccc(Cl)cc4)[C@H]3c3ccc(Cl)cc3)S2)CC1	840.0	6.08
Bioorg. Med.	2012	CHEMBL2152325	CC(C)C1=C(C(=O)N2CCC(C(=O)N(C)C)CC2)SC2=N[C@@H](c3ccc(Cl)cc3)[C@@H](c3ccc(Cl)cc3)N21	4400.0	5.36

Chem. Lett.					
Bioorg. Med. Chem. Lett.	2012	CHEMBL2152326	CC(C)C1=C(C(=O)N2CCOCC2)SC2=N[C@@H](c3ccc(Cl)cc3)[C@@H](c3ccc(Cl)cc3)N21	340.0	6.47
Bioorg. Med. Chem. Lett.	2012	CHEMBL2152327	CC(C)C1=C(C(=O)N2CCNC(=O)CC2)SC2=N[C@@H](c3ccc(Cl)cc3)[C@@H](c3ccc(Cl)cc3)N21	1100.0	5.96
Bioorg. Med. Chem. Lett.	2012	CHEMBL2152328	CC(C)C1=C(C(=O)N2CCN(CCO)CC2)SC2=N[C@@H](c3ccc(Cl)cc3)[C@@H](c3ccc(Cl)cc3)N21	1800.0	5.74
Bioorg. Med. Chem. Lett.	2012	CHEMBL2152329	CC(C)C1=C(C(=O)N2CCN(C)CC2)SC2=N[C@@H](c3ccc(Cl)cc3)[C@@H](c3ccc(Cl)cc3)N21	1200.0	5.92
Bioorg. Med. Chem. Lett.	2012	CHEMBL2152330	CC(C)C1=C(C(=O)N2C[C@H](C)N[C@H](C)C2)SC2=N[C@@H](c3ccc(Cl)cc3)[C@@H](c3ccc(Cl)cc3)N21	140.0	6.85
Bioorg. Med. Chem. Lett.	2013	CHEMBL2315846	CC(C)C1=C(C(=O)N2CC[C@H]2C(=O)N(C)C)SC2=N[C@@](C)(c3ccc(Cl)cc3)[C@@H](c3ccc(Cl)cc3)N21	140.0	6.85
J. Med. Chem.	2013	CHEMBL2347375	CC(C)(C)C[C@@H]1N[C@@H](C(=O)NCCN2CCOCC2)[C@H](c2cccc(Cl)c2F)[C@]12C(=O)Nc1cc(Cl)ccc12	9.4	8.03
J. Med. Chem.	2013	CHEMBL2347379	O=C(O)C[C@@H]1C[C@@H](c2cccc(Cl)c2)[C@H](c2ccc(Cl)cc2)N(CC2CC2)C1=O	8100.0	5.09
J. Med.	2013	CHEMBL2347378	O=C(O)C[C@H]1C[C@@H](c2cccc(Cl)c2)[C@H](c2ccc(Cl)cc2)N(CC2CC2)C1=O	2900.0	5.54

Chem.					
J. Med. Chem.	2013	CHEMBL2177203	O=C(O)C[C@@H]1C[C@H](c2cccc(Cl)c2)[C@@H](c2ccc(Cl)cc2)N(CC2CC2)C1=O	76.0	7.12
J. Med. Chem.	2013	CHEMBL2059299	O=C(O)C[C@@H]1C[C@@H](c2cccc(Cl)c2)[C@@H](c2ccc(Cl)cc2)N(CC2CC2)C1=O	2400.0	5.62
J. Med. Chem.	2013	CHEMBL2347377	O=C(O)C[C@@H]1O[C@H](c2cccc(Cl)c2)[C@@H](c2ccc(Cl)cc2)N(CC2CC2)C1=O	140.0	6.85
J. Med. Chem.	2013	CHEMBL2347376	O=C(O)C[C@H]1O[C@H](c2cccc(Cl)c2)[C@@H](c2ccc(Cl)cc2)N(CC2CC2)C1=O	290.0	6.54
J. Med. Chem.	2013	CHEMBL2347393	CC[C@@H](CO)N1C(=O)[C@H](CC(=O)O)O[C@@H](c2cccc(Cl)c2)[C@H]1c1ccc(Cl)cc1	610.0	6.21
J. Med. Chem.	2013	CHEMBL2347392	O=C(O)C[C@H]1O[C@H](c2cccc(Cl)c2)[C@H](c2ccc(Cl)cc2)N(CC2CC2)C1=O	7400.0	5.13
J. Med. Chem.	2013	CHEMBL2347391	O=C(O)CC[C@@H]1O[C@@H](c2cccc(Cl)c2)[C@@H](c2ccc(Cl)cc2)N(CC2CC2)C1=O	1300.0	5.89
J. Med. Chem.	2013	CHEMBL2347390	CNCC[C@@H]1O[C@@H](c2cccc(Cl)c2)[C@@H](c2ccc(Cl)cc2)N(CC2CC2)C1=O	9900.0	5.00
J. Med. Chem.	2013	CHEMBL2347389	O=C(O)C[C@@H]1O[C@@H](c2cccc(Cl)c2)[C@@H](c2ccc(Cl)cc2)N(CC2CC2)C1=O	300.0	6.52
J. Med. Chem.	2013	CHEMBL2347388	O=C1[C@H](CCO)O[C@@H](c2cccc(Cl)c2)[C@@H](c2ccc(Cl)cc2)N1CC1CC1	880.0	6.06

J. Med. Chem.	2013	CHEMBL2347387	C=CC[C@@H]1O[C@@H](c2cccc(Cl)c2)[C@@H](c2ccc(Cl)cc2)N(CC2CC2)C1=O	1400.0	5.85
J. Med. Chem.	2013	CHEMBL2347385	O=C1CO[C@@H](c2cccc(Cl)c2)[C@@H](c2ccc(Cl)cc2)N1CC1CC1	1800.0	5.74
J. Med. Chem.	2013	CHEMBL2347383	CCC[C@H](C(=O)O)N1C(=O)[C@H](Cc2ccc(F)cc2)O[C@@H](c2ccc(Cl)cc2)[C@H]1c1ccc(Cl)cc1	86.0	7.07
J. Med. Chem.	2013	CHEMBL2347382	COc1cccc(C[C@@H]2O[C@@H](c3ccc(Br)cc3)[C@@H](c3ccc(Br)cc3)N(C)C2=O)c1	2100.0	5.68
J. Med. Chem.	2013	CHEMBL2347381	COc1ccc(C[C@@H]2O[C@@H](c3ccc(Br)cc3)[C@@H](c3ccc(Br)cc3)N(C)C2=O)cc1	1900.0	5.72
J. Med. Chem.	2013	CHEMBL2347380	CN1C(=O)[C@H](Cc2ccc(F)cc2)O[C@@H](c2ccc(Br)cc2)[C@H]1c1ccc(Br)cc1	1800.0	5.74
J. Med. Chem.	2013	CHEMBL2347403	CN1C(=O)[C@H](Cc2ccc(Br)cc2)O[C@@H](c2ccc(Br)cc2)[C@H]1c1ccc(Br)cc1	1500.0	5.82
J. Med. Chem.	2013	CHEMBL2347401	CN1C(=O)[C@H](Cc2ccccc2)O[C@@H](c2ccc(Cl)cc2)[C@H]1c1ccc(Cl)cc1	1800.0	5.74
J. Med. Chem.	2013	CHEMBL2347399	CN1C(=O)[C@H](Cc2ccccc2)O[C@@H](c2ccc(Br)cc2)[C@H]1c1ccc(Br)cc1	2000.0	5.70
J. Med. Chem.	2013	CHEMBL2347398	CN1C(=O)CO[C@@H](c2ccc(Br)cc2)[C@H]1c1ccc(Br)cc1	5400.0	5.27
J. Med.	2013	CHEMBL2347394	COc1cccc2c1C1=C([C@H](c3ccc(Br)cc3)n3ncnc3N1C)[C@@H](c1ccc(Br)cc1)O2	70.0	7.15

Chem.					
ACS Med. Chem. Lett.	2013	CHEMBL2386350	CCOc1cc(C(C)(C)C)ccc1C1=N[C@@](C)(c2ccc(Cl)cc2)[C@@](C)(c2ccc(Cl)cc2)N1C(=O)N1CCN(CC(=O)N2CCOCC2)CC1	18.0	7.74
ACS Med. Chem. Lett.	2013	CHEMBL2386348	CCOc1cc(C(C)(C)C)ccc1C1=N[C@@](C)(c2ccc(Cl)cc2)[C@@](C)(c2ccc(Cl)cc2)N1C(=O)N1CCN(CCNC(C)=O)CC1	33.0	7.48
ACS Med. Chem. Lett.	2013	CHEMBL2386347	CCOc1cc(C(C)(C)C)ccc1C1=N[C@@](C)(c2ccc(Cl)cc2)[C@@](C)(c2ccc(Cl)cc2)N1C(=O)N1CCN(CCNS(C)(=O)=O)CC1	46.0	7.34
ACS Med. Chem. Lett.	2013	CHEMBL2386345	CCOc1cc(C(C)(C)C)ccc1C1=N[C@@](C)(c2ccc(Cl)cc2)[C@@](C)(c2ccc(Cl)cc2)N1C(=O)N1CCN(CCO)CC1	26.0	7.59
ACS Med. Chem. Lett.	2013	CHEMBL2386168	CCOc1cc(C(C)(C)C)ccc1C1=N[C@@](C)(c2ccc(Cl)cc2)[C@@](C)(c2ccc(Cl)cc2)N1C(=O)N1CCNC(=O)C1	52.0	7.28
Bioorg. Med. Chem. Lett.	2013	CHEMBL2397155	COc1ccc(COC(=O)N[C@H](C(=O)N[C@@H](CCC(C)=O)C(=O)N[C@@H](Cc2cn(S(=O)(=O)c3c(C)cc(C)cc3C)c3ccccc23)C(=O)N[C@@H](CC(C)C)C(=O)NN)C(C)C)cc1	8510.0	5.07
J. Med. Chem.	2013	CHEMBL2398479	CC(C)(C)C[C@@H]1N[C@@H](C(=O)N[C@H]2C[C@H](NS(C)(=O)=O)C2)[C@H](c2cccc(Cl)c2F)[C@]12C(=O)Nc1cc(Cl)ccc12	7.6	8.12
J. Med. Chem.	2013	CHEMBL2398478	CC(C)(C)C[C@@H]1N[C@@H](C(=O)N[C@H]2C[C@](C)(O)C2)[C@H](c2cccc(Cl)c2F)[C@]12C(=O)Nc1cc(Cl)ccc12	11.1	7.95
J. Med. Chem.	2013	CHEMBL2398477	CC(C)(C)C[C@@H]1N[C@@H](C(=O)NCC(C)(C)O)[C@H](c2cccc(Cl)c2F)[C@]12C(=O)Nc1cc(Cl)ccc12	16.3	7.79
J. Med. Chem.	2013	CHEMBL2398476	CC(C)(C)C[C@@H]1N[C@@H](C(=O)N[C@H]2CC[C@H](O)C2)[C@H](c2cccc(Cl)c2F)[C@]12C(=O)Nc1cc(Cl)ccc12	8.4	8.08

J. Med. Chem.	2013	CHEMBL2398475	CC(C)(C)C[C@@H]1N[C@@H](C(=O)N[C@H]2CC[C@@H](O)C2)[C@H](c2cccc(Cl)c2F)[C@]12C(=O)Nc1cc(Cl)ccc12	8.8	8.06
J. Med. Chem.	2013	CHEMBL2398474	CC(C)(C)C[C@@H]1N[C@@H](C(=O)N[C@H]2C[C@@H](O)C2)[C@H](c2cccc(Cl)c2F)[C@]12C(=O)Nc1cc(Cl)ccc12	8.2	8.09
J. Med. Chem.	2013	CHEMBL2398473	CC(C)(C)C[C@@H]1N[C@@H](C(=O)N[C@H]2C[C@H](O)C2)[C@H](c2cccc(Cl)c2F)[C@]12C(=O)Nc1cc(Cl)ccc12	7.1	8.15
J. Med. Chem.	2013	CHEMBL2402736	CC(C)(C)C[C@@H]1N[C@@H](C(=O)Nc2ccc(C(=O)O)cc2F)[C@H](c2cccc(Cl)c2F)[C@@]1(C#N)c1ccc(Cl)cc1F	25.0	7.60
J. Med. Chem.	2013	CHEMBL2402735	CC(C)(C)C[C@@H]1N[C@@H](C(=O)Nc2ccc(C(=O)O)c(F)c2)[C@H](c2cccc(Cl)c2F)[C@@]1(C#N)c1ccc(Cl)cc1F	21.0	7.68
J. Med. Chem.	2013	CHEMBL2402734	COc1cc(NC(=O)[C@@H]2N[C@@H](CC(C)(C)C)[C@](C#N)(c3ccc(Cl)cc3F)[C@H]2c2cccc(Cl)c2F)ccc1C(=O)O	20.0	7.70
J. Med. Chem.	2013	CHEMBL2402732	CC(C)(C)C[C@@H]1N[C@@H](C(=O)Nc2ccc(C(=O)O)cn2)[C@H](c2cccc(Cl)c2F)[C@@]1(C#N)c1ccc(Cl)cc1F	23.0	7.64
J. Med. Chem.	2013	CHEMBL2402731	CC(C)(C)C[C@@H]1N[C@@H](C(=O)Nc2ccc(C(=O)O)cc2)[C@H](c2cccc(Cl)c2F)[C@@]1(C#N)c1ccc(Cl)cc1F	22.0	7.66
J. Med. Chem.	2013	CHEMBL2402730	CC(C)(C)C[C@@H]1N[C@@H](C(=O)Nc2cccc(C(=O)O)c2)[C@H](c2cccc(Cl)c2F)[C@@]1(C#N)c1ccc(Cl)cc1F	42.0	7.38
J. Med. Chem.	2013	CHEMBL2402729	CC(C)(C)C[C@@H]1N[C@@H](C(=O)Nc2ccn(CC(C)(C)O)n2)[C@H](c2cccc(Cl)c2F)[C@@]1(C#N)c1ccc(Cl)cc1F	56.0	7.25
J. Med.	2013	CHEMBL2402574	CC(C)(C)C[C@@H]1N[C@@H](C(=O)NCC[C@H](O)CO)[C@H](c2cccc(Cl)c2F)[C@@]1(C#N)c1ccc(Cl)cc1F	74.0	7.13

Chem.					
J. Med. Chem.	2013	CHEMBL2402573	CC(C)(C)C[C@@H]1N[C@@H](C(=O)NCC[C@H](O)CO)[C@H](c2cccc(Cl)c2)[C@@]1(C#N)c1ccc(Cl)cc1	196.0	6.71
Bioorg. Med. Chem.	2013	CHEMBL2440443	O=c1nc2n(-c3cccc(Cl)c3)c3c(C(F)(F)F)cccc3cc-2c(=O)[nH]1	1500.0	5.82
ACS Med. Chem. Lett.	2014	CHEMBL3109036	COc1cc(C(N)=O)ccc1NC(=O)[C@@H]1N[C@@H](CC(C)(C)C)[C@@]2(C(=O)Nc3cc(Cl)ccc32)[C@H]1c1cccc(Cl)c1F	5.0	8.30
ACS Med. Chem. Lett.	2014	CHEMBL3109037	COc1cc(C(N)=O)ccc1NC(=O)[C@H]1N[C@H](CC(C)(C)C)[C@]2(C(=O)Nc3cc(Cl)sc32)[C@@H]1c1cccc(Cl)c1F	177.0	6.75
ACS Med. Chem. Lett.	2014	CHEMBL3109038	COc1cc(C(=O)O)ccc1NC(=O)[C@H]1N[C@H](CC(C)(C)C)[C@]2(C(=O)Nc3cc(Cl)sc32)[C@@H]1c1cccc(Cl)c1F	57.0	7.24
ACS Med. Chem. Lett.	2014	CHEMBL3109039	COc1cc(C(N)=O)ccc1NC(=O)[C@H]1N[C@H](CC(C)(C)C)[C@]2(C(=O)Nc3cc(Cl)ncc32)[C@@H]1c1cccc(Cl)c1F	5764.0	5.24
ACS Med. Chem. Lett.	2014	CHEMBL3109040	COc1cc(C(N)=O)ccc1NC(=O)[C@@H]1N[C@@H](CC(C)(C)C)[C@@]2(C(=O)Nc3cc(Cl)sc32)[C@H]1c1cccc(Cl)c1F	7.0	8.15
ACS Med. Chem. Lett.	2014	CHEMBL3109041	COc1cc(C(=O)O)ccc1NC(=O)[C@@H]1N[C@@H](CC(C)(C)C)[C@@]2(C(=O)Nc3cc(Cl)sc32)[C@H]1c1cccc(Cl)c1F	7.0	8.15
ACS Med. Chem. Lett.	2014	CHEMBL3109042	COc1cc(C(N)=O)ccc1NC(=O)[C@@H]1N[C@@H](CC(C)(C)C)[C@@]2(C(=O)Nc3cc(Cl)ncc32)[C@H]1c1cccc(Cl)c1F	6.0	8.22
ACS Med. Chem. Lett.	2014	CHEMBL3109043	COc1cc(C(N)=O)ccc1NC(=O)C1NC(CC(C)(C)C)C2(C(=O)Nc3cc(Cl)sc32)C1c1cccc(Cl)c1F	40.0	7.40

ACS Med. Chem. Lett.	2014	CHEMBL3109044	COc1cc(C(=O)O)ccc1NC(=O)C1NC(CC(C)(C)C)C2(C(=O)Nc3cc(Cl)sc32)C1c1cccc(Cl)c1F	33.0	7.48
ACS Med. Chem. Lett.	2014	CHEMBL3109045	COc1cc(C(N)=O)ccc1NC(=O)[C@@H]1N[C@@H](CC(C)(C)C)[C@@]2(C(=O)Nc3nc(Cl)ncc32)[C@H]1c1cccc(Cl)c1F	45.0	7.35
ACS Med. Chem. Lett.	2014	CHEMBL3109046	COc1cc(C(=O)O)ccc1NC(=O)[C@@H]1N[C@@H](CC(C)(C)C)[C@@]2(C(=O)Nc3nc(Cl)ncc32)[C@H]1c1cccc(Cl)c1F	25.0	7.60
ACS Med. Chem. Lett.	2014	CHEMBL3109047	COc1cc(C(N)=O)ccc1NC(=O)[C@@H]1N[C@@H](CC(C)(C)C)[C@@]2(C(=O)Nc3cc(Cl)cnc32)[C@H]1c1cccc(Cl)c1F	52.0	7.28
ACS Med. Chem. Lett.	2014	CHEMBL3109048	COc1cc(C(=O)O)ccc1NC(=O)[C@@H]1N[C@@H](CC(C)(C)C)[C@@]2(C(=O)Nc3cc(Cl)cnc32)[C@H]1c1cccc(Cl)c1F	46.0	7.34
ACS Med. Chem. Lett.	2014	CHEMBL3109055	COc1cc(C(N)=O)ccc1NC(=O)[C@@H]1N[C@@H](CC(C)(C)C)[C@@]2(C(=O)Nc3nc(Cl)ccc32)[C@H]1c1cccc(Cl)c1F	11.0	7.96
ACS Med. Chem. Lett.	2014	CHEMBL3109049	COc1cc(C(=O)O)ccc1NC(=O)[C@@H]1N[C@@H](CC(C)(C)C)[C@@]2(C(=O)Nc3nc(Cl)ccc32)[C@H]1c1cccc(Cl)c1F	18.0	7.74
ACS Med. Chem. Lett.	2014	CHEMBL3109050	COc1cc(C(=O)O)ccc1NC(=O)[C@@H]1N[C@@H](CC(C)(C)C)[C@@]2(C(=O)Nc3ncccc32)[C@H]1c1cccc(Cl)c1F	52.0	7.28
ACS Med. Chem. Lett.	2014	CHEMBL3109051	COc1cc(C(N)=O)ccc1NC(=O)C1NC(CC(C)(C)C)C2(C(=O)Nc3cc(Cl)ncc32)C1c1cccc(Cl)c1F	17.0	7.77
ACS Med. Chem. Lett.	2014	CHEMBL3109052	COc1cc(C(=O)O)ccc1NC(=O)[C@@H]1N[C@@H](CC(C)(C)C)[C@@]2(C(=O)Nc3cc(Cl)ncc32)[C@H]1c1cccc(Cl)c1F	47.0	7.33
ACS Med.	2014	CHEMBL3109053	COc1cc(C(N)=O)ccc1NC(=O)[C@@H]1N[C@@H](CC(C)(C)C)[C@@]2(C(=O)Nc3ccncc32)[C@H]1c1cccc(Cl)c1F	140.0	6.85

Chem. Lett.					
ACS Med. Chem. Lett.	2014	CHEMBL3109054	COc1cc(C(=O)O)ccc1NC(=O)[C@@H]1N[C@@H](CC(C)(C)C)[C@@]2(C(=O)Nc3ccncc32)[C@H]1c1cccc(Cl)c1F	52.0	7.28
J. Med. Chem.	2014	CHEMBL3125700	CC(C)[C@@H](CS(C)(=O)=O)N1C(=O)[C@@](C)(CC(=O)O)C[C@H](c2cccc(Cl)c2)[C@H]1c1ccc(Cl)cc1	0.3	9.52
J. Med. Chem.	2014	CHEMBL3125699	C[C@@H](CS(C)(=O)=O)N1C(=O)[C@@](C)(CC(=O)O)C[C@H](c2cccc(Cl)c2)[C@H]1c1ccc(Cl)cc1	0.9	9.05
J. Med. Chem.	2014	CHEMBL3125697	CC(C)S(=O)(=O)C[C@H](C1CC1)N1C(=O)[C@@](C)(CC(=O)O)C[C@H](c2cccc(Cl)c2)[C@H]1c1ccc(Cl)cc1	0.2	9.70
J. Med. Chem.	2014	CHEMBL3125540	CC(C)S(=O)(=O)C[C@@H](N1C(=O)[C@@](C)(CC(=O)O)C[C@H](c2cccc(Cl)c2)[C@H]1c1ccc(Cl)cc1)C(C)(C)C	0.4	9.40
J. Med. Chem.	2014	CHEMBL3125539	CC(C)S(=O)(=O)C[C@H](C)N1C(=O)[C@@](C)(CC(=O)O)C[C@H](c2cccc(Cl)c2)[C@H]1c1ccc(Cl)cc1	0.2	9.70
J. Med. Chem.	2014	CHEMBL3125536	CC(C)(C)[C@@H](CS(=O)(=O)C(C)(C)C)N1C(=O)[C@@](C)(CC(=O)O)C[C@H](c2cccc(Cl)c2)[C@H]1c1ccc(Cl)cc1	0.5	9.30
J. Med. Chem.	2014	CHEMBL3125535	CC(C)[C@@H](CS(=O)(=O)C(C)(C)C)N1C(=O)[C@@](C)(CC(=O)O)C[C@H](c2cccc(Cl)c2)[C@H]1c1ccc(Cl)cc1	0.6	9.22
J. Med. Chem.	2014	CHEMBL3125532	CC[C@@H](CS(=O)(=O)c1ccccc1)N1C(=O)[C@@](C)(CC(=O)O)C[C@H](c2cccc(Cl)c2)[C@H]1c1ccc(Cl)cc1	0.3	9.52
J. Med. Chem.	2014	CHEMBL3125531	CC[C@@H](CS(=O)(=O)C1COC1)N1C(=O)[C@@](C)(CC(=O)O)C[C@H](c2cccc(Cl)c2)[C@H]1c1ccc(Cl)cc1	0.2	9.70

J. Med. Chem.	2014	CHEMBL3125530	CC[C@@H](CS(=O)(=O)C1CCCC1)N1C(=O)[C@@](C)(CC(=O)O)C[C@H](c2cccc(Cl)c2)[C@H]1c1ccc(Cl)cc1	0.3	9.52
J. Med. Chem.	2014	CHEMBL3125529	CC[C@@H](CS(=O)(=O)C1CCC1)N1C(=O)[C@@](C)(CC(=O)O)C[C@H](c2cccc(Cl)c2)[C@H]1c1ccc(Cl)cc1	0.2	9.70
J. Med. Chem.	2014	CHEMBL3125528	CC[C@@H](CS(=O)(=O)CC1CC1)N1C(=O)[C@@](C)(CC(=O)O)C[C@H](c2cccc(Cl)c2)[C@H]1c1ccc(Cl)cc1	0.2	9.70
J. Med. Chem.	2014	CHEMBL3125526	CC[C@@H](CS(=O)(=O)C(C)C)N1C(=O)[C@@](C)(CC(=O)O)C[C@H](c2cccc(Cl)c2)[C@H]1c1ccc(Cl)cc1	0.2	9.70
J. Med. Chem.	2014	CHEMBL3125525	CC[C@@H](CS(=O)(=O)C1CC1)N1C(=O)[C@@](C)(CC(=O)O)C[C@H](c2cccc(Cl)c2)[C@H]1c1ccc(Cl)cc1	0.2	9.70
J. Med. Chem.	2014	CHEMBL3125524	CC[C@@H](CS(=O)(=O)CC)N1C(=O)[C@@](C)(CC(=O)O)C[C@H](c2cccc(Cl)c2)[C@H]1c1ccc(Cl)cc1	0.3	9.52
J. Med. Chem.	2014	CHEMBL3125523	CC[C@@H](CCS(C)(=O)=O)N1C(=O)[C@@](C)(CC(=O)O)C[C@H](c2cccc(Cl)c2)[C@H]1c1ccc(Cl)cc1	1.3	8.89
J. Med. Chem.	2014	CHEMBL3125707	CC[C@@H](C[C@H](C)O)N1C(=O)[C@@](C)(CC(=O)O)C[C@H](c2cccc(Cl)c2)[C@H]1c1ccc(Cl)cc1	0.7	9.15
J. Med. Chem.	2014	CHEMBL3125708	CC[C@@H](CC#N)N1C(=O)[C@@](C)(CC(=O)O)C[C@H](c2cccc(Cl)c2)[C@H]1c1ccc(Cl)cc1	1.2	8.92
J. Med. Chem.	2014	CHEMBL3125516	CC[C@@H](CN(C)S(=O)(=O)C1CC1)N1C(=O)[C@@](C)(CC(=O)O)C[C@H](c2cccc(Cl)c2)[C@H]1c1ccc(Cl)cc1	0.9	9.05
J. Med.	2014	CHEMBL3125706	CC[C@@H](C[C@@H](C)O)N1C(=O)[C@@](C)(CC(=O)O)C[C@H](c2cccc(Cl)c2)[C@H]1c1ccc(Cl)cc1	0.4	9.40

Chem.					
J. Med. Chem.	2014	CHEMBL3125704	CC[C@@H](CC(C)(C)O)N1C(=O)[C@@](C)(CC(=O)O)C[C@H](c2cccc(Cl)c2)[C@H]1c1ccc(Cl)cc1	1.0	9.00
J. Med. Chem.	2014	CHEMBL3125705	CC[C@@H](CCC(C)(C)O)N1C(=O)[C@@](C)(CC(=O)O)C[C@H](c2cccc(Cl)c2)[C@H]1c1ccc(Cl)cc1	1.4	8.85
J. Med. Chem.	2014	CHEMBL3125703	CC[C@H](N1C(=O)[C@@](C)(CC(=O)O)C[C@H](c2cccc(Cl)c2)[C@H]1c1ccc(Cl)cc1)C(C)(C)O	1.4	8.85
J. Med. Chem.	2014	CHEMBL2059435	CC[C@@H]([C@H](C)O)N1C(=O)[C@@](C)(CC(=O)O)C[C@H](c2cccc(Cl)c2)[C@H]1c1ccc(Cl)cc1	1.1	8.96
J. Med. Chem.	2014	CHEMBL3125518	CC(C)[C@@H](CN(C)S(=O)(=O)C1CC1)N1C(=O)[C@@](C)(CC(=O)O)C[C@H](c2cccc(Cl)c2)[C@H]1c1ccc(Cl)cc1	0.2	9.70
J. Med. Chem.	2014	CHEMBL3125514	CC[C@@H](CNS(=O)(=O)C1CC1)N1C(=O)[C@@](C)(CC(=O)O)C[C@H](c2cccc(Cl)c2)[C@H]1c1ccc(Cl)cc1	0.5	9.30
J. Med. Chem.	2014	CHEMBL3125515	CC[C@@H](CN(C)S(=O)(=O)C(C)(C)C)N1C(=O)[C@@](C)(CC(=O)O)C[C@H](c2cccc(Cl)c2)[C@H]1c1ccc(Cl)cc1	0.7	9.15
J. Med. Chem.	2014	CHEMBL3125519	CN(C)S(=O)(=O)C[C@H](C1CC1)N1C(=O)[C@@](C)(CC(=O)O)C[C@H](c2cccc(Cl)c2)[C@H]1c1ccc(Cl)cc1	0.2	9.70
J. Med. Chem.	2014	CHEMBL3125710	CC[C@@H](CNS(C)(=O)=O)N1C(=O)[C@@](C)(CC(=O)O)C[C@H](c2cccc(Cl)c2)[C@H]1c1ccc(Cl)cc1	0.7	9.15
J. Med. Chem.	2014	CHEMBL3125513	CC[C@@H](CNS(=O)(=O)C(C)(C)C)N1C(=O)[C@@](C)(CC(=O)O)C[C@H](c2cccc(Cl)c2)[C@H]1c1ccc(Cl)cc1	1.3	8.89

J. Med. Chem.	2014	CHEMBL3125709	CC[C@@H](CC(C)(C)C#N)N1C(=O)[C@@](C)(CC(=O)O)C[C@H](c2cccc(Cl)c2)[C@H]1c1ccc(Cl)cc1	1.2	8.92
J. Med. Chem.	2014	CHEMBL3125522	CC[C@@H](CS(C)(=O)=O)N1C(=O)[C@@](C)(CC(=O)O)C[C@H](c2cccc(Cl)c2)[C@H]1c1ccc(Cl)cc1	0.3	9.52
MedChemComm	2013	CHEMBL3220087	Cc1cc(/C=C2\C(=O)NC(=S)N(c3ccccc3)C2=O)c(C)n1-c1ccc(Cl)cc1	3300.0	5.48
MedChemComm	2013	CHEMBL3220088	O=C1NC(=S)NC(=O)C1=Cc1cc(-c2ccccc2)n(-c2ccc(Cl)cc2)c1-c1ccccc1	110.0	6.96
MedChemComm	2013	CHEMBL3220089	O=C1NC(=S)N(c2ccccc2)C(=O)/C1=C/c1cc(-c2ccccc2)n(-c2ccc(Cl)cc2)c1-c1ccccc1	230.0	6.64
MedChemComm	2013	CHEMBL3220093	Cc1cc(C=C2C(=O)N(c3ccccc3)C(=S)N(c3ccccc3)C2=O)c(C)n1-c1ccc(Br)cc1	199.0	6.70
MedChemComm	2013	CHEMBL3220094	Cc1ccc(-n2c(C)cc(/C=C3\C(=O)NC(=S)N(c4ccccc4)C3=O)c2C)cc1	8400.0	5.08
MedChemComm	2013	CHEMBL3220096	CCOC(=O)c1ccc(-n2c(-c3ccccc3)cc(C=C3C(=O)NC(=S)NC3=O)c2-c2ccccc2)cc1	700.0	6.15
MedChemComm	2013	CHEMBL3220097	O=C1NC(=O)C(=Cc2cc(-c3ccccc3)n(-c3ccc(Cl)cc3)c2-c2ccccc2)C(=O)N1	300.0	6.52
MedChemComm	2013	CHEMBL3220098	O=C1NC(=O)C(=Cc2cc(-c3ccccc3)n(-c3ccc(Br)cc3)c2-c2ccccc2)C(=O)N1	180.0	6.74
MedChemComm	2013	CHEMBL3220099	COc1ccc(-n2c(-c3ccccc3)cc(C=C3C(=O)NC(=O)NC3=O)c2-c2ccccc2)cc1	1900.0	5.72
MedChemComm	2013	CHEMBL3220100	CC(C)(C)c1ccc(-n2c(-c3ccccc3)cc(C=C3C(=O)NC(=O)NC3=O)c2-c2ccccc2)cc1	1900.0	5.72
MedChemComm	2013	CHEMBL3220101	N#Cc1ccc(-n2c(-c3ccccc3)cc(C=C3C(=O)NC(=O)NC3=O)c2-c2ccccc2)cc1	4700.0	5.33

MedChemComm	2013	CHEMBL3220102	N#Cc1ccc(-n2c(-c3ccccc3)cc(C=C3C(=O)NC(=S)NC3=O)c2-c2ccccc2)cc1	200.0	6.70
MedChemComm	2013	CHEMBL3220103	O=C1NC(=O)C(=Cc2cc(-c3ccccc3)n(-c3ccc([N+](=O)[O-])cc3)c2-c2ccccc2)C(=O)N1	150.0	6.82
MedChemComm	2013	CHEMBL3217780	O=C1NC(=S)NC(=O)C1=Cc1cc(-c2ccccc2)n(-c2ccc([N+](=O)[O-])cc2)c1-c1ccccc1	170.0	6.77
MedChemComm	2013	CHEMBL3220104	CCN1C(=O)C(=Cc2cc(-c3ccccc3)n(-c3ccc(Cl)cc3)c2-c2ccccc2)C(=O)N(CC)C1=S	300.0	6.52
MedChemComm	2013	CHEMBL3220105	CCN1C(=O)C(=Cc2cc(-c3ccccc3)n(-c3ccc(Br)cc3)c2-c2ccccc2)C(=O)N(CC)C1=O	890.0	6.05
MedChemComm	2013	CHEMBL3220106	CCN1C(=O)C(=Cc2cc(-c3ccccc3)n(-c3ccc(Br)cc3)c2-c2ccccc2)C(=O)N(CC)C1=S	260.0	6.59
MedChemComm	2013	CHEMBL3220108	CC1(C)OC(=O)C(Cc2cc(-c3ccccc3)n(-c3ccc(Br)cc3)c2-c2ccccc2)C(=O)O1	2900.0	5.54
MedChemComm	2013	CHEMBL3220110	OCC(=Cc1cc(-c2ccccc2)n(-c2ccc(Cl)cc2)c1-c1ccccc1)CO	2900.0	5.54
MedChemComm	2013	CHEMBL3220111	NC(=O)C(=Cc1cc(-c2ccccc2)n(-c2ccc(Cl)cc2)c1-c1ccccc1)C(N)=O	2500.0	5.60
MedChemComm	2013	CHEMBL3220112	CNC(=O)C(=Cc1cc(-c2ccccc2)n(-c2ccc(Cl)cc2)c1-c1ccccc1)C(=O)NC	7600.0	5.12
MedChemComm	2013	CHEMBL3220115	CC(C)(C)c1cc(C=C2C(=O)NC(=S)NC2=O)c(-c2ccccc2)n1-c1ccc(Br)cc1	760.0	6.12
MedChemComm	2013	CHEMBL3220116	CC(C)(C)c1cc(C=C2C(=O)NC(=S)NC2=O)c(-c2ccccc2)n1-c1ccc(Cl)cc1	1100.0	5.96
MedChemComm	2013	CHEMBL3220117	O=C1NC(=S)NC(=O)C1=Cc1cc(-c2ccccc2)n(-c2ccc(Cl)cc2)c1C1CC1	1600.0	5.80
MedChemComm	2013	CHEMBL3220118	O=C1NC(=S)NC(=O)C1=Cc1cc(-c2ccccc2)n(-c2ccc(Br)cc2)c1C1CC1	1600.0	5.80

MedChemComm	2013	CHEMBL3220309	O=C1NC(=S)NC(=O)C1=Cc1cc(C2CC2)n(-c2ccc(Cl)cc2)c1-c1ccccc1	2100.0	5.68
MedChemComm	2013	CHEMBL3220310	O=C1NC(=S)NC(=O)C1=Cc1cc(C2CC2)n(-c2ccc(Br)cc2)c1-c1ccccc1	2200.0	5.66
MedChemComm	2013	CHEMBL3220313	CN(C)CCCN(C)C1CCN(C(=O)c2[nH]c3cc(Cl)ccc3c2-c2c(Cc3ccc(Cl)cc3)ncn2-c2ccccc2)C1	190.0	6.72
Bioorg. Med. Chem. Lett.	2014	CHEMBL3233587	COc1ccccc1N1CCN(C(=O)[C@]2(Oc3ccc(C(F)(F)F)cc3)CCCN(C(=O)c3cnccc3C(F)(F)F)C2)CC1	600.0	6.22
Bioorg. Med. Chem. Lett.	2014	CHEMBL3233589	COCCOc1ccccc1N1CCN(C(=O)[C@]2(Oc3ccc(C(F)(F)F)cc3)C[C@@H](C)CN(C(=O)c3cnccc3C(F)(F)F)C2)CC1	4000.0	5.40
Bioorg. Med. Chem. Lett.	2014	CHEMBL3233590	COCCOc1ccccc1N1CCN(C(=O)[C@]2(Oc3ccc(C(F)(F)F)cc3)C[C@H](C)CN(C(=O)c3cnccc3C(F)(F)F)C2)CC1	2700.0	5.57
Bioorg. Med. Chem. Lett.	2014	CHEMBL3233591	COCCOc1ccccc1N1CCN(C(=O)[C@@]2(Oc3ccc(C(F)(F)F)cc3)CN(C(=O)c3cnccc3C(F)(F)F)CC(C)(C)C2)CC1	1000.0	6.00
Bioorg. Med. Chem. Lett.	2014	CHEMBL3233592	COc1ccccc1N1CCN(C(=O)[C@]2(Oc3ccc(C(F)(F)F)cc3)C[C@H](c3ccccc3)CN(C(=O)c3cnccc3C(F)(F)F)C2)CC1	3000.0	5.52
Bioorg. Med. Chem. Lett.	2014	CHEMBL3233593	COCCOc1ccccc1N1CCN(C(=O)[C@@]2(Oc3ccc(C(F)(F)F)cc3)CN(C(=O)c3cnccc3C(F)(F)F)CC[C@H]2c2ccc(F)cc2)CC1	3900.0	5.41
Bioorg. Med.	2014	CHEMBL3233594	COCCOc1ccccc1N1CCN(C(=O)[C@]2(Oc3ccc(C(F)(F)F)cc3)CC[C@H](c3ccccc3)N(C(=O)c3cnccc3C(F)(F)F)C2)CC1	10000.0	5.00

Chem. Lett.					
Bioorg. Med. Chem. Lett.	2014	CHEMBL3233595	COc1ccccc1N1CCN(C(=O)[C@]2(Oc3ccc(C(F)(F)F)cc3)C[C@@H]3C[C@@H]3N(C(=O)c3cnccc3C(F)(F)F)C2)CC1	5600.0	5.25
J. Med. Chem.	2014	CHEMBL3233121	CC[C@@H](CN(C)S(=O)(=O)C1CC1)N1C(=O)[C@@H](CC(=O)O)O[C@H](c2cccc(Cl)c2)[C@H]1c1ccc(Cl)cc1	11.0	7.96
J. Med. Chem.	2014	CHEMBL3233122	CC[C@@H](CN(c1ccccc1)S(=O)(=O)C1CC1)N1C(=O)[C@@H](CC(=O)O)O[C@H](c2cccc(Cl)c2)[C@H]1c1ccc(Cl)cc1	3.7	8.43
J. Med. Chem.	2014	CHEMBL3233123	CC[C@@H](CN(c1ccccc1F)S(=O)(=O)C1CC1)N1C(=O)[C@@H](CC(=O)O)O[C@H](c2cccc(Cl)c2)[C@H]1c1ccc(Cl)cc1	2.3	8.64
J. Med. Chem.	2014	CHEMBL3233124	CC[C@@H](CN(c1ccc(F)cc1)S(=O)(=O)C1CC1)N1C(=O)[C@@H](CC(=O)O)O[C@H](c2cccc(Cl)c2)[C@H]1c1ccc(Cl)cc1	6.0	8.22
J. Med. Chem.	2014	CHEMBL3233125	CC[C@@H](CN(c1ccc(F)cc1F)S(=O)(=O)C1CC1)N1C(=O)[C@@H](CC(=O)O)O[C@H](c2cccc(Cl)c2)[C@H]1c1ccc(Cl)cc1	2.0	8.70
J. Med. Chem.	2014	CHEMBL3233126	CC[C@@H](CN(c1ccccc1F)S(=O)(=O)C(C)C)N1C(=O)[C@@H](CC(=O)O)O[C@H](c2cccc(Cl)c2)[C@H]1c1ccc(Cl)cc1	2.4	8.62
J. Med. Chem.	2014	CHEMBL3233127	CC[C@@H](CN(c1ccccc1F)S(C)(=O)=O)N1C(=O)[C@@H](CC(=O)O)O[C@H](c2cccc(Cl)c2)[C@H]1c1ccc(Cl)cc1	4.0	8.40
J. Med. Chem.	2014	CHEMBL3233128	CC[C@@H](CS(=O)(=O)CC)N1C(=O)[C@@H](CC(=O)O)O[C@H](c2cccc(Cl)c2)[C@H]1c1ccc(Cl)cc1	26.0	7.59
J. Med. Chem.	2014	CHEMBL3233129	CC[C@@H](CS(=O)(=O)C(C)C)N1C(=O)[C@@H](CC(=O)O)O[C@H](c2cccc(Cl)c2)[C@H]1c1ccc(Cl)cc1	10.1	8.00

J. Med. Chem.	2014	CHEMBL3233131	CC[C@@H](CS(=O)(=O)CC(C)(C)C)N1C(=O)[C@@H](CC(=O)O)O[C@H](c2cccc(Cl)c2)[C@H]1c1ccc(Cl)cc1	18.0	7.74
J. Med. Chem.	2014	CHEMBL3233132	CC[C@@H](CS(=O)(=O)C1CCCC1)N1C(=O)[C@@H](CC(=O)O)O[C@H](c2cccc(Cl)c2)[C@H]1c1ccc(Cl)cc1	20.0	7.70
J. Med. Chem.	2014	CHEMBL3233133	CC[C@@H](CS(=O)(=O)c1ccccc1)N1C(=O)[C@@H](CC(=O)O)O[C@H](c2cccc(Cl)c2)[C@H]1c1ccc(Cl)cc1	17.0	7.77
J. Med. Chem.	2014	CHEMBL3233134	CC[C@@H](CS(=O)(=O)N(C)C)N1C(=O)[C@@H](CC(=O)O)O[C@H](c2cccc(Cl)c2)[C@H]1c1ccc(Cl)cc1	10.0	8.00
J. Med. Chem.	2014	CHEMBL3233135	CC[C@@H](CS(=O)(=O)C1COC1)N1C(=O)[C@@H](CC(=O)O)O[C@H](c2cccc(Cl)c2)[C@H]1c1ccc(Cl)cc1	23.0	7.64
J. Med. Chem.	2014	CHEMBL3233136	C[C@@H](CS(=O)(=O)C(C)(C)C)N1C(=O)[C@@H](CC(=O)O)O[C@H](c2cccc(Cl)c2)[C@H]1c1ccc(Cl)cc1	6.9	8.16
J. Med. Chem.	2014	CHEMBL3233137	CC(C)(C)S(=O)(=O)C[C@H](CC(F)(F)F)N1C(=O)[C@@H](CC(=O)O)O[C@H](c2cccc(Cl)c2)[C@H]1c1ccc(Cl)cc1	2.1	8.68
J. Med. Chem.	2014	CHEMBL3233138	CC(C)[C@@H](CS(=O)(=O)C(C)(C)C)N1C(=O)[C@@H](CC(=O)O)O[C@H](c2cccc(Cl)c2)[C@H]1c1ccc(Cl)cc1	2.5	8.60
J. Med. Chem.	2014	CHEMBL3233139	CC(C)(C)[C@@H](CS(=O)(=O)C(C)(C)C)N1C(=O)[C@@H](CC(=O)O)O[C@H](c2cccc(Cl)c2)[C@H]1c1ccc(Cl)cc1	5.1	8.29
J. Med. Chem.	2014	CHEMBL3233141	CC[C@@H](CS(=O)(=O)C(C)(C)C)N1C(=O)[C@@](C)(CC(=O)O)O[C@H](c2cccc(Cl)c2)[C@H]1c1ccc(Cl)cc1	0.5	9.30
J. Med.	2014	CHEMBL3233140	CC(C)(C)S(=O)(=O)C[C@H](C1CC1)N1C(=O)[C@@H](CC(=O)O)O[C@H](c2cccc(Cl)c2)[C@H]1c1ccc(Cl)cc1	160.0	6.80

Chem.					
J. Med. Chem.	2014	CHEMBL3233130	CC[C@@H](CS(=O)(=O)C(C)(C)C)N1C(=O)[C@@H](CC(=O)O)O[C@H](c2cccc(Cl)c2)[C@H]1c1ccc(Cl)cc1	2.0	8.70
J. Med. Chem.	2014	CHEMBL3236666	CC[C@@H](CS(=O)(=O)C(C)(C)C)N1C(=O)[C@H](CC(=O)O)O[C@H](c2cccc(Cl)c2)[C@H]1c1ccc(Cl)cc1	9.0	8.05
J. Med. Chem.	2014	CHEMBL3236358	CC[C@@H](CS(=O)(=O)C(C)(C)C)N1C(=O)[C@@](C)(Cc2nnn[nH]2)C[C@H](c2cccc(Cl)c2)[C@H]1c1ccc(Cl)cc1	0.8	9.10
J. Med. Chem.	2014	CHEMBL3236359	CC[C@@H](CS(=O)(=O)C(C)(C)C)N1C(=O)[C@@](C)(Cc2noc(=O)[nH]2)C[C@H](c2cccc(Cl)c2)[C@H]1c1ccc(Cl)cc1	1.3	8.89
J. Med. Chem.	2014	CHEMBL3236360	CC[C@@H](CS(=O)(=O)C(C)(C)C)N1C(=O)[C@@](C)(CC(=O)NS(=O)(=O)C(F)(F)F)C[C@H](c2cccc(Cl)c2)[C@H]1c1ccc(Cl)cc1	23.0	7.64
J. Med. Chem.	2014	CHEMBL3236361	CC(C)(C)S(=O)(=O)C[C@H](C1CC1)N1C(=O)[C@@](C)(CC(=O)C(=O)O)C[C@H](c2cccc(Cl)c2)[C@H]1c1ccc(Cl)cc1	1.9	8.72
J. Med. Chem.	2014	CHEMBL3236362	CC[C@@H](CS(=O)(=O)C(C)(C)C)N1C(=O)[C@@](C)(CC(=O)CO)C[C@H](c2cccc(Cl)c2)[C@H]1c1ccc(Cl)cc1	3.7	8.43
J. Med. Chem.	2014	CHEMBL3236363	CC[C@@H](CS(=O)(=O)C(C)(C)C)N1C(=O)[C@@](C)(CC(N)=O)C[C@H](c2cccc(Cl)c2)[C@H]1c1ccc(Cl)cc1	2.1	8.68
J. Med. Chem.	2014	CHEMBL3236365	CC(C)(C)S(=O)(=O)C[C@H](C1CC1)N1C(=O)[C@@](C)(CS(N)(=O)=O)C[C@H](c2cccc(Cl)c2)[C@H]1c1ccc(Cl)cc1	14.0	7.85
J. Med. Chem.	2014	CHEMBL3236635	CC(C)(C)S(=O)(=O)C[C@H](C1CC1)N1C(=O)[C@@](C)(CC(=O)N2CC[C@@H](C(=O)O)C2)C[C@H](c2cccc(Cl)c2)[C@H]1c1ccc(Cl)cc1	1.8	8.74

J. Med. Chem.	2014	CHEMBL3236636	CC(C)(C)S(=O)(=O)C[C@H](C1CC1)N1C(=O)[C@@](C)(CC(=O)N2CC[C@H](C(=O)O)C2)C[C@H](c2cccc(Cl)c2)[C@H]1c1ccc(Cl)cc1	0.7	9.15
J. Med. Chem.	2014	CHEMBL3236641	CC(C)(C)S(=O)(=O)C[C@H](C1CC1)N1C(=O)[C@@](C)(Cc2cccc(C(=O)O)n2)C[C@H](c2cccc(Cl)c2)[C@H]1c1ccc(Cl)cc1	24.0	7.62
J. Med. Chem.	2014	CHEMBL3236643	CC(C)(C)S(=O)(=O)C[C@H](C1CC1)N1C(=O)[C@@](C)(Cc2ccc(CC(=O)O)cn2)C[C@H](c2cccc(Cl)c2)[C@H]1c1ccc(Cl)cc1	2.0	8.70
J. Med. Chem.	2014	CHEMBL3236647	CC(C)(C)S(=O)(=O)C[C@H](C1CC1)N1C(=O)[C@@](C)(Cc2ncc(C(=O)O)cn2)C[C@H](c2cccc(Cl)c2)[C@H]1c1ccc(Cl)cc1	0.8	9.10
J. Med. Chem.	2014	CHEMBL3236648	CC(C)(C)S(=O)(=O)C[C@H](C1CC1)N1C(=O)[C@@](C)(Cc2ncc(C(=O)O)cn2)C[C@H](c2cccc(Cl)c2)[C@H]1c1ccc(Cl)c(F)c1	0.9	9.05
J. Med. Chem.	2014	CHEMBL3236649	CC(C)(C)S(=O)(=O)C[C@H](C1CC1)N1C(=O)[C@@](C)(Cc2cnc(C(=O)O)cn2)C[C@H](c2cccc(Cl)c2)[C@H]1c1ccc(Cl)cc1	0.7	9.15
J. Med. Chem.	2014	CHEMBL3236651	CC(C)(C)S(=O)(=O)C[C@H](C1CC1)N1C(=O)[C@@](C)(Cn2cc(C(=O)O)cn2)C[C@H](c2cccc(Cl)c2)[C@H]1c1ccc(Cl)cc1	1.0	9.00
J. Med. Chem.	2014	CHEMBL3236652	CC(C)(C)S(=O)(=O)C[C@H](C1CC1)N1C(=O)[C@@](C)(Cc2nc(C(=O)O)cs2)C[C@H](c2cccc(Cl)c2)[C@H]1c1ccc(Cl)cc1	8.1	8.09
J. Med. Chem.	2014	CHEMBL3236653	CC(C)(C)S(=O)(=O)C[C@H](C1CC1)N1C(=O)[C@@](C)(Cc2ncc(C(=O)O)s2)C[C@H](c2cccc(Cl)c2)[C@H]1c1ccc(Cl)cc1	0.7	9.15
J. Med. Chem.	2014	CHEMBL3236654	CC(C)(C)S(=O)(=O)C[C@H](C1CC1)N1C(=O)[C@@](C)(Cc2ncc(C(=O)CO)s2)C[C@H](c2cccc(Cl)c2)[C@H]1c1ccc(Cl)cc1	1.7	8.77
J. Med.	2014	CHEMBL3236657	CC(C)(C)S(=O)(=O)C[C@H](C1CC1)N1C(=O)[C@@](C)(Cc2ncc(CC(=O)O)s2)C[C@H](c2cccc(Cl)c2)[C@H]1c1ccc(Cl)cc1	1.1	8.96

Chem.					
J. Med. Chem.	2014	CHEMBL3236662	CC(C)(C)S(=O)(=O)C[C@H](C1CC1)N1C(=O)[C@@](C)(Cc2ncc(CC(=O)O)o2)C[C@H](c2cccc(Cl)c2)[C@H]1c1ccc(Cl)c(F)c1	1.3	8.89
J. Med. Chem.	2014	CHEMBL3236663	CC(C)(C)S(=O)(=O)C[C@H](C1CC1)N1C(=O)[C@@](C)(Cc2nnc(CC(=O)O)[nH]2)C[C@H](c2cccc(Cl)c2)[C@H]1c1ccc(Cl)cc1	1.0	9.00
J. Med. Chem.	2014	CHEMBL3236664	CC(C)(C)S(=O)(=O)C[C@H](C1CC1)N1C(=O)[C@@](C)(Cc2ncc(CC(=O)O)[nH]2)C[C@H](c2cccc(Cl)c2)[C@H]1c1ccc(Cl)cc1	1.5	8.82
J. Med. Chem.	2014	CHEMBL3233049	CC(C)(C)S(=O)(=O)C[C@H](C1CC1)N1C(=O)[C@@](C)(Cc2ccc(C(=O)O)cn2)O[C@H](c2cccc(Cl)c2)[C@H]1c1ccc(Cl)cc1	1.5	8.82
J. Med. Chem.	2014	CHEMBL3236665	CC(C)(C)S(=O)(=O)C[C@H](C1CC1)N1C(=O)[C@](C)(Cc2ccc(C(=O)O)cn2)O[C@H](c2cccc(Cl)c2)[C@H]1c1ccc(Cl)cc1	1.0	9.00
J. Med. Chem.	2014	CHEMBL3236667	CC(C)(C)S(=O)(=O)C[C@H](C1CC1)N1C(=O)[C@](C)(Cc2ccc(C(=O)O)cn2)C[C@H](c2cccc(Cl)c2)[C@H]1c1ccc(Cl)cc1	8.0	8.10
J. Med. Chem.	2014	CHEMBL3236356	CC(C)(C)S(=O)(=O)C[C@H](C1CC1)N1C(=O)[C@@](C)(Cc2ccc(C(=O)O)cn2)C[C@H](c2cccc(Cl)c2)[C@H]1c1ccc(Cl)cc1	5.0	8.30
J. Med. Chem.	2014	CHEMBL3236639	CC(C)(C)S(=O)(=O)C[C@H](C1CC1)N1C(=O)[C@@](C)(Cc2cc(C(=O)O)ccn2)C[C@H](c2cccc(Cl)c2)[C@H]1c1ccc(Cl)cc1	47.0	7.33
J. Med. Chem.	2014	CHEMBL3236640	CC(C)(C)S(=O)(=O)C[C@H](C1CC1)N1C(=O)[C@@](C)(Cc2nc(CC(=O)O)cs2)C[C@H](c2cccc(Cl)c2)[C@H]1c1ccc(Cl)cc1	1.1	8.96
Bioorg. Med. Chem. Lett.	2014	CHEMBL3260822	CC(C)(C)NC(=O)Cc1ccc(Cl)c(F)c1-n1c(C2CCCCC2)nc(-c2nnc(N)o2)c1-c1ccc(F)c(Cl)c1	2.0	8.70

Bioorg. Med. Chem. Lett.	2014	CHEMBL3260813	O=C(O)c1nc(-c2ccccc2)n(-c2cccc(Cl)c2F)c1-c1ccc(F)c(Cl)c1	3.0	8.52
Bioorg. Med. Chem. Lett.	2014	CHEMBL3260816	Fc1ccc(-c2c(-c3nnn[nH]3)nc(C3CCCCC3)n2-c2cccc(Cl)c2F)cc1Cl	3.0	8.52
Bioorg. Med. Chem. Lett.	2014	CHEMBL3260814	O=C(O)c1nc(C2CCCCC2)n(-c2cccc(Cl)c2F)c1-c1ccc(F)c(Cl)c1	4.0	8.40
Bioorg. Med. Chem. Lett.	2014	CHEMBL3260819	CNC(=O)Cc1ccc(Cl)c(F)c1-n1c(C2CCCCC2)nc(-c2nnc(N)o2)c1-c1ccc(F)c(Cl)c1	4.0	8.40
Bioorg. Med. Chem. Lett.	2014	CHEMBL3260818	Nc1nnc(-c2nc(C3CCCCC3)n(-c3cccc(Cl)c3F)c2-c2ccc(F)c(Cl)c2)o1	6.0	8.22
Bioorg. Med. Chem. Lett.	2014	CHEMBL3260811	O=C(O)c1nc(-c2ccccc2)n(-c2cccc(Cl)c2F)c1-c1cccc(Cl)c1	7.0	8.15
Bioorg. Med. Chem. Lett.	2014	CHEMBL3260812	Cc1cccc(-c2nc(C(=O)O)c(-c3cccc(Cl)c3)n2-c2cc(Cl)ccc2C)c1	8.0	8.10
Bioorg. Med. Chem. Lett.	2014	CHEMBL3260810	Cc1ccc(Cl)cc1-n1c(-c2ccccc2)nc(C(=O)O)c1-c1cccc(Cl)c1	11.0	7.96
Bioorg. Med.	2014	CHEMBL3260815	NC(=O)c1nc(C2CCCCC2)n(-c2cccc(Cl)c2F)c1-c1ccc(F)c(Cl)c1	18.0	7.74

Chem. Lett.					
Bioorg. Med. Chem. Lett.	2014	CHEMBL3260809	O=C(O)c1nc(-c2ccccc2)n(-c2cccc(Cl)c2)c1-c1cccc(Cl)c1	120.0	6.92
Eur. J. Med. Chem.	2014	CHEMBL3291221	Cc1[nH]c(-c2ccccc2)c(-c2ccccc2)c1-c1ccnc2ncnn12	890.0	6.05
Eur. J. Med. Chem.	2014	CHEMBL3291220	Cc1[nH]c(-c2ccccc2)c(-c2ccccc2)c1-c1ccnc2c(C#N)cnn12	910.0	6.04
Eur. J. Med. Chem.	2014	CHEMBL3291219	Cc1[nH]c(-c2ccccc2)c(-c2ccccc2)c1-c1ccon1	940.0	6.03
Eur. J. Med. Chem.	2014	CHEMBL3291218	Cc1[nH]c(-c2ccccc2)c(-c2ccccc2)c1-c1ccnn1-c1ccccc1	970.0	6.01
Eur. J. Med. Chem.	2014	CHEMBL3291217	Cc1[nH]c(-c2ccccc2)c(-c2ccccc2)c1-c1ccn[nH]1	990.0	6.00
Eur. J. Med. Chem.	2014	CHEMBL3291222	Cc1[nH]c(-c2ccccc2)c(-c2ccccc2)c1-c1ccnc2nnnn12	840.0	6.08
Eur. J. Med. Chem.	2014	CHEMBL3286447	Cc1[nH]c(-c2ccccc2)c(-c2ccccc2)c1-c1ccnc2nc3ccccc3n12	360.0	6.44
Eur. J. Med. Chem.	2014	CHEMBL3291433	Cc1[nH]c(-c2ccccc2)c(-c2ccccc2)c1-c1ccnc2[nH]c(=S)[nH]c(=O)c12	440.0	6.36
Eur. J. Med. Chem.	2014	CHEMBL3291434	CC(=O)c1ccc(-c2c(C)[nH]c(-c3ccccc3)c2-c2ccccc2)nc1C	810.0	6.09

Eur. J. Med. Chem.	2014	CHEMBL3291435	CCOC(=O)c1ccc(-c2c(C)[nH]c(-c3ccccc3)c2-c2ccccc2)nc1C	780.0	6.11
Eur. J. Med. Chem.	2014	CHEMBL3291437	Cc1[nH]c(-c2ccccc2)c(-c2ccccc2)c1-c1ccc(C#N)c(=O)[nH]1	570.0	6.24
Eur. J. Med. Chem.	2014	CHEMBL3291438	CCOC(=O)c1ccc(-c2c(C)[nH]c(-c3ccccc3)c2-c2ccccc2)[nH]c1=O	550.0	6.26
Eur. J. Med. Chem.	2014	CHEMBL3291439	COc1ccc(-c2nc(-c3ccc(Cl)cc3)c(-c3ccc(Cl)cc3)n2C(=O)N2CCN(CCO)CC2)c(OC(C)C)c1	260.0	6.59
ACS Med. Chem. Lett.	2014	CHEMBL3288447	COCCOc1ccccc1N1CCN(C(=O)[C@]2(Oc3ccc(C(F)(F)F)cc3)CCCN(C(=O)c3cnccc3C(F)(F)F)C2)CC1	169.0	6.77
ACS Med. Chem. Lett.	2014	CHEMBL3288448	C=CC[C@H]1N(C(=O)c2cnccc2C(F)(F)F)CCC[C@@]1(Oc1ccc(C(F)(F)F)cc1)C(=O)N1CCN(c2ccccc2OCCOC)CC1	41.0	7.39
ACS Med. Chem. Lett.	2014	CHEMBL3288449	CCC[C@H]1N(C(=O)c2cnccc2C(F)(F)F)CCC[C@@]1(Oc1ccc(C(F)(F)F)cc1)C(=O)N1CCN(c2ccccc2OCCOC)CC1	24.0	7.62
ACS Med. Chem. Lett.	2014	CHEMBL3288450	COCCOc1ccccc1N1CCN(C(=O)[C@]2(Oc3ccc(C(F)(F)F)cc3)CCCN(C(=O)c3cnccc3C(F)(F)F)[C@@H]2CC(O)CO)CC1	16.0	7.80
ACS Med. Chem. Lett.	2014	CHEMBL3288451	COCCOc1ccccc1N1CCN(C(=O)[C@]2(Oc3ccc(C(F)(F)F)cc3)CCCN(C(=O)c3cnccc3C(F)(F)F)[C@@H]2CCO)CC1	24.0	7.62
MedChemComm	2011	CHEMBL1236726	CN(C)CCCN(C)[C@H]1CCN(C(=O)c2[nH]c3cc(Cl)ccc3c2-c2c(-c3ccccc3)ncn2Cc2ccc(Cl)cc2)C1	190.0	6.72
J. Med. Chem.	2014	CHEMBL3355410	CC1(C)N[C@@H](C(=O)N[C@H]2CC[C@H](O)CC2)[C@H](c2cccc(Cl)c2F)[C@]12C(=O)Nc1cc(Cl)ccc12	151.0	6.82

J. Med. Chem.	2014	CHEMBL3355411	CCC1(CC)N[C@@H](C(=O)N[C@H]2CC[C@H](O)CC2)[C@H](c2cccc(Cl)c2F)[C@]12C(=O)Nc1cc(Cl)ccc12	33.0	7.48
J. Med. Chem.	2014	CHEMBL3355412	CCCC1(CCC)N[C@@H](C(=O)N[C@H]2CC[C@H](O)CC2)[C@H](c2cccc(Cl)c2F)[C@]12C(=O)Nc1cc(Cl)ccc12	775.0	6.11
J. Med. Chem.	2014	CHEMBL3355413	O=C(N[C@H]1CC[C@H](O)CC1)[C@@H]1NC2(CCC2)[C@]2(C(=O)Nc3cc(Cl)ccc32)[C@H]1c1cccc(Cl)c1F	1719.0	5.76
J. Med. Chem.	2014	CHEMBL3355415	O=C(N[C@H]1CC[C@H](O)CC1)[C@@H]1NC2(CCCC2)[C@@]2(C(=O)Nc3cc(Cl)ccc32)[C@H]1c1cccc(Cl)c1F	35.0	7.46
J. Med. Chem.	2014	CHEMBL3355416	O=C(N[C@H]1CC[C@H](O)CC1)[C@@H]1NC2(CCCCC2)[C@@]2(C(=O)Nc3cc(Cl)ccc32)[C@H]1c1cccc(Cl)c1F	17.2	7.76
J. Med. Chem.	2014	CHEMBL3355417	O=C(N[C@H]1CC[C@H](O)CC1)[C@@H]1NC2(CCCCCC2)[C@@]2(C(=O)Nc3cc(Cl)ccc32)[C@H]1c1cccc(Cl)c1F	15.6	7.81
J. Med. Chem.	2014	CHEMBL3355426	CCC1(CC)N[C@@H](C(=O)N[C@H]2C[C@@](C)(O)C2)[C@H](c2cccc(Cl)c2F)[C@]12C(=O)Nc1cc(Cl)ccc12	118.0	6.93
J. Med. Chem.	2014	CHEMBL3355427	C[C@]1(O)C[C@@H](NC(=O)[C@@H]2NC3(CCC3)[C@]3(C(=O)Nc4cc(Cl)ccc43)[C@H]2c2cccc(Cl)c2F)C1	7505.0	5.12
J. Med. Chem.	2014	CHEMBL3355428	C[C@]1(O)C[C@@H](NC(=O)[C@@H]2NC3(CCC3)[C@@]3(C(=O)Nc4cc(Cl)ccc43)[C@H]2c2cccc(Cl)c2F)C1	396.0	6.40
J. Med. Chem.	2014	CHEMBL3355429	C[C@]1(O)C[C@@H](NC(=O)[C@@H]2NC3(CCCC3)[C@@]3(C(=O)Nc4cc(Cl)ccc43)[C@H]2c2cccc(Cl)c2F)C1	20.0	7.70
Bioorg. Med.	2014	CHEMBL3318759	C[C@]1(CC(=O)O)C[C@H](c2cccc(Cl)c2)[C@@H](c2ccc(Cl)cc2)N([C@H](CS(=O)(=O)NCC2CCC2)C2CC2)C1=O	0.2	9.77

Chem. Lett.					
Bioorg. Med. Chem. Lett.	2014	CHEMBL3318755	CNS(=O)(=O)C[C@H](C1CC1)N1C(=O)[C@@](C)(CC(=O)O)C[C@H](c2cccc(Cl)c2)[C@H]1c1ccc(Cl)cc1	1.0	9.01
Bioorg. Med. Chem. Lett.	2014	CHEMBL3318763	C[C@]1(CC(=O)O)C[C@H](c2cccc(Cl)c2)[C@@H](c2ccc(Cl)cc2)N([C@H](CS(=O)(=O)NC2CCCC2)C2CC2)C1=O	0.1	9.92
Bioorg. Med. Chem. Lett.	2014	CHEMBL3318765	CCN(CC)S(=O)(=O)C[C@H](C1CC1)N1C(=O)[C@@](C)(CC(=O)O)C[C@H](c2cccc(Cl)c2)[C@H]1c1ccc(Cl)cc1	0.2	9.82
Bioorg. Med. Chem. Lett.	2014	CHEMBL3318766	C[C@]1(CC(=O)O)C[C@H](c2cccc(Cl)c2)[C@@H](c2ccc(Cl)cc2)N([C@H](CS(=O)(=O)N2CCCCC2)C2CC2)C1=O	0.6	9.19
Bioorg. Med. Chem. Lett.	2014	CHEMBL3318769	C[C@]1(CC(=O)O)C[C@H](c2cccc(Cl)c2)[C@@H](c2ccc(Cl)cc2)N([C@H](CS(=O)(=O)Nc2ccccc2F)C2CC2)C1=O	0.1	9.92
Bioorg. Med. Chem. Lett.	2014	CHEMBL3318770	C[C@]1(CC(=O)O)C[C@H](c2cccc(Cl)c2)[C@@H](c2ccc(Cl)cc2)N([C@H](CS(=O)(=O)Nc2ccccn2)C2CC2)C1=O	0.2	9.80
Bioorg. Med. Chem. Lett.	2014	CHEMBL3318772	C[C@]1(CC(=O)O)C[C@H](c2cccc(Cl)c2)[C@@H](c2ccc(Cl)cc2)N([C@H](CS(=O)(=O)N2CCc3ccccc32)C2CC2)C1=O	0.5	9.28
Bioorg. Med. Chem. Lett.	2014	CHEMBL3318773	CC(C)[C@@H](CS(=O)(=O)N1CCCC1)N1C(=O)[C@@](C)(CC(=O)O)C[C@H](c2cccc(Cl)c2)[C@H]1c1ccc(Cl)cc1	0.3	9.52

Bioorg. Med. Chem. Lett.	2014	CHEMBL3318774	CC(C)(C)[C@@H](CS(=O)(=O)N1CCCC1)N1C(=O)[C@@](C)(CC(=O)O)C[C@H](c2cccc(Cl)c2)[C@H]1c1ccc(Cl)cc1	0.3	9.51
Bioorg. Med. Chem. Lett.	2014	CHEMBL3318775	CN(C)S(=O)(=O)C[C@@H](N1C(=O)[C@@](C)(CC(=O)O)C[C@H](c2cccc(Cl)c2)[C@H]1c1ccc(Cl)cc1)C(C)(C)C	0.2	9.62
Bioorg. Med. Chem. Lett.	2014	CHEMBL3318776	CCN(C)S(=O)(=O)C[C@@H](N1C(=O)[C@@](C)(CC(=O)O)C[C@H](c2cccc(Cl)c2)[C@H]1c1ccc(Cl)cc1)C(C)(C)C	0.4	9.40
Bioorg. Med. Chem. Lett.	2014	CHEMBL3318777	CN(C1CC1)S(=O)(=O)C[C@@H](N1C(=O)[C@@](C)(CC(=O)O)C[C@H](c2cccc(Cl)c2)[C@H]1c1ccc(Cl)cc1)C(C)(C)C	0.4	9.42
Bioorg. Med. Chem. Lett.	2014	CHEMBL3318778	CC(C)(C)[C@@H](CS(=O)(=O)N1CCC(F)(F)C1)N1C(=O)[C@@](C)(CC(=O)O)C[C@H](c2cccc(Cl)c2)[C@H]1c1ccc(Cl)cc1	0.5	9.33
Bioorg. Med. Chem. Lett.	2014	CHEMBL3318779	C[C@@H]1CCCN1S(=O)(=O)C[C@@H](N1C(=O)[C@@](C)(CC(=O)O)C[C@H](c2cccc(Cl)c2)[C@H]1c1ccc(Cl)cc1)C(C)(C)C	0.2	9.82
Bioorg. Med. Chem. Lett.	2014	CHEMBL3318780	C[C@H]1CCCN1S(=O)(=O)C[C@@H](N1C(=O)[C@@](C)(CC(=O)O)C[C@H](c2cccc(Cl)c2)[C@H]1c1ccc(Cl)cc1)C(C)(C)C	1.1	8.96
Bioorg. Med. Chem. Lett.	2014	CHEMBL3318784	CC(C)(C)[C@@H](CS(=O)(=O)N1CCC(F)(F)CC1)N1C(=O)[C@@](C)(CC(=O)O)C[C@H](c2cccc(Cl)c2)[C@H]1c1ccc(Cl)cc1	0.4	9.43
Bioorg. Med.	2014	CHEMBL3318785	CC(C)(C)[C@@H](CS(=O)(=O)N1CCc2ccccc21)N1C(=O)[C@@](C)(CC(=O)O)C[C@H](c2cccc(Cl)c2)[C@H]1c1ccc(Cl)cc1	0.4	9.38

Chem. Lett.					
Bioorg. Med. Chem. Lett.	2014	CHEMBL3318787	CC(C)(C)[C@@H](CS(=O)(=O)N1CCc2cc(F)ccc21)N1C(=O)[C@@](C)(CC(=O)O)C[C@H](c2cccc(Cl)c2)[C@H]1c1ccc(Cl)cc1	0.2	9.66
Bioorg. Med. Chem.	2015	CHEMBL2398246	CC(C)C1=C(C(=O)N2[C@H](C(=O)N3CCN(C)[C@H](C)C3)CC[C@H]2C)SC2=N[C@@](C)(c3ccc(Cl)cc3)[C@@H](c3ccc(Cl)cc3)N21	15.0	7.82
Bioorg. Med. Chem.	2015	CHEMBL3402515	CC(C)C1=C(C(=O)N2[C@H](C(=O)N3CCN(C)C4(CC4)C3)CC[C@H]2C)SC2=N[C@@](C)(c3ccc(Cl)cc3)[C@@H](c3ccc(Cl)cc3)N21	21.0	7.68
Bioorg. Med. Chem.	2015	CHEMBL3402516	CC(C)C1=C(C(=O)N2[C@H](C(=O)N3CCNC4(CC4)C3)CC[C@H]2C)SC2=N[C@@](C)(c3ccc(Cl)cc3)[C@@H](c3ccc(Cl)cc3)N21	9.5	8.02
Bioorg. Med. Chem.	2015	CHEMBL3402517	CC(C)C1=C(C(=O)N2[C@H](C(=O)N3CCNC4(CC4)C3)CC[C@H]2C)SC2=N[C@@](C)(c3ccc(Cl)cc3)[C@@H](c3ccc(Cl)c(F)c3)N21	7.6	8.12
Bioorg. Med. Chem.	2015	CHEMBL3402518	CC(C)C1=C(C(=O)N2[C@H](C(=O)N3CCNC4(CC4)C3)CC[C@H]2C)SC2=N[C@@](C)(c3ccc(Cl)nc3)[C@@H](c3ccc(Cl)c(F)c3)N21	15.0	7.82
Bioorg. Med. Chem.	2015	CHEMBL3402519	CC(C)C1=C(C(=O)N2[C@H](C(=O)N3CC4(CC4)NC[C@@H]3C)CC[C@H]2C)SC2=N[C@@](C)(c3ccc(Cl)nc3)[C@@H](c3ccc(Cl)c(F)c3)N21	11.0	7.96
Bioorg. Med. Chem.	2015	CHEMBL3402520	CC(C)C1=C(C(=O)N2C[C@H](F)C[C@H]2C(=O)N2CC3(CC3)NC[C@@H]2C)SC2=N[C@@](C)(c3ccc(Cl)nc3)[C@@H](c3ccc(Cl)c(F)c3)N21	2.2	8.66

Bioorg. Med. Chem.	2015	CHEMBL3402521	CC[C@@H]1CNC2(CC2)CN1C(=O)[C@@H]1C[C@@H](F)CN1C(=O)C1=C(C(C)C)N2C(=N[C@@](C)(c3ccc(Cl)nc3)[C@H]2c2ccc(Cl)c(F)c2)S1	2.4	8.62
J. Med. Chem.	2014	CHEMBL3407574	CC(C)(C)S(=O)(=O)C[C@H](C1CC1)N1C(=O)[C@@](C)(CC(=O)N2CC(C(=O)O)C2)C[C@H](c2cccc(Cl)c2)[C@H]1c1ccc(Cl)cc1	0.3	9.59
J. Med. Chem.	2014	CHEMBL3407573	CC(C)(C)S(=O)(=O)C[C@H](C1CC1)N1C(=O)[C@@](C)(CC(=O)N[C@H]2C[C@@H](C(=O)O)C2)C[C@H](c2cccc(Cl)c2)[C@H]1c1ccc(Cl)cc1	0.8	9.08
J. Med. Chem.	2014	CHEMBL3407572	CC(C)(C)S(=O)(=O)C[C@H](C1CC1)N1C(=O)[C@@](C)(CC(=O)N[C@H]2C[C@H](C(=O)O)C2)C[C@H](c2cccc(Cl)c2)[C@H]1c1ccc(Cl)cc1	0.5	9.28
J. Med. Chem.	2014	CHEMBL3407571	CC1(C(=O)O)CCN(C(=O)C[C@@]2(C)C[C@H](c3cccc(Cl)c3)[C@@H](c3ccc(Cl)cc3)N([C@H](CS(=O)(=O)C(C)(C)C)C3CC3)C2=O)CC1	0.5	9.31
J. Med. Chem.	2014	CHEMBL3407570	CC(C)(C)S(=O)(=O)C[C@H](C1CC1)N1C(=O)[C@@](C)(CC(=O)N2CCC(C(=O)O)CC2)C[C@H](c2cccc(Cl)c2)[C@H]1c1ccc(Cl)cc1	0.8	9.10
J. Med. Chem.	2014	CHEMBL3407568	CC(C)(C)S(=O)(=O)C[C@H](C1CC1)N1C(=O)[C@@](C)(CC(=O)N[C@H]2CC[C@@H](C(=O)O)CC2)C[C@H](c2cccc(Cl)c2)[C@H]1c1ccc(Cl)cc1	0.4	9.38
J. Med. Chem.	2014	CHEMBL3407567	CC(C)(C)S(=O)(=O)C[C@H](C1CC1)N1C(=O)[C@@](C)(CC(=O)N[C@H]2CC[C@H](C(=O)O)CC2)C[C@H](c2cccc(Cl)c2)[C@H]1c1ccc(Cl)cc1	0.7	9.14
J. Med. Chem.	2014	CHEMBL3407564	CC(C)(C)S(=O)(=O)C[C@H](C1CC1)N1C(=O)[C@@](C)(CC(=O)Nc2ccc(C(=O)O)cn2)C[C@H](c2cccc(Cl)c2)[C@H]1c1ccc(Cl)cc1	0.2	9.68
J. Med. Chem.	2014	CHEMBL3407562	COc1cc(NC(=O)C[C@@]2(C)C[C@H](c3cccc(Cl)c3)[C@@H](c3ccc(Cl)cc3)N([C@H](CS(=O)(=O)C(C)(C)C)C3CC3)C2=O)ccc1C(N)=O	0.5	9.31

J. Med. Chem.	2014	CHEMBL3236364	CC(C)(C)S(=O)(=O)C[C@H](C1CC1)N1C(=O)[C@@](C)(CC(N)=O)C[C@H](c2cccc(Cl)c2)[C@H]1c1ccc(Cl)cc1	0.2	9.77
J. Med. Chem.	2014	CHEMBL3125537	CC(C)(C)S(=O)(=O)C[C@H](C1CC1)N1C(=O)[C@@](C)(CC(=O)O)C[C@H](c2cccc(Cl)c2)[C@H]1c1ccc(Cl)cc1	1.1	8.96
J. Med. Chem.	2015	CHEMBL407632	CCOc1cc(OC)ccc1C1=N[C@@H](c2ccc(Br)cc2)[C@@H](c2ccc(Br)cc2)N1C(=O)N1CCN(CCO)CC1	140.0	6.85
Bioorg. Med. Chem. Lett.	2015	CHEMBL3627748	COc1ccc(-c2cn(CCC[C@H](NC(=O)OCC3c4ccccc4-c4ccccc43)C(=O)O)nn2)cc1	5660.0	5.25
Bioorg. Med. Chem. Lett.	2015	CHEMBL3627747	O=C(N[C@@H](CCCn1cc(-c2ccc(I)cc2)nn1)C(=O)O)OCC1c2ccccc2-c2ccccc21	2900.0	5.54
Bioorg. Med. Chem. Lett.	2015	CHEMBL3627746	O=C(N[C@@H](CCCn1cc(-c2ccc(Br)cc2)nn1)C(=O)O)OCC1c2ccccc2-c2ccccc21	5510.0	5.26
	2015	CHEMBL3687236	CC(C)CCn1nc2c(c1C(C)C)C(c1ccc(F)c(F)c1)N(c1cccc(Cl)c1F)C2=O	16.6	7.78
	2014	CHEMBL3653217	COc1nc(N2CC(O)C2)ncc1-c1nc2c(n1C(C)C)C(c1ccc(Cl)cc1)N(c1cc(Cl)ccc1C)C2=O	0.1	9.89
	2014	CHEMBL3653290	CCOC(=O)Cc1cnc(OC)c(-c2nc3c(n2C(C)C)C(c2ccc(C#N)cc2)N(c2cc(Cl)ccc2C)C3=O)c1	1.6	8.79
	2014	CHEMBL3653211	[C-]#[N+]c1ccc(C2c3c(nc(-c4cnc(N(C)C)nc4OC)n3C(C)C)C(=O)N2c2cccc(Cl)c2F)cc1	0.2	9.72
	2015	CHEMBL3691748	COc1ncc(-n2nc3c(c2C(C)C)C(c2ccc(Cl)cc2)N(c2cc(Cl)c[nH]c2=O)C3=O)c(OC)n1	0.2	9.63

	2014	CHEMBL3657098	COc1ncc(-c2nc3c(n2C(C)C)C(c2ccc(Cl)cc2C)N(c2cc(Cl)cn(C)c2=O)C3=O)c(OC)n1	0.4	9.38
	2014	CHEMBL3653225	COc1ccc(C(=O)N2CCOCC2)cc1-c1nc2c(n1C(C)C)C(c1ccc(Cl)cc1)N(c1cccc(Cl)c1F)C2=O	0.2	9.74
	2014	CHEMBL3639521	COc1ncc(-c2nc3c(n2C(C)C)C(c2ccc(Cl)cc2)N(c2ccc(C#N)cc2C)C3=O)c(OC)n1	5.0	8.30
	2014	CHEMBL3653295	COc1nc(N)ncc1-c1nc2c(n1C(C)C)[C@@H](c1ccc(C#N)cc1)N(c1cccc(Cl)c1F)C2=O	105.6	6.98
	2015	CHEMBL3687295	Cc1cc(Cl)ccc1C1c2c(nn(C3CCN(C)CC3)c2C(C)C)C(=O)N1c1cc(Cl)ccc1C	3.0	8.52
	2015	CHEMBL3687237	CCN1CCN(CCn2nc3c(c2C(C)C)C(c2ccc(Cl)cc2)N(c2cccc(Cl)c2F)C3=O)C1=O	8.9	8.05
	2015	CHEMBL3687269	Cc1cc(Cl)ccc1C1c2c(nn(CC(C)(C)O)c2C(C)C)C(=O)N1c1cccc(Cl)c1F	4.0	8.40
	2015	CHEMBL3683172	COc1cc2c(cc1OC(C)C)[C@H](c1ccc(Cl)cc1)N(c1ccc(N(C)C[C@H]3CC[C@H](N4CC(=O)N(C)C4)CC3)cc1)C(=O)C2	1.8	8.74
	2014	CHEMBL3657023	COc1ncc(-c2nc3c(n2C(C)C)C(c2ccc(Cl)cc2)N(c2cccc(Cl)c2F)C3=O)c(OC)n1	0.2	9.82
	2014	CHEMBL3657105	COc1nc(N(C)C)ncc1-c1nc2c(n1C(C)C)C(c1ccc(Cl)cc1F)N(c1cc(Cl)cn(C)c1=O)C2=O	0.3	9.50
	2015	CHEMBL3691787	COc1nc(N(C)C)ncc1-n1nc2c(c1C(C)C)C(c1ccc(C#N)c(F)c1)N(c1cc(Cl)c(=O)n(C)c1)C2=O	0.2	9.67
	2014	CHEMBL3653238	COc1ncc(-c2nc3c(n2C(C)C)C(c2ccc(C#N)cc2)N(c2ccc(F)c(Cl)c2)C3=O)c(OC)n1	0.4	9.40
	2014	CHEMBL3657110	COc1cn(C)c(=O)cc1-c1nc2c(n1C(C)C)C(c1ccc(Cl)cc1)N(c1c(F)c(C3CC3)nn1C)C2=O	3.2	8.49
	2014	CHEMBL3653192	COc1nc(N(C)C)ncc1-c1nc2c(n1C(C)C)C(c1ccc(Cl)cc1)N(c1cc(Cl)ccc1C)C2=O	0.1	9.89

	2015	CHEMBL3687245	CC(=O)N1CCN(C(=O)Cn2nc3c(c2C(C)C)C(c2ccc(Cl)cc2C)N(c2cc(Cl)ccc2C)C3=O)CC1	2.9	8.54
	2014	CHEMBL3653272	COc1nc(N)ncc1-c1nc2c(n1C(C)C)C(c1ccc(Cl)cc1)N(c1ccc(C#N)cc1C)C2=O	4.3	8.37
	2014	CHEMBL3657080	COc1ncc(-c2nc3c(n2C(C)C)C(c2ccc(C#N)c(F)c2)N(c2cc(Cl)cn(C)c2=O)C3=O)c(OC)n1	1.0	9.00
	2015	CHEMBL3687316	COc1ccccc1-n1nc2c(c1C(C)C)C(C)(c1ccc(Cl)cc1)N(c1ccc(F)c(Cl)c1)C2=O	1.6	8.80
	2014	CHEMBL3653256	COc1ncc(-c2nc3c(n2C(C)C)[C@H](c2ccc(Cl)cc2)N(c2cc(Cl)cn(C)c2=O)C3=O)c(OC)n1	0.2	9.64
	2015	CHEMBL3683158	COc1cc2c(cc1OC(C)C)[C@H](c1ccc(Cl)cc1)N(c1ccc(N(C)C[C@H]3CC[C@H](NC(=O)c4cocn4)CC3)cc1)C(=O)C2	4.9	8.31
	2015	CHEMBL3691785	[C-]#[N+]c1ccc(C2c3c(nn(-c4cnc(OC)nc4OC)c3C(C)C)C(=O)N2c2cc(Cl)cn(C)c2=O)c(F)c1	10.3	7.99
	2014	CHEMBL3653223	COc1nc(N)ncc1-c1nc2c(n1C(C)C)C(c1ccc(Cl)cc1)N(c1cc(Cl)c(=O)n(C)c1)C2=O	1.2	8.94
	2014	CHEMBL3657060	COc1cnc(CC#N)cc1-c1nc2c(n1C(C)C)[C@@H](c1ccc(Cl)cc1)N(c1cccc(Cl)c1F)C2=O	4.4	8.36
	2015	CHEMBL3691791	COc1nc(N(C)C)ncc1-n1nc2c(c1C(C)C)C(c1ccc(Cl)c(F)c1)N(c1cc(Cl)c[nH]c1=O)C2=O	0.2	9.78
	2015	CHEMBL3691781	COc1ncc(-n2nc3c(c2C(C)C)C(c2ccc(Cl)cc2F)N(c2cc(Cl)cn(C)c2=O)C3=O)c(OC)n1	1.4	8.84
	2015	CHEMBL3683163	COc1cc2c(cc1OC(C)C)[C@H](c1ccc(Cl)cc1)N(c1ccc(N(C)C[C@H]3CC[C@H](N4CCCNC(=O)C4)CC3)cc1)C(=O)C2	1.4	8.85
	2014	CHEMBL3653177	COc1cc(CO)ccc1-c1nc2c(n1C(C)C)C(c1ccc(Cl)cc1)N(c1cc(Cl)ccc1C)C2=O	0.1	9.92
	2014	CHEMBL3657109	COc1ncc(-c2nc3c(n2C(C)C)C(c2ccc(Cl)cc2)N(c2c(F)c(C4CC4)nn2C)C3=O)c(OC)n1	1.8	8.75

	2015	CHEMBL3683110	CC[C@@H](C)Oc1cc2c(cc1OC)NC(=O)N(c1ccc(N(C)C)cc1)C2c1ccc(Cl)cc1	1309.5	5.88
	2015	CHEMBL3687230	Cc1cc(Cl)ccc1C1c2c(nn(Cc3cc(=O)[nH]c4c(C#N)cnn34)c2C(C)C)C(=O)N1c1cccc(Cl)c1F	18.6	7.73
	2014	CHEMBL3657053	COc1cnc(CC#N)cc1-c1nc2c(n1C(C)C)C(c1ccc(C#N)cc1C)N(c1cc(Cl)ccc1C)C2=O	0.3	9.57
	2014	CHEMBL3657095	COc1nc(N(C)C)ncc1-c1nc2c(n1C(C)C)C(c1ccc(Cl)cc1)N(c1cc(C)cn(C)c1=O)C2=O	0.3	9.60
	2015	CHEMBL3639820	COc1cc2c(cc1OC(C)C)[C@H](c1ccc(Cl)cc1)N(c1ccc(Cn3ccnc3)cc1)C(=O)C2	104.0	6.98
	2014	CHEMBL3656997	COc1ncc(-c2nc3c(n2C(C)C)C(c2ccc(C#N)cc2C)N(c2cccc(Cl)c2F)C3=O)c(OC)n1	0.2	9.74
	2015	CHEMBL3601323	COc1cc2c(cc1OC(C)C)[C@H](c1ccc(Cl)cc1)N(c1ccc(N(C)C[C@H]3CC[C@H](N(C)C)CC3)cc1)C(=O)C2	2.5	8.60
	2014	CHEMBL3653249	COc1cnc(CC#N)cc1-c1nc2c(n1C(C)C)C(c1ccc(Cl)cc1C)N(c1cccc(Cl)c1F)C2=O	0.4	9.44
	2014	CHEMBL3657091	COc1nc(N(C)C)ncc1-c1nc2c(n1C(C)C)[C@H](c1ccc(C#N)c(F)c1)N(c1cc(Cl)cn(C)c1=O)C2=O	0.2	9.82
	2015	CHEMBL3687294	Cc1cc(Cl)ccc1C1c2c(nn(-c3cc(=O)n(C)c(=O)n3C)c2C(C)C)C(=O)N1c1cc(Cl)ccc1C	24.4	7.61
	2014	CHEMBL3657029	COc1ccc(C#N)cc1-c1nc2c(n1C(C)C)C(c1ccc(Cl)cc1C)N(c1cccc(Cl)c1)C2=O	0.4	9.37
	2015	CHEMBL3691752	COc1nc(N(C)C)ncc1-n1nc2c(c1C(C)C)C(c1ccc(Cl)cc1)N(c1c[nH]c(=O)c(Cl)c1)C2=O	0.1	9.89
	2014	CHEMBL3653172	COc1cnccc1-c1nc2c(n1C(C)C)C(c1ccc(Cl)cc1C)N(c1cc(Cl)ccc1C)C2=O	0.2	9.66
	2014	CHEMBL3657028	COc1ncc(-c2nc3c(n2C(C)C)C(c2ccc(Cl)cc2C)N(c2cccc(Cl)c2)C3=O)c(OC)n1	0.5	9.30

	2015	CHEMBL3687258	COCCn1nc2c(c1C(C)C)C(c1ccc(Cl)cc1C)N(c1cccc(Cl)c1F)C2=O	4.2	8.38
	2015	CHEMBL3687305	Cc1cc(Cl)ccc1C1c2c(nn(-c3cccc(C#N)c3)c2C(C)C)C(=O)N1c1cccc(Cl)c1F	8.4	8.08
	2014	CHEMBL3653201	[C-]#[N+]c1ccc(C2c3c(nc(-c4cnc(N)nc4OC)n3C(C)C)C(=O)N2c2cc(Cl)c(=O)n(C)c2)cc1	3.0	8.53
	2015	CHEMBL3691695	COc1ccccc1-n1nc2c(c1C(C)C)C(CC(O)CO)(c1ccc(Cl)cc1)N(c1ccc(F)c(Cl)c1)C2=O	1.5	8.82
	2015	CHEMBL3687232	Cc1cc(Cl)ccc1C1c2c(nn(CCc3ccccn3)c2C(C)C)C(=O)N1c1cccc(Cl)c1F	9.9	8.00
	2015	CHEMBL3683144	CC[C@@H](C)Oc1cc2c(cc1OC)CC(=O)N(c1ccc(C(C)N(CC)C(=O)C3CCNCC3)cc1)C2c1ccc(Cl)cc1	47.2	7.33
	2015	CHEMBL3687311	COc1ccccc1-n1nc2c(c1C(C)C)C(c1ccc(Cl)cc1)N(c1ccc(F)c(Cl)c1)C2=O	2.3	8.64
	2015	CHEMBL3687227	Cc1cc(Cl)ccc1C1c2c(nn(Cc3cccnc3)c2C(C)C)C(=O)N1c1cccc(Cl)c1F	11.3	7.95
	2014	CHEMBL3657092	COc1ncc(-c2nc3c(n2C(C)C)C(c2ccc(Cl)c(F)c2)N(c2cc(Cl)c(=O)n(C)c2)C3=O)c(OC)n1	0.3	9.60
	2015	CHEMBL3691758	COc1ncc(-n2nc3c(c2C(C)C)C(c2ccc(C#N)cc2)N(c2cc(Cl)cn(C)c2=O)C3=O)c(OC)n1	1.4	8.84
	2014	CHEMBL3653165	COc1ncccc1-c1nc2c(n1C(C)C)C(c1ccc(Cl)cc1C)N(c1cc(Cl)ccc1C)C2=O	2.3	8.64
	2014	CHEMBL3653261	COc1cnc(CC#N)cc1-c1nc2c(n1C(C)C)C(c1ccc(Cl)cc1)N(c1cc(Cl)ccc1C)C2=O	0.3	9.49
	2015	CHEMBL3683128	CC[C@@H](C)Oc1cc2c(cc1OC)CC(=O)N(c1ccc(C(=O)N(C)CC)cc1)C2c1ccc(Cl)cc1	190.4	6.72
	2015	CHEMBL3601319	CC[C@@H](C)Oc1cc2c(cc1OC)CC(=O)N(c1ccc(N(C)CC3CCNCC3)cc1)C2c1ccc(Cl)cc1	3.5	8.46

	2015	CHEMBL3691719	CC(C)(C)c1[nH]nc2c1C(c1ccc(Cl)cc1)N(c1cccc(Cl)c1F)C2=O	110.1	6.96
	2014	CHEMBL3657099	COc1nc(=O)[nH]cc1-c1nc2c(n1C(C)C)C(c1ccc(Cl)cc1)N(c1cc(Cl)cn(C)c1=O)C2=O	10.2	7.99
	2014	CHEMBL3650074	COc1cc2c(cc1OC(C)C)C(c1ccc(Cl)cc1)N(C1CCC(C(C)(O)CC(C)C)CC1)C(=O)C2	3.9	8.41
	2014	CHEMBL3650065	COc1cc2c(cc1OC(C)C)[C@H](c1ccc(Cl)cc1)N(C1CCC(C(=O)C3CCOCC3)CC1)C(=O)C2	386.0	6.41
	2014	CHEMBL3657094	COc1ncc(-c2nc3c(n2C(C)C)C(c2ccc(Cl)cc2)N(c2cc(C)cn(C)c2=O)C3=O)c(OC)n1	1.2	8.94
	2015	CHEMBL3687263	CNC(=O)Cn1nc2c(c1C(C)C)C(c1ccc(Cl)cc1C)N(c1cccc(Cl)c1F)C2=O	11.4	7.94
	2014	CHEMBL3657078	COc1ncc(-c2nc3c(n2C(C)C)C(c2ccc(Cl)cn2)N(c2cc(Cl)ccc2C)C3=O)c(OC)n1	2.8	8.55
	2015	CHEMBL3691716	CC(C)c1c2c(nn1-c1cn[nH]c1)C(=O)N(c1cccc(Cl)c1F)C2c1ccc(Cl)cc1	22.5	7.65
	2015	CHEMBL3601320	CC[C@@H](C)Oc1cc2c(cc1OC)CC(=O)N(c1ccc(N(C)C[C@H]3CC[C@H](N)CC3)cc1)C2c1ccc(Cl)cc1	3.7	8.43
	2014	CHEMBL3657112	COc1nc(N2CCC[C@H](O)C2)ncc1-c1nc2c(n1C(C)C)C(c1ccc(C#N)cc1)N(c1cccc(Cl)c1F)C2=O	0.3	9.50
	2014	CHEMBL3653226	COc1ccc(C(=O)N(C)C)cc1-c1nc2c(n1C(C)C)C(c1ccc(Cl)cc1)N(c1cccc(Cl)c1F)C2=O	0.1	9.85
	2014	CHEMBL3653206	COc1ncncc1-c1nc2c(n1C(C)C)C(c1ccc(Cl)cc1)N(c1cc(Cl)ccc1C)C2=O	0.4	9.41
	2015	CHEMBL3683106	CC[C@@H](C)Oc1cc2c(cc1OC)CC(=O)N(c1ccc(N(C)C)cc1)C2c1ccc(Cl)cc1CN	5660.2	5.25
	2015	CHEMBL3683160	COc1cc2c(cc1OC(C)C)[C@H](c1ccc(Cl)cc1)N(c1ccc(N(C)C[C@H]3CC[C@H](N4CCOCC4)CC3)cc1)C(=O)C2	3.3	8.48

	2015	CHEMBL3683155	CC[C@@H](C)Oc1cc2c(cc1OC)CC(=O)N(c1ccc(C(C)N3CCOCC3=O)cc1)C2c1ccc(Cl)cc1	26.9	7.57
	2015	CHEMBL3683183	COc1cc2c(cc1OCC1CCCO1)[C@H](c1ccc(Cl)cc1)N(c1ccc(N(C)C[C@H]3CC[C@H](N4CC(=O)N(C)C4)CC3)nc1)C(=O)C2	3.8	8.42
	2014	CHEMBL3656978	COc1nc(N(C)C)ncc1-c1nc2c(n1C(C)C)[C@H](c1ccc(C#N)cc1)N(c1cc(Cl)cn(C)c1=O)C2=O	0.3	9.48
	2014	CHEMBL3653259	COc1cn(C)c(=O)cc1-c1nc2c(n1C(C)C)C(c1ccc(Cl)cc1)N(c1ccc(F)c(Cl)c1)C2=O	0.3	9.51
	2014	CHEMBL3657107	COc1ncc(-c2nc3c(n2C(C)C)[C@H](c2ccc(Cl)cc2)N(c2cc(Cl)c(=O)n(C)c2)C3=O)c(OC)n1	0.1	9.91
	2015	CHEMBL3687291	CC(C)c1c2c(nn1-c1ccncc1)C(=O)N(c1cccc(Cl)c1F)C2c1ccc(Cl)cc1	107.9	6.97
	2014	CHEMBL3657021	COc1nc(N)ncc1-c1nc2c(n1C(C)C)[C@@H](c1ccc(Cl)cc1)N(c1cc(Cl)ccc1C)C2=O	82.5	7.08
	2015	CHEMBL3691786	COc1nc(N(C)C)ncc1-n1nc2c(c1C(C)C)C(c1ccc(C#N)c(F)c1)N(c1cc(Cl)cn(C)c1=O)C2=O	0.4	9.42
	2014	CHEMBL3650055	COc1cc2c(cc1OC(C)C)C(c1ccc(Cl)cc1)N(C1CCC(N(C)C(=O)CCO)CC1)C(=O)C2	406.0	6.39
	2015	CHEMBL3683121	CCC(=O)Nc1ccc2c(c1)C(c1ccc(Cl)cc1)N(c1ccc(N(C)Cc3ccncc3)cc1)C(=O)C2	331.6	6.48
	2014	CHEMBL3657127	COc1ncc(-c2nc3c(n2C(C)C)[C@@H](c2ccc(C#N)c(F)c2)N(c2ccc(F)c(Cl)c2)C3=O)c(OC)n1	0.4	9.36
	2015	CHEMBL3687239	Cc1cc(Cn2nc3c(c2C(C)C)C(c2ccc(Cl)cc2)N(c2cccc(Cl)c2F)C3=O)n(C)n1	7.9	8.10
	2015	CHEMBL3687244	CC(=O)N1CCC(NC(=O)Cn2nc3c(c2C(C)C)C(c2ccc(Cl)cc2C)N(c2cc(Cl)ccc2C)C3=O)CC1	2.8	8.55
	2014	CHEMBL3650076	COc1cc2c(cc1OC(C)C)C(c1ccc(Cl)cc1)N(C1CCC(C(C)(O)CCC(C)C)CC1)C(=O)C2	7.0	8.15

	2014	CHEMBL3657008	COc1nc(N)ncc1-c1nc2c(n1C(C)C)C(c1ccc(Cl)cc1)N(c1ccc(F)c(Cl)c1)C2=O	0.5	9.31
	2015	CHEMBL3604660	CCOc1cc2c(cc1OCC)C(c1ccc(Cl)cc1)N(c1ccc(C)cc1OCc1nn[nH]n1)C(=O)C2	388.9	6.41
	2014	CHEMBL3650059	COc1cc2c(cc1OC(C)C)C(c1ccc(Cl)cc1)N(C1CCC(N(C(C)=O)C3CCOCC3)CC1)C(=O)C2	602.0	6.22
	2014	CHEMBL3653260	COc1cnc(CC#N)cc1-c1nc2c(n1C(C)C)C(c1ccc(Cl)cc1C)N(c1cc(Cl)ccc1C)C2=O	0.3	9.55
	2014	CHEMBL3653294	COc1nc(N)ncc1-c1nc2c(n1C(C)C)[C@H](c1ccc(C#N)cc1)N(c1cccc(Cl)c1F)C2=O	0.4	9.44
	2014	CHEMBL3657093	COc1ncc(-c2nc3c(n2C(C)C)C(c2ccc(Cl)cc2)N(c2cc(C)cc(=O)n2C)C3=O)c(OC)n1	2.4	8.62
	2015	CHEMBL3687241	CC(C)c1c2c(nn1Cc1nnn[nH]1)C(=O)N(c1cccc(Cl)c1F)C2c1ccc(Cl)cc1	16.2	7.79
	2014	CHEMBL3657043	COc1ccccc1-c1nc2c(n1C(C)C)C(c1ccc(Cl)cc1C)N(c1cc(Cl)c(=O)n(C)c1)C2=O	0.8	9.11
	2015	CHEMBL3691747	COc1nc(N(C)C)ncc1-n1nc2c(c1C(C)C)C(c1ccc(C#N)cc1)N(c1cc(Cl)cn(C)c1=O)C2=O	0.4	9.43
	2015	CHEMBL3687262	Cc1cc(Cl)ccc1C1c2c(nn(Cc3ccccn3)c2C(C)C)C(=O)N1c1cc(Cl)ccc1C	2.6	8.59
	2015	CHEMBL3687259	Cc1cc(Cl)ccc1C1c2c(nn(Cc3ccncc3)c2C(C)C)C(=O)N1c1cccc(Cl)c1F	6.2	8.21
	2014	CHEMBL3657005	COc1ccc(C(=O)N(C)C)cc1-c1nc2c(n1C(C)C)[C@H](c1ccc(Cl)cc1)N(c1cc(Cl)ccc1C)C2=O	0.1	9.89
	2014	CHEMBL3653308	COC[C@H](C)n1c(-c2cnc(OC)nc2OC)nc2c1C(c1ccc(Cl)cc1)N(c1cc(Cl)ccc1C)C2=O	1.3	8.90
	2015	CHEMBL3687290	COc1ccccc1-n1nc2c(c1C(C)C)C(c1ccc(F)c(F)c1)N(c1cccc(Cl)c1F)C2=O	3.3	8.48

	2014	CHEMBL3657010	COc1ccc(C(=O)N2CCOCC2)cc1-c1nc2c(n1C(C)C)C(c1ccc(Cl)cc1)N(c1ccc(F)c(Cl)c1)C2=O	0.2	9.82
	2015	CHEMBL3683157	COc1cc2c(cc1OC(C)C)[C@H](c1ccc(Cl)cc1)N(c1ccc(N(C)C[C@H]3CC[C@H](NS(C)(=O)=O)CC3)cc1)C(=O)C2	3.6	8.44
	2015	CHEMBL3683150	CC[C@@H](C)Oc1cc2c(cc1OC)CC(=O)N(c1ccc(C(C)N[C@H]3CC[C@H](N)CC3)cc1)C2c1ccc(Cl)cc1	106.0	6.97
	2014	CHEMBL3657059	COc1cnc(CC#N)cc1-c1nc2c(n1C(C)C)[C@H](c1ccc(Cl)cc1)N(c1cccc(Cl)c1F)C2=O	0.1	9.89
	2014	CHEMBL3653196	[C-]#[N+]c1ccc(C2c3c(nc(-c4cnc(N)nc4OC)n3C(C)C)C(=O)N2c2cc(Cl)ccc2C)cc1	0.7	9.13
	2015	CHEMBL3687318	COc1ccccc1-n1nc2c(c1C(C)C)C(CC(O)CN1CCCC1)(c1ccc(Cl)cc1)N(c1ccc(F)c(Cl)c1)C2=O	3.4	8.47
	2015	CHEMBL3691770	CNc1ncc(-n2nc3c(c2C(C)C)C(c2ccc(C#N)cc2)N(c2cc(Cl)c(=O)n(C)c2)C3=O)c(OC)n1	0.7	9.18
	2015	CHEMBL3691760	COc1ncc(-n2nc3c(c2C(C)C)C(c2ccc(Cl)cc2)N(c2cc(Cl)c(=O)n(C)c2)C3=O)c(OC)n1	0.5	9.34
	2015	CHEMBL3691773	COc1ncc(-n2nc3c(c2C(C)C)[C@@H](c2ccc(Cl)cc2)N(c2cc(Cl)c(=O)n(C)c2)C3=O)c(OC)n1	266.8	6.57
	2014	CHEMBL3653292	COc1ncc(CC(=O)O)cc1-c1nc2c(n1C(C)C)C(c1ccc(Cl)cc1)N(c1cccc(Cl)c1F)C2=O	1.3	8.88
	2014	CHEMBL3653224	COc1ccc(C(=O)N(C)C)cc1-c1nc2c(n1C(C)C)C(c1ccc(Cl)cc1)N(c1cc(Cl)c(=O)n(C)c1)C2=O	0.7	9.15
	2014	CHEMBL3657068	COC[C@@H](C)n1c(-c2cnc(OC)nc2OC)nc2c1C(c1ccc(Cl)cc1)N(c1ccc(F)c(Cl)c1)C2=O	0.2	9.82
	2014	CHEMBL3650054	COc1cc2c(cc1OC(C)C)C(c1ccc(Cl)cc1)N(C1CCC(N3CCNC3=O)CC1)C(=O)C2	681.0	6.17
	2015	CHEMBL3687260	Cc1cc(Cl)ccc1C1c2c(nn(Cc3cscn3)c2C(C)C)C(=O)N1c1cccc(Cl)c1F	8.8	8.06

	2014	CHEMBL3653167	CNC(=O)c1ccc(OC)c(-c2nc3c(n2C(C)C)C(c2ccc(Cl)cc2C)N(c2cc(Cl)ccc2C)C3=O)c1	0.2	9.70
	2014	CHEMBL3657044	CCn1cc(N2C(=O)c3nc(-c4ccccc4OC)n(C(C)C)c3C2c2ccc(Cl)cc2C)cc(Cl)c1=O	1.0	9.01
	2015	CHEMBL3687256	CC(C)c1c2c(nn1Cc1ccccc1C#N)C(=O)N(c1ccc(F)c(F)c1)C2c1ccc(Cl)cc1	19.3	7.71
	2014	CHEMBL3657082	COc1nc(N(C)C)ncc1-c1nc2c(n1C(C)C)[C@@H](c1ccc(Cl)cc1)N(c1cc(Cl)cn(C)c1=O)C2=O	417.1	6.38
	2015	CHEMBL3683184	COc1cc2c(cc1OC(C)C)[C@H](c1ccc(Cl)cc1)N(c1ccc([C@H](C)N3CCN(C(C)=O)CC3=O)cc1)C(=O)C2	33.0	7.48
	2014	CHEMBL3657039	COc1ccccc1-c1nc2c(n1C(C)C)C(c1ccc(Cl)cc1C)N(c1cc(Cl)cnc1C)C2=O	0.4	9.43
	2014	CHEMBL3653283	COc1ccc(CC#N)cc1-c1nc2c(n1C(C)C)C(c1ccc(C#N)cc1)N(c1cccc(Cl)c1F)C2=O	0.2	9.74
	2015	CHEMBL3691735	Cc1cc(Cl)ccc1[C@H]1c2c(nn(CCO)c2C(C)C)C(=O)N1c1cccc(Cl)c1F	2.6	8.59
	2015	CHEMBL3687280	Cc1cc(Cl)ccc1C1c2c(nn(CCO)c2C(C)C)C(=O)N1c1cccc(Cl)c1F	5.9	8.23
	2015	CHEMBL3683105	CC[C@@H](C)Oc1cc2c(cc1OC)CC(=O)N(c1ccc(N(C)C)cc1)C2c1ccc(Cl)cc1CO	723.2	6.14
	2014	CHEMBL3657014	COc1ncc(-c2nc3c(n2C(C)C)[C@H](c2ccc(C#N)cc2)N(c2cccc(Cl)c2F)C3=O)c(OC)n1	0.2	9.80
	2014	CHEMBL3653279	CNC(=O)Cc1cc(-c2nc3c(n2C(C)C)C(c2ccc(Cl)cc2)N(c2cccc(Cl)c2F)C3=O)c(OC)cn1	0.2	9.62
	2015	CHEMBL3691700	COc1ccccc1-n1nc2c(c1C(C)C)C(c1ccc(Cl)cc1C)N(c1cc(Cl)cc(C(N)=O)c1)C2=O	2.1	8.68
	2014	CHEMBL3650071	COc1cc2c(cc1OC(C)C)C(c1ccc(Cl)cc1)N(C1CCC(C(=O)c3cccc(F)c3)CC1)C(=O)C2	531.0	6.27

	2014	CHEMBL3653188	COc1ccc(-c2nc3c(n2C(C)C)C(c2ccc(Cl)cc2)N(c2cc(Cl)ccc2C)C3=O)c(OC)n1	0.2	9.72
	2014	CHEMBL3653200	[C-]#[N+]c1ccc(C2c3c(nc(-c4cnc(NC)nc4OC)n3C(C)C)C(=O)N2c2cc(Cl)c(=O)n(C)c2)cc1	0.6	9.22
	2015	CHEMBL3687247	Cc1cc(Cl)ccc1C1c2c(nn(CC(=O)N3CCN(C)C(=O)C3)c2C(C)C)C(=O)N1c1cc(Cl)ccc1C	3.0	8.52
	2015	CHEMBL3683143	CC[C@@H](C)Oc1cc2c(cc1OC)CC(=O)N(c1ccc(C(C)N(CC)C(=O)C3CCN(C(C)=O)CC3)cc1)C2c1ccc(Cl)cc1	51.2	7.29
	2015	CHEMBL3691728	COc1ncc(-n2nc3c(c2C(C)C)C(c2ccc(Cl)cc2C)N(c2cc(Cl)c(=O)n(C)c2)C3=O)c(OC)n1	0.5	9.35
	2014	CHEMBL3657126	COc1ncc(CC(=O)O)cc1-c1nc2c(n1C(C)C)C(c1ccc(C#N)cc1)N(c1cccc(Cl)c1F)C2=O	4.7	8.32
	2015	CHEMBL3687314	COc1ccccc1-n1nc2c(c1C(C)C)C(c1ccc(Cl)cc1C)N(c1cncc(Cl)c1)C2=O	83.3	7.08
	2014	CHEMBL3657064	COc1cnc(CC#N)cc1-c1nc2c(n1C(C)C)[C@@H](c1ccc(C#N)cc1)N(c1cccc(Cl)c1F)C2=O	60.4	7.22
	2015	CHEMBL3683151	CC[C@@H](C)Oc1cc2c(cc1OC)CC(=O)N(c1ccc(C(C)N3CCC(N(C)C)CC3)cc1)C2c1ccc(Cl)cc1	15.2	7.82
	2014	CHEMBL3657009	COc1ccc(C#N)cc1-c1nc2c(n1C(C)C)C(c1ccc(Cl)cc1)N(c1ccc(F)c(Cl)c1)C2=O	0.2	9.77
	2015	CHEMBL3683176	COc1cc2c(cc1OC(C)C)[C@H](c1ccc(Cl)cc1)N(c1ccc(N(C)C[C@H]3CC[C@H](N4CC(=O)N(C)C4)CC3)cn1)C(=O)C2	5.7	8.24
	2015	CHEMBL3683125	CC[C@@H](C)Oc1cc2c(cc1OC)CC(=O)N(c1ccc(N3CCCC3=O)cc1)C2c1ccc(Cl)cc1	592.7	6.23
	2014	CHEMBL3657057	CNc1ncc(-c2nc3c(n2C(C)C)C(c2ccc(C#N)c(F)c2)N(c2cccc(Cl)c2F)C3=O)c(OC)n1	0.2	9.82
	2015	CHEMBL3691729	COc1ncc(-n2nc3c(c2C(C)C)C(c2ccc(Cl)cc2C)N(c2cc(Cl)ccc2C)C3=O)c(OC)n1	0.2	9.66

	2015	CHEMBL3683140	CC[C@@H](C)Oc1cc2c(cc1OC)CC(=O)N(c1ccc(C(C)N3CCCC3)cc1)C2c1ccc(Cl)cc1	189.8	6.72
	2014	CHEMBL3653265	COC[C@@H](C)n1c(-c2cnc(OC)nc2OC)nc2c1C(c1ccc(Cl)cc1)N(c1cc(Cl)ccc1C)C2=O	0.3	9.60
	2015	CHEMBL3691736	Cc1cc(Cl)ccc1[C@@H]1c2c(nn(CCO)c2C(C)C)C(=O)N1c1cccc(Cl)c1F	2085.9	5.68
	2014	CHEMBL3657089	COc1nc(N(C)C)ncc1-c1nc2c(n1C(C)C)C(c1ccc(Cl)c(F)c1)N(c1cc(Cl)c(=O)n(C)c1)C2=O	0.1	9.93
	2014	CHEMBL3653273	COc1ncc(-c2nc3c(n2C(C)C)C(c2ccc(Cl)cc2)N(c2cc(Cl)ccc2C#N)C3=O)c(OC)n1	0.6	9.24
	2014	CHEMBL3657073	COc1nc(N)ncc1-c1nc2c(n1C(C)C)[C@@H](c1ccc(Cl)cc1)N(c1ccc(F)c(Cl)c1)C2=O	284.2	6.55
	2015	CHEMBL3683182	COc1cc2c(cc1OC1CCC1)[C@H](c1ccc(Cl)cc1)N(c1ccc(N(C)C[C@H]3CC[C@H](N4CC(=O)N(C)C4)CC3)nc1)C(=O)C2	2.1	8.68
	2015	CHEMBL3691753	COc1nc(N(C)C)ncc1-n1nc2c(c1C(C)C)C(c1ccc(C#N)cc1)N(c1cc(Cl)c[nH]c1=O)C2=O	0.2	9.78
	2014	CHEMBL3656985	COc1ncc(-c2nc3c(n2C(C)C)[C@@H](c2ccc(Cl)cc2)N(c2cc(Cl)ccc2C)C3=O)c(OC)n1	15.8	7.80
	2014	CHEMBL3657002	CCn1cc(OC)c(-c2nc3c(n2C(C)C)C(c2ccc(C#N)cc2)N(c2cc(Cl)ccc2C)C3=O)cc1=O	0.6	9.24
	2015	CHEMBL3687302	Cc1cc(Cl)ccc1C1c2c(nn(CCO)c2C(C)C)C(=O)N1c1ccc(F)c(Cl)c1	4.1	8.39
	2014	CHEMBL3657108	COc1ncc(-c2nc3c(n2C(C)C)[C@@H](c2ccc(Cl)cc2)N(c2cc(Cl)c(=O)n(C)c2)C3=O)c(OC)n1	125.3	6.90
	2015	CHEMBL3691771	CNc1ncc(-n2nc3c(c2C(C)C)C(c2ccc(C#N)cc2)N(c2c[nH]c(=O)c(Cl)c2)C3=O)c(OC)n1	0.4	9.44
	2015	CHEMBL3691789	COc1nc(N(C)C)ncc1-n1nc2c(c1C(C)C)C(c1ccc(C#N)c(F)c1)N(c1c[nH]c(=O)c(Cl)c1)C2=O	0.2	9.71

	2015	CHEMBL3683141	CC[C@@H](C)Oc1cc2c(cc1OC)CC(=O)N(c1ccc(C(C)N3CCOCC3)cc1)C2c1ccc(Cl)cc1	47.1	7.33
	2015	CHEMBL3691704	COc1ccccc1-n1nc2c(c1C(C)C)C(c1ccc(Cl)cc1)N(c1cc(Cl)c(=O)n(C)c1)C2=O	0.4	9.37
	2014	CHEMBL3653181	COc1ccc(C(=O)N2CCOCC2)cc1-c1nc2c(n1C(C)C)C(c1ccc(Cl)cc1)N(c1cc(Cl)ccc1C)C2=O	0.2	9.80
	2014	CHEMBL3650049	CCN(C(C)=O)C1CCC(N2C(=O)Cc3cc(OC)c(OC(C)C)cc3C2c2ccc(Cl)cc2)CC1	183.0	6.74
	2015	CHEMBL3691734	[C-]#[N+]c1ccc(C2c3c(nn(-c4cc(C#N)ccc4OC)c3C(C)C)C(=O)N2c2cc(Cl)ccc2C)c(C)c1	0.2	9.75
	2015	CHEMBL3691756	COc1ncc(-n2nc3c(c2C(C)C)C(c2ccc(C#N)cc2)N(c2cc(Cl)c[nH]c2=O)C3=O)c(OC)n1	1.2	8.91
	2014	CHEMBL3653180	COc1ccc(C#N)cc1-c1nc2c(n1C(C)C)C(c1ccc(Cl)cc1)N(c1cc(Cl)ccc1C)C2=O	0.2	9.70
	2014	CHEMBL3653212	[C-]#[N+]c1ccc(C2c3c(nc(-c4cnc(N)nc4OC)n3C(C)C)C(=O)N2c2ccc(F)c(Cl)c2)cc1	1.2	8.92
	2015	CHEMBL3683147	CC[C@@H](C)Oc1cc2c(cc1OC)CC(=O)N(c1ccc(C(C)N(C)C3CCNCC3)cc1)C2c1ccc(Cl)cc1	259.9	6.59
	2015	CHEMBL3691710	CNC(=O)c1ccc(OC)c(-n2nc3c(c2C(C)C)C(c2ccc(Cl)cc2C)N(c2cc(Cl)c(=O)n(C)c2)C3=O)c1	1.1	8.96
	2014	CHEMBL3653183	COc1ncc(-c2nc3c(n2C(C)C)C(c2ccc(Cl)cc2)N(c2cc(Cl)ccc2C)C3=O)c(OC)n1	0.1	9.86
	2014	CHEMBL3653278	COC(=O)N1CCC(N2C(=O)c3nc(-c4cnc(OC)nc4OC)n(C(C)C)c3C2c2ccc(Cl)cc2)CC1	5.0	8.30
	2014	CHEMBL3657055	COc1nn(C)c(=O)cc1-c1nc2c(n1C(C)C)C(c1ccc(Cl)cc1)N(c1cccc(Cl)c1F)C2=O	21.5	7.67
	2014	CHEMBL3653270	CCOC(=O)C1(c2ccc(Cl)cc2C)c2c(nc(-c3cnc(OC)nc3OC)n2C(C)C)C(=O)N1c1cc(Cl)ccc1C	2.3	8.64

	2014	CHEMBL3657085	COc1ncc(-c2nc3c(n2C(C)C)[C@@H](c2ccc(Cl)cc2)N(c2cc(Cl)c[nH]c2=O)C3=O)c(OC)n1	76.5	7.12
	2015	CHEMBL3691697	CNC(=O)c1ccc(OC)c(-n2nc3c(c2C(C)C)C(c2ccc(Cl)cc2C)N(c2cc(Cl)ccc2C)C3=O)c1	2.0	8.70
	2014	CHEMBL3656998	COc1ccccc1-c1nc2c(n1C(C)C)C(c1ccc(C#N)cc1C)N(c1cccc(Cl)c1F)C2=O	0.4	9.42
	2014	CHEMBL3650068	COc1cc2c(cc1OC(C)C)[C@H](c1ccc(Cl)cc1)N(C1CCC([C@@](C)(O)C3CCOCC3)CC1)C(=O)C2	12.5	7.90
	2014	CHEMBL3657101	COc1ncc(-c2nc3c(n2C(C)C)C(c2ccc(Cl)cc2)N(c2cc(Cl)cnc2OC)C3=O)c(OC)n1	0.2	9.61
	2015	CHEMBL3683167	COC(=O)[C@@H]1CCCN1[C@@H](C)c1ccc(N2C(=O)Cc3cc(OC)c(OC(C)C)cc3[C@@H]2c2ccc(Cl)cc2)cc1	216.7	6.66
	2014	CHEMBL3653252	COc1c[nH]c(=O)nc1-c1nc2c(n1C(C)C)C(c1ccc(Cl)cc1)N(c1cccc(Cl)c1F)C2=O	0.6	9.21
	2015	CHEMBL3687235	CC(C)CCn1nc2c(c1C(C)C)C(c1ccc(F)cc1)N(c1cccc(Cl)c1F)C2=O	31.4	7.50
	2014	CHEMBL3657016	COc1nc(N2CC(O)C2)ncc1-c1nc2c(n1C(C)C)C(c1ccc(Cl)cc1)N(c1cc(Cl)cn(C)c1=O)C2=O	0.3	9.59
	2014	CHEMBL3653189	CCc1nc(OC)ccc1-c1nc2c(n1C(C)C)C(c1ccc(Cl)cc1)N(c1cc(Cl)ccc1C)C2=O	0.5	9.33
	2015	CHEMBL3691733	[C-]#[N+]c1ccc(C2c3c(nn(-c4cc(C#N)ccc4OC)c3C(C)C)C(=O)N2c2cc(Cl)c(=O)n(C)c2)c(C)c1	1.0	8.99
	2014	CHEMBL3657050	COc1cnc(CC#N)cc1-c1nc2c(n1C(C)C)C(c1ccc(C#N)cc1C)N(c1cccc(Cl)c1F)C2=O	0.5	9.27
	2014	CHEMBL3653243	COc1ncc(-c2nc3c(n2C2CCC2)C(c2ccc(C#N)cc2)N(c2ccc(F)c(Cl)c2)C3=O)c(OC)n1	31.7	7.50
	2015	CHEMBL3683169	COc1cc2c(cc1OC(C)C)[C@H](c1ccc(Cl)cc1)N(c1ccc(C(C)CN3CCC(N(C)C)CC3)cc1)C(=O)C2	21.5	7.67

	2015	CHEMBL3691762	CNc1ncc(-n2nc3c(c2C(C)C)C(c2ccc(Cl)cc2)N(c2c[nH]c(=O)c(Cl)c2)C3=O)c(OC)n1	0.4	9.45
	2014	CHEMBL3653176	COc1ccc(CO)cc1-c1nc2c(n1C(C)C)C(c1ccc(Cl)cc1)N(c1cccc(Cl)c1F)C2=O	0.2	9.77
	2014	CHEMBL3656987	COc1cnc(C#N)nc1-c1nc2c(n1C(C)C)C(c1ccc(Cl)cc1)N(c1cc(Cl)ccc1C)C2=O	0.2	9.80
	2015	CHEMBL3691764	COc1ncc(-n2nc3c(c2C(C)C)[C@H](c2ccc(Cl)cc2)N(c2cc(Cl)c[nH]c2=O)C3=O)c(OC)n1	0.2	9.79
	2015	CHEMBL3691783	COc1ncc(-n2nc3c(c2C(C)C)C(c2ccc(Cl)c(F)c2)N(c2cc(Cl)cn(C)c2=O)C3=O)c(OC)n1	0.6	9.26
	2015	CHEMBL3687252	CC(=O)N1CCC(O)(Cn2nc3c(c2C(C)C)C(c2ccc(Cl)cc2C)N(c2cc(Cl)ccc2C)C3=O)CC1	3.1	8.51
	2015	CHEMBL3683164	COc1cc2c(cc1OC(C)C)[C@H](c1ccc(Cl)cc1)N(c1ccc([C@H](C)N3CCS(=O)(=O)CC3)cc1)C(=O)C2	33.3	7.48
	2015	CHEMBL3683165	COc1cc2c(cc1OC(C)C)[C@H](c1ccc(Cl)cc1)N(c1ccc([C@H](C)N3CCN(C(C)=O)CC3)cc1)C(=O)C2	34.9	7.46
	2015	CHEMBL3601316	CC[C@@H](C)Oc1cc2c(cc1OC)CC(=O)N(c1ccc(N(C)Cc3ccncc3)cc1)C2c1ccc(Cl)cc1	8.4	8.08
	2015	CHEMBL3687306	Cc1cc(Cl)ccc1C1c2c(nn(CCO)c2C(C)C)C(=O)N1c1cc(Cl)ccc1C	2.6	8.59
	2014	CHEMBL3657111	COc1nc(N2CCS(=O)(=O)CC2)ncc1-c1nc2c(n1C(C)C)C(c1ccc(C#N)cc1)N(c1cccc(Cl)c1F)C2=O	0.6	9.20
	2015	CHEMBL3687309	COc1ccccc1-n1nc2c(c1C1CC1)C(c1ccc(Cl)cc1C)N(c1cccc(Cl)c1F)C2=O	8.5	8.07
	2015	CHEMBL3687285	Cc1cc(Cl)ccc1C1c2c(nn(C3CCN(C)CC3)c2C(C)C)C(=O)N1c1cccc(Cl)c1F	20.7	7.68
	2015	CHEMBL3691692	CNC(=O)c1ccc(C)c(-n2nc3c(c2C(C)C)C(c2ccc(C(F)(F)F)cc2)N(c2cc(Cl)ccc2C)C3=O)c1	5.7	8.24

	2014	CHEMBL3657104	COc1ncc(-c2nc3c(n2C(C)C)C(c2ccc(Cl)cc2F)N(c2cc(Cl)cn(C)c2=O)C3=O)c(OC)n1	1.1	8.97
	2015	CHEMBL3683134	CC[C@@H](C)Oc1cc2c(cc1OC)CC(=O)N(c1ccc(N(C)CC3CCCN(C)C3)cc1)C2c1ccc(Cl)cc1	9.1	8.04
	2014	CHEMBL3653287	COc1cnc(CC#N)cc1-c1nc2c(n1C(C)C)C(c1ccc(C#N)cc1)N(c1cccc(Cl)c1F)C2=O	0.7	9.16
	2014	CHEMBL3653293	COc1ncc(-c2nc3c(n2C(C)C)C(c2ccc(Cl)cc2)N(c2cnc(O)c(Cl)c2)C3=O)c(OC)n1	0.2	9.70
	2014	CHEMBL3653214	[C-]#[N+]c1ccc(C2c3c(nc(-c4cnc(N(C)C)nc4OC)n3C(C)C)C(=O)N2c2ccc(F)c(Cl)c2)cc1	0.2	9.70
	2014	CHEMBL3650060	COc1cc2c(cc1OC(C)C)C(c1ccc(Cl)cc1)N(C1CCC(N(CCCO)C(C)=O)CC1)C(=O)C2	720.0	6.14
	2014	CHEMBL3653263	COc1cn(C)c(=O)cc1-c1nc2c(n1C(C)C)C(c1ccc(Cl)cc1)N(c1cccc(Cl)c1F)C2=O	0.4	9.46
	2014	CHEMBL3656984	COc1ncc(-c2nc3c(n2C(C)C)[C@@H](c2ccc(C#N)cc2)N(c2cccc(Cl)c2F)C3=O)c(OC)n1	214.3	6.67
	2014	CHEMBL3653244	COc1ncc(-c2nc3c(n2C2CC2)C(c2ccc(Cl)cc2)N(c2ccc(F)c(Cl)c2)C3=O)c(OC)n1	3.6	8.44
	2015	CHEMBL3687231	CC(C)=CCn1nc2c(c1C(C)C)C(c1ccc(Cl)cc1C)N(c1cccc(Cl)c1F)C2=O	4.8	8.32
	2014	CHEMBL3653286	COc1ncc(CC#N)cc1-c1nc2c(n1C(C)C)C(c1ccc(Cl)cc1)N(c1cccc(Cl)c1F)C2=O	0.7	9.19
	2014	CHEMBL3650062	COc1cc2c(cc1OC(C)C)C(c1ccc(Cl)cc1)N(C1CCC(C(C)(C)O)CC1)C(=O)C2	55.0	7.26
	2014	CHEMBL3657030	COc1ccc(C(=O)NCCO)cc1-c1nc2c(n1C(C)C)[C@@H](c1ccc(Cl)cc1C)N(c1cc(Cl)ccc1C)C2=O	13.4	7.87
	2015	CHEMBL3683103	CC[C@@H](C)Oc1cc2c(cc1OC)CC(=O)N(c1ccc(N(C)C)cc1)C2c1ccc(Cl)cc1N(C)C	55505.0	5.26

	2014	CHEMBL3657051	COc1cnc(CC#N)cc1-c1nc2c(n1C(C)C)C(c1ccc(C#N)cc1)N(c1ccc(F)c(Cl)c1)C2=O	1.2	8.92
	2014	CHEMBL3657061	COc1cnc(CC#N)cc1-c1nc2c(n1C(C)C)[C@H](c1ccc(C#N)cc1)N(c1cc(Cl)ccc1C)C2=O	0.2	9.70
	2015	CHEMBL3687242	Cc1cc(Cl)ccc1C1c2c(nn(CC(=O)N3CCC(O)CC3)c2C(C)C)C(=O)N1c1cc(Cl)ccc1C	6.1	8.21
	2015	CHEMBL3691755	COc1ncc(-n2nc3c(c2C(C)C)C(c2ccc(C#N)cc2)N(c2c[nH]c(=O)c(Cl)c2)C3=O)c(OC)n1	0.8	9.11
	2015	CHEMBL3601403	COc1cc2c(cc1OC(C)C)[C@H](c1ccc(Cl)cc1)N(c1cnc(N(C)C[C@H]3CC[C@H](N4CCN(C)C(=O)C4)CC3)cn1)C(=O)C2	4.3	8.37
	2014	CHEMBL3653269	COc1ncc(-c2nc3c(n2C(C)C)C([C@H]2CC[C@H](C)CC2)N(c2cc(Cl)ccc2C)C3=O)c(OC)n1	0.6	9.21
	2014	CHEMBL3650058	CCCCN(C(C)=O)C1CCC(N2C(=O)Cc3cc(OC)c(OC(C)C)cc3C2c2ccc(Cl)cc2)CC1	257.0	6.59
	2015	CHEMBL3691714	COc1ccccc1-n1nc2c(c1C(C)C)C(c1ccc(Cl)cc1C)N(c1ccnc(Cl)c1)C2=O	7.2	8.14
	2015	CHEMBL3691778	COc1nc(N(C)C)ncc1-n1nc2c(c1C(C)C)[C@@H](c1ccc(C#N)cc1)N(c1cc(Cl)c[nH]c1=O)C2=O	86.1	7.07
	2015	CHEMBL3687255	Cc1cc(Cl)ccc1C1c2c(nn(CCN(C)C)c2C(C)C)C(=O)N1c1cccc(Cl)c1F	8.1	8.09
	2014	CHEMBL3650067	COc1cc2c(cc1OC(C)C)C(c1ccc(Cl)cc1)N(C1CCC(C(C)(O)C3CCOCC3)CC1)C(=O)C2	36.0	7.44
	2014	CHEMBL3653210	[C-]#[N+]c1ccc(C2c3c(nc(-c4cnc(NC)nc4OC)n3C(C)C)C(=O)N2c2cccc(Cl)c2F)cc1	0.3	9.51
	2014	CHEMBL3653258	CNC(=O)[C@H]1CC[C@H](N2C(=O)c3nc(-c4cnc(OC)nc4OC)n(C(C)C)c3C2c2ccc(Cl)cc2)CC1	5.6	8.25
	2015	CHEMBL3691775	COc1nc(N(C)C)ncc1-n1nc2c(c1C(C)C)[C@@H](c1ccc(C#N)cc1)N(c1cc(Cl)c(=O)n(C)c1)C2=O	22.7	7.64

	2014	CHEMBL3653193	COc1nc(N)ncc1-c1nc2c(n1C(C)C)C(c1ccc(Cl)cc1)N(c1cc(Cl)ccc1C)C2=O	0.3	9.57
	2015	CHEMBL3683168	COc1cc2c(cc1OC(C)C)[C@H](c1ccc(Cl)cc1)N(c1ccc(N(C)C(=O)[C@H]3CC[C@H](N(C)C)CC3)cc1)C(=O)C2	719.1	6.14
	2014	CHEMBL3653179	COc1ccc(C(=O)N(C)C)cc1-c1nc2c(n1C(C)C)C(c1ccc(Cl)cc1)N(c1cc(Cl)ccc1C)C2=O	0.1	9.89
	2015	CHEMBL3683108	CCOc1cc2c(cc1OCC)C(c1ccc(Cl)cc1)N(c1ccc(C)cc1OCCCN1CCOCC1)C(=O)C2	884.3	6.05
	2015	CHEMBL3683153	CC[C@@H](C)Oc1cc2c(cc1OC)CC(=O)N(c1ccc(C(C)N3CCC(NC(C)=O)CC3)cc1)C2c1ccc(Cl)cc1	111.9	6.95
	2014	CHEMBL3653237	COc1ccc(C(=O)N(C)C)cc1-c1nc2c(n1C(C)C)C(c1ccc(C#N)cc1)N(c1ccc(F)c(Cl)c1)C2=O	0.4	9.46
	2014	CHEMBL3653300	CNC(=O)Cc1cnc(OC)c(-c2nc3c(n2C(C)C)C(c2ccc(C#N)cc2)N(c2cccc(Cl)c2F)C3=O)c1	1.8	8.74
	2014	CHEMBL3653209	[C-]#[N+]c1ccc(C2c3c(nc(-c4cnc(N)nc4OC)n3C(C)C)C(=O)N2c2cccc(Cl)c2F)cc1	1.0	8.99
	2015	CHEMBL3683130	CC[C@@H](C)Oc1cc2c(cc1OC)CC(=O)N(c1ccc(CO)cc1)C2c1ccc(Cl)cc1	410.5	6.39
	2014	CHEMBL3657074	COc1nc(N)ncc1-c1nc2c(n1C(C)C)[C@H](c1ccc(Cl)cc1)N(c1cccc(Cl)c1F)C2=O	0.2	9.74
	2015	CHEMBL3691761	CNc1ncc(-n2nc3c(c2C(C)C)C(c2ccc(Cl)cc2)N(c2cc(Cl)c[nH]c2=O)C3=O)c(OC)n1	0.4	9.39
	2015	CHEMBL3691751	COc1ncc(-n2nc3c(c2C(C)C)C(c2ccc(Cl)cc2)N(c2c[nH]c(=O)c(Cl)c2)C3=O)c(OC)n1	0.2	9.75
	2014	CHEMBL3653171	COc1ccc(C(=O)N2CC(O)C2)cc1-c1nc2c(n1C(C)C)C(c1ccc(Cl)cc1C)N(c1cc(Cl)ccc1C)C2=O	0.2	9.74
	2015	CHEMBL3687284	CNS(=O)(=O)Cc1ccc(-n2nc3c(c2C(C)C)C(c2ccc(Cl)cc2C)N(c2cccc(Cl)c2F)C3=O)cc1	6.8	8.17

	2014	CHEMBL3657063	COc1cnc(CC#N)cc1-c1nc2c(n1C(C)C)[C@H](c1ccc(C#N)cc1)N(c1cccc(Cl)c1F)C2=O	0.3	9.57
	2015	CHEMBL3691766	COc1nc(N(C)C)ncc1-n1nc2c(c1C(C)C)[C@@H](c1ccc(C#N)cc1)N(c1cc(Cl)cn(C)c1=O)C2=O	86.6	7.06
	2015	CHEMBL3691763	COc1ncc(-n2nc3c(c2C(C)C)[C@@H](c2ccc(Cl)cc2)N(c2cc(Cl)c[nH]c2=O)C3=O)c(OC)n1	14.8	7.83
	2014	CHEMBL3653289	COc1cnc(CC#N)cc1-c1nc2c(n1C(C)C)C(c1ccc(Cl)cc1)N(c1cccc(Cl)c1F)C2=O	0.2	9.70
	2015	CHEMBL3683123	CC(C)N(CCN(C)C)c1ccc2c(c1)C(c1ccc(Cl)cc1)N(c1ccc(N(C)Cc3ccncc3)cc1)C(=O)C2	153.8	6.81
	2014	CHEMBL3653233	COc1ccccc1-c1nc2c(n1C(C)C)C(c1ccc(C#N)cc1)N(c1cc(Cl)ccc1C)C2=O	0.2	9.72
	2014	CHEMBL3653168	COc1ccc(C(=O)NCCO)cc1-c1nc2c(n1C(C)C)C(c1ccc(Cl)cc1C)N(c1cc(Cl)ccc1C)C2=O	0.2	9.82
	2015	CHEMBL3683174	CNC(=O)CN[C@H]1CC[C@H](CN(C)c2ccc(N3C(=O)Cc4cc(OC)c(OC(C)C)cc4[C@@H]3c3ccc(Cl)cc3)nc2)CC1	7.7	8.11
	2014	CHEMBL3653306	CNC(=O)Cc1ccc(OC)c(-c2nc3c(n2C(C)C)C(c2ccc(Cl)cc2)N(c2cc(Cl)ccc2C)C3=O)c1	0.2	9.77
	2014	CHEMBL3653247	COc1nc(N(C)C)ncc1-c1nc2c(n1C(C)C)C(c1ccc(Cl)cc1)N(c1cc(C)nn1C)C2=O	0.5	9.35
	2015	CHEMBL3683177	CCN1CN([C@H]2CC[C@H](CN(C)c3ccc(N4C(=O)Cc5cc(OC)c(OC(C)C)cc5[C@@H]4c4ccc(Cl)cc4)cn3)CC2)CC1=O	4.1	8.39
	2014	CHEMBL3657062	COc1cnc(CC#N)cc1-c1nc2c(n1C(C)C)[C@@H](c1ccc(C#N)cc1)N(c1cc(Cl)ccc1C)C2=O	57.7	7.24
	2014	CHEMBL3657003	COc1ncc(-c2nc3c(n2C(C)C)C(c2ccc(C#N)cc2C)N(c2cc(Cl)ccc2C)C3=O)c(OC)n1	0.3	9.48
	2015	CHEMBL3691712	Cc1cc(Cl)ccc1C1c2c(nn(C3CC3)c2C(C)C)C(=O)N1c1cccc(Cl)c1F	8.7	8.06

	2015	CHEMBL3691717	COc1ccncc1-n1nc2c(c1C(C)C)C(c1ccc(Cl)cc1)N(c1cccc(Cl)c1F)C2=O	6.9	8.16
	2014	CHEMBL3657100	COc1ncc(-c2nc3c(n2C(C)C)C(c2ccc(Cl)cc2)N(c2cc(C)nnc2OC)C3=O)c(OC)n1	5.9	8.23
	2014	CHEMBL3657123	COC[C@@H](C)n1c(-c2cnc(OC)nc2OC)nc2c1[C@@H](c1ccc(Cl)cc1)N(c1cc(Cl)c(=O)n(C)c1)C2=O	21.7	7.66
	2014	CHEMBL3657103	COc1nc(N(C)C)ncc1-c1nc2c(n1C(C)C)C(c1ccc(Cl)cc1F)N(c1cccc(Cl)c1F)C2=O	0.4	9.45
	2015	CHEMBL3691705	CCn1cc(N2C(=O)c3nn(-c4ccccc4OC)c(C(C)C)c3C2c2ccc(Cl)cc2)cc(Cl)c1=O	2.2	8.66
	2015	CHEMBL3691724	COc1ccc(CNC=O)cc1-n1nc2c(c1C(C)C)C(c1ccc(Cl)cc1C)N(c1cc(Cl)ccc1C)C2=O	0.2	9.77
	2015	CHEMBL3691726	COc1ccc(CN)cc1-n1nc2c(c1C(C)C)C(c1ccc(Cl)cc1C)N(c1cc(Cl)c(=O)n(C)c1)C2=O	0.6	9.26
	2014	CHEMBL3657011	COc1nc(N(C)C)ncc1-c1nc2c(n1C(C)C)C(c1ccc(Cl)cc1)N(c1cc(Cl)cn(C)c1=O)C2=O	0.2	9.80
	2015	CHEMBL3687289	COc1ccccc1-n1nc2c(c1C(C)C)C(c1ccc(F)cc1)N(c1cccc(Cl)c1F)C2=O	6.7	8.17
	2014	CHEMBL3657026	COc1ccc(C#N)cc1-c1nc2c(n1C(C)C)C(c1ccc(Cl)cc1)N(c1cccc(Cl)c1)C2=O	0.4	9.41
	2014	CHEMBL3653219	COc1cc(CN)ccc1-c1nc2c(n1C(C)C)C(c1ccc(Cl)cc1)N(c1cc(Cl)ccc1C)C2=O	0.2	9.77
	2014	CHEMBL3653229	COc1ccc(C(=O)N(C)C)cc1-c1nc2c(n1C(C)C)C(c1ccc(Cl)cc1)N(c1cccc(Cl)c1)C2=O	0.4	9.38
	2014	CHEMBL3653228	COc1ncc(-c2nc3c(n2C(C)C)C(c2ccc(Cl)cc2)N(c2cncc(Cl)c2)C3=O)c(OC)n1	1.0	8.99
	2015	CHEMBL3683131	CC[C@@H](C)Oc1cc2c(cc1OC)CC(=O)N(c1ccc(N(C)C(C)=O)cc1)C2c1ccc(Cl)cc1	607.2	6.22

	2015	CHEMBL3687271	Cc1cc(Cl)ccc1C1c2c(nn(CCN3CCCC3=O)c2C(C)C)C(=O)N1c1cc(Cl)ccc1C	1.7	8.77
	2015	CHEMBL3687301	Cc1cc(Cl)ccc1C1c2c(nn(CCC#N)c2C(C)C)C(=O)N1c1cccc(Cl)c1F	15.6	7.81
	2015	CHEMBL3914742	COc1cc2c(cc1OC(C)C)[C@H](c1ccc(Cl)cc1)N(c1ccc(N(C)CC3CCC(N4CCNC(=O)C4)CC3)cn1)C(=O)C2	4.7	8.33
	2014	CHEMBL3657113	COC[C@@H](C)n1c(-c2cnc(N)nc2OC)nc2c1C(c1ccc(Cl)cc1)N(c1ccc(F)c(Cl)c1)C2=O	0.4	9.43
	2014	CHEMBL3657088	COc1ncc(-c2nc3c(n2C(C)C)C(C)(c2ccc(Cl)cc2)N(c2cc(Cl)cn(C)c2=O)C3=O)c(OC)n1	0.7	9.15
	2014	CHEMBL3650052	COc1cc2c(cc1OC(C)C)C(c1ccc(Cl)cc1)N(C1CCC(NC(N)=O)CC1)C(=O)C2	4408.0	5.36
	2015	CHEMBL3691694	COc1ccccc1-n1nc2c(c1C(C)C)C(c1ccc(Cl)cn1)N(c1cc(Cl)ccc1C)C2=O	1.9	8.72
	2014	CHEMBL3653285	COc1ncc(CC#N)cc1-c1nc2c(n1C(C)C)C(c1ccc(C#N)cc1)N(c1cccc(Cl)c1F)C2=O	1.9	8.71
	2014	CHEMBL3650053	COc1cc2c(cc1OC(C)C)C(c1ccc(Cl)cc1)N(C1CCC(N(C)C(N)=O)CC1)C(=O)C2	372.0	6.43
	2014	CHEMBL3653174	COc1ccc(CO)cc1-c1nc2c(n1C(C)C)C(c1ccc(Cl)cc1C)N(c1cc(Cl)ccc1C)C2=O	0.1	9.85
	2015	CHEMBL3683161	COc1cc2c(cc1OC(C)C)[C@H](c1ccc(Cl)cc1)N(c1ccc(N(C)C[C@H]3CC[C@H](N4CC(=O)NCC4=O)CC3)cc1)C(=O)C2	6.3	8.20
	2014	CHEMBL3657022	COc1ccc(C(=O)N(C)C)cc1-c1nc2c(n1C(C)C)C(c1ccc(Cl)cc1)N(c1cncc(Cl)c1)C2=O	1.1	8.98
	2015	CHEMBL3691701	COc1ccccc1-n1nc2c(c1C(C)C)C(c1ccc(Cl)cc1C)N(c1cc(Cl)cc(NC(C)=O)c1)C2=O	2.9	8.54
	2015	CHEMBL3687293	COc1ccccc1-n1nc2c(c1C(C)C)C(c1ccc(Cl)cc1)N(c1cccc(Cl)c1F)C2=O	2.0	8.70

	2014	CHEMBL3657015	COc1ccc(C(=O)N(C)C)cc1-c1nc2c(n1C(C)C)C(c1ccc(Cl)cc1)N(c1cc(Cl)cn(C)c1=O)C2=O	0.3	9.54
	2014	CHEMBL3657119	COC[C@@H](C)n1c(-c2cnc(OC)nc2OC)nc2c1C(c1ccc(Cl)cc1)N(c1cc(Cl)c(=O)n(C)c1)C2=O	0.4	9.38
	2014	CHEMBL3657070	COc1ncc(-c2nc3c(n2C(C)C)C(c2ccc(Cl)cc2)N(c2cc(C)ccc2OC)C3=O)c(OC)n1	0.6	9.20
	2014	CHEMBL3657096	COc1ncc(-c2nc3c(n2C(C)C)C(c2ccc(Cl)cc2)N(c2cc(Cl)c(=O)n(C(F)F)c2)C3=O)c(OC)n1	0.4	9.40
	2014	CHEMBL3657041	COc1ccccc1-c1nc2c(n1C(C)C)C(c1ccc(Cl)cc1C)N(c1cc(Cl)c(=O)n(CCO)c1)C2=O	0.8	9.11
	2015	CHEMBL3691768	CNc1ncc(-n2nc3c(c2C(C)C)C(c2ccc(Cl)cc2)N(c2cc(Cl)c(=O)n(C)c2)C3=O)c(OC)n1	0.4	9.43
	2015	CHEMBL3691749	COc1ncc(-n2nc3c(c2C(C)C)[C@H](c2ccc(Cl)cc2C)N(c2cc(Cl)c(=O)n(C)c2)C3=O)c(OC)n1	0.2	9.62
	2014	CHEMBL3653288	COc1cnc(CC#N)cc1-c1nc2c(n1C(C)C)C(c1ccc(C#N)cc1)N(c1cc(Cl)ccc1C)C2=O	0.3	9.57
	2014	CHEMBL3657000	COc1nc(N(C)C)ncc1-c1nc2c(n1C(C)C)C(c1ccc(Cl)cc1)N(c1ccc(F)c(Cl)c1)C2=O	0.4	9.44
	2015	CHEMBL3683115	CCOc1cc2c(cc1O[C@H](C)CC)C(c1ccc(Cl)cc1)N(c1ccc(OC)cc1)C(=O)C2	245.8	6.61
	2014	CHEMBL3653191	COc1nc(N(C)C)ncc1-c1nc2c(n1C(C)C)C(c1ccc(Cl)cc1C)N(c1cc(Cl)ccc1C)C2=O	0.2	9.80
	2015	CHEMBL3687283	CC(C)c1c2c(nn1CCO)C(=O)N(c1cccc(Cl)c1F)C2c1ccc(Cl)cc1Cl	13.2	7.88
	2014	CHEMBL3653170	COc1ccc(C(=O)N2CCOCC2)cc1-c1nc2c(n1C(C)C)C(c1ccc(Cl)cc1C)N(c1cc(Cl)ccc1C)C2=O	0.2	9.74
	2014	CHEMBL3657087	COc1nc(N(C)C)ncc1-c1nc2c(n1C(C)C)C(c1ccc(Cl)c(F)c1)N(c1cc(Cl)cn(C)c1=O)C2=O	0.2	9.72

	2015	CHEMBL3604661	CC[C@@H](C)Oc1cc2c(cc1OC)CC(=O)N(c1ccc(N(C)C)cc1)C2c1ccc(Cl)cc1	115.9	6.94
	2014	CHEMBL3653204	COc1nc(N)ncc1-c1nc2c(n1C(C)C)C(c1ccc(Cl)cc1C)N(c1cc(Cl)ccc1C)C2=O	0.3	9.47
	2015	CHEMBL3691757	COc1ncc(-n2nc3c(c2C(C)C)C(c2ccc(C#N)cc2)N(c2cc(Cl)c(=O)n(C)c2)C3=O)c(OC)n1	1.3	8.89
	2015	CHEMBL3687250	C=CCn1nc2c(c1C(C)C)C(c1ccc(Cl)cc1C)N(c1cc(Cl)ccc1C)C2=O	1.4	8.85
	2015	CHEMBL3691790	COc1nc(N(C)C)ncc1-n1nc2c(c1C(C)C)C(c1ccc(Cl)c(F)c1)N(c1cc(Cl)cn(C)c1=O)C2=O	0.1	9.89
	2014	CHEMBL3650066	COc1cc2c(cc1OC(C)C)[C@@H](c1ccc(Cl)cc1)N(C1CCC(C(=O)C3CCOCC3)CC1)C(=O)C2	4546.0	5.34
	2014	CHEMBL3653307	COc1ccc(CC(=O)N(C)C)cc1-c1nc2c(n1C(C)C)C(c1ccc(Cl)cc1)N(c1cccc(Cl)c1F)C2=O	0.2	9.77
	2014	CHEMBL3653257	COc1ncc(-c2nc3c(n2C(C)C)[C@@H](c2ccc(Cl)cc2)N(c2cc(Cl)cn(C)c2=O)C3=O)c(OC)n1	299.7	6.52
	2015	CHEMBL3683170	COc1cc2c(cc1OC(C)C)[C@H](c1ccc(Cl)cc1)N(c1ccc(C(C)C(=O)N3CCC(N(C)C)CC3)cc1)C(=O)C2	98.3	7.01
	2014	CHEMBL3657102	COc1nc(N(C)C)ncc1-c1nc2c(n1C(C)C)C(c1ccc(Cl)cc1)N(c1cc(C)nnc1OC)C2=O	1.3	8.90
	2014	CHEMBL3657018	COc1nc(N)ncc1-c1nc2c(n1C(C)C)[C@H](c1ccc(Cl)cc1)N(c1cc(Cl)cn(C)c1=O)C2=O	0.7	9.17
	2014	CHEMBL3650078	CCCC(C)(O)C1CCC(N2C(=O)Cc3cc(OC)c(OC(C)C)cc3C2c2ccc(Cl)cc2)CC1	7.7	8.11
	2014	CHEMBL3657001	COc1ccc(C(=O)N(C)C)cc1-c1nc2c(n1C(C)C)C(c1ccc(C#N)cc1C)N(c1cc(Cl)ccc1C)C2=O	0.3	9.57
	2014	CHEMBL3657019	COc1nc(N)ncc1-c1nc2c(n1C(C)C)[C@@H](c1ccc(Cl)cc1)N(c1cc(Cl)cn(C)c1=O)C2=O	68.6	7.16

	2015	CHEMBL3683118	CC[C@@H](C)Oc1cc2c(cc1OC)CC(=O)N(c1ccc(OC)cc1)C2c1ccc(Cl)cc1	134.6	6.87
	2014	CHEMBL3657013	COc1ncc(-c2nc3c(n2C(C)C)C(c2ccc(Cl)cc2)N(c2ccc(F)c(Cl)c2)C3=O)c(OC)n1	0.2	9.70
	2015	CHEMBL3691777	COc1nc(N(C)C)ncc1-n1nc2c(c1C(C)C)[C@@H](c1ccc(Cl)cc1)N(c1cc(Cl)c[nH]c1=O)C2=O	24.4	7.61
	2015	CHEMBL3691784	[C-]#[N+]c1ccc(C2c3c(nn(-c4cnc(OC)nc4OC)c3C(C)C)C(=O)N2c2cc(Cl)cn(C)c2=O)cc1F	1.3	8.89
	2015	CHEMBL3691707	COc1ccccc1-n1nc2c(c1C(C)C)C(c1ccc(Cl)cc1)N(c1cc(Cl)c(=O)n(CCO)c1)C2=O	10.7	7.97
	2015	CHEMBL3683117	CCCOc1cc2c(cc1OC)CC(=O)N(c1ccc(OC)cc1)C2c1ccc(Cl)cc1	424.2	6.37
	2014	CHEMBL3653218	COc1nc(N2CC(O)C2)ncc1-c1nc2c(n1C(C)C)C(c1ccc(Cl)cc1C)N(c1cc(Cl)ccc1C)C2=O	0.2	9.68
	2014	CHEMBL3653291	COc1cnc(C(N)=O)nc1-c1nc2c(n1C(C)C)C(c1ccc(Cl)cc1)N(c1cc(Cl)ccc1C)C2=O	0.7	9.18
	2015	CHEMBL3687312	COC(=O)CCC1(c2ccc(Cl)cc2)c2c(nn(-c3ccccc3OC)c2C(C)C)C(=O)N1c1ccc(F)c(Cl)c1	0.5	9.30
	2015	CHEMBL3683178	COc1cc2c(cc1OC(C)C)[C@H](c1ccc(Cl)cc1)N(c1ccc(N(C)C[C@H]3CC[C@H](N4CC(=O)N(C(C)C)C4)CC3)cn1)C(=O)C2	1.2	8.92
	2014	CHEMBL3657007	COc1nc(N)ncc1C1=NC2C(=O)N(c3cccc(Cl)c3F)C(c3ccc(Cl)cc3)C2N1C(C)C	0.4	9.38
	2014	CHEMBL3650075	CCCCC(C)(O)C1CCC(N2C(=O)Cc3cc(OC)c(OC(C)C)cc3C2c2ccc(Cl)cc2)CC1	6.3	8.20
	2014	CHEMBL3657071	COc1ncc(-c2nc3c(n2C(C)C)C(c2ccc(Cl)cc2)N(c2cc(C)nc(C)n2)C3=O)c(OC)n1	92.7	7.03
	2014	CHEMBL3657077	COc1cn(C)c(=O)cc1-c1nc2c(n1C(C)C)C(c1ccc(Cl)cc1)N(c1cc(C)ccc1OC)C2=O	1.2	8.94

	2014	CHEMBL3653235	COc1ncc(-c2nc3c(n2C(C)C)C(c2ccc(C#N)cc2)N(c2cccc(Cl)c2F)C3=O)c(OC)n1	0.4	9.44
	2014	CHEMBL3653298	COc1ncc(CC(=O)O)cc1-c1nc2c(n1C(C)C)C(c1ccc(C#N)cc1)N(c1cc(Cl)ccc1C)C2=O	2.4	8.62
	2015	CHEMBL3601402	COc1cc2c(cc1OC(C)C)[C@H](c1ccc(Cl)cc1)N(c1ccc(N(C)C[C@H]3CC[C@H](N4CCN(C)C(=O)C4)CC3)cn1)C(=O)C2	7.0	8.15
	2015	CHEMBL3691795	COc1nc(N(C)C)ncc1-n1nc2c(c1C(C)C)C(c1ccc(Cl)cc1)N(c1cc(C)cn(C)c1=O)C2=O	0.5	9.28
	2014	CHEMBL3650051	COc1cc2c(cc1OC(C)C)C(c1ccc(Cl)cc1)N(C1CCC(N(C)CCO)CC1)C(=O)C2	6429.0	5.19
	2015	CHEMBL3687234	Cc1cc(Cl)ccc1C1c2c(nn(CCN3C(=O)c4ccccc4C3=O)c2C(C)C)C(=O)N1c1cccc(Cl)c1F	8.9	8.05
	2015	CHEMBL3683120	CC(C)Nc1ccc2c(c1)C(c1ccc(Cl)cc1)N(c1ccc(N(C)Cc3ccncc3)cc1)C(=O)C2	81.8	7.09
	2014	CHEMBL3653199	[C-]#[N+]c1ccc(C2c3c(nc(-c4cnc(N(C)C)nc4OC)n3C(C)C)C(=O)N2c2cc(Cl)c(=O)n(C)c2)cc1	0.3	9.57
	2014	CHEMBL3653182	COc1cc(C#N)ccc1-c1nc2c(n1C(C)C)C(c1ccc(Cl)cc1)N(c1cc(Cl)ccc1C)C2=O	0.3	9.54
	2015	CHEMBL3687292	COc1ccccc1-n1nc2c(c1C(C)C)C(c1ccc(C)cc1)N(c1cccc(Cl)c1F)C2=O	1.8	8.74
	2015	CHEMBL3683113	COc1ccc(N2C(=O)Cc3cc(OCCN4CCOCC4)c(OC)cc3C2c2ccc(Cl)cc2)cc1	10288.5	4.99
	2014	CHEMBL3657045	CC(C)n1c(-c2ccnc(N)c2)nc2c1C(c1ccc(Cl)cc1)N(c1cccc(Cl)c1F)C2=O	6.6	8.18
	2015	CHEMBL3691782	COc1ncc(-n2nc3c(c2C(C)C)C(c2ccc(Cl)c(F)c2)N(c2cc(Cl)c(=O)n(C)c2)C3=O)c(OC)n1	0.4	9.42
	2014	CHEMBL3656980	COc1ccc(CC(=O)O)cc1-c1nc2c(n1C(C)C)C(c1ccc(Cl)cc1)N(c1cccc(Cl)c1F)C2=O	0.3	9.49

	2015	CHEMBL3683109	CCOc1cc2c(cc1OCC)C(c1ccc(Cl)cc1)N(c1ccc(C)cc1OCC(=O)O)C(=O)C2	1677.5	5.78
	2014	CHEMBL3653264	COc1cn(C)c(=O)cc1-c1nc2c(n1C(C)C)C(c1ccc(Cl)cc1)N(c1cc(Cl)ccc1C)C2=O	0.2	9.64
	2015	CHEMBL3687246	Cc1cc(Cl)ccc1C1c2c(nn(CC(=O)N3CCNC(=O)C3)c2C(C)C)C(=O)N1c1cc(Cl)ccc1C	4.4	8.36
	2015	CHEMBL3683171	COc1cc2c(cc1OC(C)C)[C@H](c1ccc(Cl)cc1)N(c1ccc(N(C)C[C@H]3CC[C@H](N4CCNC(=O)C4)CC3)cc1OC)C(=O)C2	2.0	8.70
	2014	CHEMBL3653251	COc1ncc(-c2nc3c(n2C(C)C)C(c2ccc(Cl)cc2)N(c2ccc(C(N)=O)cc2C)C3=O)c(OC)n1	1.3	8.88
	2014	CHEMBL3653194	COc1nc(N(C)CCO)ncc1-c1nc2c(n1C(C)C)C(c1ccc(Cl)cc1)N(c1cc(Cl)ccc1C)C2=O	0.2	9.72
	2014	CHEMBL3657118	COC[C@@H](C)n1c(-c2cnc(OC)nc2OC)nc2c1[C@@H](c1ccc(Cl)cc1)N(c1ccc(F)c(Cl)c1)C2=O	43.7	7.36
	2015	CHEMBL3691780	COc1ncc(-n2nc3c(c2C(C)C)C(c2ccc(Cl)cc2F)N(c2cc(Cl)c(=O)n(C)c2)C3=O)c(OC)n1	1.1	8.96
	2015	CHEMBL3691706	COc1ccccc1-n1nc2c(c1C(C)C)C(c1ccc(Cl)cc1C)N(c1cc(Cl)c(=O)n(C)c1)C2=O	0.4	9.41
	2014	CHEMBL3650069	COc1cc2c(cc1OC(C)C)[C@H](c1ccc(Cl)cc1)N(C1CCC([C@](C)(O)C3CCOCC3)CC1)C(=O)C2	158.0	6.80
	2014	CHEMBL3657072	COc1nc(N)ncc1-c1nc2c(n1C(C)C)[C@H](c1ccc(Cl)cc1)N(c1ccc(F)c(Cl)c1)C2=O	0.2	9.70
	2014	CHEMBL3650072	COc1cc2c(cc1OC(C)C)C(c1ccc(Cl)cc1)N(C1CCC(C(C)(O)c3cccc(F)c3)CC1)C(=O)C2	61.0	7.21
	2014	CHEMBL3650064	COc1cc2c(cc1OC(C)C)C(c1ccc(Cl)cc1)N(C1CCC(C(=O)C3CCOCC3)CC1)C(=O)C2	610.0	6.21
	2015	CHEMBL3683137	CC[C@@H](C)Oc1cc2c(cc1OC)CC(=O)N(c1ccc(N(C)CC3CCN(C(=O)N(C)C)CC3)cc1)C2c1ccc(Cl)cc1	12.3	7.91

	2015	CHEMBL3639860	Cc1cc(Cl)ccc1C1c2c(nn(Cc3cc(=O)[nH]c4ccccc34)c2C(C)C)C(=O)N1c1cccc(Cl)c1F	5.3	8.28
	2015	CHEMBL3691772	COc1ncc(-n2nc3c(c2C(C)C)[C@H](c2ccc(Cl)cc2)N(c2cc(Cl)c(=O)n(C)c2)C3=O)c(OC)n1	0.2	9.80
	2014	CHEMBL3653213	[C-]#[N+]c1ccc(C2c3c(nc(-c4cnc(NC)nc4OC)n3C(C)C)C(=O)N2c2ccc(F)c(Cl)c2)cc1	0.3	9.55
	2014	CHEMBL3657032	COc1cc(C(=O)NCCO)ccc1-c1nc2c(n1C(C)C)C(c1ccc(Cl)cc1C)N(c1cc(Cl)ccc1C)C2=O	0.3	9.60
	2015	CHEMBL3691713	Cc1cc(Cl)ccc1C1c2c(nn(C3CCC(O)CC3)c2C(C)C)C(=O)N1c1cccc(Cl)c1F	12.9	7.89
	2015	CHEMBL3687264	Cc1cc(Cl)ccc1C1c2c(nn(CCC(C)C)c2C2CC2)C(=O)N1c1cccc(Cl)c1F	12.0	7.92
	2014	CHEMBL3653254	COc1ncc(-c2nc3c(n2C(C)C)C(c2ccc(Cl)cc2)N([C@H]2CC[C@H](NC(C)=O)CC2)C3=O)c(OC)n1	11.8	7.93
	2014	CHEMBL3657004	COc1ccc(C(=O)N(C)C)cc1-c1nc2c(n1C(C)C)[C@@H](c1ccc(Cl)cc1)N(c1cc(Cl)ccc1C)C2=O	70.5	7.15
	2014	CHEMBL3650077	COc1cc2c(cc1OC(C)C)C(c1ccc(Cl)cc1)N(C1CCC(C(C)(O)c3ccoc3)CC1)C(=O)C2	27.6	7.56
	2015	CHEMBL3687265	Cc1cc(Cl)ccc1C1c2c(nn(CCCO)c2C(C)C)C(=O)N1c1cccc(Cl)c1F	6.0	8.22
	2015	CHEMBL3687297	COc1ccccc1-n1nc2c(c1C(C)C)C(CC(=O)N1CCOCC1)(c1ccc(Cl)cc1)N(c1cccc(Cl)c1F)C2=O	7.1	8.15
	2014	CHEMBL3653175	COc1ccc(CO)cc1-c1nc2c(n1C(C)C)C(c1ccc(Cl)cc1)N(c1cc(Cl)ccc1C)C2=O	0.2	9.82
	2014	CHEMBL3657115	COc1ncc(-c2nc3c(n2[C@H](C)CO)[C@@H](c2ccc(Cl)cc2)N(c2cccc(Cl)c2F)C3=O)c(OC)n1	41.0	7.39
	2015	CHEMBL3687240	CC(C)c1c2c(nn1Cc1ncc[nH]1)C(=O)N(c1cccc(Cl)c1F)C2c1ccc(Cl)cc1	33.9	7.47

	2015	CHEMBL3691696	COc1ccccc1-n1nc2c(c1C(C)C)C(CCO)(c1ccc(Cl)cc1)N(c1ccc(F)c(Cl)c1)C2=O	1.3	8.89
	2014	CHEMBL3653253	COc1ncc(-c2nc3c(n2C(C)C)C(c2ccc(Cl)cc2)N([C@H]2CC[C@H](C(=O)O)CC2)C3=O)c(OC)n1	15.1	7.82
	2014	CHEMBL3653215	COc1nc(N2CC(C)(O)C2)ncc1-c1nc2c(n1C(C)C)C(c1ccc(Cl)cc1)N(c1cc(Cl)ccc1C)C2=O	0.2	9.70
	2015	CHEMBL3683112	CC[C@@H](C)Oc1cc2c(cc1OC)N(C)C(=O)N(c1ccc(N(C)C)cc1)C2c1ccc(Cl)cc1	624.7	6.20
	2014	CHEMBL3657122	COC[C@@H](C)n1c(-c2cnc(OC)nc2OC)nc2c1[C@H](c1ccc(Cl)cc1)N(c1cc(Cl)c(=O)n(C)c1)C2=O	0.1	9.87
	2015	CHEMBL3683142	CC[C@@H](C)Oc1cc2c(cc1OC)CC(=O)N(c1ccc(C(C)O)cc1)C2c1ccc(Cl)cc1	72.4	7.14
	2014	CHEMBL3653184	COc1cc(-c2nc3c(n2C(C)C)C(c2ccc(Cl)cc2)N(c2cc(Cl)ccc2C)C3=O)c(OC)nn1	0.6	9.21
	2015	CHEMBL3683162	COc1cc2c(cc1OC(C)C)[C@H](c1ccc(Cl)cc1)N(c1ccc(N(C)C[C@H]3CC[C@H](N(CC(N)=O)CC(N)=O)CC3)cc1)C(=O)C2	1.8	8.74
	2014	CHEMBL3657120	COC[C@@H](C)n1c(-c2cnc(OC)nc2OC)nc2c1C(c1ccc(Cl)cc1)N(c1cc(Cl)cn(C)c1=O)C2=O	0.8	9.09
	2014	CHEMBL3657049	COc1cnc(CC#N)cc1-c1nc2c(n1C(C)C)C(c1ccc(Cl)cc1C)N(c1cc(Cl)cn(C)c1=O)C2=O	1.1	8.96
	2014	CHEMBL3650070	COc1cc2c(cc1OC(C)C)C(c1ccc(Cl)cc1)N(C1CCC(C(O)c3cccc(F)c3)CC1)C(=O)C2	72.0	7.14
	2014	CHEMBL3656977	COc1nc(N)ncc1-c1nc2c(n1C(C)C)C(c1ccc(Cl)cc1)N(c1cc(Cl)cn(C)c1=O)C2=O	1.4	8.87
	2015	CHEMBL3691742	COc1ncc(-n2nc3c(c2C(C)C)C(c2ccc(Cl)cc2C)N(c2cc(Cl)cn(C)c2=O)C3=O)c(OC)n1	0.3	9.46
	2014	CHEMBL3653299	CNC(=O)Cc1cnc(OC)c(-c2nc3c(n2C(C)C)C(c2ccc(Cl)cc2)N(c2cccc(Cl)c2F)C3=O)c1	0.7	9.18

	2014	CHEMBL3650056	CCCN(C(C)=O)C1CCC(N2C(=O)Cc3cc(OC)c(OC(C)C)cc3C2c2ccc(Cl)cc2)CC1	79.0	7.10
	2015	CHEMBL3683181	COc1cc2c(cc1OC(C)C)[C@H](c1ccc(Cl)cc1)N(c1ccc([C@H](C)NC(C)=O)cc1)C(=O)C2	521.0	6.28
	2015	CHEMBL3683166	CNC(=O)[C@H]1CCCN1[C@@H](C)c1ccc(N2C(=O)Cc3cc(OC)c(OC(C)C)cc3[C@@H]2c2ccc(Cl)cc2)cc1	14.2	7.85
	2015	CHEMBL3687277	CC(=O)N1CCCC1Cn1nc2c(c1C(C)C)C(c1ccc(Cl)cc1C)N(c1cc(Cl)ccc1C)C2=O	1.5	8.82
	2015	CHEMBL3683159	COC(=O)CN(C)[C@H]1CC[C@H](CN(C)c2ccc(N3C(=O)Cc4cc(OC)c(OC(C)C)cc4[C@@H]3c3ccc(Cl)cc3)cc2)CC1	2.9	8.54
	2015	CHEMBL3683146	CC[C@@H](C)Oc1cc2c(cc1OC)CC(=O)N(c1ccc(C(C)N(C(C)=O)C3CCNCC3)cc1)C2c1ccc(Cl)cc1	114.2	6.94
	2015	CHEMBL3691774	COc1nc(N(C)C)ncc1-n1nc2c(c1C(C)C)[C@H](c1ccc(C#N)cc1)N(c1cc(Cl)c(=O)n(C)c1)C2=O	0.1	9.92
	2015	CHEMBL3691723	COc1ccc(CNC(C)=O)cc1-n1nc2c(c1C(C)C)C(c1ccc(Cl)cc1C)N(c1cc(Cl)ccc1C)C2=O	0.2	9.71
	2014	CHEMBL3657069	COc1ncc(-c2nc3c(n2C(C)C)C(c2ccc(Cl)cc2)N(c2ccc(F)c(C)c2)C3=O)c(OC)n1	0.9	9.07
	2015	CHEMBL3691693	COc1ccccc1-n1nc2c(c1C(C)C)C(c1ccc(Cl)cn1)N(c1ccc(F)c(Cl)c1)C2=O	6.2	8.21
	2014	CHEMBL3653241	COc1ncc(-c2nc3c(n2C2CCC2)C(c2ccc(Cl)cc2)N(c2ccc(F)c(Cl)c2)C3=O)c(OC)n1	11.8	7.93
	2015	CHEMBL3687248	CC(=O)N1CCC(Cn2nc3c(c2C(C)C)C(c2ccc(Cl)cc2C)N(c2cc(Cl)ccc2C)C3=O)CC1	2.4	8.62
	2015	CHEMBL3691750	COc1ncc(-n2nc3c(c2C(C)C)[C@@H](c2ccc(Cl)cc2C)N(c2cc(Cl)c(=O)n(C)c2)C3=O)c(OC)n1	111.3	6.95
	2014	CHEMBL3657054	COc1cn(C)c(=O)cc1-c1nc2c(n1C(C)C)C(c1ccc(Cl)cc1)N(c1cc(C)nc(C)n1)C2=O	142.5	6.85

	2015	CHEMBL3691730	COc1cnc(C#N)cc1-n1nc2c(c1C(C)C)C(c1ccc(Cl)cc1C)N(c1cc(Cl)ccc1C)C2=O	0.3	9.57
	2015	CHEMBL3687313	CCOC(=O)C1(c2ccc(Cl)cc2)c2c(nn(-c3ccccc3OC)c2C(C)C)C(=O)N1c1ccc(F)c(Cl)c1	5.2	8.28
	2015	CHEMBL3601397	COc1cc2c(cc1OC(C)C)[C@H](c1ccc(Cl)cc1)N(c1ccc(N(C)C[C@H]3CC[C@H](N4CCNC(=O)C4)CC3)cc1)C(=O)C2	1.6	8.80
	2014	CHEMBL3656976	COc1nc(N(C)C)ncc1-c1nc2c(n1C(C)C)C(c1ccc(Cl)cc1)N(c1cc(Cl)cnc1O)C2=O	0.1	9.85
	2015	CHEMBL3691732	[C-]#[N+]c1ccc(C2c3c(nn(-c4cc(C#N)ccc4OC)c3C(C)C)C(=O)N2c2cc(Cl)ccc2C)cc1	0.2	9.62
	2014	CHEMBL3657075	COc1nc(N)ncc1-c1nc2c(n1C(C)C)[C@@H](c1ccc(Cl)cc1)N(c1cccc(Cl)c1F)C2=O	505.7	6.30
	2014	CHEMBL3653222	COc1nc(N(C)C)ncc1-c1nc2c(n1C(C)C)C(c1ccc(Cl)cc1)N(c1cc(Cl)c(=O)n(C)c1)C2=O	0.2	9.64
	2014	CHEMBL3657079	COc1nc(N(C)C)ncc1-c1nc2c(n1C(C)C)C(c1ccc(C#N)c(F)c1)N(c1cc(Cl)cn(C)c1=O)C2=O	0.4	9.46
	2014	CHEMBL3657076	COc1cn(C)c(=O)cc1-c1nc2c(n1C(C)C)C(c1ccc(Cl)cc1)N(c1ccc(F)c(C)c1)C2=O	1.2	8.93
	2014	CHEMBL3653305	COc1ncc(-c2nc3c(n2C(C)C)C(C)(c2ccc(Cl)cc2C)N(c2cc(Cl)ccc2C)C3=O)c(OC)n1	0.4	9.42
	2015	CHEMBL3683127	CC[C@@H](C)Oc1cc2c(cc1OC)CC(=O)N(c1ccc(-c3cn[nH]c3)cc1)C2c1ccc(Cl)cc1	1383.0	5.86
	2014	CHEMBL3657025	COc1ncc(-c2nc3c(n2C(C)C)C(c2ccc(Cl)cc2)N(c2cccc(Cl)c2)C3=O)c(OC)n1	0.6	9.20
	2015	CHEMBL3691759	CNc1ncc(-n2nc3c(c2C(C)C)C(c2ccc(Cl)cc2)N(c2cc(Cl)cn(C)c2=O)C3=O)c(OC)n1	0.3	9.50
	2014	CHEMBL3653234	Cc1ccc(Cl)cc1N1C(=O)c2nc(-c3ccccc3O)n(C(C)C)c2C1c1ccc(C#N)cc1	2.1	8.68

	2015	CHEMBL3687253	Cc1cc(Cl)ccc1C1c2c(nn(CC3(O)CCN(C(=O)NC(C)C)CC3)c2C(C)C)C(=O)N1c1cc(Cl)ccc1C	2.2	8.66
	2015	CHEMBL3683133	CC[C@@H](C)Oc1cc2c(cc1OC)CC(=O)N(c1ccc(N(C)CC3CCCNC3)cc1)C2c1ccc(Cl)cc1	4.7	8.33
	2014	CHEMBL3653266	COc1cnc(N(C)CCO)nc1-c1nc2c(n1C(C)C)C(c1ccc(Cl)cc1)N(c1ccc(F)c(Cl)c1)C2=O	0.6	9.25
	2015	CHEMBL3691731	COc1cnc(C#N)cc1-n1nc2c(c1C(C)C)C(c1ccc(Cl)cc1C)N(c1cc(Cl)c(=O)n(C)c1)C2=O	0.8	9.12
	2015	CHEMBL3683180	COc1cc2c(cc1OC(C)C)[C@H](c1ccc(Cl)cc1)N(c1ccc([C@H](C)N3CCNCC3=O)cc1)C(=O)C2	7.1	8.15
	2014	CHEMBL3653262	COc1cnc(CC#N)cc1-c1nc2c(n1C(C)C)C(c1ccc(Cl)cc1)N(c1cc(Cl)cn(C)c1=O)C2=O	1.1	8.96
	2014	CHEMBL3656999	COc1nc(N(C)C)ncc1-c1nc2c(n1C(C)C)C(c1ccc(Cl)cc1)N(c1cccc(Cl)c1F)C2=O	0.3	9.51
	2015	CHEMBL3687304	Cc1cc(Cl)ccc1C1c2c(nn(CCO)c2C2CCC2)C(=O)N1c1cccc(Cl)c1F	5.6	8.25
	2014	CHEMBL3653301	COc1ncc(-c2nc3c(n2[C@@H](C)CO)C(c2ccc(Cl)cc2)N(c2cc(Cl)ccc2C)C3=O)c(OC)n1	1.7	8.76
	2015	CHEMBL3687296	Cc1cc(Cl)ccc1C1c2c(nn(C3CCOCC3)c2C(C)C)C(=O)N1c1cccc(Cl)c1F	18.0	7.74
	2014	CHEMBL3650050	COc1cc2c(cc1OC(C)C)C(c1ccc(Cl)cc1)N(C1CCC(NC(C)=O)CC1)C(=O)C2	969.0	6.01
	2015	CHEMBL3691720	COc1ccccc1-n1nc2c(c1C(C)C)C(c1ccc(Cl)cc1)N(c1cc(Cl)ccc1OCc1nn[nH]n1)C2=O	0.2	9.74
	2014	CHEMBL3657042	COCCn1cc(N2C(=O)c3nc(-c4ccccc4OC)n(C(C)C)c3C2c2ccc(Cl)cc2C)cc(Cl)c1=O	0.6	9.25
	2015	CHEMBL3691708	COCCn1cc(N2C(=O)c3nn(-c4ccccc4OC)c(C(C)C)c3C2c2ccc(Cl)cc2C)cc(Cl)c1=O	1.1	8.96

	2015	CHEMBL3687276	Cc1cc(Cl)ccc1C1c2c(nn(CC3CCCN3C(=O)N(C)C)c2C(C)C)C(=O)N1c1cc(Cl)ccc1C	1.6	8.880
	2014	CHEMBL3656979	COc1nc(N(C)C)ncc1-c1nc2c(n1C(C)C)[C@@H](c1ccc(C#N)cc1)N(c1cc(Cl)cn(C)c1=O)C2=O	153.2	6.81
	2015	CHEMBL3683179	COc1cc2c(cc1OC(C)C)[C@H](c1ccc(Cl)cc1)N(c1cnc(N(C)C[C@H]3CC[C@H](N4CC(=O)N(C)C4)CC3)cn1)C(=O)C2	4.3	8.37
	2015	CHEMBL3687267	Cc1cc(Cl)ccc1C1c2c(nn(CC3CCCO3)c2C(C)C)C(=O)N1c1cc(Cl)ccc1C	2.5	8.60
	2014	CHEMBL3656983	COC(=O)Cc1ccc(OC)c(-c2nc3c(n2C(C)C)C(c2ccc(Cl)cc2)N(c2cccc(Cl)c2F)C3=O)c1	0.3	9.49
	2014	CHEMBL3653239	COc1ccccc1-c1nc2c(n1C(C)C)C(c1ccc(Cl)cc1)N(C1CCC(=O)N(C)C1)C2=O	1.5	8.83
	2014	CHEMBL3657097	CCn1cc(Cl)cc(N2C(=O)c3nc(-c4cnc(OC)nc4OC)n(C(C)C)c3C2c2ccc(Cl)cc2)c1=O	0.3	9.49
	2014	CHEMBL3650047	COc1cc2c(cc1OC(C)C)C(c1ccc(Cl)cc1)N(C1CCCCC1)C(=O)C2	894.0	6.05
	2015	CHEMBL3683135	CC[C@@H](C)Oc1cc2c(cc1OC)CC(=O)N(c1ccc(N(C)CC3CCN(C(C)=O)CC3)cc1)C2c1ccc(Cl)cc1	6.0	8.22
	2014	CHEMBL3653280	COc1nc(N(C)C)ncc1-c1nc2c(n1C(C)C)C(c1ccc(Cl)cc1)N(c1cnc(O)c(Cl)c1)C2=O	0.1	9.85
	2014	CHEMBL3653250	COc1nc(N(C)C)ncc1-c1nc2c(n1C(C)C)C(c1ccc(Cl)cc1)N(c1ccc(C(N)=O)cc1C)C2=O	0.3	9.49
	2015	CHEMBL3691722	COc1ccc(CN)cc1-n1nc2c(c1C(C)C)C(c1ccc(Cl)cc1C)N(c1cc(Cl)ccc1C)C2=O	1.2	8.92
	2014	CHEMBL3653178	COc1cc(CO)ccc1-c1nc2c(n1C(C)C)C(c1ccc(Cl)cc1C)N(c1cc(Cl)ccc1C)C2=O	0.1	9.89
	2014	CHEMBL3653236	COc1ccccc1-c1nc2c(n1C(C)C)C(c1ccc(C#N)cc1)N(c1ccc(F)c(Cl)c1)C2=O	0.3	9.57

	2015	CHEMBL3683173	COc1cc2c(cc1OC(C)C)[C@H](c1ccc(Cl)cc1)N(c1ccc(N(C)C[C@H]3CC[C@H](N4CCN(C(C)=O)CC4)CC3)cc1)C(=O)C2	1.4	8.85
	2014	CHEMBL3653309	CNC(=O)Cc1ccc(OC)c(-c2nc3c(n2C(C)C)C(c2ccc(Cl)cc2)N(c2cccc(Cl)c2F)C3=O)c1	0.3	9.57
	2015	CHEMBL3687261	COCCn1nc2c(c1C(C)C)C(c1ccc(Cl)cc1C)N(c1cc(Cl)ccc1C)C2=O	4.4	8.36
	2015	CHEMBL3691741	COc1ncc(-n2nc3c(c2C(C)C)C(c2ccc(Cl)cc2)N(c2cc(Cl)cn(C)c2=O)C3=O)c(OC)n1	0.5	9.28
	2015	CHEMBL3687228	Cc1cc(Cl)ccc1C1c2c(nn(CCc3c[nH]cn3)c2C(C)C)C(=O)N1c1cccc(Cl)c1F	4.0	8.40
	2015	CHEMBL3683154	CC[C@@H](C)Oc1cc2c(cc1OC)CC(=O)N(c1ccc(C(C)N3CCN(C(C)=O)CC3)cc1)C2c1ccc(Cl)cc1	15.2	7.82
	2015	CHEMBL3683145	CC[C@@H](C)Oc1cc2c(cc1OC)CC(=O)N(c1ccc(C(C)NC3CCNCC3)cc1)C2c1ccc(Cl)cc1	42.6	7.37
	2015	CHEMBL3691765	CNc1ncc(-n2nc3c(c2C(C)C)C(c2ccc(C#N)cc2)N(c2cc(Cl)cn(C)c2=O)C3=O)c(OC)n1	1.2	8.90
	2014	CHEMBL3653302	CCOC(=O)Cc1cnc(OC)c(-c2nc3c(n2C(C)C)C(c2ccc(C#N)cc2)N(c2cccc(Cl)c2F)C3=O)c1	2.2	8.66
	2014	CHEMBL3653248	COc1ncc(-c2nc3c(n2C(C)C)C(c2ccc(Cl)cc2)N(c2cc(C)nn2C)C3=O)c(OC)n1	1.6	8.79
	2015	CHEMBL3683119	COc1cc2c(cc1OC(C)C)C(c1ccc(Cl)cc1)N(c1ccc(N(C)C)cc1)C(=O)C2	115.3	6.94
	2014	CHEMBL3657065	COC[C@@H](C)n1c(-c2cnc(OC)nc2OC)nc2c1C(c1ccc(C#N)cc1)N(c1cccc(Cl)c1F)C2=O	0.3	9.55
	2015	CHEMBL3691754	COc1nc(N(C)C)ncc1-n1nc2c(c1C(C)C)C(c1ccc(C#N)cc1)N(c1c[nH]c(=O)c(Cl)c1)C2=O	0.2	9.65
	2014	CHEMBL3657017	CNc1ncc(-c2nc3c(n2C(C)C)C(c2ccc(Cl)cc2)N(c2cc(Cl)cn(C)c2=O)C3=O)c(OC)n1	0.2	9.72

	2015	CHEMBL3691776	COc1nc(N(C)C)ncc1-n1nc2c(c1C(C)C)[C@H](c1ccc(Cl)cc1)N(c1cc(Cl)c[nH]c1=O)C2=O	0.6	9.22
	2015	CHEMBL3691740	COc1ccccc1-n1nc2c(c1C(C)C)[C@@H](c1ccc(Cl)cc1C)N(c1cc(Cl)ccc1OCCN(C)C)C2=O	3.9	8.41
	2014	CHEMBL3653195	COc1nc(NCCO)ncc1-c1nc2c(n1C(C)C)C(c1ccc(Cl)cc1)N(c1cc(Cl)ccc1C)C2=O	0.2	9.77
	2014	CHEMBL3657056	COc1nc(N(C)C)ncc1-c1nc2c(n1C(C)C)C(c1ccc(Cl)cn1)N(c1cc(Cl)ccc1C)C2=O	0.1	9.92
	2014	CHEMBL3653202	CNc1ncc(-c2nc3c(n2C(C)C)C(c2ccc(Cl)cc2C)N(c2cc(Cl)ccc2C)C3=O)c(OC)n1	0.2	9.80
	2014	CHEMBL3653276	CNC(=O)N1CCC(N2C(=O)c3nc(-c4cnc(OC)nc4OC)n(C(C)C)c3C2c2ccc(Cl)cc2)CC1	3.4	8.46
	2014	CHEMBL3653268	COc1cnc(N(C)CCO)nc1-c1nc2c(n1C(C)C)C(c1ccc(Cl)cc1)N(c1cccc(Cl)c1F)C2=O	0.7	9.15
	2015	CHEMBL3687251	Cc1cc(Cl)ccc1C1c2c(nn(CC(O)CO)c2C(C)C)C(=O)N1c1cc(Cl)ccc1C	2.2	8.66
	2014	CHEMBL3657067	COC[C@@H](C)n1c(-c2cnc(OC)nc2OC)nc2c1C(c1ccc(Cl)cc1)N(c1cccc(Cl)c1F)C2=O	0.2	9.82
	2014	CHEMBL3653255	COc1nc(N(C)C)ncc1-c1nc2c(n1C(C)C)C(c1ccc(C#N)cc1)N(c1cc(Cl)cn(C)c1=O)C2=O	0.6	9.19
	2015	CHEMBL3687315	COc1ccccc1-n1nc2c(c1C(C)C)C(c1ccc(Cl)cc1C)N(c1cc(Cl)ccc1OCCN(C)C)C2=O	1.7	8.77
	2014	CHEMBL3657086	COc1ncc(-c2nc3c(n2C(C)C)C(c2ccc(Cl)c(F)c2)N(c2cc(Cl)cn(C)c2=O)C3=O)c(OC)n1	0.5	9.28
	2015	CHEMBL3687288	CC(C)c1c2c(nn1CCO)C(=O)N(c1cccc(Cl)c1F)C2c1ccc(Cl)c(F)c1	7.8	8.11
	2014	CHEMBL3653246	COc1ccc(CO)cc1-c1nc2c(n1C(C)C)[C@@H](c1ccc(Cl)cc1C)N(c1cc(Cl)ccc1C)C2=O	1.1	8.96

	2015	CHEMBL3687281	Cc1cc(Cl)ccc1C1c2c(nn(-c3ccc(C#N)cc3)c2C(C)C)C(=O)N1c1cccc(Cl)c1F	6.9	8.16
	2015	CHEMBL3683124	CC(=O)NCCN(c1ccc2c(c1)C(c1ccc(Cl)cc1)N(c1ccc(N(C)Cc3ccncc3)cc1)C(=O)C2)C(C)C	120.1	6.92
	2014	CHEMBL3657129	COc1ncc(-c2nc3c(n2C(C)C)C(c2ccc(Cl)cc2)N(c2cc(Cl)cn(C)c2=O)C3=O)c(OC)n1	0.3	9.47
	2014	CHEMBL3653304	CCOC(=O)Cc1cnc(OC)c(-c2nc3c(n2C(C)C)C(c2ccc(Cl)cc2)N(c2cccc(Cl)c2F)C3=O)c1	1.0	9.00
	2015	CHEMBL3687275	COC(=O)N1CCCC1Cn1nc2c(c1C(C)C)C(c1ccc(Cl)cc1C)N(c1cc(Cl)ccc1C)C2=O	1.1	8.96
	2015	CHEMBL3639906	[C-]#[N+]c1ccc(C2c3c(nn(-c4cnc(OC)nc4OC)c3C(C)C)C(=O)N2c2cc(Cl)c(=O)n(C)c2)c(F)c1	4.1	8.39
	2015	CHEMBL3683136	CC[C@@H](C)Oc1cc2c(cc1OC)CC(=O)N(c1ccc(N(C)CC3CCN(S(C)(=O)=O)CC3)cc1)C2c1ccc(Cl)cc1	10.7	7.97
	2014	CHEMBL3657036	COc1ccc(CO)cc1-c1nc2c(n1C(C)C)C(c1ccc(Cl)cc1C)N(c1cc(Cl)ccn1)C2=O	5.8	8.24
	2015	CHEMBL3691711	COc1ccccc1-n1nc2c(c1C(C)C)C(c1ccc(Cl)cc1)N(c1cc(Cl)c(=O)n(CCN(C)C)c1)C2=O	3.8	8.42
	2014	CHEMBL3650057	CCCN(C=O)C1CCC(N2C(=O)Cc3cc(OC)c(OC(C)C)cc3C2c2ccc(Cl)cc2)CC1	189.0	6.72
	2014	CHEMBL3657048	COc1nnc(C(=O)N(C)C)cc1-c1nc2c(n1C(C)C)C(c1ccc(Cl)cc1)N(c1cccc(Cl)c1F)C2=O	2.2	8.65
	2014	CHEMBL3650063	COc1cc2c(cc1OC(C)C)C(c1ccc(Cl)cc1)N(C1CCC(C(O)C3CCOCC3)CC1)C(=O)C2	106.0	6.97
	2015	CHEMBL3691718	COc1ccncc1-n1nc2c(c1C(C)C)C(c1ccc(Cl)cc1C)N(c1cc(Cl)ccc1C)C2=O	2.0	8.70
	2015	CHEMBL3691794	COc1ncc(-n2nc3c(c2C(C)C)C(c2ccc(Cl)cc2)N(c2cc(C)cn(C)c2=O)C3=O)c(OC)n1	2.1	8.67

	2014	CHEMBL3656996	COc1ncc(-c2nc3c(n2C2CC2)C(c2ccc(Cl)cc2)N(c2cc(Cl)ccc2C)C3=O)c(OC)n1	1.3	8.88
	2014	CHEMBL3657066	COC[C@@H](C)n1c(-c2cnc(OC)nc2OC)nc2c1C(c1ccc(C#N)cc1)N(c1cc(Cl)ccc1C)C2=O	0.2	9.77
	2014	CHEMBL3653275	COc1ncc(-c2nc3c(n2C(C)C)C(c2ccc(Cl)cc2)N(c2cc(Cl)cnc2O)C3=O)c(OC)n1	0.3	9.60
	2015	CHEMBL3691792	COc1nc(N(C)C)ncc1-n1nc2c(c1C(C)C)C(c1ccc(Cl)c(F)c1)N(c1cc(Cl)c(=O)n(C)c1)C2=O	0.2	9.81
	2014	CHEMBL3653274	COc1cnc(N(C)CCO)nc1-c1nc2c(n1C(C)C)C(c1ccc(Cl)cc1)N(c1cc(Cl)ccc1C)C2=O	0.3	9.59
	2015	CHEMBL3691703	COc1ccccc1-n1nc2c(c1C(C)C)C(c1ccc(Cl)cc1)N(c1ccc(F)c(F)c1)C2=O	1.7	8.77
	2014	CHEMBL3653207	COc1nc(N(C)CCO)ncc1-c1nc2c(n1C(C)C)C(c1ccc(Cl)cc1C)N(c1cc(Cl)ccc1C)C2=O	0.2	9.80
	2014	CHEMBL3653208	COc1nc(NCCO)ncc1-c1nc2c(n1C(C)C)C(c1ccc(Cl)cc1C)N(c1cc(Cl)ccc1C)C2=O	0.2	9.77
	2015	CHEMBL3691744	COc1nc(N(C)C)ncc1-n1nc2c(c1C(C)C)C(c1ccc(Cl)cc1)N(c1cc(Cl)cn(C)c1=O)C2=O	0.1	9.92
	2015	CHEMBL3691709	CCn1cc(N2C(=O)c3nn(-c4ccccc4OC)c(C(C)C)c3C2c2ccc(Cl)cc2C)cc(Cl)c1=O	0.4	9.38
	2015	CHEMBL3687268	Cc1cc(Cl)ccc1C1c2c(nn(CCN3CCN(C)CC3)c2C(C)C)C(=O)N1c1cc(Cl)ccc1C	0.7	9.15
	2014	CHEMBL3653277	COc1ncc(-c2nc3c(n2C(C)C)C(c2ccc(C#N)cc2)N(c2cc(Cl)cn(C)c2=O)C3=O)c(OC)n1	2.3	8.63
	2015	CHEMBL3691715	COc1cnc(Cl)cc1N1C(=O)c2nn(-c3ccccc3OC)c(C(C)C)c2C1c1ccc(Cl)cc1C	2.1	8.68
	2015	CHEMBL3601401	COc1cc2c(cc1OC(C)C)[C@H](c1ccc(Cl)cc1)N(c1ccc(N(C)C[C@H]3CC[C@H](N4CCN(C)C(=O)C4)CC3)nc1)C(=O)C2	1.7	8.77

	2015	CHEMBL3683111	COc1ccc(N2C(=O)Nc3cc(OC)c(OC)cc3C2c2ccc(Cl)cc2)cc1	970.6	6.01
	2015	CHEMBL3687270	CCn1nc2c(c1C(C)C)C(c1ccc(Cl)cc1C)N(c1cc(Cl)ccc1OCCN(C)C)C2=O	4.1	8.39
	2015	CHEMBL3687266	Cc1cc(Cl)ccc1C1c2c(nn(CC(=O)N3CCCC3)c2C(C)C)C(=O)N1c1cc(Cl)ccc1C	2.0	8.70
	2014	CHEMBL3653284	COc1ncc(CC#N)cc1-c1nc2c(n1C(C)C)C(c1ccc(C#N)cc1)N(c1cc(Cl)ccc1C)C2=O	1.7	8.78
	2015	CHEMBL3687287	Cc1cc(Cl)ccc1C1c2c(nn(-c3ccc(C(N)=O)cc3)c2C(C)C)C(=O)N1c1cccc(Cl)c1F	11.5	7.94
	2014	CHEMBL3653197	[C-]#[N+]c1ccc(C2c3c(nc(-c4cnc(NC)nc4OC)n3C(C)C)C(=O)N2c2cc(Cl)ccc2C)cc1	0.2	9.82
	2014	CHEMBL3653216	COc1nc(N2CC(C)(O)C2)ncc1-c1nc2c(n1C(C)C)C(c1ccc(Cl)cc1C)N(c1cc(Cl)ccc1C)C2=O	0.2	9.74
	2014	CHEMBL3656993	CNc1ncc(-c2nc3c(n2C(C)C)C(c2ccc(Cl)cc2)N(c2cc(Cl)c[nH]c2=O)C3=O)c(OC)n1	0.2	9.72
	2014	CHEMBL3657090	COc1nc(N(C)C)ncc1-c1nc2c(n1C(C)C)[C@@H](c1ccc(C#N)c(F)c1)N(c1cc(Cl)cn(C)c1=O)C2=O	51.1	7.29
	2015	CHEMBL3683116	CC[C@@H](C)Oc1cc2c(cc1OCC(=O)N1CCN(C)CC1)CC(=O)N(c1ccc(OC)cc1)C2c1ccc(Cl)cc1	1381.1	5.86
	2015	CHEMBL3683126	CC[C@@H](C)Oc1cc2c(cc1OC)CC(=O)N(c1ccc(-n3cccn3)cc1)C2c1ccc(Cl)cc1	876.9	6.06
	2015	CHEMBL3691788	COc1nc(N(C)C)ncc1-n1nc2c(c1C(C)C)C(c1ccc(C#N)c(F)c1)N(c1cc(Cl)c[nH]c1=O)C2=O	0.2	9.67
	2015	CHEMBL3691743	COc1nc(N(C)C)ncc1-n1nc2c(c1C(C)C)C(c1ccc(Cl)cc1)N(c1cc(Cl)c[nH]c1=O)C2=O	0.1	9.89
	2014	CHEMBL3653303	CNC(=O)Cc1cnc(OC)c(-c2nc3c(n2C(C)C)C(c2ccc(C#N)cc2)N(c2cc(Cl)ccc2C)C3=O)c1	2.0	8.71

	2015	CHEMBL3687243	Cc1cc(Cl)ccc1C1c2c(nn(CC(=O)N3CCC(CCO)CC3)c2C(C)C)C(=O)N1c1cc(Cl)ccc1C	4.2	8.38
	2014	CHEMBL3656981	COc1nc(N(C)C)ncc1-c1nc2c(n1C(C)C)[C@@H](c1ccc(Cl)cc1)N(c1cc(Cl)cnc1O)C2=O	39.6	7.40
	2015	CHEMBL3691725	COc1ccc(C#N)cc1-n1nc2c(c1C(C)C)C(c1ccc(Cl)cc1C)N(c1cc(Cl)c(=O)n(C)c1)C2=O	0.4	9.42
	2015	CHEMBL3691727	COc1ccc(CNC(C)=O)cc1-n1nc2c(c1C(C)C)C(c1ccc(Cl)cc1C)N(c1cc(Cl)c(=O)n(C)c1)C2=O	0.4	9.40
	2015	CHEMBL3691746	COc1nc(N(C)C)ncc1-n1nc2c(c1C(C)C)C(c1ccc(C#N)cc1)N(c1cc(Cl)c(=O)n(C)c1)C2=O	0.3	9.60
	2014	CHEMBL3657052	COc1cnc(CC#N)cc1-c1nc2c(n1C(C)C)C(c1ccc(Cl)cc1)N(c1ccc(F)c(Cl)c1)C2=O	0.4	9.39
	2014	CHEMBL3650061	COc1cc2c(cc1OC(C)C)C(c1ccc(Cl)cc1)N(C1CCC(C(C)=O)CC1)C(=O)C2	993.0	6.00
	2014	CHEMBL3657125	COC[C@@H](C)n1c(-c2cnc(N(C)C)nc2OC)nc2c1[C@@H](c1ccc(Cl)cc1)N(c1cc(Cl)c(=O)n(C)c1)C2=O	6.1	8.21
	2014	CHEMBL3650048	COc1cc2c(cc1OC(C)C)C(c1ccc(Cl)cc1)N(C1CCC(N(C)C(C)=O)CC1)C(=O)C2	386.0	6.41
	2014	CHEMBL3657027	COc1ccc(C(=O)N2CCOCC2)cc1-c1nc2c(n1C(C)C)C(c1ccc(Cl)cc1C)N(c1cccc(Cl)c1)C2=O	0.5	9.27
	2015	CHEMBL3691702	COc1ccc(C(=O)N(C)C)cc1-n1nc2c(c1C(C)C)C(c1ccc(Cl)cc1C)N(c1cc(Cl)ccc1C)C2=O	2.1	8.68
	2015	CHEMBL3687308	Cc1cc(Cl)ccc1C1c2c(nn(CCO)c2C2CC2)C(=O)N1c1cccc(Cl)c1F	23.2	7.63
	2015	CHEMBL3691767	COc1nc(N(C)C)ncc1-n1nc2c(c1C(C)C)[C@H](c1ccc(C#N)cc1)N(c1cc(Cl)cn(C)c1=O)C2=O	0.1	9.89
	2014	CHEMBL3657006	COc1ccccc1-c1nc2c(n1C(C)C)C(c1ccc(C#N)cc1C)N(c1cc(Cl)ccc1C)C2=O	0.3	9.57

	2014	CHEMBL3657106	COc1nc(N(C)C)ncc1-c1nc2c(n1C(C)C)C(c1ccc(Cl)cc1)N(c1c(F)c(C)nn1C)C2=O	0.2	9.69
	2014	CHEMBL3653187	CNc1ncc(-c2nc3c(n2C(C)C)C(c2ccc(Cl)cc2)N(c2cc(Cl)ccc2C)C3=O)c(OC)n1	0.2	9.72
	2014	CHEMBL3657047	COc1ccc(C(C)O)cc1-c1nc2c(n1C(C)C)C(c1ccc(Cl)cc1)N(c1cccc(Cl)c1F)C2=O	0.2	9.68
	2015	CHEMBL3687273	Cc1cc(Cl)ccc1C1c2c(nn(C(=O)N3CCN(C)CC3)c2C(C)C)C(=O)N1c1cc(Cl)ccc1C	2.7	8.57
	2014	CHEMBL3657121	COC[C@@H](C)n1c(-c2cnc(N(C)C)nc2OC)nc2c1C(c1ccc(Cl)cc1)N(c1cc(Cl)c(=O)n(C)c1)C2=O	0.1	9.84
	2015	CHEMBL3691793	COc1nc(N(C)C)ncc1-n1nc2c(c1C(C)C)C(c1ccc(Cl)c(F)c1)N(c1c[nH]c(=O)c(Cl)c1)C2=O	0.2	9.79
	2014	CHEMBL3656989	COc1cnc(N(C)C)nc1-c1nc2c(n1C(C)C)C(c1ccc(Cl)cc1)N(c1cc(Cl)ccc1C)C2=O	0.3	9.54
	2014	CHEMBL3653221	COc1ncc(-c2nc3c(n2C(C)C)C(c2ccc(Cl)cc2)N(c2cc(Cl)c(=O)n(C)c2)C3=O)c(OC)n1	3.6	8.45
	2015	CHEMBL3691769	CNc1ncc(-n2nc3c(c2C(C)C)C(c2ccc(C#N)cc2)N(c2cc(Cl)c[nH]c2=O)C3=O)c(OC)n1	0.7	9.18
	2014	CHEMBL3653242	COc1ncc(-c2nc3c(n2C2CCC2)C(c2ccc(Cl)cc2)N(c2cc(Cl)ccc2C)C3=O)c(OC)n1	4.6	8.34
	2015	CHEMBL3687257	Cc1nc(Cn2nc3c(c2C(C)C)C(c2ccc(Cl)cc2C)N(c2cccc(Cl)c2F)C3=O)cs1	6.2	8.21
	2014	CHEMBL3657114	COC[C@@H](C)n1c(-c2cnc(N)nc2OC)nc2c1C(c1ccc(Cl)cc1)N(c1cc(Cl)ccc1C)C2=O	0.2	9.67
Bioorg. Med. Chem. Lett.	2016	CHEMBL3139629	O=C(c1cnccc1C(F)(F)F)N1CCCC(Oc2ccc(C(F)(F)F)cc2)(C(=O)N2CCN(c3ccccn3)CC2)C1	2300.0	5.64

Bioorg. Med. Chem. Lett.	2016	CHEMBL3797513	CCC[C@H]1N(C(=O)c2cnccc2C(F)(F)F)CCC[C@@]1(Oc1ccc(C(F)(F)F)cc1)C(=O)N1CCC(Oc2cccc(C(=O)O)n2)CC1	42.0	7.38
Bioorg. Med. Chem. Lett.	2016	CHEMBL3799031	CCC[C@H]1N(C(=O)c2cnccc2C(F)(F)F)CCC[C@@]1(Oc1ccc(C(F)(F)F)cc1)C(=O)N1CCC(Oc2cccc(C(=O)OC)n2)CC1	392.0	6.41
Bioorg. Med. Chem. Lett.	2016	CHEMBL3799374	CCC[C@H]1N(C(=O)c2cnccc2C(F)(F)F)CCC[C@@]1(Oc1ccc(C(F)(F)F)cc1)C(=O)N1CCC(Oc2cccc(C(=O)NC)n2)CC1	114.0	6.94
Bioorg. Med. Chem. Lett.	2016	CHEMBL3798875	CCC[C@H]1N(C(=O)c2cnccc2C(F)(F)F)CCC[C@@]1(Oc1ccc(C(F)(F)F)cc1)C(=O)N1CCC(Oc2ncccc2C(N)=O)CC1	4276.0	5.37
Bioorg. Med. Chem. Lett.	2016	CHEMBL3797454	CCC[C@H]1N(C(=O)c2cnccc2C(F)(F)F)CCC[C@@]1(Oc1ccc(C(F)(F)F)cc1)C(=O)N1CCC(Oc2cc(C(N)=O)ccn2)CC1	8721.0	5.06
Bioorg. Med. Chem. Lett.	2016	CHEMBL3800127	CCC[C@H]1N(C(=O)c2cnccc2C(F)(F)F)CCC[C@@]1(Oc1ccc(C(F)(F)F)cc1)C(=O)N1CCC(Oc2ccc(C(N)=O)cn2)CC1	144.0	6.84
Bioorg. Med. Chem. Lett.	2016	CHEMBL3798136	CCC[C@H]1N(C(=O)c2cnccc2C(F)(F)F)CCC[C@@]1(Oc1ccc(C(F)(F)F)cc1)C(=O)N1CCC(Oc2cccc(C(N)=O)n2)CC1	111.0	6.95
Bioorg. Med. Chem. Lett.	2016	CHEMBL3797705	CCC[C@H]1N(C(=O)c2cnccc2C(F)(F)F)CCC[C@@]1(Oc1ccc(C(F)(F)F)cc1)C(=O)N1CCC(Oc2ncccc2CN)CC1	7744.0	5.11
Bioorg. Med.	2016	CHEMBL3800088	CCC[C@H]1N(C(=O)c2cnccc2C(F)(F)F)CCC[C@@]1(Oc1ccc(C(F)(F)F)cc1)C(=O)N1CCC(Oc2cc(CN)ccn2)CC1	4026.0	5.40

Chem. Lett.					
Bioorg. Med. Chem. Lett.	2016	CHEMBL3798867	CCC[C@H]1N(C(=O)c2cnccc2C(F)(F)F)CCC[C@@]1(Oc1ccc(C(F)(F)F)cc1)C(=O)N1CCC(Oc2ccc(CN)cn2)CC1	801.0	6.10
Bioorg. Med. Chem. Lett.	2016	CHEMBL3799552	CCC[C@H]1N(C(=O)c2cnccc2C(F)(F)F)CCC[C@@]1(Oc1ccc(C(F)(F)F)cc1)C(=O)N1CCC(Oc2cccc(CN)n2)CC1	173.0	6.76
Bioorg. Med. Chem. Lett.	2016	CHEMBL3797694	CCC[C@H]1N(C(=O)c2cnccc2C(F)(F)F)CCC[C@@]1(Oc1ccc(C(F)(F)F)cc1)C(=O)N1CCC(Oc2ncccc2C#N)CC1	2399.0	5.62
Bioorg. Med. Chem. Lett.	2016	CHEMBL3798271	CCC[C@H]1N(C(=O)c2cnccc2C(F)(F)F)CCC[C@@]1(Oc1ccc(C(F)(F)F)cc1)C(=O)N1CCC(Oc2cc(C#N)ccn2)CC1	293.0	6.53
Bioorg. Med. Chem. Lett.	2016	CHEMBL3798889	CCC[C@H]1N(C(=O)c2cnccc2C(F)(F)F)CCC[C@@]1(Oc1ccc(C(F)(F)F)cc1)C(=O)N1CCC(Oc2ccc(C#N)cn2)CC1	492.0	6.31
Bioorg. Med. Chem. Lett.	2016	CHEMBL3799050	CCC[C@H]1N(C(=O)c2cnccc2C(F)(F)F)CCC[C@@]1(Oc1ccc(C(F)(F)F)cc1)C(=O)N1CCC(Oc2cccc(C#N)n2)CC1	686.0	6.16
Bioorg. Med. Chem. Lett.	2016	CHEMBL3798126	CCC[C@H]1N(C(=O)c2cnccc2C(F)(F)F)CCC[C@@]1(Oc1ccc(C(F)(F)F)cc1)C(=O)N1CCC(Oc2cccc(Cl)n2)CC1	2359.0	5.63
Bioorg. Med. Chem. Lett.	2016	CHEMBL3797577	CCC[C@H]1N(C(=O)c2cnccc2C(F)(F)F)CCC[C@@]1(Oc1ccc(C(F)(F)F)cc1)C(=O)N1CCC(Oc2cccc(OC)n2)CC1	1146.0	5.94

Bioorg. Med. Chem. Lett.	2016	CHEMBL3800561	CCC[C@H]1N(C(=O)c2cnccc2C(F)(F)F)CCC[C@@]1(Oc1ccc(C(F)(F)F)cc1)C(=O)N1CCC(Oc2cccc(C)n2)CC1	5027.0	5.30
Bioorg. Med. Chem. Lett.	2016	CHEMBL3800178	CCC[C@H]1N(C(=O)c2cnccc2C(F)(F)F)CCC[C@@]1(Oc1ccc(C(F)(F)F)cc1)C(=O)N1CCC(Oc2cncnc2)CC1	525.0	6.28
Bioorg. Med. Chem. Lett.	2016	CHEMBL3797521	CCC[C@H]1N(C(=O)c2cnccc2C(F)(F)F)CCC[C@@]1(Oc1ccc(C(F)(F)F)cc1)C(=O)N1CCC(Oc2ccncn2)CC1	376.0	6.42
Bioorg. Med. Chem. Lett.	2016	CHEMBL3799390	CCC[C@H]1N(C(=O)c2cnccc2C(F)(F)F)CCC[C@@]1(Oc1ccc(C(F)(F)F)cc1)C(=O)N1CCC(Oc2cnccn2)CC1	1065.0	5.97
Bioorg. Med. Chem. Lett.	2016	CHEMBL3798706	CCC[C@H]1N(C(=O)c2cnccc2C(F)(F)F)CCC[C@@]1(Oc1ccc(C(F)(F)F)cc1)C(=O)N1CCC(Oc2ncccn2)CC1	334.0	6.48
Bioorg. Med. Chem. Lett.	2016	CHEMBL3797331	CCC[C@H]1N(C(=O)c2cnccc2C(F)(F)F)CCC[C@@]1(Oc1ccc(C(F)(F)F)cc1)C(=O)N1CCC(Oc2ccncc2)CC1	224.0	6.65
Bioorg. Med. Chem. Lett.	2016	CHEMBL3798260	CCC[C@H]1N(C(=O)c2cnccc2C(F)(F)F)CCC[C@@]1(Oc1ccc(C(F)(F)F)cc1)C(=O)N1CCC(Oc2cccnc2)CC1	267.0	6.57
Bioorg. Med. Chem. Lett.	2016	CHEMBL3799375	CCC[C@H]1N(C(=O)c2cnccc2C(F)(F)F)CCC[C@@]1(Oc1ccc(C(F)(F)F)cc1)C(=O)N1CCC(Oc2ccccn2)CC1	150.0	6.82
Bioorg. Med.	2016	CHEMBL3798021	CCC[C@H]1N(C(=O)c2cnccc2C(F)(F)F)CCC[C@@]1(Oc1ccc(C(F)(F)F)cc1)C(=O)N1CCC(Oc2ccc(C#N)cc2)CC1	1919.0	5.72

Chem. Lett.					
Bioorg. Med. Chem. Lett.	2016	CHEMBL3798014	CCC[C@H]1N(C(=O)c2cnccc2C(F)(F)F)CCC[C@@]1(Oc1ccc(C(F)(F)F)cc1)C(=O)N1CCC(Oc2cccc(C#N)c2)CC1	399.0	6.40
Bioorg. Med. Chem. Lett.	2016	CHEMBL3797254	CCC[C@H]1N(C(=O)c2cnccc2C(F)(F)F)CCC[C@@]1(Oc1ccc(C(F)(F)F)cc1)C(=O)N1CCC(Oc2ccccc2C#N)CC1	8714.0	5.06
Bioorg. Med. Chem. Lett.	2016	CHEMBL3800543	CCC[C@H]1N(C(=O)c2cnccc2C(F)(F)F)CCC[C@@]1(Oc1ccc(C(F)(F)F)cc1)C(=O)N1CCC(Oc2ccccc2)CC1	2400.0	5.62
J. Med. Chem.	2016	CHEMBL3819416	CCOC(=O)[C@H](Cc1ccccc1)NC(=O)[C@H](CC(C)C)NC(=O)C(=O)c1c(-c2ccccc2)[nH]c2ccccc12	11.7	7.93
J. Med. Chem.	2016	CHEMBL3819071	CC[C@H](C)[C@H](NC(=O)[C@@H](NC(=O)C(=O)c1c(-c2ccccc2)[nH]c2ccccc12)C(C)C)C(=O)OC	15.2	7.82
J. Med. Chem.	2016	CHEMBL3818934	CC[C@H](C)[C@H](NC(=O)C(=O)c1c(-c2ccccc2)[nH]c2ccccc12)C(=O)N[C@H](C(=O)OC)C(C)C	77.7	7.11
J. Med. Chem.	2016	CHEMBL3818951	CC[C@H](C)[C@H](NC(=O)C(=O)c1c(-c2ccccc2)[nH]c2ccccc12)C(=O)N[C@H](C(=O)OC)[C@@H](C)CC	11.7	7.93
J. Med. Chem.	2016	CHEMBL3819467	COC(=O)[C@@H](NC(=O)[C@H](CC(C)C)NC(=O)C(=O)c1c(-c2ccccc2)[nH]c2ccccc12)C(C)C	154.6	6.81
J. Med. Chem.	2016	CHEMBL3818586	COC(=O)[C@H](CC(C)C)NC(=O)[C@@H](NC(=O)C(=O)c1c(-c2ccccc2)[nH]c2ccccc12)C(C)C	24.8	7.61

J. Med. Chem.	2016	CHEMBL3818299	CNC(=O)[C@H](CC(C)C)NC(=O)[C@H](Cc1ccccc1)NC(=O)C(=O)c1c(-c2ccccc2)[nH]c2ccccc12	4.3	8.37
J. Med. Chem.	2016	CHEMBL3819001	CC[C@H](C)[C@H](NC(=O)[C@H](Cc1ccccc1)NC(=O)C(=O)c1c(-c2ccccc2)[nH]c2ccccc12)C(=O)NC	9.8	8.01
	2015	CHEMBL3703679	Cc1cc(Cl)cnc1NC(c1ccc2cccnc2c1O)c1c(F)c(F)c(OCC(F)(F)F)c(F)c1F	8500.0	5.07
	2013	CHEMBL3675198	N#Cc1ccc(CN2C(=O)c3ccccc3C2(O[C@H]2C=C[C@@H](O)C2)c2ccc(Cl)cc2)cc1	2300.0	5.64
	2013	CHEMBL1688255	O=C1c2ccccc2C(O[C@H]2C=C[C@@H](O)C2)(c2ccc(Cl)cc2)N1Cc1ccc(Cl)cc1	1500.0	5.82
	2013	CHEMBL1688256	Cc1ccc(CN2C(=O)c3ccccc3C2(O[C@H]2C=C[C@@H](O)C2)c2ccc(Cl)cc2)cc1	1400.0	5.85
	2013	CHEMBL1688254	O=C1c2ccccc2C(O[C@H]2C=C[C@@H](O)C2)(c2ccc(Cl)cc2)N1Cc1ccccc1	2300.0	5.64
	2013	CHEMBL1688264	O=C1c2ccccc2C(OCCCO)(c2ccc(Cl)cc2)N1CCc1ccc([N+](=O)[O-])cc1	3200.0	5.49
	2013	CHEMBL1688263	C[C@H](c1ccc(Cl)cc1)N1C(=O)c2ccccc2C1(OCCCO)c1ccc(Cl)cc1	2500.0	5.60
	2013	CHEMBL1688261	N#Cc1ccc(CN2C(=O)c3ccccc3C2(OCCCO)c2ccc(Cl)cc2)cc1	3500.0	5.46
	2013	CHEMBL1688257	O=C1c2ccccc2C(O[C@H]2C=C[C@@H](O)C2)(c2ccc(Cl)cc2)N1Cc1ccc([N+](=O)[O-])cc1	1400.0	5.85
	2013	CHEMBL1688258	O=C1c2ccccc2C(OC/C=C\CO)(c2ccc(Cl)cc2)N1Cc1ccc([N+](=O)[O-])cc1	402.0	6.40
	2013	CHEMBL3675200	O=C1c2ccccc2C(OC/C=C/CO)(c2ccc(Cl)cc2)N1Cc1ccc([N+](=O)[O-])cc1	405.0	6.39

	2013	CHEMBL3675201	O=C1c2ccccc2C(O[C@H]2CCC[C@@H](O)CCC2)(c2ccc(Cl)cc2)N1Cc1ccc([N+](=O)[O-])cc1	375.0	6.43
	2013	CHEMBL1688273	CC(C)(CO)COC1(c2ccc(Cl)cc2)c2ccccc2C(=O)N1Cc1ccc([N+](=O)[O-])cc1	395.0	6.40
	2013	CHEMBL1688274	O=C1c2ccccc2C(OCC2(CO)CC2)(c2ccc(Cl)cc2)N1Cc1ccc([N+](=O)[O-])cc1	298.0	6.53
	2013	CHEMBL1688271	O=C1c2ccccc2C(OCC#CCO)(c2ccc(Cl)cc2)N1Cc1ccc([N+](=O)[O-])cc1	656.0	6.18
	2013	CHEMBL1688268	O=C1c2ccccc2C(OC[C@H]2CC[C@H](CO)CC2)(c2ccc(Cl)cc2)N1Cc1ccc([N+](=O)[O-])cc1	582.0	6.24
	2013	CHEMBL1688272	O=C1c2ccccc2C(OC[C@@H]2CCCC[C@@H]2CO)(c2ccc(Cl)cc2)N1Cc1ccc([N+](=O)[O-])cc1	569.0	6.24
	2013	CHEMBL3928363	O=C1c2ccccc2C(OC2CCC(O)CC2)(c2ccc(Cl)cc2)N1Cc1ccc([N+](=O)[O-])cc1	388.0	6.41
	2013	CHEMBL1688276	O=C1c2ccccc2C(O[C@@H]2C=C[C@@H](O)CC2)(c2ccc(Cl)cc2)N1Cc1ccc([N+](=O)[O-])cc1	306.0	6.51
	2013	CHEMBL1688262	C[C@@H](c1ccc(Cl)cc1)N1C(=O)c2ccccc2C1(OCCCCO)c1ccc(Cl)cc1	869.0	6.06
	2013	CHEMBL1688267	O=C1c2ccccc2C(OCc2ccc(CO)cc2)(c2ccc(Cl)cc2)N1Cc1ccc([N+](=O)[O-])cc1	983.0	6.01
	2013	CHEMBL1688269	O=C1c2ccccc2C(OCc2cccc(CO)c2)(c2ccc(Cl)cc2)N1Cc1ccc([N+](=O)[O-])cc1	732.0	6.14
	2013	CHEMBL3675203	NCCCOC1(c2ccc(Cl)cc2)c2ccccc2C(=O)N1Cc1ccc([N+](=O)[O-])cc1	2460.0	5.61
	2013	CHEMBL1688266	O=C1c2ccccc2C(OCCCCO)(c2ccc(Br)cc2)N1Cc1ccc([N+](=O)[O-])cc1	570.0	6.24
	2013	CHEMBL3675204	O=C1c2ccccc2C(OCC2(CO)CC2)(c2ccc(Br)cc2)N1Cc1ccc([N+](=O)[O-])cc1	368.0	6.43

	2013	CHEMBL1688265	O=C1c2ccccc2C(OCCCO)(c2ccc(Br)cc2)N1Cc1ccc([N+](=O)[O-])cc1	1400.0	5.85
	2013	CHEMBL3675205	Cc1cccc2c1C(O)(c1ccc(Cl)cc1)N(Cc1ccc([N+](=O)[O-])cc1)C2=O	2200.0	5.66
	2013	CHEMBL3675206	Cc1cccc2c1C(=O)N(Cc1ccc([N+](=O)[O-])cc1)C2(O)c1ccc(Cl)cc1	6900.0	5.16
	2013	CHEMBL3675207	Cc1ccc2c(c1)C(O)(c1ccc(Cl)cc1)N(Cc1ccc([N+](=O)[O-])cc1)C2=O	5080.0	5.29
	2013	CHEMBL3675209	CC(C)(C)c1ccc2c(c1)C(=O)N(Cc1ccc([N+](=O)[O-])cc1)C2(O)c1ccc(Cl)cc1	837.0	6.08
	2013	CHEMBL1834378	CC(C)(C)c1ccc2c(c1)C(=O)N(Cc1ccc([N+](=O)[O-])cc1)C2(OCC1(CO)CC1)c1ccc(Cl)cc1	152.0	6.82
	2013	CHEMBL3675211	O=C1c2ccc(F)cc2C(O)(c2ccc(Cl)cc2)N1Cc1ccc([N+](=O)[O-])cc1	3760.0	5.42
	2013	CHEMBL3675212	O=C1c2cc(F)ccc2C(O)(c2ccc(Cl)cc2)N1Cc1ccc([N+](=O)[O-])cc1	5190.0	5.28
	2013	CHEMBL1834381	O=C1c2cc(Cl)c(Cl)cc2C(OCC2(CO)CC2)(c2ccc(Cl)cc2)N1Cc1ccc([N+](=O)[O-])cc1	3670.0	5.44
	2013	CHEMBL3675213	N#Cc1ccc(CN2C(=O)c3cccc(Cl)c3C2(O)c2ccc(Cl)cc2)cc1	1620.0	5.79
	2013	CHEMBL3675214	N#Cc1ccc(CN2C(=O)c3c(Cl)cccc3C2(O)c2ccc(Cl)cc2)cc1	8950.0	5.05
	2013	CHEMBL3675215	O=C1c2cccc(Cl)c2C(O)(c2ccc(Cl)cc2)N1Cc1ccc(Br)cc1	847.0	6.07
	2013	CHEMBL1834288	N#Cc1ccc(CN2C(=O)c3cccc(Cl)c3C2(OCC2(CO)CC2)c2ccc(Cl)cc2)cc1	185.0	6.73
	2013	CHEMBL1834287	O=C1c2cccc(Cl)c2C(OCC2(CO)CC2)(c2ccc(Cl)cc2)N1Cc1ccc(Br)cc1	169.0	6.77

	2013	CHEMBL3675216	O=C1C2=C(CCCC2)C(O)(c2ccc(Cl)cc2)N1Cc1ccc([N+](=O)[O-])cc1	2810.0	5.55
	2013	CHEMBL1834374	O=C1c2ccc(F)cc2C(OCC2(CO)CC2)(c2ccc(Cl)cc2)N1Cc1ccc([N+](=O)[O-])cc1	295.0	6.53
	2013	CHEMBL1834379	O=C1c2cc(F)ccc2C(OCC2(CO)CC2)(c2ccc(Cl)cc2)N1Cc1ccc([N+](=O)[O-])cc1	852.0	6.07
	2013	CHEMBL1834376	CC(C)(C)c1ccc2c(c1)C(OCC1(CO)CC1)(c1ccc(Cl)cc1)N(Cc1ccc([N+](=O)[O-])cc1)C2=O	733.0	6.13
	2013	CHEMBL1834375	Cc1ccc2c(c1)C(OCC1(CO)CC1)(c1ccc(Cl)cc1)N(Cc1ccc([N+](=O)[O-])cc1)C2=O	492.0	6.31
	2013	CHEMBL1834373	Cc1cccc2c1C(OCC1(CO)CC1)(c1ccc(Cl)cc1)N(Cc1ccc([N+](=O)[O-])cc1)C2=O	274.0	6.56
	2013	CHEMBL3675217	Cc1ccc2c(c1)C(=O)N(Cc1ccc([N+](=O)[O-])cc1)C2(O)c1ccc(Cl)cc1	1460.0	5.84
	2013	CHEMBL3675218	O=C1c2cc(Br)ccc2C(O)(c2ccc(Cl)cc2)N1Cc1ccc([N+](=O)[O-])cc1	5370.0	5.27
	2013	CHEMBL3675219	O=C1c2ccc(Br)cc2C(O)(c2ccc(Cl)cc2)N1Cc1ccc([N+](=O)[O-])cc1	5680.0	5.25
	2013	CHEMBL1688277	N#Cc1ccc(CN2C(=O)c3ccccc3C2(OCC2(CO)CC2)c2ccc(Cl)cc2)cc1	1790.0	5.75
	2013	CHEMBL1688279	O=C1c2ccccc2C(OCC2(CO)CC2)(c2ccc(Cl)cc2)N1Cc1ccc(Cl)cc1	2310.0	5.64
	2013	CHEMBL1688280	O=C1c2ccccc2C(OCC2(CO)CC2)(c2ccc(Cl)cc2)N1Cc1ccc(Br)cc1	1200.0	5.92
	2013	CHEMBL3675220	CC(c1ccc(Cl)cc1)N1C(=O)c2ccccc2C1(OCC1(CO)CC1)c1ccc(Cl)cc1	8800.0	5.06
	2013	CHEMBL1688285	C[C@@H](c1ccc(Cl)cc1)N1C(=O)c2ccccc2C1(OCC1(CO)CC1)c1ccc(Cl)cc1	8900.0	5.05

	2013	CHEMBL1834377	O=C1c2ccc(Br)cc2C(OCC2(CO)CC2)(c2ccc(Cl)cc2)N1Cc1ccc([N+](=O)[O-])cc1	902.0	6.04
	2013	CHEMBL1834380	O=C1c2cc(Br)ccc2C(OCC2(CO)CC2)(c2ccc(Cl)cc2)N1Cc1ccc([N+](=O)[O-])cc1	1030.0	5.99
	2013	CHEMBL3675210	O=C1c2cccc(Cl)c2C(O)(c2ccc(Cl)cc2)N1Cc1ccc([N+](=O)[O-])cc1	510.0	6.29
	2013	CHEMBL1834286	O=C1c2cccc(Cl)c2C(OCC2(CO)CC2)(c2ccc(Cl)cc2)N1Cc1ccc([N+](=O)[O-])cc1	1260.0	5.90
	2013	CHEMBL3675221	O=C1c2c(Cl)cccc2C(O)(c2ccc(Cl)cc2)N1Cc1ccc([N+](=O)[O-])cc1	4540.0	5.34
	2013	CHEMBL1688281	O=C1c2ccccc2C(OCC2(CO)CC2)(c2ccc(Cl)cc2)N1Cc1ccc(I)cc1	1500.0	5.82
	2013	CHEMBL3675222	O=C1c2ccccc2C(OCC2(CO)CC2)(c2ccccc2)N1Cc1ccc([N+](=O)[O-])cc1	8300.0	5.08
	2013	CHEMBL3675223	O=C(O)CCC(=O)OCC1(COC2(c3ccc(Cl)cc3)c3c(Cl)cccc3C(=O)N2Cc2ccc([N+](=O)[O-])cc2)CC1	19.0	7.72
	2013	CHEMBL3675224	N#Cc1ccc(CN2C(=O)c3cccc(Cl)c3C2(OCC2(COC(=O)CCC(=O)O)CC2)c2ccc(Cl)cc2)cc1	102.0	6.99
	2013	CHEMBL3675225	O=C(O)CCC(=O)OCC1(COC2(c3ccc(Cl)cc3)c3c(Cl)cccc3C(=O)N2Cc2ccc(Br)cc2)CC1	102.0	6.99
	2013	CHEMBL1688282	Cc1ccc(CN2C(=O)c3ccccc3C2(OCC2(CO)CC2)c2ccc(Cl)cc2)cc1	2300.0	5.64
	2013	CHEMBL1688283	COc1ccc(CN2C(=O)c3ccccc3C2(OCC2(CO)CC2)c2ccc(Cl)cc2)cc1	2800.0	5.55
	2013	CHEMBL3675226	C=C(CO)COC1(c2ccc(Cl)cc2)c2ccccc2C(=O)N1Cc1ccc([N+](=O)[O-])cc1	680.0	6.17
	2013	CHEMBL3675227	O=C1c2ccccc2C(OCC2(CO)CC2)(c2ccc(F)cc2)N1Cc1ccc([N+](=O)[O-])cc1	2700.0	5.57

	2014	CHEMBL3955477	CC1(C)N[C@@H](C(=O)NC2CCC(O)CC2)[C@H](c2cccc(Cl)c2F)[C@]12C(=O)Nc1cc(Cl)ccc12	201.0	6.70
	2014	CHEMBL3895325	O=C(NC1CCC(O)CC1)[C@@H]1NC2(CCCC2)[C@@]2(C(=O)Nc3cc(Cl)ccc32)[C@H]1c1cccc(Cl)c1F	41.8	7.38
	2014	CHEMBL3960776	O=C(NC1CCC(O)CC1)[C@@H]1NC2(CCCCCC2)[C@@]2(C(=O)Nc3cc(Cl)ccc32)[C@H]1c1cccc(Cl)c1F	15.6	7.81
	2014	CHEMBL3355418	CCC1(CC)N[C@@H](C(=O)NCCN2CCOCC2)[C@H](c2cccc(Cl)c2F)[C@]12C(=O)Nc1cc(Cl)ccc12	1000.0	6.00
	2014	CHEMBL3693957	O=C(NCCN1CCOCC1)[C@@H]1NC2(CCC(F)(F)CC2)[C@@]2(C(=O)Nc3cc(Cl)ccc32)[C@H]1c1cccc(Cl)c1F	1000.0	6.00
	2014	CHEMBL3693958	O=C(NCCN1CCOCC1)[C@@H]1NC2(CCC(F)(F)CC2)[C@]2(C(=O)Nc3cc(Cl)ccc32)[C@H]1c1cccc(Cl)c1F	5000.0	5.30
	2014	CHEMBL3918865	O=C(NC1CCC(O)CC1)[C@@H]1NC2(CCC(F)(F)CC2)[C@@]2(C(=O)Nc3cc(Cl)ccc32)[C@H]1c1cccc(Cl)c1F	100.0	7.00
	2014	CHEMBL3943264	O=C(NC1CCC(O)CC1)[C@@H]1NC2(CCOCC2)[C@]2(C(=O)Nc3cc(Cl)ccc32)[C@H]1c1cccc(Cl)c1F	5000.0	5.30
	2014	CHEMBL3920081	O=C(NC1CCC(O)CC1)[C@@H]1NC2(CCOCC2)[C@@]2(C(=O)Nc3cc(Cl)ccc32)[C@H]1c1cccc(Cl)c1F	5000.0	5.30
	2014	CHEMBL3355421	O=C(NCCN1CCOCC1)[C@@H]1NC2(CCCCC2)[C@@]2(C(=O)Nc3cc(Cl)ccc32)[C@H]1c1cccc(Cl)c1F	100.0	7.00
	2014	CHEMBL3693968	CS(=O)(=O)CCNC(=O)[C@@H]1NC2(CCCCC2)[C@@]2(C(=O)Nc3cc(Cl)ccc32)[C@H]1c1cccc(Cl)c1F	100.0	7.00
	2014	CHEMBL3693970	CS(=O)(=O)CCN1CCC(NC(=O)[C@@H]2NC3(CCCCC3)[C@@]3(C(=O)Nc4cc(Cl)ccc43)[C@H]2c2cccc(Cl)c2F)CC1	100.0	7.00
	2014	CHEMBL3693971	O=C(O)[C@@H]1NC2(CCCCC2)[C@@]2(C(=O)Nc3cc(Cl)ccc32)[C@H]1c1cccc(Cl)c1F	500.0	6.30
	2014	CHEMBL3942080	O=C(O)C1CCC(CNC(=O)[C@@H]2NC3(CCCCC3)[C@@]3(C(=O)Nc4cc(Cl)ccc43)[C@H]2c2cccc(Cl)c2F)CC1	100.0	7.00

	2014	CHEMBL3931297	CC1(O)CC(NC(=O)[C@@H]2NC3(CCCCC3)[C@@]3(C(=O)Nc4cc(Cl)ccc43)[C@H]2c2cccc(Cl)c2F)C1	100.0	7.00
	2014	CHEMBL3693979	CNC(=O)[C@@H]1NC2(CCCCC2)[C@@]2(C(=O)Nc3cc(Cl)ccc32)[C@H]1c1cc(Cl)ccc1OC(C)(C)C(=O)O	10000.0	5.00
	2014	CHEMBL3693980	CNC(=O)[C@@H]1NC2(CCCCC2)[C@@]2(C(=O)Nc3cc(Cl)ccc32)[C@H]1c1cc(Cl)cnc1OC(C)(C)C(=O)O	10000.0	5.00
	2014	CHEMBL3693984	O=C(NC1CN(C(=O)O)C1)[C@@H]1NC2(CCCCC2)[C@@]2(C(=O)Nc3cc(Cl)ccc32)[C@H]1c1cccc(Cl)c1F	100.0	7.00
	2014	CHEMBL3916402	O=C(O)C1CCC(NC(=O)[C@@H]2NC3(CCCCC3)[C@@]3(C(=O)Nc4cc(Cl)ccc43)[C@H]2c2cccc(Cl)c2F)CC1	100.0	7.00
	2014	CHEMBL3986151	O=C(NC1CCC(O)CC1)[C@@H]1NC2(CCCCC2)[C@@]2(C(=O)Nc3cc(Cl)ccc32)[C@H]1c1cccc(Cl)c1F	100.0	7.00
	2014	CHEMBL3665613	CC(C)(C)C[C@@H]1N(N(C(=O)C(F)(F)F)C2CC(C)(O)C2)[C@@H](C(=O)O)[C@H](c2cccc(F)c2F)C12C(=O)Nc1cc(Cl)c(F)cc12	388.7	6.41
	2014	CHEMBL3976723	O=C(NC1CCC(O)CC1)[C@@H]1N[C@H](CC2CCCC2)[C@]2(CNc3cc(Cl)ccc32)[C@H]1c1cccc(Cl)c1F	3000.0	5.52
	2014	CHEMBL3929802	CC(C)(C)C[C@@H]1N[C@@H](C(=O)NC2CCC(O)CC2)[C@H](c2cccc(Cl)c2)[C@]12C(=O)Nc1cc(Cl)c(F)cc12	100.0	7.00
	2014	CHEMBL3915391	O=C(NC1CCC(O)CC1)[C@@H]1N[C@@H](c2ccccc2)[C@@]2(C(=O)Nc3cc(Cl)ccc32)[C@H]1c1cccc(Cl)c1F	500.0	6.30
	2014	CHEMBL3981908	O=C(NC1CCC(O)CC1)[C@@H]1N[C@@H](c2ccccc2)[C@@]2(C(=O)Nc3cc(Cl)ccc32)[C@H]1Cc1ccccc1	5000.0	5.30
	2014	CHEMBL3985078	CC(C)(C)C[C@@H]1N[C@@H](C(=O)NC2CCC(O)CC2)[C@H](Cc2ccccc2)[C@]12C(=O)Nc1cc(Cl)ccc12	5000.0	5.30
	2014	CHEMBL3665611	CCN1[C@@H](CC(C)(C)C)[C@@]2(C(=O)Nc3cc(Cl)ccc32)[C@@H](c2cccc(Cl)c2F)[C@@H]1C(=O)N[C@H]1C[C@@](C)(O)C1	100.0	7.00

	2014	CHEMBL3665612	CN1[C@@H](CC(C)(C)C)[C@@]2(C(=O)Nc3cc(Cl)ccc32)[C@@H](c2cccc(Cl)c2F)[C@@H]1C(=O)N[C@H]1C[C@@](C)(O)C1	100.0	7.00
	2014	CHEMBL3927643	CC(C)(C)C[C@@H]1N[C@@H](C(=O)NC2CCC(O)CC2)[C@H](c2cccc(Cl)c2F)[C@@]12C(=O)Nc1cc(Cl)ccc12	5900.0	5.23
	2014	CHEMBL3665607	CC(C)(C)C[C@@H]1N[C@@H](C(=O)N[C@H]2C[C@@](C)(O)C2)[C@H](c2cc(Cl)ccc2F)[C@]12C(=O)Nc1cc(Cl)ccc12	159.0	6.80
	2014	CHEMBL3938875	Cc1c(C(=O)O)nc(N[C@@H](C)C2CCC2)c2c1nc(-c1cc(C(C)C)ccn1)n2Cc1ccc(C(F)(F)F)cc1	60.0	7.22
	2014	CHEMBL3936690	CC(C)c1ccnc(-c2nc3cc(C(=O)O)nc(N[C@@H](C)C4CCC4)c3n2Cc2ccc(C(F)(F)F)cc2)c1	29.0	7.54
	2014	CHEMBL3915460	Cc1cccc(-c2nc3cc(C(=O)O)nc(N[C@@H](C)C4CCC4)c3n2Cc2ccc(C(F)(F)F)cc2)c1	122.0	6.91
	2014	CHEMBL3647045	Cc1ccc(Cn2c(-c3cc(C(C)C)ccn3)nc3cc(C(=O)O)nc(N[C@H](C)C4CCC4)c32)cc1	4.0	8.40
	2014	CHEMBL3931087	C[C@H]1COC[C@H](C)N1c1nc2cc(-c3nnc(O)o3)nc(-c3cncc(Cl)c3)c2n1C[C@H]1CC[C@H](C)CC1	7.0	8.15
	2014	CHEMBL3944856	C[C@H]1COCCN1c1nc2cc(-c3nc(=O)o[nH]3)nc(-c3cncc(Cl)c3)c2n1C[C@H]1CC[C@H](C)CC1	4.0	8.40
	2014	CHEMBL3968578	C[C@H]1CC[C@H](C)N1c1nc2cc(-c3nc(=O)o[nH]3)nc(-c3cncc(Cl)c3)c2n1C[C@H]1CC[C@H](C)CC1	2.0	8.70
	2014	CHEMBL3649830	C[C@@H]1CCCCN1c1nc2cc(-c3noc(=O)[nH]3)nc(-c3cncc(Cl)c3)c2n1C[C@H]1CC[C@H](C)CC1	4.0	8.40
	2014	CHEMBL3649831	C[C@@H]1C[C@@H](O)CN1c1nc2cc(-c3noc(=O)[nH]3)nc(-c3cncc(Cl)c3)c2n1C[C@H]1CC[C@H](C)CC1	6.0	8.22
	2014	CHEMBL3649832	C[C@H]1CC[C@H](Cn2c(N3CCO[C@@H]4CCC[C@H]43)nc3cc(C(=O)O)nc(-c4cncc(Cl)c4)c32)CC1	2.0	8.70
	2014	CHEMBL3984635	CO[C@H]1C[C@H](C)N(c2nc3cc(-c4nc(=O)o[nH]4)nc(-c4cncc(Cl)c4)c3n2C[C@H]2CC[C@H](C)CC2)C1	4.0	8.40

	2014	CHEMBL3649836	C[C@H]1CC[C@H](Cn2c(N3CCOC4COCC43)nc3cc(-c4noc(=O)[nH]4)cc(-c4cncc(Cl)c4)c32)CC1	29.0	7.54
	2014	CHEMBL3952519	C[C@H]1CCCN1c1nc2cc(-c3nc(=O)o[nH]3)nc(-c3cncc(Cl)c3)c2n1C[C@H]1CC[C@H](C)CC1	4.0	8.40
	2014	CHEMBL3649852	C[C@H]1CC[C@H](Cn2c(N3CCCC3c3nccs3)nc3cc(-c4noc(=O)[nH]4)nc(-c4cncc(Cl)c4)c32)CC1	1.0	9.00
	2014	CHEMBL3898360	C[C@H]1CN(C)C(=O)CN1c1nc2cc(-c3nc(=O)o[nH]3)nc(-c3cncc(Cl)c3)c2n1C[C@H]1CC[C@H](C)CC1	11.0	7.96
	2014	CHEMBL3942858	COC1CC[C@H](C)N(c2nc3cc(-c4nc(=O)o[nH]4)nc(-c4cncc(Cl)c4)c3n2C[C@H]2CC[C@H](C)CC2)C1	3.0	8.52
	2014	CHEMBL3981115	CO[C@H]1C[C@H](C(C)C)N(c2nc3cc(-c4nc(=O)o[nH]4)nc(-c4cncc(Cl)c4)c3n2C[C@H]2CC[C@H](C)CC2)C1	1.0	9.00
	2014	CHEMBL3649862	CC(C)C1C[C@@H](O)CN1c1nc2cc(-c3noc(=O)[nH]3)nc(-c3cncc(Cl)c3)c2n1C[C@H]1CC[C@H](C)CC1	1.0	9.00
	2014	CHEMBL3927925	CO[C@H]1C[C@@H](CF)N(c2nc3cc(-c4nc(=O)o[nH]4)nc(-c4cncc(Cl)c4)c3n2C[C@H]2CC[C@H](C)CC2)C1	353.0	6.45
	2014	CHEMBL3649866	CO[C@H]1COC[C@@H]1N(C)c1nc2cc(-c3noc(=O)[nH]3)nc(-c3cncc(Cl)c3)c2n1C[C@H]1CC[C@H](C)CC1	26.0	7.59
	2014	CHEMBL3969880	CO[C@H]1CCN(c2nc3cc(-c4nc(=O)o[nH]4)nc(-c4cncc(Cl)c4)c3n2C[C@H]2CC[C@H](C)CC2)[C@@H](C)C1	16.0	7.80
	2014	CHEMBL3945674	CO[C@@H]1CCN(c2nc3cc(-c4nc(=O)o[nH]4)nc(-c4cncc(Cl)c4)c3n2C[C@H]2CC[C@H](C)CC2)[C@@H](C)C1	18.0	7.74
	2014	CHEMBL3929391	CC1CCC(Cn2c(N3CCCC4COCCC43)nc3cc(-c4noc(=O)[nH]4)nc(-c4cncc(Cl)c4)c32)CC1	8.0	8.10
	2014	CHEMBL4107347	CC1CCC(Cn2c(N3CCO[C@@H]4CN(C(=O)OCc5ccccc5)CC[C@H]43)nc3cc(-c4noc(=O)[nH]4)nc(-c4cncc(Cl)c4)c32)CC1	250.0	6.60
	2014	CHEMBL4111957	CC1CCC(Cn2c(N3CCO[C@@H]4CNCC[C@H]43)nc3cc(-c4noc(=O)[nH]4)nc(-c4cncc(Cl)c4)c32)CC1	512.0	6.29

	2014	CHEMBL4113308	CC1CCC(Cn2c(N3CCO[C@@H]4CN(Cc5ccccc5)CC[C@H]43)nc3cc(-c4noc(=O)[nH]4)nc(-c4cncc(Cl)c4)c32)CC1	129.0	6.89
	2014	CHEMBL3907252	CO[C@H]1C[C@H](C2(OC)CC2)N(c2nc3cc(-c4nc(=O)o[nH]4)nc(-c4cncc(Cl)c4)c3n2C[C@H]2CC[C@H](C)CC2)C1	2.0	8.70
	2014	CHEMBL3925339	CO[C@H]1C[C@@H](C2(OC)CC2)N(c2nc3cc(-c4nc(=O)o[nH]4)nc(-c4cncc(Cl)c4)c3n2C[C@H]2CC[C@H](C)CC2)C1	10.0	8.00
	2014	CHEMBL3944443	CO[C@H]1C[C@H](C(F)F)N(c2nc3cc(-c4nc(=O)o[nH]4)nc(-c4cncc(Cl)c4)c3n2C[C@H]2CC[C@H](C)CC2)C1	1.0	9.00
	2014	CHEMBL3895119	CCN1C(=O)CN(c2nc3cc(-c4nc(=O)o[nH]4)nc(-c4cncc(Cl)c4)c3n2C[C@H]2CC[C@H](C)CC2)[C@H]2CCC[C@@H]21	4.0	8.40
	2014	CHEMBL3966426	COCCO[C@H]1C[C@H](C(C)C)N(c2nc3cc(-c4nc(=O)o[nH]4)nc(-c4cncc(Cl)c4)c3n2C[C@H]2CC[C@H](C)CC2)C1	1.0	9.00
	2014	CHEMBL3649901	C[C@H]1CC[C@H](Cn2c(N3CCO[C@@H]4CCC(F)(F)[C@H]43)nc3cc(-c4noc(=O)[nH]4)nc(-c4cncc(Cl)c4)c32)CC1	1.0	9.00
	2014	CHEMBL3971691	CC1CO[C@H]2CCC[C@@H]2N1c1nc2cc(-c3nc(=O)o[nH]3)nc(-c3cncc(Cl)c3)c2n1C[C@H]1CC[C@H](C)CC1	1.0	9.00
	2014	CHEMBL3909594	CC[C@H]1[C@H](C)OCCN1c1nc2cc(-c3nc(=O)o[nH]3)nc(-c3cncc(Cl)c3)c2n1C[C@H]1CC[C@H](C)CC1	1.0	9.00
	2014	CHEMBL3979791	C[C@H]1CC[C@H](Cn2c(N3CCO[C@H]4CC(F)(F)C[C@@H]43)nc3cc(-c4nc(=O)o[nH]4)nc(-c4cncc(Cl)c4)c32)CC1	130.0	6.89
	2014	CHEMBL3927923	C[C@H]1CC[C@H](Cn2c(N3CCO[C@H]4CC(F)C[C@@H]43)nc3cc(-c4nc(=O)o[nH]4)nc(-c4cncc(Cl)c4)c32)CC1	48.0	7.32
	2014	CHEMBL3649916	CC1CN(c2nc3cc(-c4noc(=O)[nH]4)nc(-c4cncc(Cl)c4)c3n2C[C@H]2CC[C@H](C)CC2)[C@H](C)[C@@H](C)O1	1.0	9.00
	2014	CHEMBL3971736	CC([C@H]1CC[C@H](C)CC1)n1c(N2CCO[C@H]3CCC[C@@H]32)nc2cc(-c3nc(=O)o[nH]3)nc(-c3cncc(Cl)c3)c21	1.0	9.00
	2014	CHEMBL3966472	CC([C@H]1CC[C@H](C)CC1)n1c(N2CCOC[C@@H]2C)nc2cc(-c3nc(=O)o[nH]3)nc(-c3cncc(Cl)c3)c21	1.0	9.00

	2014	CHEMBL3957580	CC([C@H]1CC[C@H](C)CC1)n1c(N2CC(=O)N(C)C[C@@H]2C)nc2cc(-c3nc(=O)o[nH]3)nc(-c3cncc(Cl)c3)c21	13.0	7.89
	2014	CHEMBL3649927	CC(C)[C@@H]1C[C@@H](O)CN1c1nc2cc(-c3noc(=O)[nH]3)nc(-c3cncc(Cl)c3)c2n1C(C)[C@H]1CC[C@H](C)CC1	1.0	9.00
	2014	CHEMBL3649929	CC([C@H]1CC[C@H](C)CC1)n1c(N2C[C@H](O)C[C@H]2C)nc2cc(-c3noc(=O)[nH]3)nc(-c3cncc(Cl)c3)c21	3.0	8.52
	2014	CHEMBL3649928	CO[C@@H]1C[C@@H](C)N(c2nc3cc(-c4noc(=O)[nH]4)nc(-c4cncc(Cl)c4)c3n2C(C)[C@H]2CC[C@H](C)CC2)C1	2.0	8.70
	2014	CHEMBL3649930	CC(=O)N1CCN(c2nc3cc(-c4noc(=O)[nH]4)nc(-c4cncc(Cl)c4)c3n2C[C@H]2CC[C@H](C)CC2)[C@H](C)C1	6.0	8.22
	2014	CHEMBL3649931	C[C@@H]1CN(C(=O)C2CC2)CCN1c1nc2cc(-c3noc(=O)[nH]3)nc(-c3cncc(Cl)c3)c2n1C[C@H]1CC[C@H](C)CC1	5.0	8.30
	2014	CHEMBL3649932	C[C@@H]1CN(C(=O)C2CC2)C[C@@H](C)N1c1nc2cc(-c3noc(=O)[nH]3)nc(-c3cncc(Cl)c3)c2n1C[C@H]1CC[C@H](C)CC1	1.0	9.00
	2014	CHEMBL3894230	C[C@H]1CN(S(C)(=O)=O)C[C@H](C)N1c1nc2cc(-c3nc(=O)o[nH]3)nc(-c3cncc(Cl)c3)c2n1C[C@H]1CC[C@H](C)CC1	3.0	8.52
	2014	CHEMBL3965668	CCOC(=O)N1C[C@H](C)N(c2nc3cc(-c4nc(=O)o[nH]4)nc(-c4cncc(Cl)c4)c3n2C[C@H]2CC[C@H](C)CC2)[C@@H](C)C1	2.0	8.70
	2014	CHEMBL3986537	CCNC(=O)N1C[C@H](C)N(c2nc3cc(-c4nc(=O)o[nH]4)nc(-c4cncc(Cl)c4)c3n2C[C@H]2CC[C@H](C)CC2)[C@@H](C)C1	3.0	8.52
	2014	CHEMBL3954453	CCC(=O)N1C[C@H](C)N(c2nc3cc(-c4nc(=O)o[nH]4)nc(-c4cncc(Cl)c4)c3n2C[C@H]2CC[C@H](C)CC2)[C@@H](C)C1	3.0	8.52
	2014	CHEMBL3978308	C[C@H]1CN(C(=O)C2CCC2)C[C@H](C)N1c1nc2cc(-c3nc(=O)o[nH]3)nc(-c3cncc(Cl)c3)c2n1C[C@H]1CC[C@H](C)CC1	1.0	9.00
	2014	CHEMBL3649938	CCCC(=O)N1C[C@@H](C)N(c2nc3cc(-c4noc(=O)[nH]4)nc(-c4cncc(Cl)c4)c3n2C[C@H]2CC[C@H](C)CC2)[C@H](C)C1	3.0	8.52
	2014	CHEMBL3649939	COC(=O)N1C[C@@H](C)N(c2nc3cc(-c4noc(=O)[nH]4)nc(-c4cncc(Cl)c4)c3n2C[C@H]2CC[C@H](C)CC2)[C@H](C)C1	2.0	8.70

	2014	CHEMBL3649940	CC(C)OC(=O)N1C[C@@H](C)N(c2nc3cc(-c4noc(=O)[nH]4)nc(-c4cncc(Cl)c4)c3n2C[C@H]2CC[C@H](C)CC2)[C@H](C)C1	3.0	8.52
	2014	CHEMBL3649941	CCCNC(=O)N1C[C@@H](C)N(c2nc3cc(-c4noc(=O)[nH]4)nc(-c4cncc(Cl)c4)c3n2C[C@H]2CC[C@H](C)CC2)[C@H](C)C1	2.0	8.70
	2014	CHEMBL3649942	CC(C)NC(=O)N1C[C@@H](C)N(c2nc3cc(-c4noc(=O)[nH]4)nc(-c4cncc(Cl)c4)c3n2C[C@H]2CC[C@H](C)CC2)[C@H](C)C1	3.0	8.52
	2014	CHEMBL3979460	CC(C)C(=O)N1C[C@H](C)N(c2nc3cc(-c4nc(=O)o[nH]4)nc(-c4cncc(Cl)c4)c3n2C[C@H]2CC[C@H](C)CC2)[C@@H](C)C1	3.0	8.52
	2014	CHEMBL3909218	C[C@H]1CC[C@H](Cn2c(N3CCN(C(=O)C4CC4)[C@H]4CCC[C@@H]43)nc3cc(-c4nc(=O)o[nH]4)nc(-c4cncc(Cl)c4)c32)CC1	5.0	8.30
	2014	CHEMBL3972127	CC(=O)N1CCN(c2nc3cc(-c4nc(=O)o[nH]4)nc(-c4cncc(Cl)c4)c3n2C[C@H]2CC[C@H](C)CC2)[C@H]2CCC[C@@H]21	4.0	8.40
	2014	CHEMBL3946174	C[C@H]1CC[C@H](Cn2c(N3CCN(C(=O)C(F)F)[C@H]4CCC[C@@H]43)nc3cc(-c4nc(=O)o[nH]4)nc(-c4cncc(Cl)c4)c32)CC1	3.0	8.52
	2014	CHEMBL3963317	C[C@H]1CC[C@H](Cn2c(N3CCN(C(=O)C4CCC4)[C@H]4CCC[C@@H]43)nc3cc(-c4nc(=O)o[nH]4)nc(-c4cncc(Cl)c4)c32)CC1	5.0	8.30
	2014	CHEMBL3958237	C[C@H]1CN(C(=O)N(C)C)C[C@H](C)N1c1nc2cc(-c3nc(=O)o[nH]3)nc(-c3cncc(Cl)c3)c2n1C[C@H]1CC[C@H](C)CC1	1.0	9.00
	2014	CHEMBL3939191	CCN(C)C(=O)N1C[C@H](C)N(c2nc3cc(-c4nc(=O)o[nH]4)nc(-c4cncc(Cl)c4)c3n2C[C@H]2CC[C@H](C)CC2)[C@@H](C)C1	1.0	9.00
	2014	CHEMBL3649952	C[C@@H]1CN(C(=O)C2(F)CC2)C[C@@H](C)N1c1nc2cc(-c3noc(=O)[nH]3)nc(-c3cncc(Cl)c3)c2n1C[C@H]1CC[C@H](C)CC1	1.0	9.00
	2014	CHEMBL3649953	C[C@@H]1CN(C(=O)C2CC2(F)F)C[C@@H](C)N1c1nc2cc(-c3noc(=O)[nH]3)nc(-c3cncc(Cl)c3)c2n1C[C@H]1CC[C@H](C)CC1	4.0	8.40
	2014	CHEMBL3649954	C[C@@H]1CN(C(=O)C(F)F)C[C@@H](C)N1c1nc2cc(-c3noc(=O)[nH]3)nc(-c3cncc(Cl)c3)c2n1C[C@H]1CC[C@H](C)CC1	4.0	8.40
	2014	CHEMBL3649955	C[C@@H]1CN(C(=O)C2(C)COC2)C[C@@H](C)N1c1nc2cc(-c3noc(=O)[nH]3)nc(-c3cncc(Cl)c3)c2n1C[C@H]1CC[C@H](C)CC1	11.0	7.96

	2014	CHEMBL3649956	C[C@@H]1CN(C(=O)C2(C)CC2)C[C@@H](C)N1c1nc2cc(-c3noc(=O)[nH]3)nc(-c3cncc(Cl)c3)c2n1C[C@H]1CC[C@H](C)CC1	10.0	8.00
	2014	CHEMBL3649957	C[C@@H]1CN(C(=O)C2COC2)C[C@@H](C)N1c1nc2cc(-c3noc(=O)[nH]3)nc(-c3cncc(Cl)c3)c2n1C[C@H]1CC[C@H](C)CC1	4.0	8.40
	2014	CHEMBL3649958	COCC(=O)N1C[C@@H](C)N(c2nc3cc(-c4noc(=O)[nH]4)nc(-c4cncc(Cl)c4)c3n2C[C@H]2CC[C@H](C)CC2)[C@H](C)C1	10.0	8.00
	2014	CHEMBL3650001	C[C@H]1CC[C@H](Cn2c(N3CCO[C@@H]4CCC[C@H]43)nc3c(Cl)c(-c4noc(=O)[nH]4)nc(-c4cncc(Cl)c4)c32)CC1	1.0	9.00
	2014	CHEMBL3650002	C[C@H]1CC[C@H](Cn2c(N3CCO[C@@H]4CCC[C@H]43)nc3c(F)c(-c4noc(=O)[nH]4)nc(-c4cncc(Cl)c4)c32)CC1	1.0	9.00
	2014	CHEMBL3971319	COc1c(-c2nc(=O)o[nH]2)nc(-c2cncc(Cl)c2)c2c1nc(N1CCO[C@H]3CCC[C@@H]31)n2C[C@H]1CC[C@H](C)CC1	1.0	9.00
	2014	CHEMBL3906549	Cc1c(-c2nc(=O)o[nH]2)nc(-c2cncc(Cl)c2)c2c1nc(N1CCO[C@H]3CCC[C@@H]31)n2C[C@H]1CC[C@H](C)CC1	2.0	8.70
	2014	CHEMBL3934510	CN(C)c1c(C(=O)O)nc(-c2cncc(Cl)c2)c2c1nc(N1CCO[C@H]3CCC[C@@H]31)n2C[C@H]1CC[C@H](C)CC1	24.0	7.62
	2014	CHEMBL3650006	CS(=O)(=O)NC(=O)c1cc2nc(N3CCO[C@@H]4CCC[C@H]43)n(C[C@H]3CC[C@H](C)CC3)c2c(-c2cncc(Cl)c2)n1	1.0	9.00
	2014	CHEMBL3957470	CN(C)S(=O)(=O)NC(=O)c1cc2nc(N3CCO[C@H]4CCC[C@@H]43)n(C[C@H]3CC[C@H](C)CC3)c2c(-c2cncc(Cl)c2)n1	2.0	8.70
	2014	CHEMBL3650014	C[C@H]1CC[C@H](Cn2c(N3CCO[C@@H]4CCC[C@H]43)nc3cc(-c4nnn[nH]4)nc(-c4cncc(Cl)c4)c32)CC1	1.0	9.00
	2014	CHEMBL3943959	CCOC(=O)c1cc2nc(N3CCOC[C@H]3c3ccccc3)n(CC3CCC(C)CC3)c2c(-c2cncc(Cl)c2)n1	53.0	7.28
	2014	CHEMBL3650017	CCON(c1nc2cc(-c3noc(=O)[nH]3)nc(-c3cncc(Cl)c3)c2n1C(C)[C@H]1CC[C@H](C)CC1)C1CC1	1.0	9.00
	2014	CHEMBL3650021	CCON(COC)c1nc2cc(-c3noc(=O)[nH]3)nc(-c3cncc(Cl)c3)c2n1C(C)[C@H]1CC[C@H](C)CC1	3.0	8.52

	2014	CHEMBL3650022	CC([C@H]1CC[C@H](C)CC1)n1c(N2CCN(C(=O)C3(F)CC3)C[C@H]2C)nc2cc(-c3noc(=O)[nH]3)nc(-c3cncc(Cl)c3)c21	1.0	9.00
	2014	CHEMBL3650023	O=c1[nH]c(-c2cc3nc(N4CCO[C@@H]5CCC[C@H]54)n(Cc4ccc(C(F)(F)F)cc4)c3c(-c3cncc(Cl)c3)n2)no1	39.0	7.41
	2014	CHEMBL3650024	CC[C@H]1CC[C@H](Cn2c(N3CCO[C@@H]4CCC[C@H]43)nc3cc(-c4noc(=O)[nH]4)nc(-c4cncc(Cl)c4)c32)CC1	5.0	8.30
	2014	CHEMBL3653310	CC[C@H]1CC[C@H](Cn2c(N3CCO[C@@H]4CCC[C@H]43)nc3cc(-c4n[nH]c(=O)o4)nc(-c4cncc(Cl)c4)c32)CC1	25.0	7.60
	2014	CHEMBL3653311	O=c1[nH]c(-c2cc3nc(N4CCO[C@@H]5CCC[C@H]54)n(Cc4ccc(C(F)(F)F)c(F)c4)c3c(-c3cncc(Cl)c3)n2)no1	28.0	7.55
	2014	CHEMBL3947091	O=c1nc(-c2cc3nc(N4CCO[C@H]5CCC[C@@H]54)n(C[C@H]4CC[C@H](C(F)(F)F)CC4)c3c(-c3cncc(Cl)c3)n2)[nH]o1	38.0	7.42
	2014	CHEMBL3914761	CCC1CCC(Cn2c(N3CCO[C@H]4CCC[C@@H]43)nc3cc(-c4nc(=O)o[nH]4)nc(-c4cncc(Cl)c4)c32)C1	16.0	7.80
	2014	CHEMBL3890287	CN(C)c1ncc(Cl)cc1-c1nc(-c2nc(=O)o[nH]2)cc2nc(N3CCO[C@H]4CCC[C@@H]43)n(C[C@H]3CC[C@H](C)CC3)c12	2.0	8.70
	2014	CHEMBL3971378	C[C@H]1CC[C@H](Cn2c(N3CCO[C@H]4CCC[C@@H]43)nc3cc(-c4nc(=O)o[nH]4)nc(-c4cc(Cl)cnc4OCCO)c32)CC1	1.0	9.00
	2014	CHEMBL3984644	C[C@H]1CC[C@H](Cn2c(N3CCO[C@H]4CCC[C@@H]43)nc3cc(-c4nc(=O)o[nH]4)nc(-c4cc(Cl)cnc4O)c32)CC1	2.0	8.70
	2014	CHEMBL3653325	CNc1ncc(Cl)cc1-c1nc(-c2noc(=O)[nH]2)cc2nc(N3CCO[C@@H]4CCC[C@H]43)n(C[C@H]3CC[C@H](C)CC3)c12	2.0	8.70
	2014	CHEMBL3653336	C[C@H]1CC[C@H](Cn2c(N3CCOC[C@H]3c3ccccc3)nc3cc(C4CC=CC(=O)N4)nc(-c4cncc(Cl)c4)c32)CC1	70.0	7.15
	2015	CHEMBL3647042	Cc1c(C(=O)O)nc(N[C@H](C)C2CCC2)c2c1nc(-c1cc(C(C)C)ccn1)n2Cc1ccc(C(F)(F)F)cc1	60.0	7.22
	2015	CHEMBL3647043	CC(C)c1ccnc(-c2nc3cc(C(=O)O)nc(N[C@H](C)C4CCC4)c3n2Cc2ccc(C(F)(F)F)cc2)c1	29.0	7.54

	2015	CHEMBL3647044	Cc1cccc(-c2nc3cc(C(=O)O)nc(N[C@H](C)C4CCC4)c3n2Cc2ccc(C(F)(F)F)cc2)c1	122.0	6.91
	2015	CHEMBL3654183	CC(C)c1ccnc(-c2nc3cc(C(=O)O)nc(N[C@H](C)C4CCC4)c3n2C[C@H]2CC[C@H](C)CC2)c1	4.0	8.40
	2015	CHEMBL3647046	C[C@H]1CC[C@H](Cn2c(N3CCOC[C@H]3c3ccccc3)nc3cc(-c4noc(=O)[nH]4)nc(-c4cncc(Cl)c4)c32)CC1	1.0	9.00
	2015	CHEMBL3647047	C[C@H]1CC[C@H](Cn2c(N3CCOC[C@H]3c3ccccc3)nc3cc(-c4n[nH]c(=O)o4)nc(-c4cncc(Cl)c4)c32)CC1	1.0	9.00
	2015	CHEMBL3647048	Cc1cccc(-c2nc(-c3noc(=O)[nH]3)cc3nc(N4CCOC[C@H]4c4ccccc4)n(C[C@H]4CC[C@H](C)CC4)c23)c1	3.0	8.52
	2015	CHEMBL3647049	C[C@H]1CC[C@H](Cn2c(N3CCOC[C@H]3c3ccccc3)nc3cc(-c4noc(=O)[nH]4)nc(-c4cccc(Cl)c4)c32)CC1	3.0	8.52
	2015	CHEMBL3647050	C[C@H]1CC[C@H](Cn2c(N3CCOC[C@H]3c3ccccc3)nc3cc(-c4n[nH]c(=O)o4)nc(-c4cccc(Cl)c4)c32)CC1	9.0	8.05
	2015	CHEMBL3647051	C[C@H]1CC[C@H](Cn2c(N3CCC[C@H]3CF)nc3cc(-c4noc(=O)[nH]4)nc(-c4cncc(Cl)c4)c32)CC1	1.0	9.00
	2015	CHEMBL3647052	C[C@H]1CC[C@H](Cn2c(N3CCO[C@@H]4CCC[C@H]43)nc3cc(-c4noc(=O)[nH]4)nc(-c4cncc(Cl)c4)c32)CC1	1.0	9.00
	2015	CHEMBL3647053	C[C@H]1CC[C@H](Cn2c(N3CCCC3C(F)(F)F)nc3cc(-c4noc(=O)[nH]4)nc(-c4cncc(Cl)c4)c32)CC1	1.0	9.00
	2015	CHEMBL3647054	C[C@H]1CC[C@H](Cn2c(N3CCOC4CCCC43)nc3cc(-c4noc(=O)[nH]4)nc(-c4cncc(Cl)c4)c32)CC1	2.0	8.70
	2015	CHEMBL3647056	C[C@H]1CC[C@H](Cn2c(N3CCOC4CCCC43)nc3cc(-c4n[nH]c(=O)o4)nc(-c4cncc(Cl)c4)c32)CC1	4.0	8.40
	2015	CHEMBL3647057	COC(C)(C)[C@@H]1CCCN1c1nc2cc(-c3noc(=O)[nH]3)nc(-c3cncc(Cl)c3)c2n1C[C@H]1CC[C@H](C)CC1	1.0	9.00
	2015	CHEMBL3647058	C[C@H]1CC[C@H](Cn2c(N3CCC[C@H]3CF)nc3cc(-c4n[nH]c(=O)o4)nc(-c4cncc(Cl)c4)c32)CC1	4.0	8.40

	2015	CHEMBL3647059	C[C@H]1CC[C@H](Cn2c(N3CCO[C@H]4CCC[C@@H]43)nc3cc(-c4noc(=O)[nH]4)nc(-c4cncc(Cl)c4)c32)CC1	59.0	7.23
	2015	CHEMBL3647055	C[C@H]1CC[C@H](Cn2c(N3CCOC4CCCCC43)nc3cc(-c4noc(=O)[nH]4)nc(-c4cncc(Cl)c4)c32)CC1	17.0	7.77
	2015	CHEMBL3647061	COC(C)(C)[C@@H]1CCCN1c1nc2cc(-c3n[nH]c(=O)o3)nc(-c3cncc(Cl)c3)c2n1C[C@H]1CC[C@H](C)CC1	2.0	8.70
	2015	CHEMBL3647062	C[C@H]1CC[C@H](Cn2c(N3CCO[C@H]4CCC[C@@H]43)nc3cc(-c4n[nH]c(=O)o4)nc(-c4cncc(Cl)c4)c32)CC1	164.0	6.79
	2015	CHEMBL3647063	C[C@H]1CC[C@H](Cn2c(N3CCO[C@@H]4CCC[C@H]43)nc3cc(-c4n[nH]c(=O)o4)nc(-c4cncc(Cl)c4)c32)CC1	2.0	8.70
	2015	CHEMBL3647064	C[C@@H]1COC[C@@H](C)N1c1nc2cc(-c3noc(=O)[nH]3)nc(-c3cncc(Cl)c3)c2n1C[C@H]1CC[C@H](C)CC1	2.0	8.70
	2015	CHEMBL3647060	C[C@H]1CC[C@H](Cn2c(N3CCOC4CCCCC43)nc3cc(-c4n[nH]c(=O)o4)nc(-c4cncc(Cl)c4)c32)CC1	4.0	8.40
	2015	CHEMBL3647065	C[C@@H]1COC[C@@H](C)N1c1nc2cc(-c3n[nH]c(=O)o3)nc(-c3cncc(Cl)c3)c2n1C[C@H]1CC[C@H](C)CC1	7.0	8.15
	2015	CHEMBL3647066	C[C@H]1CC[C@H](Cn2c(N3CCC[C@H]3C(F)(F)F)nc3cc(-c4n[nH]c(=O)o4)nc(-c4cncc(Cl)c4)c32)CC1	2.0	8.70
	2015	CHEMBL3647067	C[C@H]1CC[C@H](Cn2c(N3CCOC[C@H]3c3ccccc3)nc3cc(C(=O)O)nc(-c4cncc(Cl)c4)c32)CC1	1.0	9.00
	2015	CHEMBL3647068	C[C@@H]1COCCN1c1nc2cc(-c3noc(=O)[nH]3)nc(-c3cncc(Cl)c3)c2n1C[C@H]1CC[C@H](C)CC1	4.0	8.40
	2015	CHEMBL3647069	C[C@H]1COC[C@H](C)N1c1nc2cc(-c3noc(=O)[nH]3)nc(-c3cncc(Cl)c3)c2n1C[C@H]1CC[C@H](C)CC1	157.0	6.80
	2015	CHEMBL3647070	C[C@H]1CC[C@H](Cn2c(N3CCCC4CCCC43)nc3cc(-c4noc(=O)[nH]4)nc(-c4cncc(Cl)c4)c32)CC1	1.0	9.00
	2015	CHEMBL3649814	C[C@H]1CC[C@H](Cn2c(N3CCC4C3C4C(F)(F)F)nc3cc(-c4noc(=O)[nH]4)nc(-c4cncc(Cl)c4)c32)CC1	32.0	7.49

	2015	CHEMBL3649815	CCC1COCCN1c1nc2cc(-c3noc(=O)[nH]3)nc(-c3cncc(Cl)c3)c2n1C[C@H]1CC[C@H](C)CC1	3.0	8.52
	2015	CHEMBL3649816	C[C@H]1CC[C@H](Cn2c(N3CCOC4CC43)nc3cc(-c4noc(=O)[nH]4)nc(-c4cncc(Cl)c4)c32)CC1	43.0	7.37
	2015	CHEMBL3649818	CC1COCCCN1c1nc2cc(-c3noc(=O)[nH]3)nc(-c3cncc(Cl)c3)c2n1C[C@H]1CC[C@H](C)CC1	16.0	7.80
	2015	CHEMBL3649819	CC(C)C1CCCN1c1nc2cc(-c3noc(=O)[nH]3)nc(-c3cncc(Cl)c3)c2n1C[C@H]1CC[C@H](C)CC1	1.0	9.00
	2015	CHEMBL3649820	C[C@@H]1CC[C@@H](C)N1c1nc2cc(-c3noc(=O)[nH]3)nc(-c3cncc(Cl)c3)c2n1C[C@H]1CC[C@H](C)CC1	2.0	8.70
	2015	CHEMBL3649817	CC1CN(c2nc3cc(-c4noc(=O)[nH]4)nc(-c4cncc(Cl)c4)c3n2C[C@H]2CC[C@H](C)CC2)C(C)CO1	20.0	7.70
	2015	CHEMBL3649821	C[C@H]1CC[C@H](Cn2c(N3CCC[C@H]3C(F)(F)F)nc3cc(C(=O)O)nc(-c4cncc(Cl)c4)c32)CC1	1.0	9.00
	2015	CHEMBL3649823	COC(C)C1CCCN1c1nc2cc(-c3noc(=O)[nH]3)nc(-c3cncc(Cl)c3)c2n1C[C@H]1CC[C@H](C)CC1	3.0	8.52
	2015	CHEMBL3649822	C[C@H]1CC[C@H](Cn2c(N3CCOCC3c3ccccc3F)nc3cc(-c4noc(=O)[nH]4)nc(-c4cncc(Cl)c4)c32)CC1	8.0	8.10
	2015	CHEMBL3649824	CC1CCN(c2nc3cc(-c4noc(=O)[nH]4)nc(-c4cncc(Cl)c4)c3n2C[C@H]2CC[C@H](C)CC2)C1C	9.0	8.05
	2015	CHEMBL3649825	C[C@H]1CC[C@H](Cn2c(N3CCCC3C3CC3)nc3cc(-c4noc(=O)[nH]4)nc(-c4cncc(Cl)c4)c32)CC1	1.0	9.00
	2015	CHEMBL3649826	CC(C)(C)C1CCCN1c1nc2cc(-c3noc(=O)[nH]3)nc(-c3cncc(Cl)c3)c2n1C[C@H]1CC[C@H](C)CC1	3.0	8.52
	2015	CHEMBL3649827	C[C@H]1CC[C@H](Cn2c(N3CCCC34CCC4)nc3cc(-c4noc(=O)[nH]4)nc(-c4cncc(Cl)c4)c32)CC1	8.0	8.10
	2015	CHEMBL3649828	CCC(C)(C)C1CCCN1c1nc2cc(-c3noc(=O)[nH]3)nc(-c3cncc(Cl)c3)c2n1C[C@H]1CC[C@H](C)CC1	2.0	8.70

	2015	CHEMBL3916078	C[C@H]1CCCCN1c1nc2cc(-c3nc(=O)o[nH]3)nc(-c3cncc(Cl)c3)c2n1C[C@H]1CC[C@H](C)CC1	4.0	8.40
	2015	CHEMBL3938767	C[C@H]1C[C@H](O)CN1c1nc2cc(-c3nc(=O)o[nH]3)nc(-c3cncc(Cl)c3)c2n1C[C@H]1CC[C@H](C)CC1	6.0	8.22
	2015	CHEMBL3649829	Cc1nonc1C1CCCN1c1nc2cc(-c3noc(=O)[nH]3)nc(-c3cncc(Cl)c3)c2n1C[C@H]1CC[C@H](C)CC1	80.0	7.10
	2015	CHEMBL3942009	C[C@H]1CC[C@H](Cn2c(N3CCO[C@H]4CCC[C@@H]43)nc3cc(C(=O)O)nc(-c4cncc(Cl)c4)c32)CC1	2.0	8.70
	2015	CHEMBL3649833	C[C@H]1CC[C@H](Cn2c(N3CCC[C@H]3CF)nc3cc(C(=O)O)nc(-c4cncc(Cl)c4)c32)CC1	3.0	8.52
	2015	CHEMBL3649835	CO[C@@H]1C[C@@H](C)N(c2nc3cc(-c4noc(=O)[nH]4)nc(-c4cncc(Cl)c4)c3n2C[C@H]2CC[C@H](C)CC2)C1	4.0	8.40
	2015	CHEMBL3649834	CC1CN(c2nc3cc(-c4noc(=O)[nH]4)nc(-c4cncc(Cl)c4)c3n2C[C@H]2CC[C@H](C)CC2)C(c2ccccc2)CO1	16.0	7.80
	2015	CHEMBL3649837	CN(C)C(=O)[C@H]1CCCN1c1nc2cc(-c3noc(=O)[nH]3)nc(-c3cncc(Cl)c3)c2n1C[C@H]1CC[C@H](C)CC1	54.0	7.27
	2015	CHEMBL3649838	CCN(C)C(=O)[C@H]1CCCN1c1nc2cc(-c3noc(=O)[nH]3)nc(-c3cncc(Cl)c3)c2n1C[C@H]1CC[C@H](C)CC1	35.0	7.46
	2015	CHEMBL3649839	CCc1noc(C)c1C1CCCN1c1nc2cc(-c3noc(=O)[nH]3)nc(-c3cncc(Cl)c3)c2n1C[C@H]1CC[C@H](C)CC1	17.0	7.77
	2015	CHEMBL3649841	C[C@H]1CC[C@H](Cn2c(N3CCCC3COC(F)(F)F)nc3cc(-c4noc(=O)[nH]4)nc(-c4cncc(Cl)c4)c32)CC1	7.0	8.15
	2015	CHEMBL3649842	C[C@@H]1CCCN1c1nc2cc(-c3noc(=O)[nH]3)nc(-c3cncc(Cl)c3)c2n1C[C@H]1CC[C@H](C)CC1	4.0	8.40
	2015	CHEMBL3649843	COC[C@@H]1CCCN1c1nc2cc(-c3noc(=O)[nH]3)nc(-c3cncc(Cl)c3)c2n1C[C@H]1CC[C@H](C)CC1	3.0	8.52
	2015	CHEMBL3649840	CC(C)OCC1CCCN1c1nc2cc(-c3noc(=O)[nH]3)nc(-c3cncc(Cl)c3)c2n1C[C@H]1CC[C@H](C)CC1	47.0	7.33

	2015	CHEMBL3649844	C[C@H]1CC[C@H](Cn2c(N3CCC[C@H]3C(F)F)nc3cc(-c4noc(=O)[nH]4)nc(-c4cncc(Cl)c4)c32)CC1	1.0	9.00
	2015	CHEMBL3649846	C[C@H]1CC[C@H](Cn2c(N3CCCC34CCOC4)nc3cc(-c4noc(=O)[nH]4)nc(-c4cncc(Cl)c4)c32)CC1	6.0	8.22
	2015	CHEMBL3649847	C[C@H]1CC[C@H](Cn2c(N3CCCC34CCOCC4)nc3cc(-c4noc(=O)[nH]4)nc(-c4cncc(Cl)c4)c32)CC1	3.0	8.52
	2015	CHEMBL3649845	CC1OCCN(c2nc3cc(-c4noc(=O)[nH]4)nc(-c4cncc(Cl)c4)c3n2C[C@H]2CC[C@H](C)CC2)C1C	25.0	7.60
	2015	CHEMBL3649849	C[C@H]1CC[C@H](Cn2c(N3CCCC3c3cnn[nH]3)nc3cc(-c4noc(=O)[nH]4)nc(-c4cncc(Cl)c4)c32)CC1	1.0	9.00
	2015	CHEMBL3649850	Cn1cnc(C2CCCN2c2nc3cc(-c4noc(=O)[nH]4)nc(-c4cncc(Cl)c4)c3n2C[C@H]2CC[C@H](C)CC2)n1	12.0	7.92
	2015	CHEMBL3649851	Cn1ncnc1C1CCCN1c1nc2cc(-c3noc(=O)[nH]3)nc(-c3cncc(Cl)c3)c2n1C[C@H]1CC[C@H](C)CC1	21.0	7.68
	2015	CHEMBL3654184	C[C@H]1CC[C@H](Cn2c(N3CCC[C@H]3c3nccs3)nc3cc(-c4noc(=O)[nH]4)nc(-c4cncc(Cl)c4)c32)CC1	1.0	9.00
	2015	CHEMBL3649853	Cc1cc(C2CCCN2c2nc3cc(-c4noc(=O)[nH]4)nc(-c4cncc(Cl)c4)c3n2C[C@H]2CC[C@H](C)CC2)on1	2.0	8.70
	2015	CHEMBL3649854	Cc1noc(C)c1C1CCCN1c1nc2cc(-c3noc(=O)[nH]3)nc(-c3cncc(Cl)c3)c2n1C[C@H]1CC[C@H](C)CC1	11.0	7.96
	2015	CHEMBL3649856	CCNC(=O)[C@H]1CCCN1c1nc2cc(-c3noc(=O)[nH]3)nc(-c3cncc(Cl)c3)c2n1C[C@H]1CC[C@H](C)CC1	144.0	6.84
	2015	CHEMBL3649857	C[C@@H]1CN(C)C(=O)CN1c1nc2cc(-c3noc(=O)[nH]3)nc(-c3cncc(Cl)c3)c2n1C[C@H]1CC[C@H](C)CC1	11.0	7.96
	2015	CHEMBL3649858	COC1CC[C@@H](C)N(c2nc3cc(-c4noc(=O)[nH]4)nc(-c4cncc(Cl)c4)c3n2C[C@H]2CC[C@H](C)CC2)C1	3.0	8.52
	2015	CHEMBL3649859	CC1CS(=O)(=O)CCN1c1nc2cc(-c3noc(=O)[nH]3)nc(-c3cncc(Cl)c3)c2n1C[C@H]1CC[C@H](C)CC1	8.0	8.10

	2015	CHEMBL3649861	CO[C@@H]1C[C@@H](C(C)C)N(c2nc3cc(-c4noc(=O)[nH]4)nc(-c4cncc(Cl)c4)c3n2C[C@H]2CC[C@H](C)CC2)C1	1.0	9.00
	2015	CHEMBL3654185	CC(C)[C@@H]1C[C@@H](O)CN1c1nc2cc(-c3noc(=O)[nH]3)nc(-c3cncc(Cl)c3)c2n1C[C@H]1CC[C@H](C)CC1	1.0	9.00
	2015	CHEMBL3649863	CO[C@@H]1C[C@@H](CF)N(c2nc3cc(-c4noc(=O)[nH]4)nc(-c4cncc(Cl)c4)c3n2C[C@H]2CC[C@H](C)CC2)C1	1.0	9.00
	2015	CHEMBL3649864	CO[C@@H]1C[C@H](CF)N(c2nc3cc(-c4noc(=O)[nH]4)nc(-c4cncc(Cl)c4)c3n2C[C@H]2CC[C@H](C)CC2)C1	353.0	6.45
	2015	CHEMBL3649865	CN(CC(F)(F)F)c1nc2cc(-c3noc(=O)[nH]3)nc(-c3cncc(Cl)c3)c2n1C[C@H]1CC[C@H](C)CC1	1.0	9.00
	2015	CHEMBL3982496	CO[C@H]1COC[C@@H]1N(C)c1nc2cc(-c3noc(=O)[nH]3)nc(-c3cncc(Cl)c3)c2n1CC1CCC(C)CC1	26.0	7.59
	2015	CHEMBL3649867	Cn1cc(C2CCCN2c2nc3cc(-c4noc(=O)[nH]4)nc(-c4cncc(Cl)c4)c3n2C[C@H]2CC[C@H](C)CC2)nn1	13.0	7.89
	2015	CHEMBL3654187	CCN(C)C(=O)[C@@H]1CCCN1c1nc2cc(-c3noc(=O)[nH]3)nc(-c3cncc(Cl)c3)c2n1C[C@H]1CC[C@H](C)CC1	7.0	8.15
	2015	CHEMBL3649869	C[C@H]1CC[C@H](Cn2c(Nc3ccccc3F)nc3cc(-c4noc(=O)[nH]4)nc(-c4cncc(Cl)c4)c32)CC1	1.0	9.00
	2015	CHEMBL3649870	CCn1nccc1C1CCCN1c1nc2cc(-c3noc(=O)[nH]3)nc(-c3cncc(Cl)c3)c2n1C[C@H]1CC[C@H](C)CC1	3.0	8.52
	2015	CHEMBL3649871	Cn1cc(C2CCCN2c2nc3cc(-c4noc(=O)[nH]4)nc(-c4cncc(Cl)c4)c3n2C[C@H]2CC[C@H](C)CC2)cn1	7.0	8.15
	2015	CHEMBL3649872	Cc1nc(C2CCCN2c2nc3cc(-c4noc(=O)[nH]4)nc(-c4cncc(Cl)c4)c3n2C[C@H]2CC[C@H](C)CC2)no1	1.0	9.00
	2015	CHEMBL3649855	Cc1noc(C2CCCN2c2nc3cc(-c4noc(=O)[nH]4)nc(-c4cncc(Cl)c4)c3n2C[C@H]2CC[C@H](C)CC2)n1	116.0	6.94
	2015	CHEMBL3649873	C[C@H]1CC[C@H](Cn2c(N3CCC34COC4)nc3cc(-c4noc(=O)[nH]4)nc(-c4cncc(Cl)c4)c32)CC1	19.0	7.72

	2015	CHEMBL3649874	COC(C)(C)CN(C)c1nc2cc(-c3noc(=O)[nH]3)nc(-c3cncc(Cl)c3)c2n1C[C@H]1CC[C@H](C)CC1	9.0	8.05
	2015	CHEMBL3649875	Cc1nc(C2CCCN2c2nc3cc(-c4noc(=O)[nH]4)nc(-c4cncc(Cl)c4)c3n2C[C@H]2CC[C@H](C)CC2)cs1	1.0	9.00
	2015	CHEMBL3649876	COCCN(C)c1nc2cc(-c3noc(=O)[nH]3)nc(-c3cncc(Cl)c3)c2n1C[C@H]1CC[C@H](C)CC1	41.0	7.39
	2015	CHEMBL3649877	CCN(CCOC)c1nc2cc(-c3noc(=O)[nH]3)nc(-c3cncc(Cl)c3)c2n1C[C@H]1CC[C@H](C)CC1	11.0	7.96
	2015	CHEMBL3649868	CN(C)C(=O)[C@@H]1CCCN1c1nc2cc(-c3noc(=O)[nH]3)nc(-c3cncc(Cl)c3)c2n1C[C@H]1CC[C@H](C)CC1	14.0	7.85
	2015	CHEMBL3649878	CCCN(CCOC)c1nc2cc(-c3noc(=O)[nH]3)nc(-c3cncc(Cl)c3)c2n1C[C@H]1CC[C@H](C)CC1	9.0	8.05
	2015	CHEMBL3649879	CC(Nc1nc2cc(-c3noc(=O)[nH]3)nc(-c3cncc(Cl)c3)c2n1C[C@H]1CC[C@H](C)CC1)c1ccccn1	2.0	8.70
	2015	CHEMBL3649880	CCNC(=O)[C@@H]1CCCN1c1nc2cc(-c3noc(=O)[nH]3)nc(-c3cncc(Cl)c3)c2n1C[C@H]1CC[C@H](C)CC1	50.0	7.30
	2015	CHEMBL3649881	COC[C@@H]1CC[C@@H](COC)N1c1nc2cc(-c3noc(=O)[nH]3)nc(-c3cncc(Cl)c3)c2n1C[C@H]1CC[C@H](C)CC1	2.0	8.70
	2015	CHEMBL3649883	CO[C@H]1CCN(c2nc3cc(-c4noc(=O)[nH]4)nc(-c4cncc(Cl)c4)c3n2C[C@H]2CC[C@H](C)CC2)[C@H](C)C1	18.0	7.74
	2015	CHEMBL3649882	CO[C@@H]1CCN(c2nc3cc(-c4noc(=O)[nH]4)nc(-c4cncc(Cl)c4)c3n2C[C@H]2CC[C@H](C)CC2)[C@H](C)C1	31.0	7.51
	2015	CHEMBL3649884	CN1C(=O)CN(c2nc3cc(-c4noc(=O)[nH]4)nc(-c4cncc(Cl)c4)c3n2C[C@H]2CC[C@H](C)CC2)[C@H]2CCC[C@@H]21	32.0	7.49
	2015	CHEMBL3649885	CCC1CN(C)C(=O)CN1c1nc2cc(-c3noc(=O)[nH]3)nc(-c3cncc(Cl)c3)c2n1C[C@H]1CC[C@H](C)CC1	25.0	7.60
	2015	CHEMBL3649848	C[C@H]1CC[C@H](Cn2c(N3CCCC4COCCC43)nc3cc(-c4noc(=O)[nH]4)nc(-c4cncc(Cl)c4)c32)CC1	8.0	8.10

	2015	CHEMBL3654188	C[C@H]1CC[C@H](Cn2c(N3CCO[C@@H]4CN(C(=O)OCc5ccccc5)CC[C@H]43)nc3cc(-c4noc(=O)[nH]4)nc(-c4cncc(Cl)c4)c32)CC1	250.0	6.60
	2015	CHEMBL3654189	C[C@H]1CC[C@H](Cn2c(N3CCO[C@@H]4CNCC[C@H]43)nc3cc(-c4noc(=O)[nH]4)nc(-c4cncc(Cl)c4)c32)CC1	512.0	6.29
	2015	CHEMBL3654190	C[C@H]1CC[C@H](Cn2c(N3CCO[C@@H]4CN(Cc5ccccc5)CC[C@H]43)nc3cc(-c4noc(=O)[nH]4)nc(-c4cncc(Cl)c4)c32)CC1	129.0	6.89
	2015	CHEMBL3649890	C[C@H]1CC[C@H](Cn2c(N3C4CCC3C(O)C4)nc3cc(-c4noc(=O)[nH]4)nc(-c4cncc(Cl)c4)c32)CC1	14.0	7.85
	2015	CHEMBL3649860	COC1CC2CCC1N2c1nc2cc(-c3noc(=O)[nH]3)nc(-c3cncc(Cl)c3)c2n1C[C@H]1CC[C@H](C)CC1	9.0	8.05
	2015	CHEMBL3649891	CC(C)[C@@H]1C[C@](C)(O)CN1c1nc2cc(-c3noc(=O)[nH]3)nc(-c3cncc(Cl)c3)c2n1C[C@H]1CC[C@H](C)CC1	1.0	9.00
	2015	CHEMBL3649892	COC[C@@H]1C[C@@H](OC)CN1c1nc2cc(-c3noc(=O)[nH]3)nc(-c3cncc(Cl)c3)c2n1C[C@H]1CC[C@H](C)CC1	3.0	8.52
	2015	CHEMBL3649893	C[C@H]1CC[C@H](Cn2c(N3CC(O)CC4CCCCC43)nc3cc(-c4noc(=O)[nH]4)nc(-c4cncc(Cl)c4)c32)CC1	5.0	8.30
	2015	CHEMBL3649894	CO[C@@H]1C[C@@H](C2(OC)CC2)N(c2nc3cc(-c4noc(=O)[nH]4)nc(-c4cncc(Cl)c4)c3n2C[C@H]2CC[C@H](C)CC2)C1	2.0	8.70
	2015	CHEMBL3649895	CO[C@@H]1C[C@H](C2(OC)CC2)N(c2nc3cc(-c4noc(=O)[nH]4)nc(-c4cncc(Cl)c4)c3n2C[C@H]2CC[C@H](C)CC2)C1	10.0	8.00
	2015	CHEMBL3649896	CC1CSCCN1c1nc2cc(-c3noc(=O)[nH]3)nc(-c3cncc(Cl)c3)c2n1C[C@H]1CC[C@H](C)CC1	9.0	8.05
	2015	CHEMBL3649897	CO[C@@H]1C[C@@H](C(F)F)N(c2nc3cc(-c4noc(=O)[nH]4)nc(-c4cncc(Cl)c4)c3n2C[C@H]2CC[C@H](C)CC2)C1	1.0	9.00
	2015	CHEMBL3649898	CCO[C@@H]1C[C@@H](CF)N(c2nc3cc(-c4noc(=O)[nH]4)nc(-c4cncc(Cl)c4)c3n2C[C@H]2CC[C@H](C)CC2)C1	1.0	9.00
	2015	CHEMBL3649899	CCN1C(=O)CN(c2nc3cc(-c4noc(=O)[nH]4)nc(-c4cncc(Cl)c4)c3n2C[C@H]2CC[C@H](C)CC2)[C@@H]2CCC[C@H]21	4.0	8.40

	2015	CHEMBL3649900	COCCO[C@@H]1C[C@@H](C(C)C)N(c2nc3cc(-c4noc(=O)[nH]4)nc(-c4cncc(Cl)c4)c3n2C[C@H]2CC[C@H](C)CC2)C1	1.0	9.00
	2015	CHEMBL3904045	C[C@H]1CC[C@H](Cn2c(N3CCO[C@H]4CCC(F)(F)[C@@H]43)nc3cc(-c4nc(=O)o[nH]4)nc(-c4cncc(Cl)c4)c32)CC1	1.0	9.00
	2015	CHEMBL3649902	CC1CO[C@@H]2CCC[C@H]2N1c1nc2cc(-c3noc(=O)[nH]3)nc(-c3cncc(Cl)c3)c2n1C[C@H]1CC[C@H](C)CC1	1.0	9.00
	2015	CHEMBL3649903	CC[C@@H]1[C@@H](C)OCCN1c1nc2cc(-c3noc(=O)[nH]3)nc(-c3cncc(Cl)c3)c2n1C[C@H]1CC[C@H](C)CC1	1.0	9.00
	2015	CHEMBL3649904	C[C@H]1CC[C@H](Cn2c(N3CCO[C@@H]4CC(F)(F)C[C@H]43)nc3cc(-c4noc(=O)[nH]4)nc(-c4cncc(Cl)c4)c32)CC1	130.0	6.89
	2015	CHEMBL3649905	C[C@H]1CC[C@H](Cn2c(N3CCOC[C@H]3c3ccccn3)nc3cc(-c4noc(=O)[nH]4)nc(-c4cncc(Cl)c4)c32)CC1	1.0	9.00
	2015	CHEMBL3649906	C[C@H]1CC[C@H](Cn2c(N3CCOC4CCOCC43)nc3cc(-c4noc(=O)[nH]4)nc(-c4cncc(Cl)c4)c32)CC1	21.0	7.68
	2015	CHEMBL3639487	CC1CN(c2nc3cc(-c4noc(=O)[nH]4)nc(-c4cncc(Cl)c4)c3n2C[C@H]2CC[C@H](C)CC2)C2COCCC2O1	63.0	7.20
	2015	CHEMBL3649907	C[C@H]1CC[C@H](Cn2c(N3CCOC4COCC43)nc3cc(-c4noc(=O)[nH]4)nc(-c4cncc(Cl)c4)c32)CC1	131.0	6.88
	2015	CHEMBL3649908	C[C@H]1CC[C@H](Cn2c(N3CCO[C@@H]4CC(F)C[C@H]43)nc3cc(-c4noc(=O)[nH]4)nc(-c4cncc(Cl)c4)c32)CC1	48.0	7.32
	2015	CHEMBL3649909	C[C@H]1CC[C@H](Cn2c(N3CCCOC4CCCC43)nc3cc(-c4noc(=O)[nH]4)nc(-c4cncc(Cl)c4)c32)CC1	1.0	9.00
	2015	CHEMBL3649910	CC1N(c2nc3cc(-c4noc(=O)[nH]4)nc(-c4cncc(Cl)c4)c3n2C[C@H]2CC[C@H](C)CC2)CCOC1(C)C	17.0	7.77
	2015	CHEMBL3649911	CC1CN(c2nc3cc(-c4noc(=O)[nH]4)nc(-c4cncc(Cl)c4)c3n2C[C@H]2CC[C@H](C)CC2)C2CCCCC2O1	3.0	8.52
	2015	CHEMBL3649912	C[C@H]1CC[C@H](Cn2c(NCc3ccccc3)nc3cc(-c4noc(=O)[nH]4)nc(-c4cncc(Cl)c4)c32)CC1	5.0	8.30

	2015	CHEMBL3649913	COCC(C)Nc1nc2cc(-c3noc(=O)[nH]3)nc(-c3cncc(Cl)c3)c2n1C[C@H]1CC[C@H](C)CC1	14.0	7.85
	2015	CHEMBL3649914	CN(Cc1ccccc1)c1nc2cc(-c3noc(=O)[nH]3)nc(-c3cncc(Cl)c3)c2n1C[C@H]1CC[C@H](C)CC1	19.0	7.72
	2015	CHEMBL3649915	CC1CN(c2nc3cc(-c4noc(=O)[nH]4)nc(-c4cncc(Cl)c4)c3n2C[C@H]2CC[C@H](C)CC2)C2CCCC2O1	1.0	9.00
	2015	CHEMBL3933957	CC1CN(c2nc3cc(-c4nc(=O)o[nH]4)nc(-c4cncc(Cl)c4)c3n2C[C@H]2CC[C@H](C)CC2)[C@@H](C)[C@H](C)O1	1.0	9.00
	2015	CHEMBL3649917	COCC(C)N(C)c1nc2cc(-c3noc(=O)[nH]3)nc(-c3cncc(Cl)c3)c2n1C[C@H]1CC[C@H](C)CC1	42.0	7.38
	2015	CHEMBL3649918	C[C@H]1CC[C@H](Cn2c(Nc3ccccn3)nc3cc(-c4noc(=O)[nH]4)nc(-c4cncc(Cl)c4)c32)CC1	23.0	7.64
	2015	CHEMBL3649919	C[C@H]1CC[C@H](Cn2c(Nc3ccccn3)nc3cc(C(=O)O)nc(-c4cncc(Cl)c4)c32)CC1	40.0	7.40
	2015	CHEMBL3649920	CN(c1ccccc1)c1nc2cc(-c3noc(=O)[nH]3)nc(-c3cncc(Cl)c3)c2n1C[C@H]1CC[C@H](C)CC1	132.0	6.88
	2015	CHEMBL3649921	CN(c1ccccc1)c1nc2cc(C(=O)O)nc(-c3cncc(Cl)c3)c2n1C[C@H]1CC[C@H](C)CC1	86.0	7.07
	2015	CHEMBL3649922	C[C@H]1CC[C@H](Cn2c(Nc3ccccc3)nc3cc(-c4noc(=O)[nH]4)nc(-c4cncc(Cl)c4)c32)CC1	18.0	7.74
	2015	CHEMBL3649923	CC([C@H]1CC[C@H](C)CC1)n1c(N2CCO[C@@H]3CCC[C@H]32)nc2cc(-c3noc(=O)[nH]3)nc(-c3cncc(Cl)c3)c21	1.0	9.00
	2015	CHEMBL3649924	CC([C@H]1CC[C@H](C)CC1)n1c(N2CCOC[C@H]2C)nc2cc(-c3noc(=O)[nH]3)nc(-c3cncc(Cl)c3)c21	1.0	9.00
	2015	CHEMBL3649925	CC([C@H]1CC[C@H](C)CC1)n1c(N2CC(=O)N(C)C[C@H]2C)nc2cc(-c3noc(=O)[nH]3)nc(-c3cncc(Cl)c3)c21	13.0	7.89
	2015	CHEMBL3649926	CO[C@@H]1C[C@@H](CF)N(c2nc3cc(-c4noc(=O)[nH]4)nc(-c4cncc(Cl)c4)c3n2C(C)[C@H]2CC[C@H](C)CC2)C1	1.0	9.00

	2015	CHEMBL3949967	CC(C)[C@H]1C[C@H](O)CN1c1nc2cc(-c3nc(=O)o[nH]3)nc(-c3cncc(Cl)c3)c2n1C(C)[C@H]1CC[C@H](C)CC1	1.0	9.00
	2015	CHEMBL3945283	CC([C@H]1CC[C@H](C)CC1)n1c(N2C[C@@H](O)C[C@@H]2C)nc2cc(-c3nc(=O)o[nH]3)nc(-c3cncc(Cl)c3)c21	3.0	8.52
	2015	CHEMBL3894245	CO[C@H]1C[C@H](C)N(c2nc3cc(-c4nc(=O)o[nH]4)nc(-c4cncc(Cl)c4)c3n2C(C)[C@H]2CC[C@H](C)CC2)C1	2.0	8.70
	2015	CHEMBL3907671	CC(=O)N1CCN(c2nc3cc(-c4nc(=O)o[nH]4)nc(-c4cncc(Cl)c4)c3n2C[C@H]2CC[C@H](C)CC2)[C@@H](C)C1	6.0	8.22
	2015	CHEMBL3978215	C[C@H]1CN(C(=O)C2CC2)CCN1c1nc2cc(-c3nc(=O)o[nH]3)nc(-c3cncc(Cl)c3)c2n1C[C@H]1CC[C@H](C)CC1	5.0	8.30
	2015	CHEMBL3654191	CC(=O)N1C[C@@H](C)N(c2nc3cc(-c4noc(=O)[nH]4)nc(-c4cncc(Cl)c4)c3n2C[C@H]2CC[C@H](C)CC2)[C@H](C)C1	1.0	9.00
	2015	CHEMBL3931299	C[C@H]1CN(C(=O)C2CC2)C[C@H](C)N1c1nc2cc(-c3nc(=O)o[nH]3)nc(-c3cncc(Cl)c3)c2n1C[C@H]1CC[C@H](C)CC1	1.0	9.00
	2015	CHEMBL3649933	C[C@@H]1CN(S(C)(=O)=O)C[C@@H](C)N1c1nc2cc(-c3noc(=O)[nH]3)nc(-c3cncc(Cl)c3)c2n1C[C@H]1CC[C@H](C)CC1	3.0	8.52
	2015	CHEMBL3649934	CCOC(=O)N1C[C@@H](C)N(c2nc3cc(-c4noc(=O)[nH]4)nc(-c4cncc(Cl)c4)c3n2C[C@H]2CC[C@H](C)CC2)[C@H](C)C1	2.0	8.70
	2015	CHEMBL3649935	CCNC(=O)N1C[C@@H](C)N(c2nc3cc(-c4noc(=O)[nH]4)nc(-c4cncc(Cl)c4)c3n2C[C@H]2CC[C@H](C)CC2)[C@H](C)C1	3.0	8.52
	2015	CHEMBL3649936	CCC(=O)N1C[C@@H](C)N(c2nc3cc(-c4noc(=O)[nH]4)nc(-c4cncc(Cl)c4)c3n2C[C@H]2CC[C@H](C)CC2)[C@H](C)C1	3.0	8.52
	2015	CHEMBL3649937	C[C@@H]1CN(C(=O)C2CCC2)C[C@@H](C)N1c1nc2cc(-c3noc(=O)[nH]3)nc(-c3cncc(Cl)c3)c2n1C[C@H]1CC[C@H](C)CC1	1.0	9.00
	2015	CHEMBL3949194	CCCC(=O)N1C[C@H](C)N(c2nc3cc(-c4nc(=O)o[nH]4)nc(-c4cncc(Cl)c4)c3n2C[C@H]2CC[C@H](C)CC2)[C@@H](C)C1	3.0	8.52
	2015	CHEMBL3968776	COC(=O)N1C[C@H](C)N(c2nc3cc(-c4nc(=O)o[nH]4)nc(-c4cncc(Cl)c4)c3n2C[C@H]2CC[C@H](C)CC2)[C@@H](C)C1	2.0	8.70

	2015	CHEM BL394 2694	CC(C)OC(=O)N1C[C@H](C)N(c2nc3cc(-c4nc(=O)o[nH]4)nc(-c4cncc(Cl)c4)c3n2C[C@H]2CC[C@H](C)CC2)[C@@H](C)C1	3.0	8.52
	2015	CHEM BL396 6104	CCCNC(=O)N1C[C@H](C)N(c2nc3cc(-c4nc(=O)o[nH]4)nc(-c4cncc(Cl)c4)c3n2C[C@H]2CC[C@H](C)CC2)[C@@H](C)C1	2.0	8.70
	2015	CHEM BL389 4749	CC(C)NC(=O)N1C[C@H](C)N(c2nc3cc(-c4nc(=O)o[nH]4)nc(-c4cncc(Cl)c4)c3n2C[C@H]2CC[C@H](C)CC2)[C@@H](C)C1	3.0	8.52
	2015	CHEM BL364 9943	CC(=O)N1CCN(c2nc3cc(-c4noc(=O)[nH]4)nc(-c4cncc(Cl)c4)c3n2C[C@H]2CC[C@H](C)CC2)C(C)C1C	82.0	7.09
	2015	CHEM BL364 9944	CC1C(C)N(c2nc3cc(-c4noc(=O)[nH]4)nc(-c4cncc(Cl)c4)c3n2C[C@H]2CC[C@H](C)CC2)CCN1C(=O)C1CC1	68.0	7.17
	2015	CHEM BL364 9945	CC(C)C(=O)N1C[C@@H](C)N(c2nc3cc(-c4noc(=O)[nH]4)nc(-c4cncc(Cl)c4)c3n2C[C@H]2CC[C@H](C)CC2)[C@H](C)C1	3.0	8.52
	2015	CHEM BL364 9946	C[C@H]1CC[C@H](Cn2c(N3CCN(C(=O)C4CC4)[C@@H]4CCC[C@H]43)nc3cc(-c4noc(=O)[nH]4)nc(-c4cncc(Cl)c4)c32)CC1	5.0	8.30
	2015	CHEM BL364 9947	CC(=O)N1CCN(c2nc3cc(-c4noc(=O)[nH]4)nc(-c4cncc(Cl)c4)c3n2C[C@H]2CC[C@H](C)CC2)[C@@H]2CCC[C@H]21	4.0	8.40
	2015	CHEM BL364 9948	C[C@H]1CC[C@H](Cn2c(N3CCN(C(=O)C(F)F)[C@@H]4CCC[C@H]43)nc3cc(-c4noc(=O)[nH]4)nc(-c4cncc(Cl)c4)c32)CC1	3.0	8.52
	2015	CHEM BL364 9949	C[C@H]1CC[C@H](Cn2c(N3CCN(C(=O)C4CCC4)[C@@H]4CCC[C@H]43)nc3cc(-c4noc(=O)[nH]4)nc(-c4cncc(Cl)c4)c32)CC1	5.0	8.30
	2015	CHEM BL364 9950	C[C@@H]1CN(C(=O)N(C)C)C[C@@H](C)N1c1nc2cc(-c3noc(=O)[nH]3)nc(-c3cncc(Cl)c3)c2n1C[C@H]1CC[C@H](C)CC1	1.0	9.00
	2015	CHEM BL364 9951	CCN(C)C(=O)N1C[C@@H](C)N(c2nc3cc(-c4noc(=O)[nH]4)nc(-c4cncc(Cl)c4)c3n2C[C@H]2CC[C@H](C)CC2)[C@H](C)C1	1.0	9.00
	2015	CHEM BL391 1237	C[C@H]1CN(C(=O)C2(F)CC2)C[C@H](C)N1c1nc2cc(-c3nc(=O)o[nH]3)nc(-c3cncc(Cl)c3)c2n1C[C@H]1CC[C@H](C)CC1	1.0	9.00
	2015	CHEM BL394 8683	C[C@H]1CN(C(=O)C2CC2(F)F)C[C@H](C)N1c1nc2cc(-c3nc(=O)o[nH]3)nc(-c3cncc(Cl)c3)c2n1C[C@H]1CC[C@H](C)CC1	4.0	8.40

	2015	CHEMBL3923676	C[C@H]1CN(C(=O)C(F)F)C[C@H](C)N1c1nc2cc(-c3nc(=O)o[nH]3)nc(-c3cncc(Cl)c3)c2n1C[C@H]1CC[C@H](C)CC1	4.0	8.40
	2015	CHEMBL3952603	C[C@H]1CN(C(=O)C2(C)COC2)C[C@H](C)N1c1nc2cc(-c3nc(=O)o[nH]3)nc(-c3cncc(Cl)c3)c2n1C[C@H]1CC[C@H](C)CC1	11.0	7.96
	2015	CHEMBL3965113	C[C@H]1CN(C(=O)C2(C)CC2)C[C@H](C)N1c1nc2cc(-c3nc(=O)o[nH]3)nc(-c3cncc(Cl)c3)c2n1C[C@H]1CC[C@H](C)CC1	10.0	8.00
	2015	CHEMBL3933691	C[C@H]1CN(C(=O)C2COC2)C[C@H](C)N1c1nc2cc(-c3nc(=O)o[nH]3)nc(-c3cncc(Cl)c3)c2n1C[C@H]1CC[C@H](C)CC1	4.0	8.40
	2015	CHEMBL3930337	COCC(=O)N1C[C@H](C)N(c2nc3cc(-c4nc(=O)o[nH]4)nc(-c4cncc(Cl)c4)c3n2C[C@H]2CC[C@H](C)CC2)[C@@H](C)C1	10.0	8.00
	2015	CHEMBL3649959	CCOC(c1nc2cc(-c3noc(=O)[nH]3)nc(-c3cncc(Cl)c3)c2n1C[C@H]1CC[C@H](C)CC1)C1CCCC1	2.0	8.70
	2015	CHEMBL3654192	CCOC(c1nc2cc(-c3n[nH]c(=O)[nH]3)nc(-c3cncc(Cl)c3)c2n1C[C@H]1CC[C@H](C)CC1)C1CCCC1	3.0	8.52
	2015	CHEMBL3649960	CCOC(c1nc2cc(-c3n[nH]c(=O)o3)nc(-c3cncc(Cl)c3)c2n1C[C@H]1CC[C@H](C)CC1)C1CCCC1	10.0	8.00
	2015	CHEMBL3649961	CCOC(C)c1nc2cc(-c3noc(=O)[nH]3)nc(-c3cncc(Cl)c3)c2n1C[C@H]1CC[C@H](C)CC1	18.0	7.74
	2015	CHEMBL3649962	CCOC(CC)c1nc2cc(-c3noc(=O)[nH]3)nc(-c3cncc(Cl)c3)c2n1C[C@H]1CC[C@H](C)CC1	8.0	8.10
	2015	CHEMBL3649963	CCC(OCC(F)(F)F)c1nc2cc(-c3noc(=O)[nH]3)nc(-c3cncc(Cl)c3)c2n1C[C@H]1CC[C@H](C)CC1	2.0	8.70
	2015	CHEMBL3649964	CCCOC(CC)c1nc2cc(-c3noc(=O)[nH]3)nc(-c3cncc(Cl)c3)c2n1C[C@H]1CC[C@H](C)CC1	3.0	8.52
	2015	CHEMBL3649965	CCOC(CO)c1nc2cc(-c3noc(=O)[nH]3)nc(-c3cncc(Cl)c3)c2n1C[C@H]1CC[C@H](C)CC1	15.0	7.82
	2015	CHEMBL3649966	CCOC(COC)c1nc2cc(-c3noc(=O)[nH]3)nc(-c3cncc(Cl)c3)c2n1C[C@H]1CC[C@H](C)CC1	9.0	8.05

	2015	CHEMBL3649967	CCOC(c1nc2cc(-c3noc(=O)[nH]3)nc(-c3cncc(Cl)c3)c2n1C[C@H]1CC[C@H](C)CC1)C1CC1	4.0	8.40
	2015	CHEMBL3649968	CCOC(c1nc2cc(-c3noc(=O)[nH]3)nc(-c3cncc(Cl)c3)c2n1C[C@H]1CC[C@H](C)CC1)C1CCOCC1	3.0	8.52
	2015	CHEMBL3649969	COCCOC(c1nc2cc(-c3noc(=O)[nH]3)nc(-c3cncc(Cl)c3)c2n1C[C@H]1CC[C@H](C)CC1)C1CC1	7.0	8.15
	2015	CHEMBL3649970	CCOC(c1ccccc1)c1nc2cc(-c3noc(=O)[nH]3)nc(-c3cncc(Cl)c3)c2n1C[C@H]1CC[C@H](C)CC1	4.0	8.40
	2015	CHEMBL3649971	C[C@H]1CC[C@H](Cn2c(C(O)c3ccc(F)cc3F)nc3cc(-c4noc(=O)[nH]4)nc(-c4cncc(Cl)c4)c32)CC1	19.0	7.72
	2015	CHEMBL3649972	C[C@H]1CC[C@H](Cn2c(C(O)c3ccccc3F)nc3cc(-c4noc(=O)[nH]4)nc(-c4cncc(Cl)c4)c32)CC1	2.0	8.70
	2015	CHEMBL3649973	COCC(O)(c1ccccc1)c1nc2cc(-c3noc(=O)[nH]3)nc(-c3cncc(Cl)c3)c2n1C[C@H]1CC[C@H](C)CC1	5.0	8.30
	2015	CHEMBL3649975	CCOC(c1cccnc1)c1nc2cc(-c3noc(=O)[nH]3)nc(-c3cncc(Cl)c3)c2n1C[C@H]1CC[C@H](C)CC1	5.0	8.30
	2015	CHEMBL3649974	CCOC(c1ccccn1)c1nc2cc(-c3noc(=O)[nH]3)nc(-c3cncc(Cl)c3)c2n1C[C@H]1CC[C@H](C)CC1	2.0	8.70
	2015	CHEMBL3649977	CCOC(c1ccn(C)n1)c1nc2cc(-c3noc(=O)[nH]3)nc(-c3cncc(Cl)c3)c2n1C[C@H]1CC[C@H](C)CC1	3.0	8.52
	2015	CHEMBL3649976	CCOC(c1cscn1)c1nc2cc(-c3noc(=O)[nH]3)nc(-c3cncc(Cl)c3)c2n1C[C@H]1CC[C@H](C)CC1	7.0	8.15
	2015	CHEMBL3649978	C[C@H]1CC[C@H](Cn2c(C(OCC(F)(F)F)c3ccccn3)nc3cc(-c4noc(=O)[nH]4)nc(-c4cncc(Cl)c4)c32)CC1	3.0	8.52
	2015	CHEMBL3649979	C[C@H]1CC[C@H](Cn2c(C(=O)c3ccccc3F)nc3cc(-c4noc(=O)[nH]4)nc(-c4cncc(Cl)c4)c32)CC1	2.0	8.70
	2015	CHEMBL3649980	CC(F)(c1ccccc1F)c1nc2cc(-c3noc(=O)[nH]3)nc(-c3cncc(Cl)c3)c2n1C[C@H]1CC[C@H](C)CC1	20.0	7.70

	2015	CHEMBL3649981	CC(F)(c1ccc(F)cc1F)c1nc2cc(-c3noc(=O)[nH]3)nc(-c3cncc(Cl)c3)c2n1C[C@H]1CC[C@H](C)CC1	2.0	8.70
	2015	CHEMBL3649982	CC(F)(c1ncccc1F)c1nc2cc(-c3noc(=O)[nH]3)nc(-c3cncc(Cl)c3)c2n1C[C@H]1CC[C@H](C)CC1	1.0	9.00
	2015	CHEMBL3649983	CC(F)(c1ccncc1F)c1nc2cc(-c3noc(=O)[nH]3)nc(-c3cncc(Cl)c3)c2n1C[C@H]1CC[C@H](C)CC1	1.0	9.00
	2015	CHEMBL3649984	Cc1cccnc1C(C)(F)c1nc2cc(-c3noc(=O)[nH]3)nc(-c3cncc(Cl)c3)c2n1C[C@H]1CC[C@H](C)CC1	1.0	9.00
	2015	CHEMBL3649985	CC(F)(c1ccccn1)c1nc2cc(-c3noc(=O)[nH]3)nc(-c3cncc(Cl)c3)c2n1C[C@H]1CC[C@H](C)CC1	2.0	8.70
	2015	CHEMBL3649986	COCC(C)(F)c1nc2cc(-c3noc(=O)[nH]3)nc(-c3cncc(Cl)c3)c2n1C[C@H]1CC[C@H](C)CC1	21.0	7.68
	2015	CHEMBL3649987	CC(c1ncccc1F)c1nc2cc(-c3noc(=O)[nH]3)nc(-c3cncc(Cl)c3)c2n1C[C@H]1CC[C@H](C)CC1	4.0	8.40
	2015	CHEMBL3649988	C=C(c1ccccc1)c1nc2cc(-c3noc(=O)[nH]3)nc(-c3cncc(Cl)c3)c2n1C[C@H]1CC[C@H](C)CC1	4.0	8.40
	2015	CHEMBL3649989	COCC(c1ccccc1)c1nc2cc(-c3noc(=O)[nH]3)nc(-c3cncc(Cl)c3)c2n1C[C@H]1CC[C@H](C)CC1	2.0	8.70
	2015	CHEMBL3649990	C=C(CCC)c1nc2cc(-c3noc(=O)[nH]3)nc(-c3cncc(Cl)c3)c2n1C[C@H]1CC[C@H](C)CC1	2.0	8.70
	2015	CHEMBL3649991	CCCC(COC)c1nc2cc(-c3noc(=O)[nH]3)nc(-c3cncc(Cl)c3)c2n1C[C@H]1CC[C@H](C)CC1	3.0	8.52
	2015	CHEMBL3649992	C=C(C)c1nc2cc(-c3noc(=O)[nH]3)nc(-c3cncc(Cl)c3)c2n1C[C@H]1CC[C@H](C)CC1	7.0	8.15
	2015	CHEMBL3649993	COCC(C)c1nc2cc(-c3noc(=O)[nH]3)nc(-c3cncc(Cl)c3)c2n1C[C@H]1CC[C@H](C)CC1	17.0	7.77
	2015	CHEMBL3649994	CCOCC(C)c1nc2cc(-c3noc(=O)[nH]3)nc(-c3cncc(Cl)c3)c2n1C[C@H]1CC[C@H](C)CC1	8.0	8.10

	2015	CHEMBL3649995	CC(C)OCC(C)c1nc2cc(-c3noc(=O)[nH]3)nc(-c3cncc(Cl)c3)c2n1C[C@H]1CC[C@H](C)CC1	8.0	8.10
	2015	CHEMBL3649996	CCC(COC)c1nc2cc(-c3noc(=O)[nH]3)nc(-c3cncc(Cl)c3)c2n1C[C@H]1CC[C@H](C)CC1	6.0	8.22
	2015	CHEMBL3649997	COCC(COC)c1nc2cc(-c3noc(=O)[nH]3)nc(-c3cncc(Cl)c3)c2n1C[C@H]1CC[C@H](C)CC1	8.0	8.10
	2015	CHEMBL3649998	COCC(c1nc2cc(-c3noc(=O)[nH]3)nc(-c3cncc(Cl)c3)c2n1C[C@H]1CC[C@H](C)CC1)C(C)C	5.0	8.30
	2015	CHEMBL3649999	CC(C)c1ccnc(-c2nc3cc(-c4noc(=O)[nH]4)nc(-c4cncc(Cl)c4)c3n2C[C@H]2CC[C@H](C)CC2)c1	4.0	8.40
	2015	CHEMBL3650000	C[C@H]1CC[C@H](Cn2c(N3CCOC[C@H]3c3ccccc3)nc3c(Br)c(-c4noc(=O)[nH]4)nc(-c4cncc(Cl)c4)c32)CC1	2.0	8.70
	2015	CHEMBL3959078	C[C@H]1CC[C@H](Cn2c(N3CCO[C@H]4CCC[C@@H]43)nc3c(Cl)c(-c4nc(=O)o[nH]4)nc(-c4cncc(Cl)c4)c32)CC1	1.0	9.00
	2015	CHEMBL3941748	C[C@H]1CC[C@H](Cn2c(N3CCO[C@H]4CCC[C@@H]43)nc3c(F)c(-c4nc(=O)o[nH]4)nc(-c4cncc(Cl)c4)c32)CC1	1.0	9.00
	2015	CHEMBL3650003	COc1c(-c2noc(=O)[nH]2)nc(-c2cncc(Cl)c2)c2c1nc(N1CCO[C@@H]3CCC[C@H]31)n2C[C@H]1CC[C@H](C)CC1	1.0	9.00
	2015	CHEMBL3650004	Cc1c(-c2noc(=O)[nH]2)nc(-c2cncc(Cl)c2)c2c1nc(N1CCO[C@@H]3CCC[C@H]31)n2C[C@H]1CC[C@H](C)CC1	2.0	8.70
	2015	CHEMBL3650005	CN(C)c1c(C(=O)O)nc(-c2cncc(Cl)c2)c2c1nc(N1CCO[C@@H]3CCC[C@H]31)n2C[C@H]1CC[C@H](C)CC1	24.0	7.62
	2015	CHEMBL3639488	COCC(C)c1nc2cc(-c3noc(=O)[nH]3)nc(-c3cncc(Cl)c3)c2n1C(C)[C@H]1CC[C@H](C)CC1	161.0	6.79
	2015	CHEMBL3933428	CS(=O)(=O)NC(=O)c1cc2nc(N3CCO[C@H]4CCC[C@@H]43)n(C[C@H]3CC[C@H](C)CC3)c2c(-c2cncc(Cl)c2)n1	1.0	9.00
	2015	CHEMBL3650007	CN(C)S(=O)(=O)NC(=O)c1cc2nc(N3CCO[C@@H]4CCC[C@H]43)n(C[C@H]3CC[C@H](C)CC3)c2c(-c2cncc(Cl)c2)n1	2.0	8.70

	2015	CHEMBL3650008	CNC(=O)c1cc2nc(N3CCOC[C@H]3c3ccccc3)n(C[C@H]3CC[C@H](C)CC3)c2c(-c2cncc(Cl)c2)n1	160.0	6.80
	2015	CHEMBL3650009	CN(C)C(=O)c1cc2nc(N3CCOC[C@H]3c3ccccc3)n(C[C@H]3CC[C@H](C)CC3)c2c(-c2cncc(Cl)c2)n1	175.0	6.76
	2015	CHEMBL3650010	CS(=O)(=O)Nc1cc2nc(N3CCOC[C@H]3c3ccccc3)n(C[C@H]3CC[C@H](C)CC3)c2c(-c2cncc(Cl)c2)n1	122.0	6.91
	2015	CHEMBL3650011	C[C@H]1CC[C@H](Cn2c(N3CCC[C@H]3C(F)(F)F)nc3cc(-c4nnn[nH]4)nc(-c4cncc(Cl)c4)c32)CC1	1.0	9.00
	2015	CHEMBL3650012	Cn1[nH]c(-c2cc3nc(N4CCC[C@H]4CF)n(C[C@H]4CC[C@H](C)CC4)c3c(-c3cncc(Cl)c3)n2)nc1=O	20.0	7.70
	2015	CHEMBL3650013	C[C@H]1CC[C@H](Cn2c(N3CCC[C@H]3CF)nc3cc(-c4n[nH]c(=O)[nH]4)nc(-c4cncc(Cl)c4)c32)CC1	10.0	8.00
	2015	CHEMBL3952705	C[C@H]1CC[C@H](Cn2c(N3CCO[C@H]4CCC[C@@H]43)nc3cc(-c4nn[nH]n4)nc(-c4cncc(Cl)c4)c32)CC1	1.0	9.00
	2015	CHEMBL3650015	COC(=O)c1cc2nc(N3CCOC[C@H]3c3ccccc3)n(C[C@H]3CC[C@H](C)CC3)c2c(-c2cncc(Cl)c2)n1	28.0	7.55
	2015	CHEMBL3654193	CCOC(=O)c1cc2nc(N3CCOC[C@H]3c3ccccc3)n(C[C@H]3CC[C@H](C)CC3)c2c(-c2cncc(Cl)c2)n1	53.0	7.28
	2015	CHEMBL3650018	CCOC(c1nc2cc(-c3noc(=O)[nH]3)nc(-c3cncc(Cl)c3)c2n1C(C)[C@H]1CC[C@H](C)CC1)C1CC1	4.0	8.40
	2015	CHEMBL3650019	CCOC(C)c1nc2cc(-c3noc(=O)[nH]3)nc(-c3cncc(Cl)c3)c2n1C(C)[C@H]1CC[C@H](C)CC1	5.0	8.30
	2015	CHEMBL3650020	CCOC(COC)c1nc2cc(-c3noc(=O)[nH]3)nc(-c3cncc(Cl)c3)c2n1C(C)[C@H]1CC[C@H](C)CC1	3.0	8.52
	2015	CHEMBL3959820	CC([C@H]1CC[C@H](C)CC1)n1c(N2CCN(C(=O)C3(F)CC3)C[C@@H]2C)nc2cc(-c3nc(=O)o[nH]3)nc(-c3cncc(Cl)c3)c21	1.0	9.00
	2015	CHEMBL3923370	O=c1nc(-c2cc3nc(N4CCO[C@H]5CCC[C@@H]54)n(Cc4ccc(C(F)(F)F)cc4)c3c(-c3cncc(Cl)c3)n2)[nH]o1	39.0	7.41

	2015	CHEMBL3951089	CC[C@H]1CC[C@H](Cn2c(N3CCO[C@H]4CCC[C@@H]43)nc3cc(-c4nc(=O)o[nH]4)nc(-c4cncc(Cl)c4)c32)CC1	5.0	8.30
	2015	CHEMBL3916778	CC[C@H]1CC[C@H](Cn2c(N3CCO[C@H]4CCC[C@@H]43)nc3cc(-c4nnc(O)o4)nc(-c4cncc(Cl)c4)c32)CC1	25.0	7.60
	2015	CHEMBL3941220	O=c1nc(-c2cc3nc(N4CCO[C@H]5CCC[C@@H]54)n(Cc4ccc(C(F)(F)F)c(F)c4)c3c(-c3cncc(Cl)c3)n2)[nH]o1	28.0	7.55
	2015	CHEMBL3653312	O=c1[nH]c(-c2cc3nc(N4CCO[C@@H]5CCC[C@H]54)n(C[C@H]4CC[C@H](C(F)(F)F)CC4)c3c(-c3cncc(Cl)c3)n2)no1	38.0	7.42
	2015	CHEMBL3653313	CCC1CCC(Cn2c(N3CCO[C@@H]4CCC[C@H]43)nc3cc(-c4noc(=O)[nH]4)nc(-c4cncc(Cl)c4)c32)C1	16.0	7.80
	2015	CHEMBL3653314	CN(C)c1ncc(Cl)cc1-c1nc(-c2noc(=O)[nH]2)cc2nc(N3CCO[C@@H]4CCC[C@H]43)n(C[C@H]3CC[C@H](C)CC3)c12	2.0	8.70
	2015	CHEMBL3653315	Cc1ncc(Cl)cc1-c1nc(-c2noc(=O)[nH]2)cc2nc(N3CCO[C@H]4CCC[C@@H]43)n(C[C@H]3CC[C@H](C)CC3)c12	33.0	7.48
	2015	CHEMBL3653316	Cc1ncc(Cl)cc1-c1nc(-c2noc(=O)[nH]2)cc2nc(N3CCO[C@@H]4CCC[C@H]43)n(C[C@H]3CC[C@H](C)CC3)c12	1.0	9.00
	2015	CHEMBL3653317	C[C@H]1CC[C@H](Cn2c(N3CCO[C@@H]4CCC[C@H]43)nc3cc(-c4noc(=O)[nH]4)nc(-c4cc(Cl)cnc4OCCO)c32)CC1	1.0	9.00
	2015	CHEMBL3653318	COc1ncc(Cl)cc1-c1nc(-c2noc(=O)[nH]2)cc2nc(N3CCO[C@H]4CCC[C@@H]43)n(C[C@H]3CC[C@H](C)CC3)c12	24.0	7.62
	2015	CHEMBL3653319	COc1ncc(Cl)cc1-c1nc(-c2noc(=O)[nH]2)cc2nc(N3CCO[C@@H]4CCC[C@H]43)n(C[C@H]3CC[C@H](C)CC3)c12	1.0	9.00
	2015	CHEMBL3653320	COCCOc1ncc(Cl)cc1-c1nc(-c2noc(=O)[nH]2)cc2nc(N3CCO[C@@H]4CCC[C@H]43)n(C[C@H]3CC[C@H](C)CC3)c12	1.0	9.00
	2015	CHEMBL3653321	COCCOc1ncc(Cl)cc1-c1nc(-c2noc(=O)[nH]2)cc2nc(N3CCO[C@H]4CCC[C@@H]43)n(C[C@H]3CC[C@H](C)CC3)c12	21.0	7.68
	2015	CHEMBL3653322	C[C@H]1CC[C@H](Cn2c(N3CCO[C@@H]4CCC[C@H]43)nc3cc(-c4noc(=O)[nH]4)nc(-c4cc(Cl)c[nH]c4=O)c32)CC1	2.0	8.70

	2015	CHEMBL3653323	Cc1cncc(-c2nc(-c3noc(=O)[nH]3)cc3nc(N4CCO[C@@H]5CCC[C@H]54)n(C[C@H]4CC[C@H](C)CC4)c23)c1	2.0	8.70
	2015	CHEMBL3653324	Cc1cncc(-c2nc(-c3noc(=O)[nH]3)cc3nc(N4CCO[C@H]5CCC[C@@H]54)n(C[C@H]4CC[C@H](C)CC4)c23)c1	210.0	6.68
	2015	CHEMBL3971741	CNc1ncc(Cl)cc1-c1nc(-c2nc(=O)o[nH]2)cc2nc(N3CCO[C@H]4CCC[C@@H]43)n(C[C@H]3CC[C@H](C)CC3)c12	2.0	8.70
	2015	CHEMBL3653326	C[C@H]1CC[C@H](Cn2c(N3CCOC[C@H]3c3ccccc3)nc3cc(-c4noc(=O)[nH]4)nc(-c4cc(Cl)c[nH]c4=O)c32)CC1	1.0	9.00
	2015	CHEMBL3653327	C[C@H]1CC[C@H](Cn2c(N3CCOC[C@H]3c3ccccc3)nc3cc(-c4noc(=O)[nH]4)nc(-c4cncnc4)c32)CC1	65.0	7.19
	2015	CHEMBL3653328	C[C@H]1CC[C@H](Cn2c(N3CCOC[C@H]3c3ccccc3)nc3cc(-c4noc(=O)[nH]4)nc(-c4cnccn4)c32)CC1	119.0	6.92
	2015	CHEMBL3653329	C[C@H]1CC[C@H](Cn2c(N3CCOC[C@H]3c3ccccc3)nc3cc(-c4noc(=O)[nH]4)nc(-c4ccnnc4)c32)CC1	72.0	7.14
	2015	CHEMBL3653330	C[C@H]1CC[C@H](Cn2c(N3CCOC[C@H]3c3ccccc3)nc3cc(-c4noc(=O)[nH]4)nc(-c4cccnn4)c32)CC1	112.0	6.95
	2015	CHEMBL3653331	CSCC(C)c1nc2cc(-c3noc(=O)[nH]3)nc(-c3cncc(Cl)c3)c2n1C[C@H]1CC[C@H](C)CC1	7.0	8.15
	2015	CHEMBL3653332	CC(CS(C)(=O)=O)c1nc2cc(-c3noc(=O)[nH]3)nc(-c3cncc(Cl)c3)c2n1C[C@H]1CC[C@H](C)CC1	37.0	7.43
	2015	CHEMBL3653333	CCC(CSC)c1nc2cc(-c3noc(=O)[nH]3)nc(-c3cncc(Cl)c3)c2n1C[C@H]1CC[C@H](C)CC1	2.0	8.70
	2015	CHEMBL3653334	CCC(CS(C)(=O)=O)c1nc2cc(-c3noc(=O)[nH]3)nc(-c3cncc(Cl)c3)c2n1C[C@H]1CC[C@H](C)CC1	7.0	8.15
	2015	CHEMBL3653335	CC(C)C(CS(C)(=O)=O)c1nc2cc(-c3noc(=O)[nH]3)nc(-c3cncc(Cl)c3)c2n1C[C@H]1CC[C@H](C)CC1	5.0	8.30
	2015	CHEMBL3654194	C[C@H]1CC[C@H](Cn2c(N3CCOC[C@H]3c3ccccc3)nc3cc(-c4cccc(=O)[nH]4)nc(-c4cncc(Cl)c4)c32)CC1	70.0	7.15

	2015	CHEMBL3653337	C[C@H]1CC[C@H](Cn2c(N3CCOC[C@H]3c3ccccc3)nc3cc(-c4noc(=O)[nH]4)nc(OCC4CCC4)c32)CC1	37.0	7.43
	2015	CHEMBL3653338	CC(Oc1nc(-c2noc(=O)[nH]2)cc2nc(N3CCOC[C@H]3c3ccccc3)n(C[C@H]3CC[C@H](C)CC3)c12)C1CCC1	28.0	7.55
	2015	CHEMBL3703317	COCCN(CCOC)C(=O)OC(C)OC(=O)c1ccc(NC(=O)[C@@H]2N[C@@H](CC(C)(C)C)[C@](C#N)(c3ccc(Cl)cc3F)[C@H]2c2cccc(Cl)c2F)c(OC)c1	16.3	7.79
	2015	CHEMBL3703318	COc1cc(C(=O)OC(C)OC(=O)N2CCN(C)CC2)ccc1NC(=O)[C@@H]1N[C@@H](CC(C)(C)C)[C@](C#N)(c2ccc(Cl)cc2F)[C@H]1c1cccc(Cl)c1F	18.7	7.73
	2015	CHEMBL3703319	COc1cc(C(=O)OC(C)OC(C)=O)ccc1NC(=O)[C@@H]1N[C@@H](CC(C)(C)C)[C@](C#N)(c2ccc(Cl)cc2F)[C@H]1c1cccc(Cl)c1F	10.4	7.98
	2015	CHEMBL3703321	COc1cc(C(=O)OCOC(C)=O)ccc1NC(=O)[C@@H]1N[C@@H](CC(C)(C)C)[C@](C#N)(c2ccc(Cl)cc2F)[C@H]1c1cccc(Cl)c1F	45.0	7.35
	2015	CHEMBL3703324	COc1cc(C(=O)OC(C)OC(=O)N2CCOCC2)ccc1NC(=O)[C@@H]1N[C@@H](CC(C)(C)C)[C@](C#N)(c2ccc(Cl)cc2F)[C@H]1c1cccc(Cl)c1F	53.6	7.27
	2015	CHEMBL3703325	COc1cc(C(=O)O[C@@H](C)OC(=O)N2CCOCC2)ccc1NC(=O)[C@@H]1N[C@@H](CC(C)(C)C)[C@](C#N)(c2ccc(Cl)cc2F)[C@H]1c1cccc(Cl)c1F	10.1	8.00
	2015	CHEMBL3703360	COc1cc(C(=O)O[C@H](C)OC(=O)N2CCOCC2)ccc1NC(=O)[C@@H]1N[C@@H](CC(C)(C)C)[C@](C#N)(c2ccc(Cl)cc2F)[C@H]1c1cccc(Cl)c1F	19.4	7.71
	2015	CHEMBL3703327	COc1cc(C(=O)OC(C)OC(=O)N2CCNCC2)ccc1NC(=O)[C@@H]1N[C@@H](CC(C)(C)C)[C@](C#N)(c2ccc(Cl)cc2F)[C@H]1c1cccc(Cl)c1F	14.2	7.85
	2015	CHEMBL3703328	COc1cc(C(=O)OC(C)OC(=O)N2CCS(=O)(=O)CC2)ccc1NC(=O)[C@@H]1N[C@@H](CC(C)(C)C)[C@](C#N)(c2ccc(Cl)cc2F)[C@H]1c1cccc(Cl)c1F	12.0	7.92
	2015	CHEMBL3703330	COc1cc(C(=O)OC(C)OC(=O)NCC(O)CO)ccc1NC(=O)[C@@H]1N[C@@H](CC(C)(C)C)[C@](C#N)(c2ccc(Cl)cc2F)[C@H]1c1cccc(Cl)c1F	13.4	7.87
	2015	CHEMBL3703338	COc1cc(C(=O)OC(C)OC(=O)NCC(=O)O)ccc1NC(=O)[C@@H]1N[C@@H](CC(C)(C)C)[C@](C#N)(c2ccc(Cl)cc2F)[C@H]1c1cccc(Cl)c1F	4.4	8.36
	2015	CHEMBL3703340	COc1cc(C(=O)OCOC(=O)NCC(=O)O)ccc1NC(=O)[C@@H]1N[C@@H](CC(C)(C)C)[C@](C#N)(c2ccc(Cl)cc2F)[C@H]1c1cccc(Cl)c1F	8.2	8.09

	2015	CHEMBL3703342	COc1cc(C(=O)OCOC(=O)N[C@@H](CCC(=O)O)C(=O)O)ccc1NC(=O)[C@@H]1N[C@@H](CC(C)(C)C)[C@](C#N)(c2ccc(Cl)cc2F)[C@H]1c1cccc(Cl)c1F	162.0	6.79
	2015	CHEMBL3703344	COCCOCCOCCOC(=O)OCOC(=O)c1ccc(NC(=O)[C@@H]2N[C@@H](CC(C)(C)C)[C@](C#N)(c3ccc(Cl)cc3F)[C@H]2c2cccc(Cl)c2F)c(OC)c1	9.1	8.04
	2015	CHEMBL3703345	COCCOCCOCCOCCOCCOC(=O)OCOC(=O)c1ccc(NC(=O)[C@@H]2N[C@@H](CC(C)(C)C)[C@](C#N)(c3ccc(Cl)cc3F)[C@H]2c2cccc(Cl)c2F)c(OC)c1	5.3	8.28
	2015	CHEMBL3703346	COCCOCCOC(=O)OCOC(=O)c1ccc(NC(=O)[C@@H]2N[C@@H](CC(C)(C)C)[C@](C#N)(c3ccc(Cl)cc3F)[C@H]2c2cccc(Cl)c2F)c(OC)c1	4.2	8.38
	2015	CHEMBL3703347	COCCOCCOCCOCCOCCOCCOC(=O)OCOC(=O)c1ccc(NC(=O)[C@@H]2N[C@@H](CC(C)(C)C)[C@](C#N)(c3ccc(Cl)cc3F)[C@H]2c2cccc(Cl)c2F)c(OC)c1	5.1	8.30
	2015	CHEMBL3703350	COCCOCCNC(=O)OC(C)OC(=O)c1ccc(NC(=O)[C@@H]2N[C@@H](CC(C)(C)C)[C@](C#N)(c3ccc(Cl)cc3F)[C@H]2c2cccc(Cl)c2F)c(OC)c1	4.9	8.31
	2015	CHEMBL3703351	COCCOCCNC(=O)OCOC(=O)c1ccc(NC(=O)[C@@H]2N[C@@H](CC(C)(C)C)[C@](C#N)(c3ccc(Cl)cc3F)[C@H]2c2cccc(Cl)c2F)c(OC)c1	30.3	7.52
	2015	CHEMBL3703352	COc1cc(C(=O)OCOC(=O)N[C@@H](C)C(=O)O)ccc1NC(=O)[C@@H]1NC(CC(C)(C)C)[C@](C#N)(c2ccc(Cl)cc2F)C1c1cccc(Cl)c1F	7.0	8.16
	2015	CHEMBL3703354	COc1cc(C(=O)OCOC(=O)OCCOCCOCCOCCOP(=O)(O)O)ccc1NC(=O)[C@@H]1N[C@@H](CC(C)(C)C)[C@](C#N)(c2ccc(Cl)cc2F)[C@H]1c1cccc(Cl)c1F	26.5	7.58
	2015	CHEMBL3703355	COc1cc(C(=O)OCOP(=O)(O)O)ccc1NC(=O)[C@@H]1N[C@@H](CC(C)(C)C)[C@](C#N)(c2ccc(Cl)cc2F)[C@H]1c1cccc(Cl)c1F	5.1	8.29
	2015	CHEMBL3703357	COc1cc(C(=O)OC(C)OC(=O)O[C@@H]2[C@@H](O)[C@@H](O)O[C@@H]2[C@H](O)CO)ccc1NC(=O)[C@@H]1N[C@@H](CC(C)(C)C)[C@](C#N)(c2ccc(Cl)cc2F)[C@H]1c1cccc(Cl)c1F	3.4	8.47
	2015	CHEMBL3703359	COc1cc(C(=O)OC(C)OC(=O)OCCOCCOCCOCCOP(=O)(O)O)ccc1NC(=O)[C@@H]1N[C@@H](CC(C)(C)C)[C@](C#N)(c2ccc(Cl)cc2F)[C@H]1c1cccc(Cl)c1F	4.8	8.32
	2015	CHEMBL3703646	COc1ccc(C(Nc2cc(C)ccn2)c2ccc3cccnc3c2O)cc1O	4200.0	5.38
	2015	CHEMBL3703676	Oc1c(C(Nc2cccnc2)c2cccs2)ccc2cccnc12	1500.0	5.82

	2015	CHEMBL548572	Oc1c(C(Nc2ccccn2)c2ccc(Cl)cc2Cl)ccc2cccnc12	7400.0	5.13
	2015	CHEMBL3703678	Cc1cccc(NC(c2ccc(C(F)(F)F)cc2)c2ccc3cccnc3c2O)n1	4000.0	5.40
	2015	CHEMBL2094874	Cc1ccnc(NC(c2ccc3cccnc3c2O)c2sccc2C)c1	2000.0	5.70
	2015	CHEMBL3703647	Cc1cccc(NC(c2ccc(OC(F)(F)F)cc2)c2ccc3cccnc3c2O)n1	9400.0	5.03
	2015	CHEMBL524251	Cc1ccnc(NC(c2cccc(Cl)c2Cl)c2ccc3cccnc3c2O)c1	4400.0	5.36
	2015	CHEMBL3703650	Cc1ccnc(NC(c2ccccc2Cl)c2ccc3cccnc3c2O)c1	9200.0	5.04
	2015	CHEMBL3703651	COc1ccc(C(Nc2cccc(C)n2)c2ccc3cccnc3c2O)cc1O	4200.0	5.38
	2015	CHEMBL3703652	Oc1c(C(Nc2ccccn2)c2cc(Cl)ccc2Cl)ccc2cccnc12	7500.0	5.12
	2015	CHEMBL3112895	Cc1ccnc(NC(c2ccc(Cl)c(Cl)c2)c2ccc3cccnc3c2O)c1	3600.0	5.44
	2015	CHEMBL3703653	Cc1cccc(NC(c2ccc3cccnc3c2O)c2c(F)c(F)cc(F)c2F)n1	5200.0	5.28
	2015	CHEMBL3703655	Oc1c(C(Nc2ccc(Cl)cn2)c2ccc(Cl)c(Cl)c2)ccc2cccnc12	9000.0	5.05
	2015	CHEMBL3703658	Oc1c(C(Nc2ccc(Cl)cn2)c2ccc(Cl)c(C(F)(F)F)c2)ccc2cccnc12	8000.0	5.10
	2015	CHEMBL3703659	Oc1c(C(Nc2ccc(F)cn2)c2c(F)c(F)cc(F)c2F)ccc2cccnc12	8000.0	5.10
	2015	CHEMBL3703660	Cc1cccc(NC(c2ccc(Cl)c(Cl)c2)c2ccc3ccc(C)nc3c2O)n1	6000.0	5.22

	2015	CHEMBL3703662	Oc1c(C(Nc2ccc(F)cn2)c2ccc(F)cc2Cl)ccc2cccnc12	10000.0	5.00
	2015	CHEMBL3640025	Oc1c(C(Nc2ccc(F)cn2)c2ccc(Cl)c(Cl)c2)ccc2cccnc12	9000.0	5.05
	2015	CHEMBL3703669	COc1ccc(C(Nc2cc(C)c(Cl)cn2)c2ccc3cccnc3c2O)c(OC)c1OC	10000.0	5.00
	2015	CHEMBL3703671	Cc1nc(NC(c2ccc(Cl)c(Cl)c2)c2ccc3cccnc3c2O)c(Cl)cc1Cl	10000.0	5.00
	2015	CHEMBL3704482	CC(C)(C)C[C@H]1N[C@@H](C(=O)NCC[C@H](O)C=O)[C@H](c2cccc(Cl)c2)[C@@]12C(=O)Nc1cc(Cl)c(F)cc12	1000.0	6.00
	2015	CHEMBL3704483	CC(C)(C)C[C@H]1N[C@@H](C(=O)NCCC(=O)CO)[C@H](c2cccc(Cl)c2)[C@@]12C(=O)Nc1cc(Cl)c(F)cc12	1000.0	6.00
	2015	CHEMBL3704484	CC(C)(C)C[C@H]1N[C@@H](C(=O)NCC[C@H](O)CNC(C)(C)C)[C@H](c2cccc(Cl)c2)[C@@]12C(=O)Nc1cc(Cl)c(F)cc12	2000.0	5.70
	2015	CHEMBL3704485	CC(C)(C)C[C@H]1N[C@@H](C(=O)N[C@H]2CC[C@@H](O)C2)[C@H](c2cccc(Cl)c2)[C@@]12C(=O)Nc1cc(Cl)c(F)cc12	1000.0	6.00
	2015	CHEMBL3704486	CC(C)(C)C[C@H]1N[C@@H](C(=O)NCC[C@H](O)CN2CCCC2)[C@H](c2cccc(Cl)c2)[C@@]12C(=O)Nc1cc(Cl)c(F)cc12	2000.0	5.70
	2015	CHEMBL3704487	CC(C)(C)C[C@H]1N[C@@H](C(=O)NCC[C@H](O)CN2CC[C@@H](O)C2)[C@H](c2cccc(Cl)c2)[C@@]12C(=O)Nc1cc(Cl)c(F)cc12	1000.0	6.00
	2015	CHEMBL3704488	CC(O)C(O)CCNC(=O)[C@@H]1N[C@H](CC(C)(C)C)[C@]2(C(=O)Nc3cc(Cl)c(F)cc32)[C@H]1c1cccc(Cl)c1	2000.0	5.70
	2015	CHEMBL3704489	CC(C)(C)C[C@H]1N[C@@H](C(=O)NCCN2CC[C@H](O)C2)[C@H](c2cccc(Cl)c2)[C@@]12C(=O)Nc1cc(Cl)c(F)cc12	1000.0	6.00
	2015	CHEMBL3704490	CC(C)(C)C[C@H]1N[C@@H](C(=O)NCC2CC(O)C(O)C2)[C@H](c2cccc(Cl)c2)[C@@]12C(=O)Nc1cc(Cl)c(F)cc12	1000.0	6.00

	2015	CHEMBL3704491	CC(C)(C)C[C@H]1N[C@@H](C(=O)NCCCN2CCCC2)[C@H](c2cccc(Cl)c2)[C@@]12C(=O)Nc1cc(Cl)c(F)cc12	1000.0	6.00
	2015	CHEMBL3704492	CNC(=O)[C@@H]1N[C@H](CC(C)(C)C)[C@]2(C(=O)Nc3cc(Cl)c(F)cc32)[C@H]1c1cccc(Cl)c1	1000.0	6.00
	2015	CHEMBL3704493	CC(C)(C)C[C@H]1N[C@@H](C(N)=O)[C@H](c2cccc(Cl)c2)[C@@]12C(=O)Nc1cc(Cl)c(F)cc12	1000.0	6.00
	2015	CHEMBL3704494	CC(C)(C)C[C@H]1N[C@@H](C(=O)NCCCN2CCCC2)[C@H](c2cccc(Cl)c2F)[C@@]12C(=O)Nc1cc(Cl)ccc12	10000.0	5.00
	2015	CHEMBL3704495	CC(C)(C)C[C@H]1N[C@@H](C(=O)NCCCN2CCCCC2)[C@H](c2cccc(Cl)c2F)[C@@]12C(=O)Nc1cc(Cl)ccc12	10000.0	5.00
	2015	CHEMBL3704496	CC(C)N1CCN(CCCNC(=O)[C@@H]2N[C@H](CC(C)(C)C)[C@]3(C(=O)Nc4cc(Cl)ccc43)[C@H]2c2cccc(Cl)c2F)CC1	10000.0	5.00
	2015	CHEMBL3704497	CN1CCN(CCCNC(=O)[C@@H]2N[C@H](CC(C)(C)C)[C@]3(C(=O)Nc4cc(Cl)ccc43)[C@H]2c2cccc(Cl)c2F)CC1	1000.0	6.00
	2015	CHEMBL3704498	CC(C)(C)C[C@H]1N[C@@H](C(=O)NCCCN2CCN(C3CC3)CC2)[C@H](c2cccc(Cl)c2F)[C@@]12C(=O)Nc1cc(Cl)ccc12	10000.0	5.00
	2015	CHEMBL3704500	CC(C)(C)C[C@H]1N[C@@H](C(N)=O)[C@H](c2cccc(Cl)c2F)[C@@]12C(=O)Nc1cc(Cl)ccc12	1000.0	6.00
	2015	CHEMBL3704501	CC(C)(C)C[C@H]1N[C@@H](C(=O)N[C@H]2CC[C@@H](O)C2)[C@H](c2cccc(Cl)c2F)[C@@]12C(=O)Nc1cc(Cl)ccc12	1000.0	6.00
	2015	CHEMBL3704502	CC(C)(C)C[C@H]1N[C@@H](C(=O)N[C@H]2CC[C@H](O)C2)[C@H](c2cccc(Cl)c2F)[C@@]12C(=O)Nc1cc(Cl)ccc12	1000.0	6.00
	2015	CHEMBL3704503	CC(C)(C)C[C@H]1N[C@@H](C(=O)NC2CCN(CCF)CC2)[C@H](c2cccc(Cl)c2F)[C@@]12C(=O)Nc1cc(Cl)ccc12	1000.0	6.00
	2015	CHEMBL3704505	CC(C)(C)C[C@H]1N[C@@H](C(=O)N[C@H]2C[C@H](NS(C)(=O)=O)C2)[C@H](c2cccc(Cl)c2F)[C@@]12C(=O)Nc1cc(Cl)ccc12	1000.0	6.00

	2015	CHEMBL3704506	CC(C)(C)C[C@H]1N[C@@H](C(=O)NCC(C)(C)O)[C@H](c2cccc(Cl)c2F)[C@@]12C(=O)Nc1cc(Cl)ccc12	1000.0	6.00
	2015	CHEMBL4113856	CC(C)(C)C[C@H]1N[C@@H](C(=O)NC2CC(O)C2)[C@H](c2cccc(Cl)c2F)[C@@]12C(=O)Nc1cc(Cl)ccc12	1000.0	6.00
	2015	CHEMBL3665579	CC(C)(C)C[C@H]1N[C@@H](C(=O)N[C@H]2C[C@@](C)(O)C2)[C@H](c2cccc(Cl)c2F)[C@@]12C(=O)Nc1cc(Cl)ccc12	1000.0	6.00
	2015	CHEMBL3665580	CC(C)(C)C[C@H]1N[C@@H](C(=O)N[C@H]2C[C@](C)(O)C2)[C@H](c2cccc(Cl)c2F)[C@@]12C(=O)Nc1cc(Cl)ccc12	1000.0	6.00
	2015	CHEMBL3704507	CC(C)(C)C[C@H]1N[C@@H](C(=O)NCCN2CCNCC2)[C@H](c2cccc(Cl)c2)[C@@]12C(=O)Nc1cc(Cl)c(F)cc12	1000.0	6.00
	2015	CHEMBL3704508	CN1CCN(CCNC(=O)[C@@H]2N[C@H](CC(C)(C)C)[C@]3(C(=O)Nc4cc(Cl)c(F)cc43)[C@H]2c2cccc(Cl)c2)CC1	1000.0	6.00
	2015	CHEMBL3704509	CN1CCN(CCCNC(=O)[C@@H]2N[C@H](CC(C)(C)C)[C@]3(C(=O)Nc4cc(Cl)c(F)cc43)[C@H]2c2cccc(Cl)c2)CC1	1000.0	6.00
	2015	CHEMBL3704510	CC(C)(C)C[C@H]1N[C@@H](C(=O)NCCC(C)(O)CO)[C@H](c2cccc(Cl)c2)[C@@]12C(=O)Nc1cc(Cl)c(F)cc12	1000.0	6.00
	2015	CHEMBL3704511	CC(C)(C)C[C@H]1N[C@@H](C(=O)NCCCN2CCCC2)[C@H](c2cccc(Cl)c2F)[C@@]12C(=O)Nc1cc(Cl)c(F)cc12	1000.0	6.00
	2015	CHEMBL4107313	CC(C)(C)C[C@H]1N[C@@H](C(=O)NC2CCC(NS(C)(=O)=O)CC2)[C@H](c2cccc(Cl)c2F)[C@@]12C(=O)Nc1cc(Cl)ccc12	1000.0	6.00
	2015	CHEMBL3986239	CC(C)(C)C[C@H]1N[C@@H](C(=O)NC2CCC(O)CC2)[C@H](c2cccc(Cl)c2F)[C@@]12C(=O)Nc1cc(Cl)ccc12	1000.0	6.00
	2015	CHEMBL3704513	CC(C)(C)C[C@H]1N[C@@H](C(=O)NC2CCN(S(C)(=O)=O)CC2)[C@H](c2cccc(Cl)c2F)[C@@]12C(=O)Nc1cc(Cl)ccc12	100.0	7.00
	2015	CHEMBL3704514	CCNC(=O)N1CCC(NC(=O)[C@@H]2N[C@H](CC(C)(C)C)[C@]3(C(=O)Nc4cc(Cl)ccc43)[C@H]2c2cccc(Cl)c2F)CC1	100.0	7.00
	2015	CHEMBL3704515	CC(C)(C)C[C@H]1N[C@@H](C(=O)N2CCNCC2)[C@H](c2cccc(Cl)c2)[C@@]12C(=O)Nc1cc(Cl)c(F)cc12	5000.0	5.30

	2015	CHEMBL3704516	CN1CCN(C(=O)[C@@H]2N[C@H](CC(C)(C)C)[C@]3(C(=O)Nc4cc(Cl)c(F)cc43)[C@H]2c2cccc(Cl)c2)CC1	5000.0	5.30
	2015	CHEMBL3704517	CC(=O)N1CCN(C(=O)[C@@H]2N[C@H](CC(C)(C)C)[C@]3(C(=O)Nc4cc(Cl)c(F)cc43)[C@H]2c2cccc(Cl)c2)CC1	10000.0	5.00
	2015	CHEMBL3704518	CN(C)CC(=O)N1CCN(C(=O)[C@@H]2N[C@H](CC(C)(C)C)[C@]3(C(=O)Nc4cc(Cl)c(F)cc43)[C@H]2c2cccc(Cl)c2)CC1	10000.0	5.00
	2015	CHEMBL3704519	CN(C)CCC(=O)N1CCN(C(=O)[C@@H]2N[C@H](CC(C)(C)C)[C@]3(C(=O)Nc4cc(Cl)c(F)cc43)[C@H]2c2cccc(Cl)c2)CC1	10000.0	5.00
	2015	CHEMBL3704520	CC(C)(C)C[C@H]1N[C@@H](C(=O)NCCN2CCC(O)CC2)[C@H](c2cccc(Cl)c2)[C@@]12C(=O)Nc1cc(Cl)c(F)cc12	1000.0	6.00
	2015	CHEMBL3704521	CC(C)(C)C[C@H]1N[C@@H](C(=O)NCC[C@H]2CC[C@H](NS(C)(=O)=O)C2)[C@H](c2cccc(Cl)c2F)[C@@]12C(=O)Nc1cc(Cl)c(F)cc12	1000.0	6.00
	2015	CHEMBL3704522	CC(C)(C)C[C@H]1N[C@@H](C(=O)NCC[C@H]2CC[C@H](N)C2)[C@H](c2cccc(Cl)c2F)[C@@]12C(=O)Nc1cc(Cl)c(F)cc12	1000.0	6.00
	2015	CHEMBL3704523	CC(C)(C)C[C@H]1N[C@@H](C(=O)NCCCNS(C)(=O)=O)[C@H](c2cccc(Cl)c2F)[C@@]12C(=O)Nc1cc(Cl)c(F)cc12	1000.0	6.00
	2015	CHEMBL3894836	C[C@H](N)C(=O)OC1CCC(NC(=O)[C@@H]2N[C@H](CC(C)(C)C)[C@]3(C(=O)Nc4cc(Cl)ccc43)[C@H]2c2cccc(Cl)c2F)CC1	100.0	7.00
	2015	CHEMBL4113676	CC(C)(C)C[C@H]1N[C@@H](C(=O)NC2CCC(N)CC2)[C@H](c2cccc(Cl)c2)[C@@]12C(=O)Nc1cc(Cl)c(F)cc12	1000.0	6.00
	2015	CHEMBL3704527	CC(C)(C)C[C@H]1N[C@@H](C(=O)NC2CCNCC2)[C@H](c2cccc(Cl)c2F)[C@@]12C(=O)Nc1cc(Cl)ccc12	1000.0	6.00
	2015	CHEMBL3704528	CC(=O)N1CCC(NC(=O)[C@@H]2N[C@H](CC(C)(C)C)[C@]3(C(=O)Nc4cc(Cl)ccc43)[C@H]2c2cccc(Cl)c2F)CC1	500.0	6.30
	2015	CHEMBL3704529	CC(C)(C)C[C@H]1N[C@@H](C(=O)NC2CCN(CCO)CC2)[C@H](c2cccc(Cl)c2F)[C@@]12C(=O)Nc1cc(Cl)ccc12	500.0	6.30

	2015	CHEMBL3704530	CC(C)(C)C[C@H]1N[C@@H](C(=O)NC2CCN(CC(C)(C)O)CC2)[C@H](c2cccc(Cl)c2F)[C@@]12C(=O)Nc1cc(Cl)ccc12	500.0	6.30
	2015	CHEMBL4114365	CC(C)(C)C[C@H]1N[C@@H](C(=O)NC2CCC(NS(=O)(=O)C(F)(F)F)CC2)[C@H](c2cccc(Cl)c2)[C@@]12C(=O)Nc1cc(Cl)c(F)cc12	5000.0	5.30
	2015	CHEMBL3704532	CC(C)(C)C[C@H]1N[C@@H](C(=O)NC2CCN(S(=O)(=O)C(F)(F)F)CC2)[C@H](c2cccc(Cl)c2F)[C@@]12C(=O)Nc1cc(Cl)ccc12	3000.0	5.52
	2015	CHEMBL3665583	CC(C)(C)C[C@H]1N[C@@H](C(=O)N[C@H]2C[C@@](C)(O)C2)[C@H](c2cccc(F)c2F)[C@@]12C(=O)Nc1cc(Cl)ccc12	1000.0	6.00
	2015	CHEMBL3665585	CC(C)(C)C[C@H]1N[C@@H](C(=O)N[C@H]2C[C@@](C)(O)C2)[C@H](c2cccc(Cl)c2F)[C@@]12C(=O)Nc1cc(F)ccc12	5000.0	5.30
	2015	CHEMBL3665586	CC(C)(C)C[C@H]1N[C@@H](C(=O)N[C@H]2C[C@@](C)(O)C2)[C@H](c2cccc(Cl)c2)[C@@]12C(=O)Nc1cc(Cl)ccc12	1000.0	6.00
	2015	CHEMBL3665587	CC(C)(C)C[C@H]1N[C@@H](C(=O)N[C@H]2C[C@@](C)(O)C2)[C@H](c2cccc(Cl)c2)[C@@]12C(=O)Nc1cc(F)c(F)cc12	3000.0	5.52
	2015	CHEMBL3665588	CC(C)(C)C[C@H]1N[C@@H](C(=O)N[C@H]2C[C@@](C)(O)C2)[C@H](c2cccc(F)c2F)[C@@]12C(=O)Nc1c2ccc(Cl)c1F	3000.0	5.52
	2015	CHEMBL3929636	CC(C)(C)C[C@H]1N[C@@H](C(=O)NC2CCC(O)CC2)[C@H](c2cccc(F)c2F)[C@@]12C(=O)Nc1cc(Cl)ccc12	3000.0	5.52
	2015	CHEMBL3951933	CC(C)(C)C[C@H]1N[C@@H](C(=O)NC2CCC(O)CC2)[C@H](c2cccc(Cl)c2F)[C@@]12C(=O)Nc1cc(F)c(F)cc12	3000.0	5.52
	2015	CHEMBL3924221	CC(C)(C)C[C@H]1N[C@@H](C(=O)NC2CCC(O)CC2)[C@H](c2cccc(Cl)c2F)[C@@]12C(=O)Nc1cc(F)ccc12	3000.0	5.52
	2015	CHEMBL4111089	CC(C)(C)C[C@H]1N[C@@H](C(=O)NCC[C@](C)(O)CO)[C@H](c2cccc(Cl)c2F)[C@@]12C(=O)Nc1cc(Cl)ccc12	3000.0	5.52
	2015	CHEMBL4112640	CC(C)(C)C[C@H]1N[C@@H](C(=O)NCC[C@@](C)(O)CO)[C@H](c2cccc(Cl)c2F)[C@@]12C(=O)Nc1cc(Cl)ccc12	3000.0	5.52
	2015	CHEMBL4115330	O=C(NC1CCC(O)CC1)[C@@H]1N[C@H](CC2CCCC2)[C@]2(C(=O)Nc3cc(Cl)ccc32)[C@H]1c1cccc(Cl)c1F	3000.0	5.52

	2015	CHEMBL4114738	O=C(NC1CCC(O)CC1)[C@@H]1N[C@H](C=C2CCCC2)[C@]2(C(=O)Nc3cc(Cl)ccc32)[C@H]1c1cccc(Cl)c1F	5000.0	5.30
	2015	CHEMBL3704536	CC(C)(C)C[C@H]1N[C@@H](C(=O)NC2CN(CC(C)(C)O)C2)[C@H](c2cccc(Cl)c2F)[C@@]12C(=O)Nc1cc(Cl)ccc12	1000.0	6.00
	2015	CHEMBL3704537	CC(C)NC(=O)N1CC(NC(=O)[C@@H]2N[C@H](CC(C)(C)C)[C@]3(C(=O)Nc4cc(Cl)ccc43)[C@H]2c2cccc(Cl)c2F)C1	3000.0	5.52
	2015	CHEMBL3704539	CC(C)(C)C[C@H]1N[C@@H](C(=O)NC2CN(C(=O)NC3CCCCC3)C2)[C@H](c2cccc(Cl)c2F)[C@@]12C(=O)Nc1cc(Cl)ccc12	3000.0	5.52
	2015	CHEMBL3704540	CC(=O)OC(C)(C)C(=O)N1CC(NC(=O)[C@@H]2N[C@H](CC(C)(C)C)[C@]3(C(=O)Nc4cc(Cl)ccc43)[C@H]2c2cccc(Cl)c2F)C1	1000.0	6.00
	2015	CHEMBL3704541	CC(=O)N1CC(NC(=O)[C@@H]2N[C@H](CC(C)(C)C)[C@]3(C(=O)Nc4cc(Cl)ccc43)[C@H]2c2cccc(Cl)c2F)C1	3000.0	5.52
	2015	CHEMBL3704542	CCCCNC(=O)N1CC(NC(=O)[C@@H]2N[C@H](CC(C)(C)C)[C@]3(C(=O)Nc4cc(Cl)ccc43)[C@H]2c2cccc(Cl)c2F)C1	100.0	7.00
	2015	CHEMBL3704543	CCN(CC)C(=O)N1CC(NC(=O)[C@@H]2N[C@H](CC(C)(C)C)[C@]3(C(=O)Nc4cc(Cl)ccc43)[C@H]2c2cccc(Cl)c2F)C1	1000.0	6.00
	2015	CHEMBL4111476	CC(C)(C)C[C@H]1N[C@@H](C(=O)NC2CCC(OC(=O)CN)CC2)[C@H](c2cccc(Cl)c2F)[C@@]12C(=O)Nc1cc(Cl)ccc12	1000.0	6.00
	2015	CHEMBL4109826	CC(C)[C@H](N)C(=O)OC1CCC(NC(=O)[C@@H]2N[C@H](CC(C)(C)C)[C@]3(C(=O)Nc4cc(Cl)ccc43)[C@H]2c2cccc(Cl)c2F)CC1	3000.0	5.52
	2015	CHEMBL3959463	CC(C)C[C@H](N)C(=O)OC1CCC(NC(=O)[C@@H]2N[C@H](CC(C)(C)C)[C@]3(C(=O)Nc4cc(Cl)ccc43)[C@H]2c2cccc(Cl)c2F)CC1	3000.0	5.52
	2015	CHEMBL3935478	CC[C@H](C)[C@H](N)C(=O)OC1CCC(NC(=O)[C@@H]2N[C@H](CC(C)(C)C)[C@]3(C(=O)Nc4cc(Cl)ccc43)[C@H]2c2cccc(Cl)c2F)CC1	1000.0	6.00
	2015	CHEMBL3950747	CC(C)(C)C[C@H]1N[C@@H](C(=O)NC2CCC(OC(=O)[C@@H](N)Cc3ccccc3)CC2)[C@H](c2cccc(Cl)c2F)[C@@]12C(=O)Nc1cc(Cl)ccc12	1000.0	6.00
	2015	CHEMBL4109822	CC(C)[C@@H](N)C(=O)OC1CCC(NC(=O)[C@@H]2N[C@H](CC(C)(C)C)[C@]3(C(=O)Nc4cc(Cl)ccc43)[C@H]2c2cccc(Cl)c2F)CC1	1000.0	6.00

	2015	CHEMBL4107182	C[C@@H](N)C(=O)OC1CCC(NC(=O)[C@@H]2N[C@H](CC(C)(C)C)[C@]3(C(=O)Nc4cc(Cl)ccc43)[C@H]2c2cccc(Cl)c2F)CC1	1000.0	6.00
	2015	CHEMBL4107983	CC(C)(C)C[C@H]1N[C@@H](C(=O)NC2CCC(OC(=O)C(C)(C)N)CC2)[C@H](c2cccc(Cl)c2F)[C@@]12C(=O)Nc1cc(Cl)ccc12	1000.0	6.00
	2015	CHEMBL2396674	CC(C)(C)C[C@@H]1N[C@@H](C(=O)N[C@H]2C[C@@](C)(O)C2)[C@H](c2cccc(Cl)c2F)[C@]12C(=O)Nc1cc(Cl)ccc12	100.0	7.00
	2015	CHEMBL3961782	CC(C)(C)C[C@@H]1N[C@@H](C(=O)NC2CCC(O)CC2)[C@H](c2cccc(Cl)c2F)[C@]12C(=O)Nc1cc(Cl)ccc12	100.0	7.00
	2015	CHEMBL3665593	CC(C)(C)C[C@@H]1N[C@@H](C(=O)N[C@H]2C[C@@](C)(O)C2)[C@H](c2cccc(F)c2F)[C@]12C(=O)Nc1cc(Cl)ccc12	100.0	7.00
	2015	CHEMBL3665594	CC(C)(C)C[C@@H]1N[C@@H](C(=O)N[C@H]2C[C@@](C)(O)C2)[C@H](c2cccc(Cl)c2F)[C@]12C(=O)Nc1cc(F)c(F)cc12	100.0	7.00
	2015	CHEMBL3665595	CC(C)(C)C[C@@H]1N[C@@H](C(=O)N[C@H]2C[C@@](C)(O)C2)[C@H](c2cccc(Cl)c2F)[C@]12C(=O)Nc1cc(Cl)c(F)cc12	100.0	7.00
	2015	CHEMBL3665596	CC(C)(C)C[C@@H]1N[C@@H](C(=O)N[C@H]2C[C@@](C)(O)C2)[C@H](c2cccc(Cl)c2)[C@]12C(=O)Nc1cc(Cl)ccc12	100.0	7.00
	2015	CHEMBL3665597	CC(C)(C)C[C@@H]1N[C@@H](C(=O)N[C@H]2C[C@@](C)(O)C2)[C@H](c2cccc(Cl)c2)[C@]12C(=O)Nc1cc(Cl)c(F)cc12	100.0	7.00
	2015	CHEMBL3665598	CC(C)(C)C[C@@H]1N[C@@H](C(=O)N[C@H]2C[C@@](C)(O)C2)[C@H](c2cccc(Cl)c2F)[C@]12C(=O)Nc1c2ccc(Cl)c1F	100.0	7.00
	2015	CHEMBL3665599	CC(C)(C)C[C@@H]1N[C@@H](C(=O)N[C@H]2C[C@@](C)(O)C2)[C@H](c2cccc(F)c2F)[C@]12C(=O)Nc1c2ccc(Cl)c1F	500.0	6.30
	2015	CHEMBL3890230	CC(C)(C)C[C@@H]1N[C@@H](C(=O)NC2CCC(C)(O)CC2)[C@H](c2cccc(Cl)c2F)[C@]12C(=O)Nc1cc(Cl)ccc12	100.0	7.00
	2015	CHEMBL3665601	CC(C)(C)C[C@@H]1N[C@@H](C(=O)N[C@H]2C[C@@](C)(O)C2)[C@H](c2cc(F)cc(Cl)c2)[C@]12C(=O)Nc1cc(Cl)ccc12	100.0	7.00
	2015	CHEMBL3932760	CC(C)(C)C[C@@H]1N[C@@H](C(=O)NC2CCC(O)CC2)[C@H](c2cccc(F)c2F)[C@]12C(=O)Nc1cc(Cl)ccc12	100.0	7.00

	2015	CHEMBL3960238	CC(C)(C)C[C@@H]1N[C@@H](C(=O)NC2CCC(O)CC2)[C@H](c2cccc(Cl)c2F)[C@]12C(=O)Nc1cc(Cl)c(F)cc12	100.0	7.00
	2015	CHEMBL3920895	CC(C)(C)C[C@@H]1N[C@@H](C(=O)NC2CCC(O)CC2)[C@H](c2cccc(Cl)c2F)[C@]12C(=O)Nc1cc(F)c(F)cc12	500.0	6.30
	2015	CHEMBL3892878	CC(C)(C)C[C@@H]1N[C@@H](C(=O)NC2CCC(O)CC2)[C@H](c2cccc(Cl)c2)[C@]12C(=O)Nc1cc(Cl)ccc12	100.0	7.00
	2015	CHEMBL3704552	O=C(NCC[C@H](O)CO)[C@@H]1N[C@H](CCc2ccccc2)[C@]2(C(=O)Nc3cc(Cl)c(F)cc32)[C@H]1c1cccc(Cl)c1	10000.0	5.00
	2015	CHEMBL3704554	O=C(NCC[C@H](O)CO)[C@@H]1N[C@H](CCc2cccs2)[C@]2(C(=O)Nc3cc(Cl)c(F)cc32)[C@H]1c1cccc(Cl)c1	1000.0	6.00
	2015	CHEMBL3663809	O=C(NCC[C@H](O)CO)[C@@H]1N[C@H](CCCc2ccccc2)[C@]2(C(=O)Nc3cc(Cl)c(F)cc32)[C@H]1c1cccc(Cl)c1	1000.0	6.00
	2015	CHEMBL3663812	O=C(NCC[C@H](O)CO)[C@@H]1N[C@H](CCc2ccsc2)[C@]2(C(=O)Nc3cc(Cl)c(F)cc32)[C@H]1c1cccc(Cl)c1	5000.0	5.30
	2015	CHEMBL3663814	CN1[C@@H](C(=O)NCC[C@H](O)CO)[C@H](c2cccc(Cl)c2)[C@@]2(C(=O)Nc3cc(Cl)c(F)cc32)[C@H]1CCCc1ccccc1	10000.0	5.00
	2015	CHEMBL3663815	O=C(NCC[C@H](O)CO)[C@@H]1N[C@H](CCCc2cccs2)[C@]2(C(=O)Nc3cc(Cl)c(F)cc32)[C@H]1c1cccc(Cl)c1	1000.0	6.00
	2015	CHEMBL3663816	CN1[C@@H](C(=O)NCC[C@H](O)CO)[C@H](c2cccc(Cl)c2)[C@@]2(C(=O)Nc3cc(Cl)c(F)cc32)[C@H]1CCCc1cccs1	5000.0	5.30
	2015	CHEMBL3663817	O=C(NCC[C@H](O)CO)[C@@H]1N[C@H](CCC2CCCC2)[C@]2(C(=O)Nc3cc(Cl)c(F)cc32)[C@H]1c1cccc(Cl)c1	3000.0	5.52
	2015	CHEMBL3663818	CN1[C@@H](C(=O)NCC[C@H](O)CO)[C@H](c2cccc(Cl)c2)[C@@]2(C(=O)Nc3cc(Cl)c(F)cc32)[C@H]1CCC1CCCC1	10000.0	5.00
	2015	CHEMBL3663819	CN1[C@@H](C(=O)NCC[C@H](O)CO)[C@H](c2cccc(Cl)c2)[C@@]2(C(=O)Nc3cc(Cl)c(F)cc32)[C@H]1CCc1cccs1	10000.0	5.00

	2015	CHEMBL3663821	O=C(NCC[C@H](O)CO)[C@@H]1N[C@H](CC2CCCCC2)[C@]2(C(=O)Nc3cc(Cl)c(F)cc32)[C@H]1c1cccc(Cl)c1	1000.0	6.00
	2015	CHEMBL3663813	CN1[C@@H](C(=O)NCC[C@H](O)CO)[C@H](c2cccc(Cl)c2)[C@@]2(C(=O)Nc3cc(Cl)c(F)cc32)[C@H]1CC(C)(C)C	3000.0	5.52
	2015	CHEMBL3705041	CC(C)(C)C[C@@H]1[C@]2(C(=O)Nc3cc(Cl)c(F)cc32)[C@@H](c2cccc(Cl)c2)[C@H](C(=O)NCC[C@H](O)CO)[N+]1(C)C	10000.0	5.00
	2015	CHEMBL3663824	CCC(C)(C)C[C@H]1N[C@@H](C(=O)NCC[C@H](O)CO)[C@H](c2cccc(Cl)c2)[C@@]12C(=O)Nc1cc(Cl)c(F)cc12	1000.0	6.00
	2015	CHEMBL3663825	CC(C)(C)C[C@H]1N[C@@H](C(=O)NCCN2CCOCC2)[C@H](c2cccc(Cl)c2F)[C@@]12C(=O)Nc1cc(F)c(F)cc12	3000.0	5.52
	2015	CHEMBL3663826	CCC(C)(C)C[C@H]1N(C)[C@@H](C(=O)NCC[C@H](O)CO)[C@H](c2cccc(Cl)c2F)[C@@]12C(=O)Nc1cc(F)c(F)cc12	5000.0	5.30
	2015	CHEMBL3663827	CCCCC(C)(C)C[C@H]1N[C@@H](C(=O)NCC[C@H](O)CO)[C@H](c2cccc(Cl)c2)[C@@]12C(=O)Nc1cc(Cl)c(F)cc12	1000.0	6.00
	2015	CHEMBL3663828	CC(C)CC(C)(C)C[C@H]1N[C@@H](C(=O)NCC[C@H](O)CO)[C@H](c2cccc(Cl)c2)[C@@]12C(=O)Nc1cc(Cl)c(F)cc12	1000.0	6.00
	2015	CHEMBL3663829	CC(C)CC(C)(C)C[C@H]1N(C)[C@@H](C(=O)NCC[C@H](O)CO)[C@H](c2cccc(Cl)c2)[C@@]12C(=O)Nc1cc(Cl)c(F)cc12	5000.0	5.30
	2015	CHEMBL3663831	CCCCC(C)(C)C[C@H]1N(C)[C@@H](C(=O)NCC[C@H](O)CO)[C@H](c2cccc(Cl)c2)[C@@]12C(=O)Nc1cc(Cl)c(F)cc12	5000.0	5.30
	2015	CHEMBL3663832	CCCC(C)(C)C[C@H]1N[C@@H](C(=O)NCC[C@H](O)CO)[C@H](c2cccc(Cl)c2)[C@@]12C(=O)Nc1cc(Cl)c(F)cc12	1000.0	6.00
	2015	CHEMBL3663833	CC(C)CC(C)(C)C[C@H]1N[C@@H](C(=O)NCCN2CCOCC2)[C@H](c2cccc(Cl)c2F)[C@@]12C(=O)Nc1cc(F)c(F)cc12	1000.0	6.00
	2015	CHEMBL3663834	CC(C)CC(C)(C)C[C@H]1N[C@@H](C(=O)NCC[C@H](O)CO)[C@H](c2cccc(Cl)c2F)[C@@]12C(=O)Nc1cc(Cl)c(F)cc12	1000.0	6.00
	2015	CHEMBL3663835	CC(C)CC(C)(C)C[C@H]1N[C@@H](C(=O)NCC[C@H](O)CO)[C@H](c2cccc(Cl)c2F)[C@@]12C(=O)Nc1cc(F)c(F)cc12	1000.0	6.00

	2015	CHEMBL3663836	CC(C)CC(C)(C)C[C@H]1N(C)[C@@H](C(=O)NCC[C@H](O)CO)[C@H](c2cccc(Cl)c2F)[C@@]12C(=O)Nc1cc(Cl)c(F)cc12	1000.0	6.00
	2015	CHEMBL3663837	CC(C)CC(C)(C)C[C@H]1N(C)[C@@H](C(=O)NCC[C@H](O)CO)[C@H](c2cccc(Cl)c2F)[C@@]12C(=O)Nc1cc(F)c(F)cc12	5000.0	5.30
	2015	CHEMBL3663838	CC(C)CC(C)(C)C[C@H]1N[C@@H](C(=O)NCC[C@H](O)CO)[C@H](c2cccc(Cl)c2)[C@@]12C(=O)Nc1cc(F)c(F)cc12	1000.0	6.00
	2015	CHEMBL3663839	CC(C)CC(C)(C)C[C@H]1N[C@@H](C(=O)NCC[C@H](O)CO)[C@H](c2ccc(F)c(Cl)c2)[C@@]12C(=O)Nc1cc(F)c(F)cc12	1000.0	6.00
	2015	CHEMBL3663840	CC(C)CC(C)(C)C[C@H]1N(C)[C@@H](C(=O)NCC[C@H](O)CO)[C@H](c2cccc(Cl)c2)[C@@]12C(=O)Nc1cc(F)c(F)cc12	1000.0	6.00
	2015	CHEMBL3663841	CC(C)CC(C)(C)C[C@H]1N(C)[C@@H](C(=O)NCC[C@H](O)CO)[C@H](c2ccc(F)c(Cl)c2)[C@@]12C(=O)Nc1cc(F)c(F)cc12	3000.0	5.52
	2015	CHEMBL3663842	CC(C)(C)C[C@H]1N[C@@H](C(=O)N2CCN(C(=O)C3CC3)CC2)[C@H](c2cccc(Cl)c2)[C@@]12C(=O)Nc1cc(Cl)c(F)cc12	1000.0	6.00
	2015	CHEMBL3663844	CC(C)(C)C[C@H]1N[C@@H](C(=O)NCC[C@H]2CC[C@@H](O)C2)[C@H](c2cccc(Cl)c2F)[C@@]12C(=O)Nc1cc(Cl)ccc12	1000.0	6.00
	2015	CHEMBL3663845	CC(C)(C)C[C@H]1N[C@@H](C(=O)NCCC2CCC(O)C2)[C@H](c2cccc(Cl)c2F)[C@@]12C(=O)Nc1cc(Cl)ccc12	1000.0	6.00
	2015	CHEMBL3663849	CC(C)(C)C[C@H]1N[C@@H](C(=O)NCC[C@H](O)COC(=O)CC(=O)O)[C@H](c2ccc(F)c(Cl)c2)[C@@]12C(=O)Nc1cc(Cl)c(F)cc12	1000.0	6.00
	2015	CHEMBL3663850	CCOP(=O)(OCC)OC[C@@H](O)CCNC(=O)[C@@H]1N[C@H](CC(C)(C)C)[C@]2(C(=O)Nc3cc(Cl)c(F)cc32)[C@H]1c1ccc(F)c(Cl)c1	5000.0	5.30
	2015	CHEMBL3663851	CN(C)C[C@@H](O)CCNC(=O)[C@@H]1N[C@H](CC(C)(C)C)[C@]2(C(=O)Nc3cc(Cl)c(F)cc32)[C@H]1c1ccc(F)c(Cl)c1	5000.0	5.30
	2015	CHEMBL3663852	CN(CC[C@H](O)CO)C(=O)[C@@H]1N[C@H](CC(C)(C)C)[C@]2(C(=O)Nc3cc(Cl)c(F)cc32)[C@H]1c1cccc(Cl)c1	5000.0	5.30
	2015	CHEMBL3663853	CNC[C@@H](O)CCNC(=O)[C@@H]1N[C@H](CC(C)(C)C)[C@]2(C(=O)Nc3cc(Cl)c(F)cc32)[C@H]1c1ccc(F)c(Cl)c1	5000.0	5.30

	2015	CHEMBL3663859	O=C(NCC[C@H](O)CO)[C@H]1[C@H](c2cccc(Cl)c2)[C@@]2(C(=O)Nc3cc(Cl)c(F)cc32)[C@H]2CC3(CCCC3)CCN12	3000.0	5.52
	2015	CHEMBL3663868	CC(C)(C)C[C@H]1N[C@@H](C(=O)NCCCO)[C@H](c2cccc(Cl)c2)[C@@]12C(=O)Nc1cc(Cl)c(F)cc12	1000.0	6.00
	2015	CHEMBL3663869	CC(C)(C)C[C@H]1N[C@@H](CO)[C@H](c2cccc(Cl)c2)[C@@]12C(=O)Nc1cc(Cl)c(F)cc12	5000.0	5.30
	2015	CHEMBL3663870	CCCN1CCN(CCNC(=O)[C@@H]2N[C@H](CC(C)(C)C)[C@]3(C(=O)Nc4cc(Cl)c(F)cc43)[C@H]2c2cccc(Cl)c2)CC1	3000.0	5.52
	2015	CHEMBL3663871	CC(C)(C)C[C@H]1N[C@@H](C(=O)NCCC2CCC(O)C2)[C@H](c2cccc(Cl)c2)[C@@]12C(=O)Nc1cc(F)c(F)cc12	10000.0	5.00
	2015	CHEMBL3663872	CN1CCC(NC(=O)[C@@H]2N[C@H](CC(C)(C)C)[C@]3(C(=O)Nc4cc(Cl)ccc43)[C@H]2c2cccc(Cl)c2F)CC1	1000.0	6.00
	2015	CHEMBL3663873	CN1CCC(CNC(=O)[C@@H]2N[C@H](CC(C)(C)C)[C@]3(C(=O)Nc4cc(Cl)ccc43)[C@H]2c2cccc(Cl)c2F)CC1	1000.0	6.00
	2015	CHEMBL3663874	CC(C)(C)C[C@H]1N[C@@H](C(=O)N[C@H]2CC[C@@H](N)C2)[C@H](c2cccc(Cl)c2F)[C@@]12C(=O)Nc1cc(Cl)ccc12	1000.0	6.00
	2015	CHEMBL3663875	CC(C)(C)C[C@H]1N[C@@H](C(=O)NC2CCN(CCC(F)(F)F)CC2)[C@H](c2cccc(Cl)c2F)[C@@]12C(=O)Nc1cc(Cl)ccc12	1000.0	6.00
	2015	CHEMBL3663876	CN1CC[C@@H](NC(=O)[C@@H]2N[C@H](CC(C)(C)C)[C@]3(C(=O)Nc4cc(Cl)ccc43)[C@H]2c2cccc(Cl)c2F)[C@@H](F)C1	1000.0	6.00
	2015	CHEMBL3663877	CC(C)(C)C[C@H]1N[C@@H](C(=O)N[C@@H]2CCN(CCF)C[C@@H]2F)[C@H](c2cccc(Cl)c2F)[C@@]12C(=O)Nc1cc(Cl)ccc12	1000.0	6.00
	2015	CHEMBL3663878	CC(C)(C)C[C@H]1N[C@@H](C(=O)NC2CN(CC(F)F)C2)[C@H](c2cccc(Cl)c2F)[C@@]12C(=O)Nc1cc(Cl)ccc12	1000.0	6.00
J Med Chem	2017	CHEMBL4083298	CC1(C)CCC2(CC1)N[C@@H](C(=O)Nc1ccc(C(=O)O)cc1)[C@H](c1cccc(Cl)c1F)[C@]21C(=O)Nc2cc(Cl)ccc21	2.4	8.62
J Med Chem	2017	CHEMBL4101295	COc1cc(C(N)=O)ccc1NC(=O)[C@@H]1NC2(CCCCC2)[C@@]2(C(=O)Nc3cc(Cl)ccc32)[C@H]1c1cccc(Cl)c1F	14.0	7.85

J Med Chem	2017	CHEMBL4102205	NC(=O)c1ccc(NC(=O)[C@@H]2NC3(CCCCC3)[C@@]3(C(=O)Nc4cc(Cl)ccc43)[C@H]2c2cccc(Cl)c2F)cc1	12.0	7.92
J Med Chem	2017	CHEMBL4077946	CS(=O)(=O)c1ccc(NC(=O)[C@@H]2NC3(CCCCC3)[C@@]3(C(=O)Nc4cc(Cl)ccc43)[C@H]2c2cccc(Cl)c2F)cc1	128.0	6.89
J Med Chem	2017	CHEMBL4064261	NS(=O)(=O)c1ccc(NC(=O)[C@@H]2NC3(CCCCC3)[C@@]3(C(=O)Nc4cc(Cl)ccc43)[C@H]2c2cccc(Cl)c2F)cc1	256.0	6.59
J Med Chem	2017	CHEMBL4085815	CS(=O)(=O)NC(=O)c1ccc(NC(=O)[C@@H]2NC3(CCCCC3)[C@@]3(C(=O)Nc4cc(Cl)ccc43)[C@H]2c2cccc(Cl)c2F)cc1	8.1	8.09
J Med Chem	2017	CHEMBL4103467	O=C(O)c1ccc(NC(=O)[C@@H]2NC3(CCCCC3)[C@@]3(C(=O)Nc4cc(Cl)ccc43)[C@H]2c2cccc(Cl)c2F)o1	18.0	7.74
J Med Chem	2017	CHEMBL3693981	O=C(O)c1ccc(NC(=O)[C@@H]2NC3(CCCCC3)[C@@]3(C(=O)Nc4cc(Cl)ccc43)[C@H]2c2cccc(Cl)c2F)cn1	9.5	8.02
J Med Chem	2017	CHEMBL4095706	O=C(O)c1ccc(NC(=O)[C@@H]2NC3(CCCCC3)[C@@]3(C(=O)Nc4cc(Cl)ccc43)[C@H]2c2cccc(Cl)c2F)c(F)c1	12.7	7.90
J Med Chem	2017	CHEMBL4077687	O=C(O)c1ccc(NC(=O)[C@@H]2NC3(CCCCC3)[C@@]3(C(=O)Nc4cc(Cl)ccc43)[C@H]2c2cccc(Cl)c2F)cc1F	7.2	8.14
J Med Chem	2017	CHEMBL3693976	COc1cc(C(=O)O)ccc1NC(=O)[C@@H]1NC2(CCCCC2)[C@@]2(C(=O)Nc3cc(Cl)ccc32)[C@H]1c1cccc(Cl)c1F	4.7	8.33
J Med Chem	2017	CHEMBL4066694	COc1cc(NC(=O)[C@@H]2NC3(CCCCC3)[C@@]3(C(=O)Nc4cc(Cl)ccc43)[C@H]2c2cccc(Cl)c2F)ccc1C(=O)O	7.5	8.12
J Med Chem	2017	CHEMBL4080118	O=C(O)Cc1ccc(NC(=O)[C@@H]2NC3(CCCCC3)[C@@]3(C(=O)Nc4cc(Cl)ccc43)[C@H]2c2cccc(Cl)c2F)cc1	17.0	7.77
J Med Chem	2017	CHEMBL3693974	O=C(O)c1ccc(CNC(=O)[C@@H]2NC3(CCCCC3)[C@@]3(C(=O)Nc4cc(Cl)ccc43)[C@H]2c2cccc(Cl)c2F)cc1	9.9	8.00
J Med Chem	2017	CHEMBL3693988	O=C(O)c1cccc(NC(=O)[C@@H]2NC3(CCCCC3)[C@@]3(C(=O)Nc4cc(Cl)ccc43)[C@H]2c2cccc(Cl)c2F)c1	8.8	8.06
J Med Chem	2017	CHEMBL3693983	CC(=O)N1CC(NC(=O)[C@@H]2NC3(CCCCC3)[C@@]3(C(=O)Nc4cc(Cl)ccc43)[C@H]2c2cccc(Cl)c2F)C1	16.9	7.77

J Med Chem	2017	CHEMBL3693982	O=C(O)CN1CCC(NC(=O)[C@@H]2NC3(CCCCC3)[C@@]3(C(=O)Nc4cc(Cl)ccc43)[C@H]2c2cccc(Cl)c2F)CC1	19.0	7.72
J Med Chem	2017	CHEMBL4099868	O=C(NC1CCS(=O)(=O)CC1)[C@@H]1NC2(CCCCC2)[C@@]2(C(=O)Nc3cc(Cl)ccc32)[C@H]1c1cccc(Cl)c1F	20.7	7.68
J Med Chem	2017	CHEMBL4072993	O=C(NC[C@H]1CC[C@H](C(=O)O)CC1)[C@@H]1NC2(CCCCC2)[C@@]2(C(=O)Nc3cc(Cl)ccc32)[C@H]1c1cccc(Cl)c1F	17.3	7.76
J Med Chem	2017	CHEMBL4092120	O=C(N[C@H]1CC[C@@H](C(=O)O)CC1)[C@@H]1NC2(CCCCC2)[C@@]2(C(=O)Nc3cc(Cl)ccc32)[C@H]1c1cccc(Cl)c1F	20.4	7.69
J Med Chem	2017	CHEMBL4064410	O=C(N[C@H]1CC[C@H](C(=O)O)CC1)[C@@H]1NC2(CCCCC2)[C@@]2(C(=O)Nc3cc(Cl)ccc32)[C@H]1c1cccc(Cl)c1F	4.4	8.36
J Med Chem	2017	CHEMBL4084366	O=C(N[C@H]1C[C@H](C(=O)O)C1)[C@@H]1NC2(CCCCC2)[C@@]2(C(=O)Nc3cc(Cl)ccc32)[C@H]1c1cccc(Cl)c1F	7.0	8.15
J Med Chem	2017	CHEMBL3693972	O=C(O)CCCNC(=O)[C@@H]1NC2(CCCCC2)[C@@]2(C(=O)Nc3cc(Cl)ccc32)[C@H]1c1cccc(Cl)c1F	9.4	8.03
J Med Chem	2017	CHEMBL3693975	CS(=O)(=O)NC(=O)[C@@H]1NC2(CCCCC2)[C@@]2(C(=O)Nc3cc(Cl)ccc32)[C@H]1c1cccc(Cl)c1F	65.0	7.19
J Med Chem	2017	CHEMBL4081906	CN1[C@@H](c2nc(C(N)=O)cs2)[C@H](c2cccc(Cl)c2F)[C@]2(C(=O)Nc3cc(Cl)ccc32)C12CCCCC2	156.0	6.81
J Med Chem	2017	CHEMBL4060006	CN1[C@@H](c2nc(CC(=O)O)cs2)[C@H](c2cccc(Cl)c2F)[C@]2(C(=O)Nc3cc(Cl)ccc32)C12CCCCC2	12.5	7.90
J Med Chem	2017	CHEMBL4066543	Cc1oc([C@H]2[C@H](c3cccc(Cl)c3F)[C@]3(C(=O)Nc4cc(Cl)ccc43)C3(CCCCC3)N2C)nc1C(=O)O	30.9	7.51
J Med Chem	2017	CHEMBL4094076	Cc1nnc([C@H]2[C@H](c3cccc(Cl)c3F)[C@]3(C(=O)Nc4cc(Cl)ccc43)C3(CCCCC3)N2C)s1	38.6	7.41
J Med Chem	2017	CHEMBL4075155	Cc1nnc([C@H]2[C@H](c3cccc(Cl)c3F)[C@]3(C(=O)Nc4cc(Cl)ccc43)C3(CCCCC3)N2C)o1	161.0	6.79
J Med Chem	2017	CHEMBL4101818	Cc1noc([C@H]2[C@H](c3cccc(Cl)c3F)[C@]3(C(=O)Nc4cc(Cl)ccc43)C3(CCCCC3)N2C)n1	603.0	6.22

J Med Chem	2017	CHEMBL4085388	CN1[C@@H](c2nc(C(=O)O)co2)[C@H](c2cccc(Cl)c2F)[C@]2(C(=O)Nc3cc(Cl)ccc32)C12CCCCC2	27.5	7.56
J Med Chem	2017	CHEMBL4063804	CN1[C@@H](c2nc(C(=O)O)cs2)[C@H](c2cccc(Cl)c2F)[C@]2(C(=O)Nc3cc(Cl)ccc32)C12CCCCC2	12.8	7.89
J Med Chem	2017	CHEMBL4093145	O=C(N[C@H]1CC[C@H](O)CC1)[C@@H]1NC2(CCCCC2)[C@@]2(C(=O)Nc3cc(Cl)sc32)[C@H]1c1cccc(Cl)c1F	31.2	7.51
J Med Chem	2017	CHEMBL4072394	O=C(N[C@H]1CC[C@H](O)CC1)[C@@H]1NC2(CCCCC2)[C@@]2(C(=O)Nc3nc(Cl)ncc32)[C@H]1c1cccc(Cl)c1F	56.8	7.25
J Med Chem	2017	CHEMBL4084744	O=C(N[C@H]1CC[C@H](O)CC1)[C@@H]1NC2(CCCCC2)[C@@]2(C(=O)Nc3nc(Cl)ccc32)[C@H]1c1cccc(Cl)c1F	51.4	7.29
J Med Chem	2017	CHEMBL4102664	O=C(N[C@H]1CC[C@H](O)CC1)[C@@H]1NC2(CCCCC2)[C@@]2(C(=O)Nc3cc(Cl)ncc32)[C@H]1c1cccc(Cl)c1F	52.6	7.28
J Med Chem	2017	CHEMBL3693987	O=C(N[C@H]1CC[C@H](O)CC1)[C@@H]1NC2(CCCCC2)[C@@]2(C(=O)Nc3cc(Cl)ccc32)[C@H]1c1cncc(Cl)c1	260.0	6.59
J Med Chem	2017	CHEMBL4064790	CC1(C)CCC2(CC1)N[C@@H](C(=O)N[C@H]1CC[C@H](O)CC1)[C@H](c1cccc(Cl)c1F)[C@]21C(=O)Nc2cc(Cl)ccc21	6.5	8.19
J Med Chem	2017	CHEMBL4100233	CC1(C)CC2(C1)N[C@@H](C(=O)N[C@H]1CC[C@H](O)CC1)[C@H](c1cccc(Cl)c1F)[C@]21C(=O)Nc2cc(Cl)ccc21	14.0	7.85
J Med Chem	2017	CHEMBL3355414	O=C(N[C@H]1CC[C@H](O)CC1)[C@@H]1NC2(CCC2)[C@@]2(C(=O)Nc3cc(Cl)ccc32)[C@H]1c1cccc(Cl)c1F	149.0	6.83
J Med Chem	2017	CHEMBL4062028	O=C(N[C@H]1CC[C@H](O)CC1)[C@@H]1NC2(CCC(F)(F)CC2)[C@@]2(C(=O)Nc3cc(Cl)ccc32)[C@H]1c1cccc(Cl)c1F	41.0	7.39
J Med Chem	2017	CHEMBL4083764	O=C(N[C@H]1CC[C@H](O)CC1)[C@@H]1NC2(CCOCC2)[C@@]2(C(=O)Nc3cc(Cl)ccc32)[C@H]1c1cccc(Cl)c1F	282.0	6.55
J Med Chem	2017	CHEMBL4093562	O=C(NC12CC(C(=O)O)(C1)C2)[C@@H]1NC2(CCCCC2)[C@@]2(C(=O)Nc3cc(Cl)ccc32)[C@H]1c1cccc(Cl)c1F	6.4	8.19
J Med Chem	2017	CHEMBL4072857	O=C(NC12CCC(C(=O)O)(CC1)CC2)[C@@H]1NC2(CCCCC2)[C@@]2(C(=O)Nc3cc(Cl)ccc32)[C@H]1c1cccc(Cl)c1F	3.7	8.43

J Med Chem	2017	CHEMBL4071840	O=C(NC12CCC(C(=O)O)(CC1)CC2)[C@@H]1NC2(CCC(F)(F)CC2)[C@@]2(C(=O)Nc3cc(Cl)ccc32)[C@H]1c1cccc(Cl)c1F	11.5	7.94
J Med Chem	2017	CHEMBL4098834	CS(=O)(=O)NC(=O)C12CCC(NC(=O)[C@@H]3NC4(CCCCC4)[C@@]4(C(=O)Nc5cc(Cl)ccc54)[C@H]3c3cccc(Cl)c3F)(CC1)CC2	6.3	8.20
J Med Chem	2017	CHEMBL4064867	CN1[C@@H](C(=O)NC23CCC(C(=O)O)(CC2)CC3)[C@H](c2cccc(Cl)c2F)[C@]2(C(=O)Nc3cc(Cl)ccc32)C12CCCCC2	4.5	8.35
J Med Chem	2017	CHEMBL4091801	CCN1[C@@H](C(=O)NC23CCC(C(=O)O)(CC2)CC3)[C@H](c2cccc(Cl)c2F)[C@]2(C(=O)Nc3cc(Cl)ccc32)C12CCCCC2	3.8	8.42
J Med Chem	2017	CHEMBL2152332	COc1ccc(C2=N[C@H](c3ccc(Cl)cc3)[C@H](c3ccc(Cl)cc3)N2C(=O)N2CCNC(=O)C2)c(OC(C)C)c1	108.0	6.97
J Med Chem	2017	CHEMBL3347643	CCOc1cc(/C=C2\C(=O)N(c3ccccc3)N=C2C)cc(Cl)c1OCC(=O)OC	1000.0	6.00
J Med Chem	2017	CHEMBL4068667	CC(CC(=O)N[C@@H](Cc1ccc(OCCCNc2ccccn2)cc1)C(=O)O)c1ccccc1	141.5	6.85
J Med Chem	2017	CHEMBL4095983	O=C(CC(c1ccccc1)c1ccccc1)N[C@@H](Cc1ccc(OCCCNc2ccccn2)cc1)C(=O)O	7.9	8.11
J Med Chem	2017	CHEMBL4064911	O=C(CC(c1ccccc1)c1ccccc1)N[C@@H](Cc1ccc(OCc2ccccc2)cc1)C(=O)O	9.2	8.04
J Med Chem	2017	CHEMBL4092607	CC(C)(C)Oc1ccc(C[C@H](NC(=O)CC(c2ccccc2)c2ccccc2)C(=O)O)cc1	115.0	6.94
J Med Chem	2017	CHEMBL4100348	O=C(CC(c1ccccc1)c1ccccc1)N[C@@H](Cc1ccc([N+](=O)[O-])cc1)C(=O)O	13.8	7.86
J Med Chem	2017	CHEMBL4095042	O=C(CC(c1ccccc1)c1ccccc1)N[C@@H](Cc1ccc(Cl)cc1)C(=O)O	65.3	7.19
J Med Chem	2017	CHEMBL4074577	O=C(CC(c1ccccc1)c1ccccc1)N[C@@H](Cc1ccccc1)C(=O)O	43.0	7.37
J Med Chem	2017	CHEMBL4080951	COC(=O)[C@H](Cc1ccc(O)cc1)NC(=O)CC(c1ccccc1)c1ccccc1	18.9	7.72

J Med Chem	2017	CHEMBL4105199	COC(=O)[C@H](Cc1ccc(O)cc1)NC(=O)CC(c1ccccc1)(c1ccccc1)c1ccccc1	35.4	7.45
J Med Chem	2017	CHEMBL4065471	O=C(CC(c1ccccc1)(c1ccccc1)c1ccccc1)N[C@@H](Cc1ccc(OCCCNc2ccccn2)cc1)C(=O)O	93.7	7.03
Eur J Med Chem	2018	CHEMBL1818538	COc1ccc(C2=NC(c3ccc(Cl)cc3)C(c3ccc(Cl)cc3)N2C(=O)N2CCN(C(C)=O)CC2)c(OC(C)C)c1	260.0	6.59
Eur J Med Chem	2018	CHEMBL2381406	CCOc1cc(OC)ccc1C1=NC(c2ccc(Br)cc2)C(c2ccc(Br)cc2)N1C(=O)N1CCN(CCO)CC1	140.0	6.85
Bioorg Med Chem Lett	2018	CHEMBL3653232	COc1ncc(-c2nc3c(n2C(C)C)[C@@H](c2ccc(Cl)cc2C)N(c2cc(Cl)ccc2C)C3=O)c(OC)n1	13.0	7.89
Bioorg Med Chem Lett	2018	CHEMBL3653164	COc1ncc(-c2nc3c(n2C(C)C)C(c2ccc(Cl)cc2C)N(c2cc(Cl)ccc2C)C3=O)c(OC)n1	0.2	9.80
Bioorg Med Chem Lett	2018	CHEMBL3653186	COc1ncncc1-c1nc2c(n1C(C)C)C(c1ccc(Cl)cc1C)N(c1cc(Cl)ccc1C)C2=O	0.3	9.59
Bioorg Med Chem Lett	2018	CHEMBL3653185	COc1ccc(-c2nc3c(n2C(C)C)C(c2ccc(Cl)cc2C)N(c2cc(Cl)ccc2C)C3=O)c(OC)n1	0.2	9.66
Bioorg Med Chem Lett	2018	CHEMBL4293896	COc1ccncc1-c1nc2c(n1C(C)C)[C@H](c1ccc(Cl)cc1C)N(c1cc(Cl)ccc1C)C2=O	0.3	9.59
Eur J Med Chem	2018	CHEMBL3125517	CN(C[C@H](C1CC1)N1C(=O)[C@@](C)(CC(=O)O)C[C@H](c2cccc(Cl)c2)[C@H]1c1ccc(Cl)cc1)S(=O)(=O)C1CC1	0.2	9.70
Eur J Med Chem	2018	CHEMBL4292264	CC1(C)CCC2(CC1)N[C@@H](C(=O)N[C@@H]1CC[C@@H](C(N)=O)OC1)[C@H](c1ccnc(Cl)c1F)[C@]21C(=O)Nc2cc(Cl)ccc21	5.6	8.25

J Med Chem	2019	CHEMBL4449512	O=C1CCC(N2Cc3c(CCCCCNC(=O)[C@@H]4NC5(CCCCC5)[C@@]5(C(=O)Nc6cc(Cl)ccc65)[C@H]4c4cccc(Cl)c4F)cccc3C2=O)C(=O)N1	67.5	7.17
J Med Chem	2019	CHEMBL3355430	O=C(O)c1ccc(NC(=O)[C@@H]2NC3(CCCCC3)[C@@]3(C(=O)Nc4cc(Cl)ccc43)[C@H]2c2cccc(Cl)c2F)cc1	9.5	8.02
J Med Chem	2019	CHEMBL3693969	CNC(=O)[C@@H]1NC2(CCCCC2)[C@@]2(C(=O)Nc3cc(Cl)ccc32)[C@H]1c1cccc(Cl)c1F	48.1	7.32
J Med Chem	2020	CHEMBL211045	COc1ccc(C2=NC(c3ccc(Cl)cc3)C(c3ccc(Cl)cc3)N2C(=O)N2CCNC(=O)C2)c(OC(C)C)c1	600.0	6.22
Eur J Med Chem	2019	CHEMBL3601398	COc1cc2c(cc1OC(C)C)[C@H](c1ccc(Cl)cc1)N(c1ccc(N(C)C[C@H]3CC[C@H](N4CCN(C)C(=O)C4)CC3)cc1)C(=O)C2	100000.0	5.00
Eur J Med Chem	2019	CHEMBL4463050	CC(C)[C@H](N1C(=O)[C@@H](CC(=O)O)C[C@@H](c2cccc(Cl)c2)[C@H]1c1ccc(Cl)cc1)S(=O)(=O)C(C)C	9.1	8.04
Eur J Med Chem	2019	CHEMBL4539285	CC[C@H]([C@@H](C)O)N1C(=O)[C@@](C)(CC(=O)O)C[C@H](c2cccc(Cl)c2)[C@H]1c1ccc(Cl)cc1	1.1	8.96
Eur J Med Chem	2019	CHEMBL3687226	Cc1cc(Cl)ccc1C1c2c(nn(Cc3ccccn3)c2C(C)C)C(=O)N1c1cccc(Cl)c1F	7800.0	5.11
Eur J Med Chem	2019	CHEMBL2177813	O=C(O)C[C@H]1C[C@H](c2cccc(Cl)c2)[C@@H](c2ccc(Cl)cc2)N(CC2CC2)C1=O	34.0	7.47
Eur J Med Chem	2019	CHEMBL4514512	COc1cc2c(cc1OC(C)C)[C@H](c1ccccc1)N(c1ccc(N(C)C[C@H]3CC[C@H](N4CCN(C)C(=O)C4)CC3)cc1)C(=O)C2	0.8	9.10
Eur J Med Chem	2019	CHEMBL4437666	O=C1Nc2cc(Cl)ccc2[C@@]1(Cc1cccc(Cl)c1)Nc1c(F)cccc1F	1.2	8.92
Eur J Med Chem	2019	CHEMBL4566706	CC(C)(C)NC(=O)[C@H](c1c(C(=O)O)[nH]c2cc(Cl)ccc12)N(C=O)Cc1ccc(Cl)cc1	400.0	6.40
Eur J Med Chem	2019	CHEMBL4447592	CC(C)(C)NC(=O)[C@H](c1c(C(=O)O)[nH]c2cc(Cl)ccc12)N(C=O)Cc1ccc(OCc2ccc(Cl)cc2)cc1	600.0	6.22
Eur J Med Chem	2019	CHEMBL208562	C[C@H](c1ccc(Cl)cc1N)N1C(=O)c2cc(I)ccc2N(CCCN2CCN(C)CC2)C(=O)[C@@H]1c1ccc(Cl)cc1	704.0	6.15

Eur J Med Chem	2019	CHEMBL2396744	O=C1Nc2ccc(I)cc2C(=O)N([C@@H](C(=O)O)c2ccc(Cl)cc2)[C@H]1c1ccc(Cl)cc1	490.0	6.31
Eur J Med Chem	2019	CHEMBL4548050	CC(C)C1=C(C(=O)N2C[C@H](F)C[C@H]2C(=O)N2CC3(CC3)NC[C@@H]2C)SC2=N[C@@](C)(c3ccc(Cl)cc3)[C@@H](c3ccc(Cl)c(F)c3)N21	1.2	8.92
Eur J Med Chem	2019	CHEMBL2402737	COc1cc(C(=O)O)ccc1NC(=O)[C@@H]1N[C@@H](CC(C)(C)C)[C@](C#N)(c2ccc(Cl)cc2F)[C@H]1c1cccc(Cl)c1F	6.0	8.22
Eur J Med Chem	2019	CHEMBL3125702	CC(C)[C@@H](CS(=O)(=O)C(C)C)N1C(=O)[C@@](C)(CC(=O)O)C[C@H](c2cccc(Cl)c2)[C@H]1c1ccc(Cl)cc1	0.6	9.22
J Med Chem	2020	CHEMBL191334	COc1ccc(C2=N[C@@H](c3ccc(Cl)cc3)[C@@H](c3ccc(Cl)cc3)N2C(=O)N2CCNC(=O)C2)c(OC(C)C)c1	88.0	7.06
J Med Chem	2020	CHEMBL2381408	CC(C)(C)C[C@@H]1N[C@@H](C(=O)N[C@H]2CC[C@H](O)CC2)[C@H](c2cccc(Cl)c2F)[C@]12C(=O)Nc1cc(Cl)ccc12	100.0	7.00

S02: SCRIPT TO RUN AUTODOCK VINA

```
#!/bin/bash

for file in ./*; do tmp=${file%.pdbqt}; name="${tmp##*/}"; \

./vina-w --receptor 5c5a_clean.pdbqt --ligand "$file" --out $name.pdbqt --log $name.log --cpu 20 --exhaustiveness 256 \

--num_modes 10 --center_x -14.768 --center_y 3.77996 --center_z -23.8846 --size_x 25.0 --size_y 25.0 --size_z 20.0; \

awk '/^[-+]+$/{getline;print FILENAME,$0}' $name.log >> temp; done; sort temp -nk 3 > 5c5a_redocking.txt; rm temp; \

mkdir logs; mv *.log logs; mkdir outputs; mv *.pdbqt outputs
```

S03: SCORING MATRIX

A scoring matrix was devised to facilitate the identification of drug candidates that are most promising for further screening and validation in the present study. The scoring matrix was constructed to assign each FDA-approved drug a numerical score ranging from 0 to 1, with the highest score corresponding to the most favorable candidates. In this study, the pIC50 values and AutoDock Vina affinity values of FDA-approved drugs were standardized using to facilitate comparison among these values. Hence, the following steps were followed to construct this matrix:

$$\boldsymbol{y_{std}} = \frac{\boldsymbol{y - y_{min}}}{\boldsymbol{y_{max} - y_{min}}} \qquad (1)$$

i. The estimated pIC50 values of FDA-approved drugs were standardized using formula (1); where *y* is the pIC50 value of a drug, y_{min} is the lowest pIC50 value among all pIC50 values predicted, and y_{max} is the highest pIC50 value among all pIC50 values predicted. This way, all pIC50 values were transformed to be distributed between 0 and 1.

ii. The determined *AutoDock Vina* affinity values of all FDA-approved drugs were also standardized using formula (1); where *y* is the AutoDock Vina affinity value (Kcal/mol), y_{min} is the lowest affinity value among all AutoDock Vina affinity values calculated, and y_{max} is the highest affinity value among all AutoDock Vina affinity values calculated. This way, all AutoDock Vina affinity values were transformed to be distributed between 0 and 1.

$$\boldsymbol{Score} = (\boldsymbol{0.3} \times \boldsymbol{y_{std,\ pIC50}}) + (\boldsymbol{0.7} \times \boldsymbol{y_{std,\ affinity}}) \qquad (2)$$

iii. In preparation for the subsequent molecular dynamics analysis, candidate drugs were scored based on the computational evidence in regard to their binding pose to MDM2. The scoring was performed using formula (2), which assigns a weight of 70% to the AutoDock Vina affinity value and 30% to the pIC50 value predicted by the machine learning model. The resulting scores, ranging from 0 to 1 (with 1 being the most preferred candidate and 0 being the least preferred), were used to select the drugs for analysis by molecular dynamics method in a sequential manner.

S04: NAMD CONFIGURATION FILES

```
#########EQUILBIRATION RUN#############
structure            step3_input.psf
coordinates          step3_input.pdb

set temp             310.15;

set outputname       step4_equilibration;

# read system values written by CHARMM (need to convert uppercases to lowercases)
exec tr "\[:upper:\]" "\[:lower:\]" < ../step3_pbcsetup.str | sed -e "s/ =//g" > step3_input.str
source               step3_input.str

temperature          $temp;

outputName           $outputname;      # base name for output from this run
                                       # NAMD writes two files at the end, final coord and vel
                                       # in the format of first-dyn.coor and first-dyn.vel
firsttimestep        0;                # last step of previous run

restartfreq          50000;            # 500 steps = every 1ps
dcdfreq              50000;
dcdUnitCell          yes;              # the file will contain unit cell info in the style of
                                       # charmm dcd files. if yes, the dcd files will contain
                                       # unit cell information in the style of charmm DCD files.
xstFreq              50000;            # XSTFreq: control how often the extended systen configuration
```

```
                        # will be appended to the XST file
outputEnergies      50000;        # 125 steps = every 0.25ps
                        # The number of timesteps between each energy output of NAMD
outputTiming        50000;        # The number of timesteps between each timing output
shows
                        # time per step and time to completion


# Force-Field Parameters
paraTypeCharmm      on;           # We're using charmm type parameter file(s)
                        # multiple definitions may be used but only one file per definition
parameters          toppar/par_all36m_prot.prm
parameters          toppar/par_all36_na.prm
parameters          toppar/par_all36_carb.prm
parameters          toppar/par_all36_lipid.prm
parameters          toppar/par_all36_cgenff.prm
parameters          toppar/par_interface.prm
parameters          toppar/toppar_all36_moreions.str
parameters          toppar/toppar_all36_nano_lig.str
parameters          toppar/toppar_all36_nano_lig_patch.str
parameters          toppar/toppar_all36_synthetic_polymer.str
parameters          toppar/toppar_all36_synthetic_polymer_patch.str
parameters          toppar/toppar_all36_polymer_solvent.str
parameters          toppar/toppar_water_ions.str
parameters          toppar/toppar_dum_noble_gases.str
parameters          toppar/toppar_ions_won.str
parameters          toppar/toppar_all36_prot_arg0.str
parameters          toppar/toppar_all36_prot_c36m_d_aminoacids.str
```

parameters	toppar/toppar_all36_prot_fluoro_alkanes.str
parameters	toppar/toppar_all36_prot_heme.str
parameters	toppar/toppar_all36_prot_na_combined.str
parameters	toppar/toppar_all36_prot_retinol.str
parameters	toppar/toppar_all36_prot_model.str
parameters	toppar/toppar_all36_prot_modify_res.str
parameters	toppar/toppar_all36_na_nad_ppi.str
parameters	toppar/toppar_all36_na_rna_modified.str
parameters	toppar/toppar_all36_lipid_sphingo.str
parameters	toppar/toppar_all36_lipid_archaeal.str
parameters	toppar/toppar_all36_lipid_bacterial.str
parameters	toppar/toppar_all36_lipid_cardiolipin.str
parameters	toppar/toppar_all36_lipid_cholesterol.str
parameters	toppar/toppar_all36_lipid_dag.str
parameters	toppar/toppar_all36_lipid_inositol.str
parameters	toppar/toppar_all36_lipid_lnp.str
parameters	toppar/toppar_all36_lipid_lps.str
parameters	toppar/toppar_all36_lipid_mycobacterial.str
parameters	toppar/toppar_all36_lipid_miscellaneous.str
parameters	toppar/toppar_all36_lipid_model.str
parameters	toppar/toppar_all36_lipid_prot.str
parameters	toppar/toppar_all36_lipid_tag.str
parameters	toppar/toppar_all36_lipid_yeast.str
parameters	toppar/toppar_all36_lipid_hmmm.str
parameters	toppar/toppar_all36_lipid_detergent.str
parameters	toppar/toppar_all36_lipid_ether.str
parameters	toppar/toppar_all36_carb_glycolipid.str

```
parameters          toppar/toppar_all36_carb_glycopeptide.str
parameters          toppar/toppar_all36_carb_imlab.str
parameters          toppar/toppar_all36_label_spin.str
parameters          toppar/toppar_all36_label_fluorophore.str
parameters          ../lig/lig.prm # Custom topology and parameter files for LIG

# Nonbonded Parameters
exclude             scaled1-4         # non-bonded exclusion policy to use "none,1-2,1-3,1-4,or
scaled1-4"
                                      # 1-2: all atoms pairs that are bonded are going to be ignored
                                      # 1-3: 3 consecutively bonded are excluded
                                      # scaled1-4: include all the 1-3, and modified 1-4 interactions
                                      # electrostatic scaled by 1-4scaling factor 1.0
                                      # vdW special 1-4 parameters in charmm parameter file.
1-4scaling          1.0
switching           on
vdwForceSwitching       on;              # New option for force-based switching of vdW
                                      # if both switching and vdwForceSwitching are on CHARMM force
                                      # switching is used for vdW forces.

# You have some freedom choosing the cutoff
cutoff              12.0;             # may use smaller, maybe 10., with PME
switchdist          10.0;             # cutoff - 2.
                                      # switchdist - where you start to switch
                                      # cutoff - where you stop accounting for nonbond interactions.
                                      # correspondence in charmm:
                                      # (cutnb,ctofnb,ctonnb = pairlistdist,cutoff,switchdist)
```

```
pairlistdist          16.0;           # stores the all the pairs with in the distance it should be larger
                                      # than cutoff( + 2.)
stepspercycle         20;             # 20 redo pairlists every ten steps
pairlistsPerCycle     2;              # 2 is the default
                                      # cycle represents the number of steps between atom reassignments
                                      # this means every 20/2=10 steps the pairlist will be updated

# Integrator Parameters
timestep              2.0;            # fs/step
rigidBonds            all;            # Bound constraint all bonds involving H are fixed in length
nonbondedFreq         1;              # nonbonded forces every step
fullElectFrequency    1;              # PME every step

# Constant Temperature Control ONLY DURING EQUILB
reassignFreq          10000;          # reassignFreq:  use this to reassign velocity every 500 steps
reassignTemp          $temp;

# Periodic Boundary conditions. Need this since for a start...
cellBasisVector1    $a   0.0   0.0;       # vector to the next image
cellBasisVector2    0.0   $b   0.0;
cellBasisVector3    0.0   0.0   $c;
cellOrigin          0.0   0.0 $zcen;      # the *center* of the cell

wrapWater             on;             # wrap water to central cell
wrapAll               on;             # wrap other molecules too
wrapNearest           off;            # use for non-rectangular cells (wrap to the nearest image)
```

```
# PME (for full-system periodic electrostatics)
PME                  yes;
PMEInterpOrder       6;           # interpolation order (spline order 6 in charmm)
PMEGridSpacing       1.0;         # maximum PME grid space / used to calculate grid size

# Constant Temperature Control
langevin             on
langevinDamping      1.0
langevinTemp         $temp
langevinHydrogen     off

constraints          on
consexp              2
consref              restraints/prot_posres.ref
conskfile            restraints/prot_posres.ref
conskcol             B
constraintScaling    1.0

minimize             50000
run                  5000000;     # 10ns
```

```
#########PRODUCTION RUN#############
structure            step3_input.psf
coordinates          step3_input.pdb

set temp             310.15;
outputName           step5_production;   # base name for output from this run
                            # NAMD writes two files at the end, final coord and vel
                            # in the format of first-dyn.coor and first-dyn.vel

set inputname        step4_equilibration;
binCoordinates       $inputname.coor;    # coordinates from last run (binary)
binVelocities        $inputname.vel;     # velocities from last run (binary)
extendedSystem       $inputname.xsc;     # cell dimensions from last run (binary)

dcdfreq              5000;
dcdUnitCell          yes;         # the file will contain unit cell info in the style of
                            # charmm dcd files. if yes, the dcd files will contain
                            # unit cell information in the style of charmm DCD files.
xstFreq              5000;        # XSTFreq: control how often the extended systen configuration
                            # will be appended to the XST file
outputEnergies       5000;        # 5000 steps = every 10ps
                            # The number of timesteps between each energy output of NAMD
outputTiming         5000;        # The number of timesteps between each timing output
shows
                            # time per step and time to completion
restartfreq          5000;        # 5000 steps = every 10ps
```

```
# Force-Field Parameters
paraTypeCharmm      on;         # We're using charmm type parameter file(s)
                                # multiple definitions may be used but only one file per definition
parameters      toppar/par_all36m_prot.prm
parameters      toppar/par_all36_na.prm
parameters      toppar/par_all36_carb.prm
parameters      toppar/par_all36_lipid.prm
parameters      toppar/par_all36_cgenff.prm
parameters      toppar/par_interface.prm
parameters      toppar/toppar_all36_moreions.str
parameters      toppar/toppar_all36_nano_lig.str
parameters      toppar/toppar_all36_nano_lig_patch.str
parameters      toppar/toppar_all36_synthetic_polymer.str
parameters      toppar/toppar_all36_synthetic_polymer_patch.str
parameters      toppar/toppar_all36_polymer_solvent.str
parameters      toppar/toppar_water_ions.str
parameters      toppar/toppar_dum_noble_gases.str
parameters      toppar/toppar_ions_won.str
parameters      toppar/toppar_all36_prot_arg0.str
parameters      toppar/toppar_all36_prot_c36m_d_aminoacids.str
parameters      toppar/toppar_all36_prot_fluoro_alkanes.str
parameters      toppar/toppar_all36_prot_heme.str
parameters      toppar/toppar_all36_prot_na_combined.str
parameters      toppar/toppar_all36_prot_retinol.str
parameters      toppar/toppar_all36_prot_model.str
parameters      toppar/toppar_all36_prot_modify_res.str
parameters      toppar/toppar_all36_na_nad_ppi.str
```

```
parameters	toppar/toppar_all36_na_rna_modified.str
parameters	toppar/toppar_all36_lipid_sphingo.str
parameters	toppar/toppar_all36_lipid_archaeal.str
parameters	toppar/toppar_all36_lipid_bacterial.str
parameters	toppar/toppar_all36_lipid_cardiolipin.str
parameters	toppar/toppar_all36_lipid_cholesterol.str
parameters	toppar/toppar_all36_lipid_dag.str
parameters	toppar/toppar_all36_lipid_inositol.str
parameters	toppar/toppar_all36_lipid_lnp.str
parameters	toppar/toppar_all36_lipid_lps.str
parameters	toppar/toppar_all36_lipid_mycobacterial.str
parameters	toppar/toppar_all36_lipid_miscellaneous.str
parameters	toppar/toppar_all36_lipid_model.str
parameters	toppar/toppar_all36_lipid_prot.str
parameters	toppar/toppar_all36_lipid_tag.str
parameters	toppar/toppar_all36_lipid_yeast.str
parameters	toppar/toppar_all36_lipid_hmmm.str
parameters	toppar/toppar_all36_lipid_detergent.str
parameters	toppar/toppar_all36_lipid_ether.str
parameters	toppar/toppar_all36_carb_glycolipid.str
parameters	toppar/toppar_all36_carb_glycopeptide.str
parameters	toppar/toppar_all36_carb_imlab.str
parameters	toppar/toppar_all36_label_spin.str
parameters	toppar/toppar_all36_label_fluorophore.str
parameters	../lig/lig.prm # Custom topology and parameter files for LIG

# Nonbonded Parameters
```

```
exclude             scaled1-4        # non-bonded exclusion policy to use "none,1-2,1-3,1-4,or
scaled1-4"
                              # 1-2: all atoms pairs that are bonded are going to be ignored
                              # 1-3: 3 consecutively bonded are excluded
                              # scaled1-4: include all the 1-3, and modified 1-4 interactions
                              # electrostatic scaled by 1-4scaling factor 1.0
                              # vdW special 1-4 parameters in charmm parameter file.
1-4scaling          1.0
switching           on
vdwForceSwitching      on;           # New option for force-based switching of vdW
                              # if both switching and vdwForceSwitching are on CHARMM force
                              # switching is used for vdW forces.

# You have some freedom choosing the cutoff
cutoff              12.0;          # may use smaller, maybe 10., with PME
switchdist          10.0;          # cutoff - 2.
                              # switchdist - where you start to switch
                              # cutoff - where you stop accounting for nonbond interactions.
                              # correspondence in charmm:
                              # (cutnb,ctofnb,ctonnb = pairlistdist,cutoff,switchdist)
pairlistdist        16.0;          # stores the all the pairs with in the distance it should be larger
                              # than cutoff( + 2.)
stepspercycle         20;           # 20 redo pairlists every ten steps
pairlistsPerCycle      2;           # 2 is the default
                              # cycle represents the number of steps between atom reassignments
                              # this means every 20/2=10 steps the pairlist will be updated
```

Integrator Parameters

timestep 2.0; # fs/step

rigidBonds all; # Bound constraint all bonds involving H are fixed in length

nonbondedFreq 1; # nonbonded forces every step

fullElectFrequency 1; # PME every step

wrapWater on; # wrap water to central cell

wrapAll on; # wrap other molecules too

wrapNearest off; # use for non-rectangular cells (wrap to the nearest image)

PME (for full-system periodic electrostatics)

PME yes;

PMEInterpOrder 6; # interpolation order (spline order 6 in charmm)

PMEGridSpacing 1.0; # maximum PME grid space / used to calculate grid size

Constant Pressure Control (variable volume)

useGroupPressure yes; # use a hydrogen-group based pseudo-molecular viral to calcualte pressure and

has less fluctuation, is needed for rigid bonds (rigidBonds/SHAKE)

useFlexibleCell no; # yes for anisotropic system like membrane

useConstantRatio no; # keeps the ratio of the unit cell in the x-y plane constant A=B

Constant Temperature Control

langevin on; # langevin dynamics

langevinDamping 1.0; # damping coefficient of 1/ps (keep low)

langevinTemp $temp; # random noise at this level

langevinHydrogen off; # don't couple bath to hydrogens

```
# Constant pressure

langevinPiston          on;               # Nose-Hoover Langevin piston pressure control

langevinPistonTarget    1.01325;          # target pressure in bar 1atm = 1.01325bar

langevinPistonPeriod    50.0;             # oscillation period in fs. correspond to pgamma
T=50fs=0.05ps

                                 # f=1/T=20.0(pgamma)

langevinPistonDecay     25.0;             # oscillation decay time. smaller value corresponds to
larger random

                                 # forces and increased coupling to the Langevin temp bath.

                                 # Equal or smaller than piston period

langevinPistonTemp      $temp;            # coupled to heat bath


# run

run                 50000000;         # 100ns
```

S05: SPREADSHEET OF NEUROBLASTOMA AND GLIOBLASTOMA CELL LINES WITH WILD-TYPE P53-MDM2

Table S2. Spreadsheet of neuroblastoma and glioblastoma cell lines with wild type *TP53* and *MDM2* genes.

LongTable-checkbox	lineage1	lineage2	lineage3	depmapId	displayName	TP53 (p53, LFS1) Hotspot Mutations	MDM2 (HDM2, MGC5370) Damaging Mutations
TRUE	CNS/Brain	Diffuse Glioma	Glioblastoma	ACH-000075	U87MG	0	0
FALSE	CNS/Brain	Diffuse Glioma	Glioblastoma	ACH-000283	A1207	0	0
FALSE	CNS/Brain	Diffuse Glioma	Glioblastoma	ACH-000558	A172	0	0
FALSE	Peripheral Nervous System	Neuroblastoma	Neuroblastoma	ACH-002389	ACN	0	0
FALSE	CNS/Brain	Diffuse Glioma	Glioblastoma	ACH-000269	AM38	0	0
FALSE	CNS/Brain	Diffuse Glioma	Glioblastoma	ACH-001329	ANGMCSS	0	0
FALSE	Peripheral Nervous System	Neuroblastoma	Neuroblastoma	ACH-001300	CHLA15	0	0

FALSE	Peripheral Nervous System	Neuroblastoma	Neuroblastoma	ACH-000136	CHP126	0	0
FALSE	Peripheral Nervous System	Neuroblastoma	Neuroblastoma	ACH-001338	CHP134	0	0
FALSE	Peripheral Nervous System	Neuroblastoma	Neuroblastoma	ACH-000120	CHP212	0	0
FALSE	Peripheral Nervous System	Neuroblastoma	Neuroblastoma	ACH-001301	COGN278	0	0
FALSE	Peripheral Nervous System	Neuroblastoma	Neuroblastoma	ACH-001302	COGN305	0	0
FALSE	CNS/Brain	Diffuse Glioma	Glioblastoma	ACH-002223	D245MG	0	0
FALSE	CNS/Brain	Diffuse Glioma	Glioblastoma	ACH-002227	D392MG	0	0
FALSE	CNS/Brain	Diffuse Glioma	Glioblastoma	ACH-002229	D502MG	0	0
FALSE	CNS/Brain	Diffuse Glioma	Glioblastoma	ACH-000863	DBTRG05MG	0	0
FALSE	CNS/Brain	Diffuse Glioma	Glioblastoma	ACH-000244	DKMG	0	0
FALSE	CNS/Brain	Diffuse Glioma	Glioblastoma	ACH-000738	GB1	0	0
FALSE	Peripheral Nervous System	Neuroblastoma	Neuroblastoma	ACH-001344	GIMEN	0	0
FALSE	CNS/Brain	Diffuse Glioma	Glioblastoma	ACH-000027	GOS3	0	0
FALSE	Peripheral Nervous System	Neuroblastoma	Neuroblastoma	ACH-001716	GOTO	0	0

FALSE	Peripheral Nervous System	Neuroblastoma	Neuroblastoma	ACH-000310	IMR32	0	0
FALSE	Peripheral Nervous System	Neuroblastoma	Neuroblastoma	ACH-002248	IMR5	0	0
FALSE	Peripheral Nervous System	Neuroblastoma	Neuroblastoma	ACH-000259	KELLY	0	0
FALSE	CNS/Brain	Diffuse Glioma	Glioblastoma	ACH-000479	KNS81	0	0
FALSE	CNS/Brain	Diffuse Glioma	Glioblastoma	ACH-002259	KNS81FD	0	0
FALSE	Peripheral Nervous System	Neuroblastoma	Neuroblastoma	ACH-000345	KPNRTBM1	0	0
FALSE	Peripheral Nervous System	Neuroblastoma	Neuroblastoma	ACH-000446	KPNSI9S	0	0
FALSE	Peripheral Nervous System	Neuroblastoma	Neuroblastoma	ACH-000227	KPNYN	0	0
FALSE	CNS/Brain	Diffuse Glioma	Glioblastoma	ACH-000631	KS1	0	0
FALSE	CNS/Brain	Diffuse Glioma	Glioblastoma	ACH-000819	LN18	0	0
FALSE	CNS/Brain	Diffuse Glioma	Glioblastoma	ACH-000328	LN215	0	0
FALSE	CNS/Brain	Diffuse Glioma	Glioblastoma	ACH-000595	LN229	0	0
FALSE	CNS/Brain	Diffuse Glioma	Glioblastoma	ACH-000634	LN340	0	0
FALSE	CNS/Brain	Diffuse Glioma	Glioblastoma	ACH-000215	LN382	0	0

FALSE	CNS/Brain	Diffuse Glioma	Glioblastoma	ACH-000676	LN464	0	0
FALSE	CNS/Brain	Diffuse Glioma	Glioblastoma	ACH-000760	LNZ308	0	0
FALSE	Peripheral Nervous System	Neuroblastoma	Neuroblastoma	ACH-001548	LS	0	0
FALSE	Peripheral Nervous System	Neuroblastoma	Neuroblastoma	ACH-000078	MHHNB11	0	0
FALSE	Peripheral Nervous System	Neuroblastoma	Neuroblastoma	ACH-000804	NB1	0	0
FALSE	Peripheral Nervous System	Neuroblastoma	Neuroblastoma	ACH-002278	NB10	0	0
FALSE	Peripheral Nervous System	Neuroblastoma	Neuroblastoma	ACH-002279	NB12	0	0
FALSE	Peripheral Nervous System	Neuroblastoma	Neuroblastoma	ACH-002281	NB14	0	0
FALSE	Peripheral Nervous System	Neuroblastoma	Neuroblastoma	ACH-001303	NB1643	0	0
FALSE	Peripheral Nervous System	Neuroblastoma	Neuroblastoma	ACH-002282	NB17	0	0
FALSE	Peripheral Nervous System	Neuroblastoma	Neuroblastoma	ACH-002283	NB5	0	0
FALSE	Peripheral Nervous System	Neuroblastoma	Neuroblastoma	ACH-002083	NB69	0	0
FALSE	Peripheral Nervous System	Neuroblastoma	Neuroblastoma	ACH-002277	NBTU110	0	0
FALSE	Peripheral Nervous System	Neuroblastoma	Neuroblastoma	ACH-001603	NH12	0	0

FALSE	Peripheral Nervous System	Neuroblastoma	Neuroblastoma	ACH-000203	NH6	0	0
FALSE	CNS/Brain	Diffuse Glioma	Glioblastoma	ACH-000200	NMCG1	0	0
FALSE	CNS/Brain	Diffuse Glioma	Glioblastoma	ACH-001609	NP3	0	0
FALSE	CNS/Brain	Diffuse Glioma	Glioblastoma	ACH-001611	NP8	0	0
FALSE	CNS/Brain	Diffuse Glioma	Glioblastoma	ACH-001622	ONDA7	0	0
FALSE	CNS/Brain	Diffuse Glioma	Glioblastoma	ACH-001624	ONDA9	0	0
FALSE	CNS/Brain	Diffuse Glioma	Glioblastoma	ACH-000609	SF126	0	0
TRUE	Peripheral Nervous System	Neuroblastoma	Neuroblastoma	ACH-001188	SHSY5Y	0	0
FALSE	Peripheral Nervous System	Neuroblastoma	Neuroblastoma	ACH-000099	SIMA	0	0
FALSE	Peripheral Nervous System	Neuroblastoma	Neuroblastoma	ACH-000260	SKNAS	0	0
FALSE	Peripheral Nervous System	Neuroblastoma	Neuroblastoma	ACH-000341	SKNFI	0	0
FALSE	Peripheral Nervous System	Neuroblastoma	Neuroblastoma	ACH-000149	SKNSH	0	0
FALSE	CNS/Brain	Diffuse Glioma	Glioblastoma	ACH-000504	SNB75	0	0
FALSE	CNS/Brain	Diffuse Glioma	Glioblastoma	ACH-000623	SNU201	0	0

FALSE	CNS/Brain	Diffuse Glioma	Glioblast oma	ACH-000289	SNU466	0	0
FALSE	CNS/Brain	Diffuse Glioma	Glioblast oma	ACH-000543	SNU489	0	0
FALSE	CNS/Brain	Diffuse Glioma	Glioblast oma	ACH-000370	SNU626	0	0
FALSE	Peripheral Nervous System	Neurobla stoma	Neurobla stoma	ACH-001674	TGW	0	0
FALSE	Peripheral Nervous System	Neurobla stoma	Neurobla stoma	ACH-002080	TN2	0	0
FALSE	CNS/Brain	Diffuse Glioma	Glioblast oma	ACH-000036	U343	0	0
FALSE	CNS/Brain	Diffuse Glioma	Glioblast oma	ACH-000469	YH13	0	0
FALSE	CNS/Brain	Diffuse Glioma	Glioblast oma	ACH-000570	YKG1	0	0

S06: IC_{50} CALCULATION

For IC_{50} calculation, we first calculated the percentage of cell viability (V) using the formula below:

$$\boldsymbol{V_x}\,(\%) = \frac{\boldsymbol{A^{x}_{590-690}}}{\boldsymbol{A^{\overline{cx}}_{590-690}}}\ \boldsymbol{X\ 100}$$

Where V_x is the cell viability at given concentration, A is the absorbance, $A^{x}_{590-690}$ is $A_{590} - A_{690}$ for a given concentration x, and $A^{\overline{cx}}_{590-690}$ is average of $A_{590} - A_{690}$ for a given concentration x of the vehicle in the control group.

After calculating cell viability for each concentration, log_{10} transformation was applied to the concentrations using GraphPad Prism (v8.4.3 for Windows, GraphPad Software, San Diego, California USA, www.graphpad.com). Cell viability percentages were normalized with the same application and a log(inhibitor) vs. normalized response (variable slope) nonlinear curve fit was performed [log(inhibitor) vs. normalized response - variable slope nonlinear regression curve fit performed in GraphPad Prisim v8.4.3 for Windows, GraphPad Software, San Diego, California USA, www.graphpad.com)]. The IC50 for each drug-cell line was determined from their respective nonlinear curve fit by GraphPad Prism.

S07: REACTOME & GO ENRICHMENT

Table S3. Reactome enrichment results.

#term ID	term description	observed gene count	background gene count	strength	false discovery rate	matching proteins in your network (IDs)	matching proteins in your network (labels)
HSA-3700989	Transcriptional Regulation by TP53	4	363	1.73	0.00015	9606.ENSP00000256996,9606.ENSP00000269305,9606.ENSP00000293288,9606.ENSP00000384849	DDB2,TP53,BAX,CDKN1A
HSA-6791312	TP53 Regulates Transcription of Cell Cycle Genes	3	49	2.48	0.00015	9606.ENSP00000269305,9606.ENSP00000293288,9606.ENSP00000384849	TP53,BAX, CDKN1A
HSA-69895	Transcriptional activation of cell cycle inhibitor p21	2	4	3.39	0.00034	9606.ENSP00000269305,9606.ENSP00000384849	TP53,CDKN1A
HSA-8941855	RUNX3 regulates CDKN1A transcription	2	7	3.15	0.00049	9606.ENSP00000269305,9606.ENSP00000384849	TP53,CDKN1A
HSA-6804116	TP53 Regulates Transcription of Genes Involved in G1 Cell Cycle Arrest	2	14	2.84	0.0014	9606.ENSP00000269305,9606.ENSP00000384849	TP53,CDKN1A

HSA-6803204	TP53 Regulates Transcription of Genes Involved in Cytochrome C Release	2	19	2.71	0.0018	9606.ENSP00000269305,9606.ENSP00000293288	TP53,BAX
HSA-6804114	TP53 Regulates Transcription of Genes Involved in G2 Cell Cycle Arrest	2	18	2.74	0.0018	9606.ENSP00000269305,9606.ENSP00000293288	TP53,BAX
HSA-109606	Intrinsic Pathway for Apoptosis	2	51	2.28	0.0072	9606.ENSP00000269305,9606.ENSP00000293288	TP53,BAX
HSA-2559586	DNA Damage/Telomere Stress Induced Senescence	2	61	2.21	0.0095	9606.ENSP00000269305,9606.ENSP00000384849	TP53,CDKN1A
HSA-6796648	TP53 Regulates Transcription of DNA Repair Genes	2	65	2.18	0.0095	9606.ENSP00000256996,9606.ENSP00000269305	DDB2,TP53
HSA-8852276	The role of GTSE1 in G2/M progression after G2 checkpoint	2	75	2.12	0.0104	9606.ENSP00000269305,9606.ENSP00000384849	TP53,CDKN1A
HSA-6785807	Interleukin-4 and Interleukin-13 signaling	2	107	1.96	0.0189	9606.ENSP00000269305,9606.ENSP00000384849	TP53,CDKN1A
HSA-8878166	Transcriptional regulation by RUNX2	2	118	1.92	0.0219	9606.ENSP00000293288,9606.ENSP00000384849	BAX,CDKN1A

Table S4. Gene ontology enrichment results.

#term ID	term description	observed gene count	background gene count	strength	false discovery rate	matching proteins in your network (IDs)	matching proteins in your network (labels)
GO:0034644	Cellular response to uv	4	83	2.37	4.68E-06	9606.ENSP00000256996,9606.ENSP00000269305,9606.ENSP00000293288,9606.ENSP00000384849	DDB2,TP53,BAX,CDKN1A
GO:0006974	Cellular response to dna damage stimulus	4	793	1.39	0.00086	9606.ENSP00000256996,9606.ENSP00000269305,9606.ENSP00000293288,9606.ENSP00000384849	DDB2,TP53,BAX,CDKN1A
GO:0090304	Nucleic acid metabolic process	4	2178	0.95	0.0178	9606.ENSP00000256996,9606.ENSP00000269305,9606.ENSP00000293288,9606.ENSP00000384849	DDB2,TP53,BAX,CDKN1A
GO:0051128	Regulation of cellular component organization	4	2402	0.91	0.0232	9606.ENSP00000256996,9606.ENSP00000269305,9606.ENSP00000293288,9606.ENSP00000384849	DDB2,TP53,BAX,CDKN1A
GO:0072332	Intrinsic apoptotic signaling pathway by p53 class mediator	3	51	2.46	0.00015	9606.ENSP00000269305,9606.ENSP00000293288,9606.ENSP00000384849	TP53,BAX,CDKN1A
GO:0006977	DNA damage response, signal transduction by p53 class mediator resulting in cell cycle arrest	3	59	2.4	0.00016	9606.ENSP00000269305,9606.ENSP00000293288,9606.ENSP00000384849	TP53,BAX,CDKN1A

GO:0008630	Intrinsic apoptotic signaling pathway in response to dna damage	3	71	2.32	0.00016	9606.ENSP00000269305,9606.ENSP00000293288,9606.ENSP00000384849	TP53,BAX,CDKN1A
GO:0010332	Response to gamma radiation	3	58	2.4	0.00016	9606.ENSP00000269305,9606.ENSP00000293288,9606.ENSP00000384849	TP53,BAX,CDKN1A
GO:0048145	Regulation of fibroblast proliferation	3	83	2.25	0.00017	9606.ENSP00000269305,9606.ENSP00000293288,9606.ENSP00000384849	TP53,BAX,CDKN1A
GO:0031334	Positive regulation of protein-containing complex assembly	3	249	1.77	0.0021	9606.ENSP00000256996,9606.ENSP00000269305,9606.ENSP00000293288	DDB2,TP53,BAX
GO:0051052	Regulation of dna metabolic process	3	360	1.61	0.0043	9606.ENSP00000269305,9606.ENSP00000293288,9606.ENSP00000384849	TP53,BAX,CDKN1A
GO:0072594	Establishment of protein localization to organelle	3	433	1.53	0.0067	9606.ENSP00000269305,9606.ENSP00000293288,9606.ENSP00000384849	TP53,BAX,CDKN1A
GO:0045936	Negative regulation of phosphate metabolic process	3	561	1.42	0.0125	9606.ENSP00000269305,9606.ENSP00000293288,9606.ENSP00000384849	TP53,BAX,CDKN1A
GO:0006351	Transcription, dna-templated	3	567	1.41	0.0126	9606.ENSP00000269305,9606.ENSP00000293288,9606.ENSP00000384849	TP53,BAX,CDKN1A
GO:0043	Positive regulation of programmed cell death	3	666	1.34	0.0178	9606.ENSP00000269305,9606.ENSP00000293288,9606.ENSP00000384849	TP53,BAX,CDKN1A

068							
GO:0008285	Negative regulation of cell population proliferation	3	696	1.32	0.0195	9606.ENSP00000269305,9606.ENSP00000293288,9606.ENSP00000384849	TP53,BAX,CDKN1A
GO:0072359	Circulatory system development	3	872	1.23	0.0331	9606.ENSP00000269305,9606.ENSP00000293288,9606.ENSP00000384849	TP53,BAX,CDKN1A
GO:0043066	Negative regulation of apoptotic process	3	893	1.22	0.0352	9606.ENSP00000269305,9606.ENSP00000293288,9606.ENSP00000384849	TP53,BAX,CDKN1A
GO:0006886	Intracellular protein transport	3	999	1.17	0.0442	9606.ENSP00000269305,9606.ENSP00000293288,9606.ENSP00000384849	TP53,BAX,CDKN1A
GO:0032269	Negative regulation of cellular protein metabolic process	3	1043	1.15	0.0485	9606.ENSP00000269305,9606.ENSP00000293288,9606.ENSP00000384849	TP53,BAX,CDKN1A
GO:0090400	Stress-induced premature senescence	2	8	3.09	0.00053	9606.ENSP00000269305,9606.ENSP00000384849	TP53,CDKN1A
GO:0090399	Replicative senescence	2	13	2.88	0.00098	9606.ENSP00000269305,9606.ENSP00000384849	TP53,CDKN1A
GO:0006978	DNA damage response, signal transduction by p53 class mediator resulting in transcription of p21 class mediator	2	15	2.81	0.0012	9606.ENSP00000269305,9606.ENSP00000384849	TP53,CDKN1A

GO:0071850	Mitotic cell cycle arrest	2	16	2.79	0.0013	9606.ENSP00000269305,9606.ENSP00000384849	TP53,CDKN1A
GO:1900119	Positive regulation of execution phase of apoptosis	2	16	2.79	0.0013	9606.ENSP00000269305,9606.ENSP00000293288	TP53,BAX
GO:0070230	Positive regulation of lymphocyte apoptotic process	2	19	2.71	0.0017	9606.ENSP00000269305,9606.ENSP00000293288	TP53,BAX
GO:0001836	Release of cytochrome c from mitochondria	2	22	2.65	0.0021	9606.ENSP00000269305,9606.ENSP00000293288	TP53,BAX
GO:0035794	Positive regulation of mitochondrial membrane permeability	2	22	2.65	0.0021	9606.ENSP00000269305,9606.ENSP00000293288	TP53,BAX
GO:0009651	Response to salt stress	2	23	2.63	0.0022	9606.ENSP00000269305,9606.ENSP00000293288	TP53,BAX
GO:1902108	Regulation of mitochondrial membrane permeability involved in apoptotic process	2	23	2.63	0.0022	9606.ENSP00000269305,9606.ENSP00000293288	TP53,BAX
GO:0090200	Positive regulation of release of cytochrome c from mitochondria	2	28	2.54	0.0029	9606.ENSP00000269305,9606.ENSP00000293288	TP53,BAX
GO:0097	Programmed necrotic cell death	2	28	2.54	0.0029	9606.ENSP00000269305,9606.ENSP00000293288	TP53,BAX

300							
GO:0048147	Negative regulation of fibroblast proliferation	2	29	2.53	0.003	9606.ENSP00000269305,9606.ENSP00000293288	TP53,BAX
GO:0010165	Response to x-ray	2	31	2.5	0.0031	9606.ENSP00000269305,9606.ENSP00000384849	TP53,CDKN1A
GO:0042771	Intrinsic apoptotic signaling pathway in response to dna damage by p53 class mediator	2	30	2.51	0.0031	9606.ENSP00000269305,9606.ENSP00000384849	TP53,CDKN1A
GO:0071480	Cellular response to gamma radiation	2	31	2.5	0.0031	9606.ENSP00000269305,9606.ENSP00000384849	TP53,CDKN1A
GO:0070059	Intrinsic apoptotic signaling pathway in response to endoplasmic reticulum stress	2	34	2.46	0.0035	9606.ENSP00000269305,9606.ENSP00000293288	TP53,BAX
GO:0042098	T cell proliferation	2	36	2.43	0.0039	9606.ENSP00000269305,9606.ENSP00000293288	TP53,BAX
GO:1901030	Positive regulation of mitochondrial outer membrane permeabilization involved in apoptotic signaling pathway	2	37	2.42	0.004	9606.ENSP00000269305,9606.ENSP00000293288	TP53,BAX
GO:2000279	Negative regulation of dna biosynthetic process	2	42	2.37	0.0049	9606.ENSP00000269305,9606.ENSP00000384849	TP53,CDKN1A

GO:0051402	Neuron apoptotic process	2	46	2.33	0.0057	9606.ENSP00000269305,9606.ENSP00000293288	TP53,BAX
GO:0043525	Positive regulation of neuron apoptotic process	2	58	2.23	0.008	9606.ENSP00000269305,9606.ENSP00000293288	TP53,BAX
GO:2001244	Positive regulation of intrinsic apoptotic signaling pathway	2	60	2.21	0.0084	9606.ENSP00000269305,9606.ENSP00000293288	TP53,BAX
GO:0034103	Regulation of tissue remodeling	2	77	2.1	0.0126	9606.ENSP00000269305,9606.ENSP00000293288	TP53,BAX
GO:0006606	Protein import into nucleus	2	97	2	0.0176	9606.ENSP00000269305,9606.ENSP00000384849	TP53,CDKN1A
GO:2000379	Positive regulation of reactive oxygen species metabolic process	2	103	1.98	0.019	9606.ENSP00000269305,9606.ENSP00000384849	TP53,CDKN1A
GO:0032091	Negative regulation of protein binding	2	106	1.97	0.0196	9606.ENSP00000293288,9606.ENSP00000384849	BAX,CDKN1A
GO:0006289	Nucleotide-excision repair	2	111	1.95	0.0209	9606.ENSP00000256996,9606.ENSP00000269305	DDB2,TP53
GO:003030	B cell differentiation	2	110	1.95	0.0209	9606.ENSP00000269305,9606.ENSP00000293288	TP53,BAX

183							
GO:0032392	DNA geometric change	2	114	1.93	0.0218	9606.ENSP00000256996,9606.ENSP00000269305	DDB2,TP53
GO:0009267	Cellular response to starvation	2	154	1.8	0.0354	9606.ENSP00000269305,9606.ENSP00000384849	TP53,CDKN1A
GO:0006367	Transcription initiation from rna polymerase ii promoter	2	162	1.78	0.038	9606.ENSP00000293288,9606.ENSP00000384849	BAX,CDKN1A
GO:0050821	Protein stabilization	2	186	1.72	0.046	9606.ENSP00000269305,9606.ENSP00000384849	TP53,CDKN1A
GO:0030308	Negative regulation of cell growth	2	187	1.72	0.0462	9606.ENSP00000269305,9606.ENSP00000384849	TP53,CDKN1A

S08: PRIMER SEQUENCES

Primers against mRNA transcripts of *BAX*, *CDKN1A*, and *DDB2* genes were designed through NCBI's Primer-BLAST tool (Ye et al., 2012). *ACTB* (β-actin) and *GAPDH* (GlycerAldehyde 3-Phosphate DeHydrogenase) were used as housekeeping/endogenous gene controls.

Table S5. Sequence of primers used in the study.

Gene	Forward (F) and Reverse (R) primer sequences
BAX	F CCCGAGAGGTCTTTTTCCGAG<br>R CCAGCCCATGATGGTTCTGAT
CDKN1A	F CGATGGAACTTCGACTTTGTCA<br>R GCACAAGGGTACAAGACAGTG
DDB2	F ACCTCCGAGATTGTATTACGCC<br>R TCACATCTTCTGCTAGGACCG
ACTB	F CCCGAGAGGTCTTTTTCCGAG<br>R CCAGCCCATGATGGTTCTGAT
GAPDH	F TCAAGGCTGAGAACGGGAAG<br>R CGCCCCACTTGATTTTGGAG

S09: ASSESSMENT OF RNA QUANTITY

The quantity of the RNA samples was measured using a Thermo Scientific MultiScan GO UV-spectrophotometer and its accompanied software SkanIt (Cat No: 51119300). Briefly, the formula below was used to quantify the concentration of each RNA sample.

$$\boldsymbol{RNA\ Concentration}\ \left(\frac{\mu g}{mL}\right) = 40 * A_{260}$$

Based on this formula, the concentration of the RNA samples is provided in Table S6.

Table S6. Concentration of RNA samples.

	**U87**	**SH-SY5Y**
**CZ**	133.65/247.61	51.84/187.76
**RP**	71.14/162.35	38.59/274.90

All values are the averages of triplicates.
Values are in “treatment”/”vehicle control” format and in µg/mL unit.

Since the quantity of RNA is each treatment group is lower than its respective vehicle control, the RNA sample in each treatment group was dilute with RNase free water to the concentration of its corresponding vehicle control.

S10: MDM2PRED USAGE

MDM2pred is an online (open source) web application developed using the K Neighbors machine learning developed in the present study. The application is developed in Python programming language and utilizes the *Streamlit* library (https://github.com/streamlit/streamlit) for its web components.

The application accepts a SMILES string (single mode) or a list of SMILES in a CSV file (batch mode), it processes the SMILES of any chemical compound as input and provides the predicted pIC50/IC50 values against MDM2 as the output along with a 2D diagram and the IPUAC name of the input (only in the single mode). *MDM2pred* is accessible from http://ynlab.mu.edu.tr/tr/mdm2pred-6997 . The basic aspects and usage of the application are explained in Figure S1.

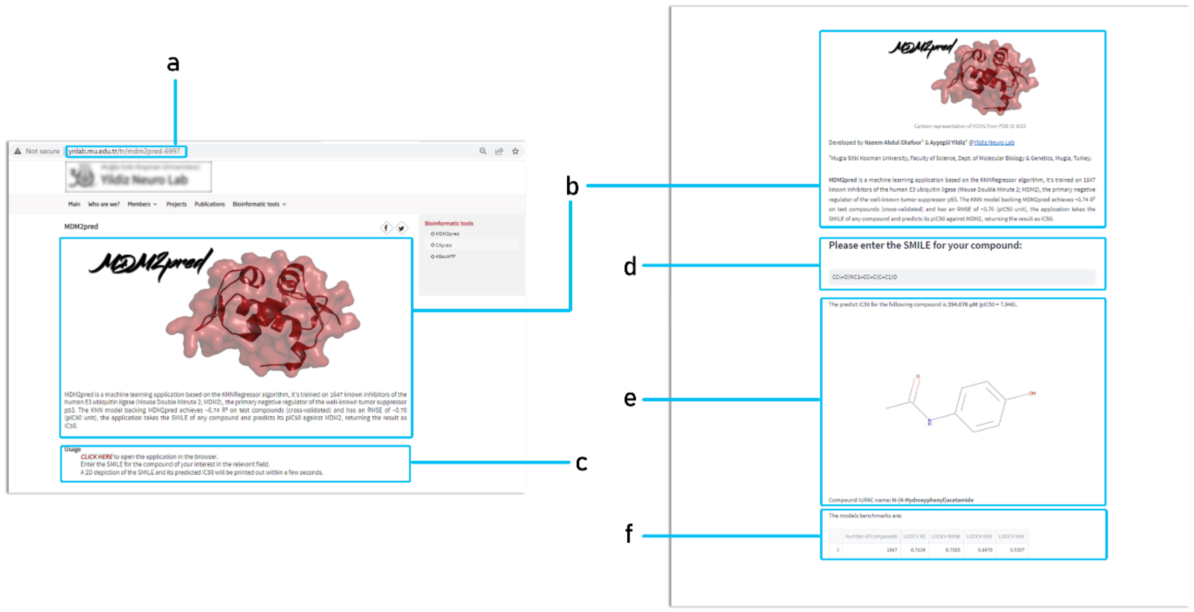

Figure S1. Access and usage of the *MDM2pred* application. (a) link to *MDM2pred*'s main page application; (b) general information and description about the application; (c) instructions for using the application; (d) input SMILES entry section; (e) result of the application, namely, the IC50, pIC50, 2D diagram of the compound, and It's IUPAC name; (f) performance matrix of the K Neighbors model.

The source files of the application and codes to reproduce its results are available at:
https://github.com/naeemmrz/MDM2pred

S11: PRINCIPLE COMPONENT ANALYSIS

The K-Neighbors algorithm (k-NN) marginally outperformed more complex algorithms like Random Forest, Gradient boosting, and even multilayer perceptron despite it being a relatively simple distance-neighbor based approach. While there is no consensus in what algorithm would work the best for what datasets, the distribution of the dataset and feature space plays a critical role. We attempted to explain the outstanding performance of a relatively simple algorithm for our dataset could be (at least partially) attributed to the distribution of the data. K-NN performs exceptionally when the data has a natural clustering or grouping pattern, i.e., K-NN can be effective in identifying similar datapoints and make accurate predictions based on the nearest neighbors. We tested this by performing a k-mean clustering of the MDM2 dataset, the optimal value of k via the silhouette score in Python environment. The best value of k was identified as 8 in this case. We then performed Principle Component Analysis (PCA) and visualized the first four Principle Components (PCs) which explained roughly half of the variance in the dataset (55.73%), we also colored the datapoints by cluster in each of the PC vs PC plots. As it can be observed, along several PCs, it is quite apparent that certain molecules cluster together and form local clusters.

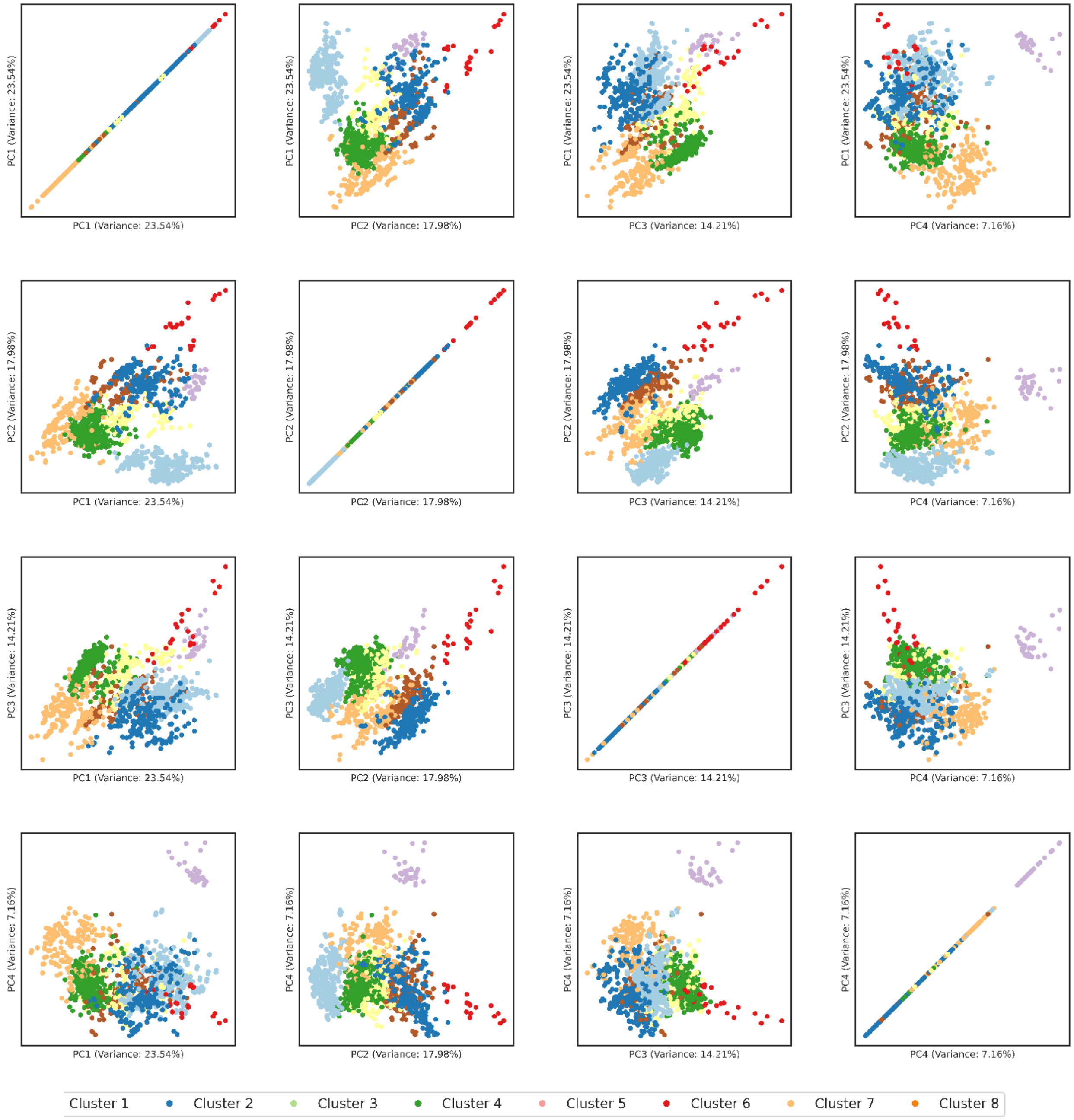

Figure S2. Principle Component Analysis (PCA) and K-means clustering. PCA of the first 4 PCs against each other, samples are colored based on the clusters they belong to according to the K-means clustering.

S12: APPLICABILITY DOMAIN

These are the results for the Applicability Domain (AD) assessment using 2P/n instead of the recommended 3P/n to calculate the leverage threshold (h*). As illustrated below, the h* was calculated as 0.37, according to this threshold 137 samples were considered as outliers. These were removed and the model was trained and evaluated via 10-fold cross-validation on the normal (i.e. samples within it's AD only). The results are provided in the table below, the cross-validated R^2 is slightly higher but the RMSE and MAE didn't improve.

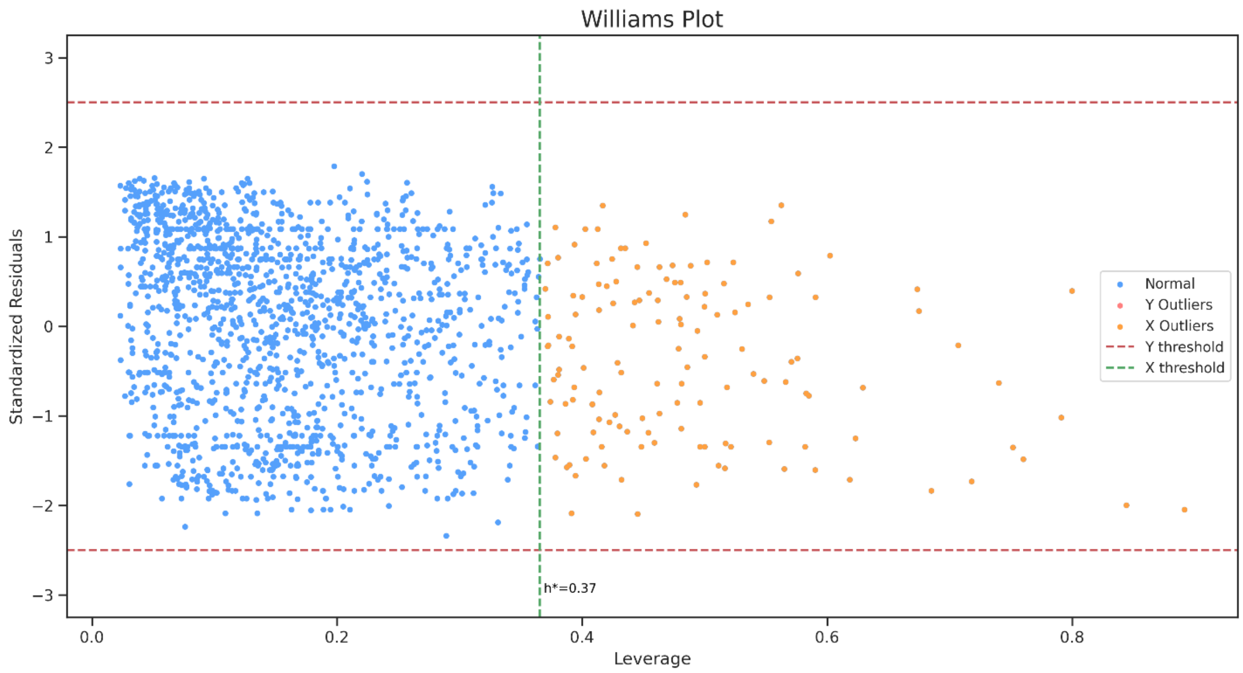

Figure S3. Williams plot for describing the applicability domain of the K-Neighbors models. Area enclosed by red and green dotted lines represent the model's AD, datapoints outside the enclosed space are outliers. No Y outliers were identified. h* calculated using 2P/n formula.

Table S7: Performance of the K-Neighbors after removing the outliers shown In Figure S2.

Iteration	Test R^2	Test RMSE	Test MAE
**1**	0.73	0.71	0.51
**2**	0.79	0.63	0.48
**3**	0.68	0.77	0.57
**4**	0.78	0.65	0.47
**5**	0.83	0.56	0.44
**6**	0.73	0.73	0.48
**7**	0.73	0.68	0.52
**8**	0.77	0.68	0.50
**9**	0.74	0.77	0.55
**10**	0.77	0.69	0.51
**Average**	0.76	0.69	0.50
**Standard Deviation**	0.04	0.06	0.04

The hyperparameters of the model were: “algorithm”, “auto”; “leaf_size”, 30; “metric”, “minkowski”; “metric_params”, None; “n_neighbors”, 5; “p”, 2; “weights”, ‘uniform’.
